# DeepISLES: a clinically validated ischemic stroke segmentation model from the ISLES'22 challenge

**DOI:** 10.1038/s41467-025-62373-x

**Published:** 2025-08-09

**Authors:** Ezequiel de la Rosa, Mauricio Reyes, Sook-Lei Liew, Alexandre Hutton, Roland Wiest, Johannes Kaesmacher, Uta Hanning, Arsany Hakim, Richard Zubal, Waldo Valenzuela, David Robben, Diana M. Sima, Vincenzo Anania, Arne Brys, James A. Meakin, Anne Mickan, Gabriel Broocks, Christian Heitkamp, Shengbo Gao, Kongming Liang, Ziji Zhang, Md Mahfuzur Rahman Siddiquee, Andriy Myronenko, Pooya Ashtari, Sabine Van Huffel, Hyunsu Jeong, Chiho Yoon, Chulhong Kim, Jiayu Huo, Sebastien Ourselin, Rachel Sparks, Albert Clèrigues, Arnau Oliver, Xavier Lladó, Liam Chalcroft, Ioannis Pappas, Jeroen Bertels, Ewout Heylen, Juliette Moreau, Nima Hatami, Carole Frindel, Abdul Qayyum, Moona Mazher, Domenec Puig, Shao-Chieh Lin, Chun-Jung Juan, Tianxi Hu, Lyndon Boone, Maged Goubran, Yi-Jui Liu, Susanne Wegener, Florian Kofler, Ivan Ezhov, Suprosanna Shit, Moritz R. Hernandez Petzsche, Michael Müller, Bjoern Menze, Jan S. Kirschke, Benedikt Wiestler

**Affiliations:** 1 Department of Quantitative Biomedicine, University of Zurich, Zurich, Switzerland; 2 Department of Informatics, Technical University of Munich, Munich, Germany; 3 icometrix, Leuven, Belgium; 4 ARTORG Center for Biomedical Research, University of Bern, Bern, Switzerland; 5 Department of Radiation Oncology, University Hospital Bern, University of Bern, Bern, Switzerland; 6 Chan Division of Occupational Science and Occupational Therapy, University of Southern California, Los Angeles, CA USA; 7 Stevens Neuroimaging and Informatics Institute, Keck School of Medicine, University of Southern California, Los Angeles, CA USA; 8 Support Center of Advanced Neuroimaging (SCAN), University Institute of Diagnostic and Interventional Neuroradiology, Inselspital, Bern Switzerland; 9 University Institute of Diagnostic and Interventional Neuroradiology, University Hospital Bern, Inselspital, University of Bern, Bern, Switzerland; 10 Diagnostic and Interventional Neuroradiology, CIC-IT 1415, CHRU de Tours, Tours, France; 11 Department of Diagnostic and Interventional Neuroradiology, University Medical Center Hamburg-Eppendorf, Hamburg, Germany; 12 Department of Medical Imaging, Radboud University Medical Center, Institute for Health Sciences, Nijmegen, The Netherlands; 13 Deepwise AI Lab, Beijing, China; 14 Beijing University of Posts and Telecommunications, Beijing, China; 15 School of Computing and Augmented Intelligence, Arizona State University, Tempe, AZ USA; 16 NVIDIA, Santa Clara, CA USA; 17 Department of Electrical Engineering (ESAT), STADIUS Center for Dynamical Systems, Signal Processing, and Data Analytics, KU Leuven, Leuven Belgium; 18 Graduate School of Artificial Intelligence, Pohang University of Science and Technology (POSTECH), Pohang, Republic of Korea; 19 Department of Electrical Engineering, Pohang University of Science and Technology (POSTECH), Pohang, Republic of Korea; 20 Department of Convergence IT Engineering, Pohang University of Science and Technology (POSTECH), Pohang, Republic of Korea; 21 Medical Device Innovation Center, Pohang University of Science and Technology (POSTECH), Pohang, Republic of Korea; 22 Department of Mechanical Engineering, Pohang University of Science and Technology (POSTECH), Pohang, Republic of Korea; 23 Department of Medical Science and Engineering, Pohang University of Science and Technology (POSTECH), Pohang, Republic of Korea; 24 School of Biomedical Engineering & Imaging Sciences, King’s College London, London, UK; 25 Institute of Computer Vision and Robotics, University of Girona, Girona, Spain; 26 Wellcome Centre for Human Neuroimaging, University College London, London, UK; 27 Laboratory of Neuro Imaging, Stevens Institute for Neuroimaging and Informatics, Keck School of Medicine, University of Southern California, Los Angeles, CA USA; 28 Department of Electrical Engineering (ESAT), Processing Speech and Images (PSI), KU Leuven, Leuven, Belgium; 29 CREATIS, Université Lyon1, CNRS UMR5220, INSERM U1206, INSA-Lyon, Villeurbanne, France; 30 National Heart and Lung Institute, Faculty of Medicine, Imperial College London, London, UK; 31 Centre for Medical Image Computing, Department of Computer Science, University College London, London, UK; 32 Department of Computer Engineering and Mathematics, University Rovira I Virgili, Tarragona, Spain; 33 Department of Medical Imaging, China Medical University Hsinchu Hospital, Hsinchu, Taiwan Republic of China; 34 Department of Medical Biophysics, University of Toronto, Toronto, Canada; 35 Hurvitz Brain Sciences Research Program, Sunnybrook Research Institute, Toronto, Canada; 36 Department of Automatic Control Engineering, Feng Chia University, Taichung, Taiwan Republic of China; 37 Department of Neurology, University Hospital of Zurich, Zurich, Switzerland; 38 University of Zurich, Zurich, Switzerland; 39 Helmholtz AI, Helmholtz Munich, Neuherberg, Germany; 40 Department of Diagnostic and Interventional Neuroradiology, Klinikum rechts der Isar, Technical University of Munich, Munich, Germany; 41 TranslaTUM, Center for Translational Cancer Research, Technical University of Munich, Munich, Germany; 42 AI for Image-Guided Diagnosis and Therapy, School of Medicine & Health, Technical University of Munich, Munich, Germany

**Keywords:** Biomedical engineering, Stroke, Computer science

## Abstract

Diffusion-weighted MRI is critical for diagnosing and managing ischemic stroke, but variability in images and disease presentation limits the generalizability of AI algorithms. We present *DeepISLES*, a robust ensemble algorithm developed from top submissions to the 2022 Ischemic Stroke Lesion Segmentation challenge we organized. By combining the strengths of best-performing methods from leading research groups, *DeepISLES* achieves superior accuracy in detecting and segmenting ischemic lesions, generalizing well across diverse axes. Validation on a large external dataset (*N* = 1685) confirms its robustness, outperforming previous state-of-the-art models by 7.4% in Dice score and 12.6% in F1 score. It also excels at extracting clinical biomarkers and correlates strongly with clinical stroke scores, closely matching expert performance. Neuroradiologists prefer *DeepISLES*’ segmentations over manual annotations in a Turing-like test. Our work demonstrates *DeepISLES’* clinical relevance and highlights the value of biomedical challenges in developing real-world, generalizable AI tools. *DeepISLES* is freely available at https://github.com/ezequieldlrosa/DeepIsles.

## Abstract (web summary)

Accurate segmentation of ischemic stroke lesions from brain MRI is crucial for timely diagnosis and treatment planning. Here, the authors present DeepISLES, an AI ensemble for stroke MRI analysis that outperforms previous methods and matches expert radiologist performance in identifying stroke lesions.

## Introduction

Brain imaging is crucial to evaluate tissue viability and fate in ischemic stroke. Magnetic resonance imaging (MRI) supports physicians through various stages of the disease. It helps define the optimal reperfusion treatment, unveils the stroke etiology, and sheds light on prognostic clinical outcomes^1^. Diffusion-weighted imaging (DWI) is considered the current gold standard for imaging the ischemic core^2,3^. Although imperfect, DWI is the only imaging technique reliably demonstrating parenchymal injury within minutes to hours from the stroke onset^4^. Currently, deep learning algorithms are revolutionizing medical imaging, demonstrating unprecedented performance across multiple radiological tasks. Segmentation of ischemic stroke tissue using deep learning has been proposed in different works^5–9^. The complexity of the task lies in multiple sources of variability that involve image- (e.g., driven by center- and scanner-specific MRI acquisition differences, artifacts mimicking ischemic lesions^10^, time-dependent DWI signaling^4,11^, etc.), patient- (e.g., age^12^) and disease-specific characteristics (such as the subtype of stroke and its etiology^13^). Little is known, however, about the real-world transferability potential of deep learning algorithms for ischemic stroke segmentation, their generalization towards diverse cohorts and image characteristics, and their ultimate clinical utility.

Biomedical challenges are international competitions aiming to benchmark task-specific algorithms under controlled settings^14^. The organization of medical image challenges has rapidly grown, enabling to tackle problems related to diverse organs, tasks (e.g., lesion detection or anatomy segmentation), and image modalities (such as MRI, CT, among others)^15–19^. Challenges are now considered a de facto gold standard for algorithm comparison by the research community^20^ and have also been adopted by the Radiological Society of North America (RSNA) (https://www.rsna.org/rsnai/ai-image-challenge). Segmentation of stroke lesions from MRI has not been an exception, and the number of methods devised targeting this task considerably increased following the 2015 Ischemic Stroke Lesion Segmentation (ISLES) challenge^21^. ISLES’15 is considered a reference evaluation tool for the segmentation of brain ischemia. In the past few years, studies highlighting the strengths and weaknesses of challenge organization emerged, providing good implementation practices^14,22,23^. Such initiatives considerably improved the quality of current challenges regarding execution, interpretation, fairness, transparency, and reproducibility.

Biomedical challenges, when properly designed, are powerful. They operate as international problem-solving sprints that involve leading researchers worldwide. Therefore, we take advantage of such an event to rapidly prototype and identify candidate ischemic stroke segmentation algorithms. We hypothesized that (1) a challenge might yield an algorithm or a strategy that reliably detects and segments brain ischemia under real-world, heterogeneous data scenarios, and (2) such an algorithm may generalize beyond the challenge context to real-world data, thus becoming relevant to downstream clinical analysis. We organized the ISLES’22 challenge during the 2022 International Conference on Medical Image Computing and Computer-Assisted Intervention (MICCAI)^24,25^ to test these hypotheses. ISLES’22 builds on top of the experience gained from the earlier ISLES’15, overcoming some of its drawbacks by adhering to current challenge standards^22,23^, by using a standardized platform^24^ for the fair assessment of software solutions, and by including more than six times the number of patients than ISLES’15.

In this paper, we introduce *DeepISLES*, a robust and ready-to-use deep learning algorithm for ischemic stroke segmentation developed from algorithmically diverse submissions to the ISLES’22 challenge. *DeepISLES* exhibits strong generalization capabilities across various data variability dimensions and achieves performance levels comparable to expert radiologists on a large external real-world dataset. Furthermore, our study underscores the potential of biomedical challenges to produce models that extend beyond the challenge dataset itself, emphasizing their real-world clinical relevance and bridging the gap between biomedical research and clinical practice. To promote wider use, validation, and adoption, *DeepISLES* is publicly available in multiple formats including a standalone application with a graphical user interface, a web-based service, a Docker image, and its source Git repository. All versions can be accessed from https://github.com/ezequieldlrosa/DeepIsles^26^.

## Results

### DeepISLES: a robust algorithm derived from leading ISLES’22 submissions

**Fig. 1:**
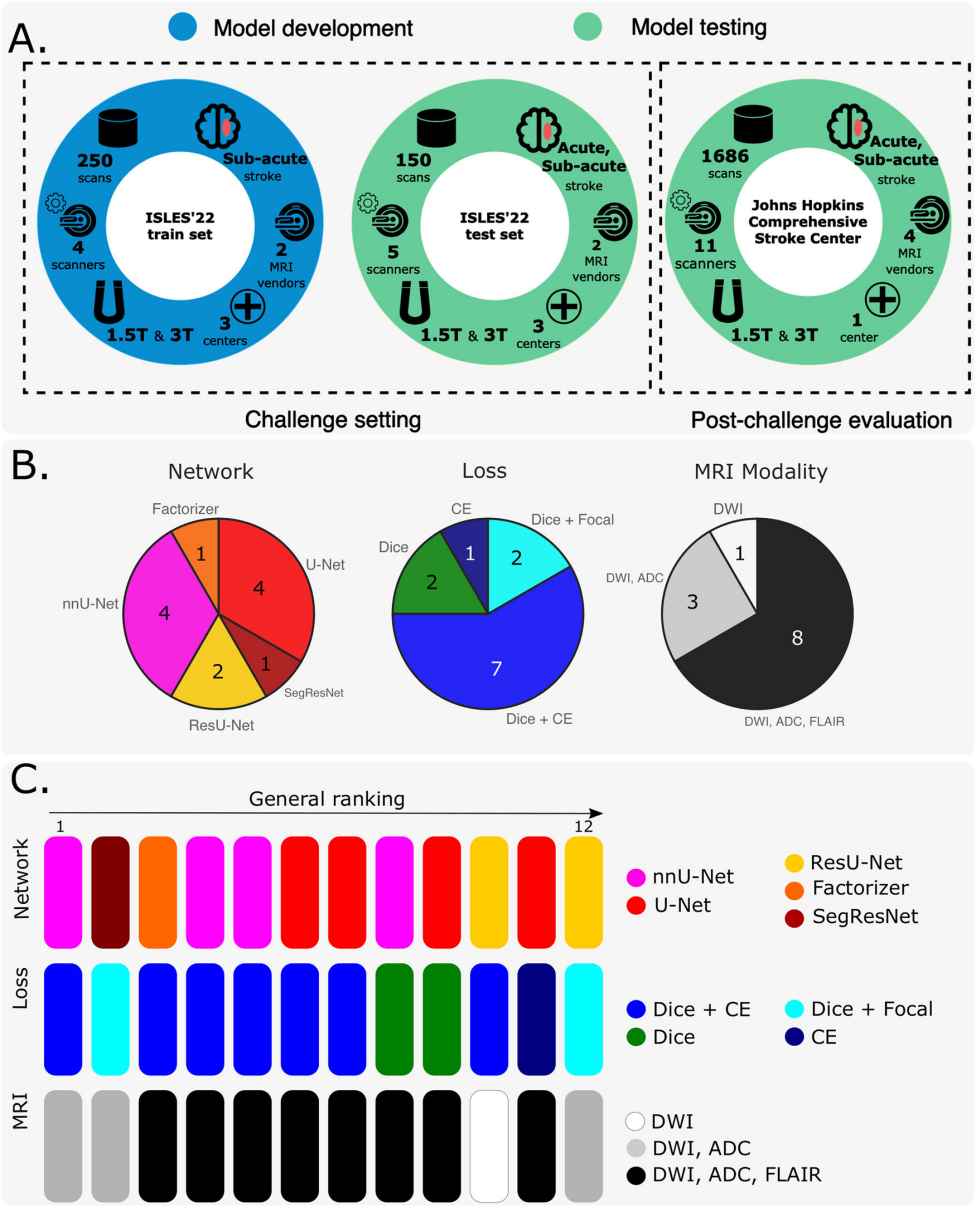
Overview of the ISLES’22 challenge and post-challenge experimental design, including the developed algorithmic solutions. **A** Challenge and post-challenge phases and datasets. **B** Summary of algorithmic solutions stratified by network architecture, loss function, and input modalities. **C** Challenge leaderboard stratified by network architecture, loss function, and input modalities. CE Cross-entropy.

During the ISLES’22 challenge (May-August 2022), a total of 476 participants registered, and 325 dataset downloads were recorded by the closing date. Twenty teams validated their algorithms on the remote servers during the *sanity-check* phase, leading to 15 deep learning submissions in the final *test-phase*, of which 12 met the participation criteria^25^. Details about these solutions are available in supplementary material section 1. Figure 1(A) provides an overview of the challenge structure and its phases. In the model development (a.k.a. *train*) phase, teams leveraged labeled datasets to develop algorithmic solutions, which were subsequently assessed on undisclosed data during the model testing phase. The challenge datasets were intentionally left raw and unprocessed to simulate real-world scenarios, compelling participants to design end-to-end algorithmic methods. The external dataset utilized for evaluating the performance of our proposed algorithm in a post-challenge, “real-world” setting is also summarized in Fig. 1(A).

The ISLES’22 challenge represented a pivotal opportunity to foster a wide array of technical methods and learning strategies, achieving a level of heterogeneity that would be difficult for an individual researcher to accomplish independently. A fingerprint of the participating algorithms is presented in Fig. 1(B). The most prevalent architectures included nnU-Net^27^ and U-Net-like^28,29^ neural networks, though other approaches were also submitted. Similarly, various loss functions were employed, with Dice combined with categorical cross-entropy being the most commonly chosen. Figure 1(C) provides a visual ranking of the submitted methods. Notably, the top three teams employed diverse algorithmic strategies, differing in deep learning architectures, loss functions, and even model inputs. The winner of the ISLES’22 challenge, algorithm SEALS, led the leaderboard in detection metrics, specifically the lesion-wise F1 score and absolute lesion count difference (Table S2.1 and Fig. S2.1 in supplementary material). The lesion-wise F1 score quantifies detection performance at the individual lesion level, reflecting the model’s ability to correctly localize and identify each distinct lesion. In contrast, the Dice coefficient measures spatial overlap at the voxel level and is therefore biased toward larger lesions; as a result, failure to detect small lesions has limited effect on the Dice score but leads to a marked decrease in the lesion-wise F1 score. Assessing both, therefore, helps to paint a clearer picture of model performance. The second-ranked method, NVAUTO, excelled in segmentation-derived metrics, leading in both Dice coefficient and absolute volume difference. The third position was jointly held by two algorithms (SWAN and PAT—see section 3 in the supplementary material); however, post-challenge analyses exploring variations in ranking methodologies ultimately favored the algorithm SWAN (supplementary material section 4).

Inspired by the diversity of the submitted methods, we sought to leverage these varied approaches to develop a comprehensive ensemble solution for ischemic stroke segmentation, aiming to combine the strengths while simultaneously addressing the limitations of individual algorithms. Therefore, in a post-challenge scenario and in collaboration with participants from the three top-ranked teams, we developed *DeepISLES*, a comprehensive algorithm for stroke lesion segmentation. *DeepISLES* facilitates end-to-end processing of scans, starting from native image series obtained in clinical settings (possibly even in DICOM format). When compared with the other methods submitted to the challenge, *DeepISLES* achieved the highest position on the (post-challenge) leaderboard (supplementary material section 5), demonstrating exceptional performance across all evaluated metrics. The leaderboard is presented in Table S2.1 and Fig. S2.1 (supplementary material section 2). Additional statistics regarding the challenge rankings, derived from a thousand bootstrap experiments, are detailed in the sections 3–5 in the supplementary material. These include analyses of *DeepISLES*’ performance, ranking stability, robustness to ranking methodologies, and inter-algorithmic comparisons.

### From a MICCAI challenge to a real-world solution

We aim to test the hypothesis that a method derived from a challenge might indeed be relevant for real-world, downstream clinical tasks. The hypothesis is tested in two steps. First, we evaluate *DeepISLES* over diverse clinical and imaging scenarios of the challenge test set to expose potential suboptimal or biased performance toward specific data subgroups. Disease and imaging confounders such as the imaging center, ischemic lesion size, stroke phase, type of stroke pattern/configuration, and vascular territory affected are considered. Second, *DeepISLES* is evaluated on a large, external stroke dataset to assess its lesion segmentation performance and clinical relevance in real-world settings, and is compared with a state-of-the-art model trained and validated on the same dataset. In the following subsections, we focus on each of these aspects.

#### Can DeepISLES identify ischemic lesions in scans from an unseen imaging center?

**Fig. 2:**
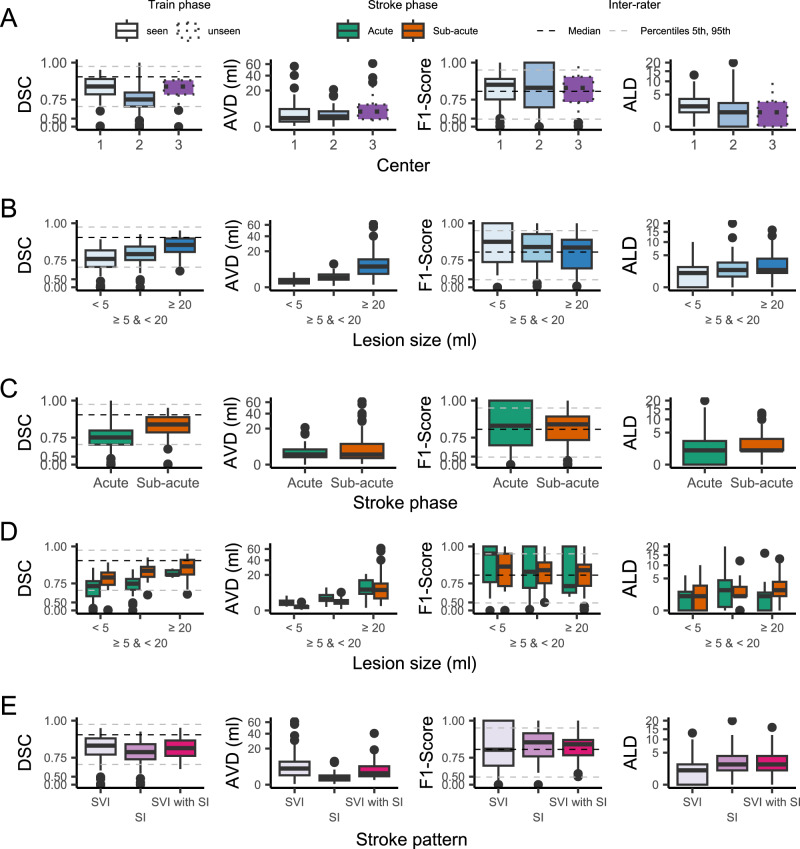
Test set performance metrics obtained by *DeepISLES.* **A** Performance by imaging center. Data is grouped by the center where the images come from (*#*1, *#*2, or *#*3) and by a *seen* or *unseen* label indicating if images from the same center were used for training the models. **B** Performance by lesion size. **C** Performance by stroke phase (acute or sub-acute). **D** Performance by the stroke phase (acute or sub-acute) grouped by lesion size. **E** Performance by stroke pattern subgroups, including single vessel infarcts, scattered infarcts based on micro-occlusions, and single vessel infarcts with accompanying scattered infarcts. All boxplots are based on a sample size of *N* = 150. Boxes show the interquartile range (IQR; 25th–75th percentiles), the center line marks the median, whiskers span values within 1.5 × IQR, and points beyond are displayed as outliers. 5th, 50th, and 95th inter-rater variability percentiles are plotted in dashed lines for Dice and F1 score. SVI: single vessel infarct; SI: scattered infarcts based on micro-occlusions; SVI with SI: single vessel infarct with accompanying scattered infarcts. DSC Dice Similarity Coefficient; F1 score lesion-wise F1 score; AVD absolute volume difference; ALD absolute lesion count difference. y-axes are displayed using a non-linear scale to enhance data visibility. Source data are provided as a Source Data file.

Algorithmic robustness to out-of-domain data (unseen during the model’s development phase) is crucial for evaluating the algorithm’s transferability to real-world centers. Figure 2(A) shows how *DeepISLES* performs over test-phase data from seen (centers *#*1 and *#*2) and unseen (center *#*3) centers during the development of the algorithm. The distribution of the metrics obtained over the unseen center *#*3 is similar to the metric’s distribution obtained over the seen center *#*1, suggesting an overall good generalization to new center data (Dice *p*-value = 0.73, F1 score *p*-value = 0.60, ALD *p*-value = 0.42, AVD *p*-value = 0.08, Wilcoxon rank-sum tests). The performance obtained over the seen center *#*2 is lower in terms of Dice score compared to the center *#*1 (p-value < 0.001, Wilcoxon rank-sum test). The F1 score, AVD and ALD metrics are similar between centers *#*1 and *#*2 (F1 score *p*-value = 0.60, ALD *p-*value = 0.26, AVD *p*-value = 0.28, Wilcoxon rank-sum tests). The lower Dice scores in center *#*2 can be explained by two cohort confounders. Firstly, the scans from center *#*2 include smaller lesion volumes than scans from the other two centers^30^ (*p*-value = 0.039 for center *#*1 vs center *#*2, *p*-value = 0.001 for center *#*2 vs center *#*3, *p*-value = 0.56 for center *#*1 vs center *#*3, Wilcoxon rank-sum tests). Figure S2.1 (supplementary material section 2) shows the non-linear, monotonic correlation between lesion size and Dice scores for the test set data. The fact that larger objects (i.e., brain lesions) benefit from higher Dice scores is well known and, therefore, is associated with the found results^31,32^. Secondly, unlike the train phase data, which considers scans acquired in the sub-acute stroke phase after reperfusion treatment, the test set scans from center *#*2 are acquired in the acute stroke phase, before the patient’s reperfusion treatment, which is known to be a harder task for the algorithms^21^. The following sections analyze both of the aforementioned confounding factors. It is worth noting a potential third confounder related to the imbalance in training data (4:1 for centers #1:#2, as shown in Table 3). However, we chose to disregard this factor because the model demonstrated strong generalization abilities to scans from an entirely new center.

#### Can DeepISLES identify stroke lesions of variable size?

We test *DeepISLES* performance over lesions smaller than 5 ml, lesions larger than or equal to 5 ml but smaller than 20 ml, and lesions larger than or equal to 20 ml. The performance metrics over these groups are shown in Fig. 2(B). The relationship between lesion size and metrics like Dice, AVD, and ALD is anticipated; larger lesions typically yield higher Dice values, while AVD and ALD tend to increase with lesion size. Despite this, *DeepISLES* demonstrates strong generalization performance across varying ischemic lesion sizes, achieving comparable ischemia detectability as measured by F1 scores. Furthermore, there is a high volumetric agreement between the algorithm and ground truth masks for all lesion sizes, with Pearson *r* = 0.98 for the entire test set, *r* = 0.87 for lesions smaller than 5 ml, *r* = 0.90 for lesions equal to or larger than 5 ml but smaller than 20 ml, and *r* = 0.96 for lesions larger than or equal to 20 ml. *DeepISLES* demonstrated robustness towards diverse stroke lesion sizes. Figure S3 (supplementary material section 2) shows the volumetric agreement between the ground truth and *DeepISLES* predictions for different lesion sizes.

#### Can DeepISLES identify ischemia in acute and sub-acute scans?

In order to assess the generalizability of the algorithm to diverse stroke phases, we split the challenge test set scans into two subgroups: acute (i.e., scans that were acquired as part of the acute stroke diagnostics, within a few hours of stroke onset and before thrombectomy treatment) and sub-acute (i.e., scans acquired within days after stroke onset and after thrombectomy treatment). In Fig. 2(C), the performance metrics for the two subgroups are shown. It can be observed that the algorithm predicts acute scans with similar lesion-wise F1 scores (*p*-value = 0.45, Wilcoxon rank-sum test) but with lower Dice scores (*p*-value < 0.001, Wilcoxon rank-sum test) than the sub-acute group. On the contrary, the performance in terms of absolute volume difference and lesion count difference is better for the acute stroke group than for the sub-acute stroke group. These trends are partially due, as earlier introduced, to the lower overall lesion size of the acute group compared to the sub-acute one. However, it remains unclear if the lesion size is the sole responsible for this behavior and what the role of the stroke phase is, especially considering that the training dataset exclusively comprises sub-acute scans. To get insights about it, the test set is grouped considering both the variables: lesion size and the stroke phase. Figure 2(D) shows the corresponding performance metrics. It can be seen that even when splitting the scans using matched lesion-size groups, the lower Dice and AVD performance persists. This indicates that the decline in performance may be attributed to the earlier acute phase of the disease, which was not included in the models’ development phase. Moreover, in Figure S2.3 (supplementary material section 2), volumetric scatter plots and Bland-Altman plots are shown. There is an excellent agreement between the ground truth and *DeepISLES*-predicted lesion volumes for both groups (Pearson *r* = 0.99 and *r* = 0.98 for the acute and sub-acute groups, respectively).

#### Can DeepISLES predict different stroke clinical patterns?

We evaluate whether *DeepISLES* performs reliably under diverse stroke lesion patterns. With this aim, the test-phase scans are classified into three stroke sub-groups: single vessel infarcts (SVI), scattered infarcts based on micro-occlusions, and SVI with accompanying scattered infarcts. First, looking for a potential bias towards a specific stroke subgroup, the algorithm performance is evaluated in a subgroup-stratified approach. Second, we frame the problem into a clinically relevant question: Can *DeepISLES* identify the stroke subgroup? In Fig. 2(E), the lesion segmentation performance of the algorithm is shown for each metric and for each type of stroke pattern. A similar performance in terms of Dice score and F1 score for the different stroke subgroups can be appreciated. The lower AVD seen in the group *scattered infarcts based on micro-occlusions* is due to the fact that emboli are typically smaller lesions than SVI and, therefore, this group includes scans with smaller lesion volumes (percentiles [5th, 50th, 95th] of [0.9, 4.4, 36.9] ml) compared to SVI lesions (percentiles [5th, 50th, 95th] of [1.5, 24.9, 137.9] ml) and SVI with scattered infarcts (percentiles [5th, 50th, 95th] of [6.4, 24.5, 134.5] ml). Moreover, the SVI group exhibits lower ALD since their scans have, by definition (see Section “Methods”), less disconnected ischemic lesions. Next, the algorithm’s capability to predict each scan’s stroke subgroup is evaluated. Prediction of the stroke subgroup is generated by applying a heuristic rule defined by radiologists to the stroke masks (details of the classification criteria are available in the *Methods* section). Results for each of the top-3 ranked methods, as well as for *DeepISLES*, are summarized in Table 1. The most challenging scans to identify are the ones exhibiting an SVI with accompanying scattered infarcts. It is also worth noting that the solution submitted by the team NVAUTO (which ranked second in the challenge) yields a better stroke pattern classification performance than the other challenge submissions. The best overall performance is obtained by *DeepISLES*, which remarkably outperforms any single challenge solution (balanced accuracy of 86.9% for the ensemble method compared to the 78.9% achieved by the team ‘NVAUTO’), thus demonstrating a strong capability to classify stroke sub-groups.

**Table 1:** Algorithmic classification performance of stroke patterns (above) and vascular territories (below)

	Stroke pattern			
	SVI (*N*=62)	SI based on micro-occlusions (*N* = 48)	SVI with accompanying SI (*N* = 38)	All stroke (*N* = 148)
Team	F1 score			Balanced Accuracy
SEALS	87.6	75.6	68.8	78.1
NVAUTO	**88.1**	78.6	68.1	78.9
SWAN	85.0	75.9	68.2	76.2
DeepISLES	87.6	**91.8**	**81.6**	**86.9**

	Vascular territory					
	MCA (*N* = 97)	PCA (*N* = 23)	ACA (*N* = 4)	Pons/Medula (*N* = 4)	Cerebellum (*N* = 20)	All stroke (*N* = 148)
Team	F1 score					Balanced Accuracy
SEALS	97.9	93.3	88.9	80.0	**97.6**	97.4
NVAUTO	97.9	**95.7**	80.0	88.9	97.4	97.3
SWAN	96.8	93.3	66.7	**100.0**	**97.6**	92.2
DeepISLES	**98.4**	93.3	**100.0**	88.9	**97.6**	**97.6**

*DeepISLES* is notably superior to any individual solution in identifying the stroke pattern and the vascular territory. All metrics are reported in percentage values. The best results are highlighted in bold. Source data are provided as a Source Data file.

*SVI* single vessel infarct, *SI* scattered infarcts, *MCA* middle cerebral artery, *ACA* anterior cerebral artery, *PCA* posterior cerebral artery.

#### Can DeepISLES identify the ischemic vascular territory?

In this experiment, we evaluate whether the algorithms can identify the affected vascular territory among the middle, anterior, and posterior cerebral arteries, the vasculature of the cerebellum, and the vasculature of the pons/medulla. To this end, we quantify through the predicted lesion masks the lesion load per vascular territory from a reference atlas of vascular territories. Then, the territory with the absolute largest lesion volume is considered the most affected territory and is compared with the vascular territory affected in the ground truth masks. In Table 1, the results from this experiment are shown. Overall, the challenge algorithms accurately predict most vascular territories. The solution submitted by the winner of the challenge (team SEALS) obtains the best performance in this task when compared to the other teams. The best overall performance is obtained, again, with *DeepISLES*, yielding a remarkable 97.6% of balanced accuracy and F1 score  > 88% for each considered vascular territory. These results show that the proposed algorithm can accurately identify the impacted vascular territory.

### Inter-rater performance and qualitative analysis in a Turing-like test

**Fig. 3:**
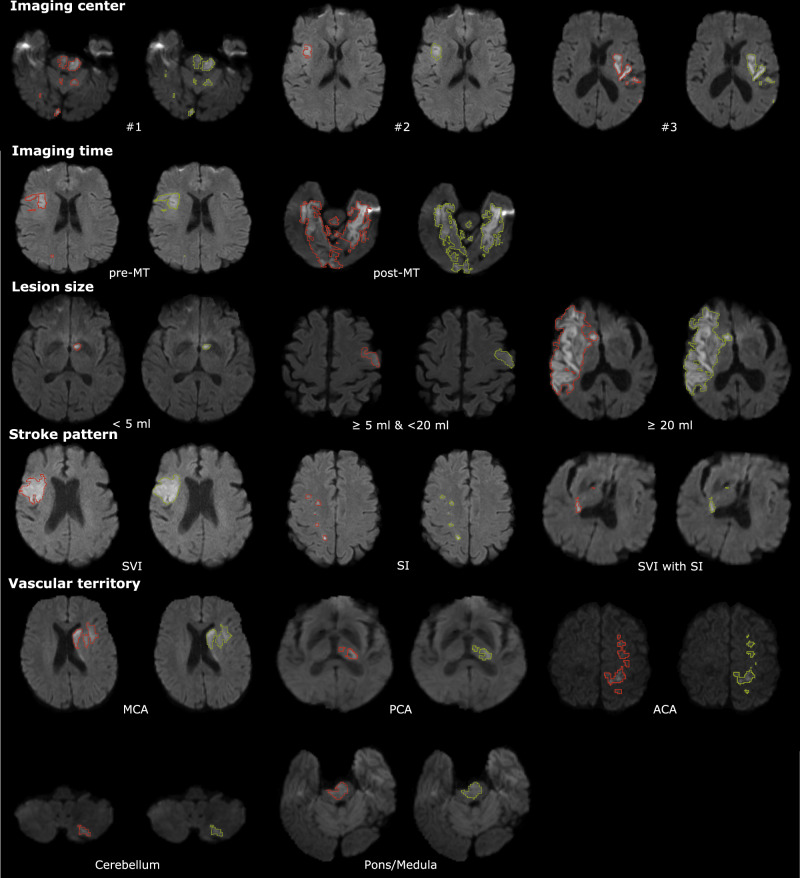
Lesion ground truth (red) and *DeepISLES* predictions (green) for scans from the ISLES’22 test set. Note that we selected the scans with median Dice scores (and not the best performing scans) to paint a realistic picture. Model outputs closely align with expert annotations across various types of stroke patterns and configurations. Results are grouped by healthcare center, imaging time, lesion size, stroke pattern, and vascular territory affected. MT mechanical thrombectomy; SVI single vessel infarct; SI scattered infarcts based on micro-occlusions. SVI with SI single vessel infarct with accompanying scattered infarcts. MCA middle cerebral artery; ACA anterior cerebral artery; PCA posterior cerebral artery.

Two expert neuroradiologists annotated ten randomly sampled scans from the ISLES’22 training set^30^. When comparing their delineations against the ground truth masks, they achieved a median ± interquartile range Dice score of 0.92  ± 0.16 and a lesion-wise F1 score of 0.82 ± 0.30. Over the entire test set, *DeepISLES* yielded a Dice score of 0.82  ± 0.12 and an F1 score of 0.86 ± 0.21. Besides, Fig. 3 shows model predictions for scans with median Dice score (to avoid “cherry-picking") for diverse stroke scenarios. The visual results of the predicted ischemic masks suggest that the algorithmic predictions (green delineations) closely follow the manually segmented lesions (red delineations), highlighting *DeepISLES’* capability to generalize to diverse types of stroke patterns and configurations. The quantitative and qualitative results suggest robustness towards heterogeneous clinical and imaging scenarios.

**Fig. 4:**
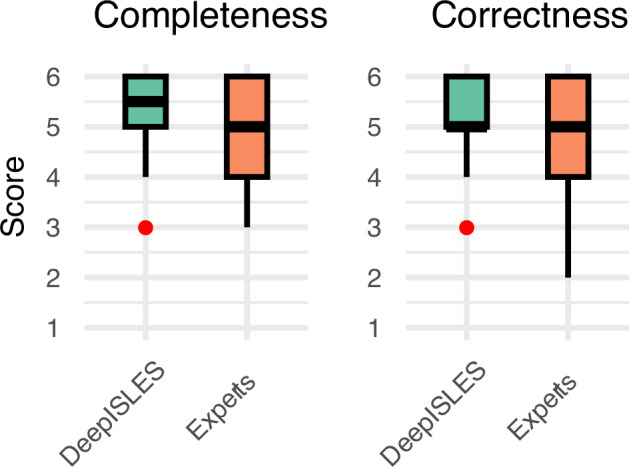
Qualitative lesion segmentation results obtained in a Turing-like test. Neuroradiologists prefer lesions delineated by *DeepISLES* over manual expert delineations (sample size *N* = 150). Score values range between 1 and 6 (worst and best quality scenarios, respectively). Boxes show the interquartile range (IQR; 25th-75th percentiles), the center line marks the median, whiskers span values within 1.5 × IQR, and points beyond are displayed as outliers.

In a Turing-like test, nine experienced radiologists rated the stroke segmentation quality of the ISLES’22 test data. Radiologists received forty or forty-one randomized images, with each image being delineated either by an expert or by the ensemble algorithm, and were asked to rate the *completeness* and *correctness* of the lesion masks in a 1-to-6 (worst-to-best) scale. Boxplots of this experiment are shown in Fig. 4. Interestingly, the ensemble algorithm exhibits statistically significantly higher ratings than the experts (*p*-value = 0.02 when considering the segmentation *completeness* and *p*-value < 0.001 when considering the segmentation *correctness*, Wilcoxon signed-rank tests). The observation that experts find deep learning segmentations to be qualitatively superior to manually traced ones is unsurprising, given that similar findings were reported in prior research^33^.

### External validation and clinical relevance

We tested *DeepISLES* in the largest available acute and early sub-acute ischemic stroke cohort (*N* = 1685), retrieved from the Johns Hopkins Comprehensive Stroke Center^34^. The data were collected over ten years utilizing eleven magnetic resonance scanners operating at either 1.5T or 3T, sourced from four different vendors and comprising diverse MRI acquisition protocols and machine technologies, leading to strong variations in image quality such as resolution, signal-to-noise ratio, and contrast-to-noise ratio. This makes this dataset uniquely suited to evaluate the generalizability of algorithms. Ischemic lesion masks were annotated by the dataset authors through manual delineation. Patient age was 62.5  ± 13.3 years. 907 patients (53.8%) were male. Reported race or ethnicity included 753 (44.7%) Black or African American, 490 (29.1%) White, and 40 (2.4%) Asian. 876 (52.0%) MRI scans were acquired after thrombolysis treatment with intravenous tissue-type plasminogen activator.

**Fig. 5:**
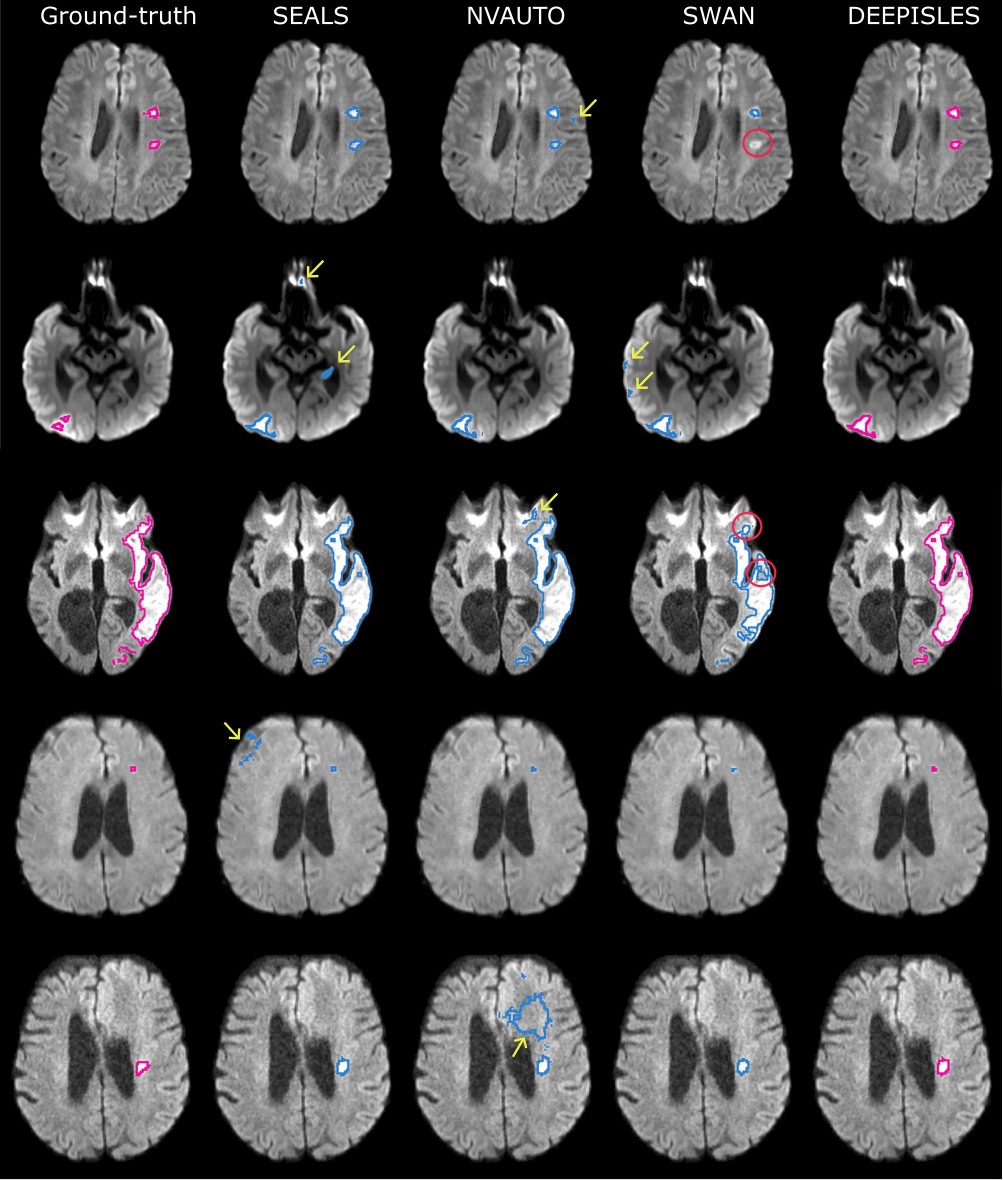
Segmentation outcomes on the external Johns Hopkins dataset. *DeepISLES* improves upon suboptimal segmentations generated by individual algorithmic approaches. Yellow arrows indicate false positives, while red circles highlight false negatives.

Table 2 summarizes the algorithmic results in this external dataset. The performance of the individual algorithms well reflects the patterns observed in the ISLES’22 test set (Table S2.1, supplementary material section 2): while the team SEALS leads the lesion-wise detection in terms of F1 score, the team NVAUTO leads the segmentation performance in terms of Dice scores. *DeepISLES*, however, outperforms all individual algorithms, exhibiting statistical significance across all metrics and comparisons, showing enhanced robustness and combining the strengths while mitigating the weaknesses observed in the individual algorithms: *DeepISLES* retains the strong lesion segmentation from the NVAUTO algorithm and the superior lesion detection of the SEALS algorithm. Consequently, it achieves 7% and 1% higher 5th and 50th Dice percentiles, respectively, than the SEALS algorithm, while outperforming both NVAUTO and SEALS in lesion-wise detection with a 6% and 2% higher 50th percentile F1 score, respectively. Of note, this improved performance is further underlined by a reduced amount of false positive detections (an important issue with DWI, where imaging artifacts are common): the false positive volumetric error is lesser for *DeepISLES* (2.7 ± 5.3 ml) compared to the individual algorithmic solutions (2.9 ± 5.9 ml for NVAUTO, 2.8 ± 5.8 ml for SEALS, and 2.9  ± 6.1 ml for SWAN). Figure 5 illustrates example cases where *DeepISLES* rectifies suboptimal segmentations produced by underperforming individual algorithms, effectively reducing false positive (or false negative) lesions and delivering more accurate results. Moreover, there is a very high agreement between the lesion volumes estimated through *DeepISLES* and those manually obtained by experts (Pearson’s *r* = 0.97). It is noteworthy that the performance achieved on the external Johns Hopkins dataset closely mirrors the results obtained on the ISLES’22 test set, with Dice and F1 scores aligning closely with those reported in the challenge dataset: there are no statistically significant differences in performance between the Johns Hopkins dataset results and ISLES’22 (Dice coefficient *p*-value = 0.46, F1 score *p*-value = 0.66, Wilcoxon rank-sum tests). Despite its robust overall performance, *DeepISLES* still exhibits limitations in specific scenarios. Examples of suboptimal segmentations are shown in the supplementary material section 7, with common failure modes including small infarcts in areas prone to DWI artifacts (e.g., cerebellum and cortical sulci) or in patients with chronic lesions, such as old (post-ischemic) lesions, which introduce complex imaging patterns that may challenge model generalization.

**Table 2:** Algorithm performance in the Johns Hopkins Comprehensive Stroke Center dataset

Algorithm	DSC *↑*	*p*-value	F1 *↑*	*p*-value	AVD (ml) *↓*	*p*-value	ALD *↓*	*p*-value
DeepISLES	**0.82** ± 0.15	–	**0.86** ± 0.33	–	**0.84** ± 3.96	–	**1.00** ± 2.00	–
	[0.45, 0.94]		[0.4, 1.00]		[0.03, 18.36]		[0.00, 9.00]	
SEALS	0.81 ± 0.16	2.2 × 10^−16^	0.84 ± 0.33	4.5 × 10^−7^	0.91 ± 3.95	0.0008	**1.00** ± 2.00	0.0026
	[0.38, 0.94]		[0.4, 1.00]		[0.03, 18.62]		[0.00, 9.00]	
NVAUTO	**0.82** ± 0.15	1.4 × 10^−6^	0.80 ± 0.33	2.2 × 10^−16^	**0.84** ± 3.87	0.0072	**1.00** ± 3.00	2.2 × 10^−16^
	[0.47, 0.94]		[0.4, 1.00]		[0.03, 18.50]		[0.00, 10.00]	
SWAN	0.79 ± 0.20	2.2 × 10^−16^	0.80 ± 0.33	2.2 × 10^−16^	1.01 ± 4.11	3.0 × 10^−9^	**1.00** ± 3.00	2.5 × 10^−7^
	[0.10, 0.92]		[0.29, 1.00]		[0.03, 20.65]		[0.00, 11.00]	

*DeepISLES* significantly outperforms all individual methods and effectively combines their strengths. Values are median  ± interquartile range and [5th, 95th percentile]. Best median values in bold. Wilcoxon signed-rank tests used for comparisons. Source data are provided as a Source Data file.

*DSC* dice similarity coefficient, *F1* lesion-wise F1 score, *AVD* absolute volume difference, *ALD* absolute lesion count difference.

**Fig. 6:**
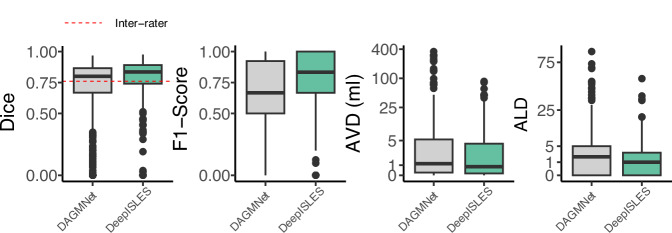
Algorithmic comparison on a subset of the Johns Hopkins dataset (sample size *N* = 417). DeepISLES demonstrates exceptional generalizability outperforming DAGMNet, despite DAGMNet being specifically trained on the Johns Hopkins dataset. The inter-rater Dice line indicates the Dice coefficient obtained between manual delineations by two experts on a subset of scans (*N* = 220), as reported by Liu et al.^34^ AVD Absolute Volume Difference, ALD Absolute Lesion Count Difference. Boxes show the interquartile range (IQR; 25th–75th percentiles), the center line marks the median, whiskers span values within 1.5 × IQR, and points beyond are displayed as outliers. Source data are provided as a Source Data file.

To further evaluate the performance of the proposed solution, we conducted a direct comparison between *DeepISLES* and DAGMNet, a state-of-the-art deep learning algorithm specifically devised and trained on the Johns Hopkins dataset^7^. The evaluation was conducted on a subset (*N* = 417) of scans from the same dataset, which were also part of DAGMNet test set^7^. Figure 6 presents the performance results of both methods. *DeepISLES* consistently outperformed DAGMNet across all evaluated metrics, achieving superior mean (median) Dice scores of 7.4% (3.6%) and lesion-wise F1 scores of 12.6% (16.7%). Furthermore, *DeepISLES* reduced the mean absolute volume difference by 6.7 ml and the mean absolute lesion count difference by 2.5 lesions compared to DAGMnet. In correlation terms with ground-truth lesion volumes, *DeepISLES* achieved a Pearson’s *r* of 0.98, compared to *r* = 0.74 for DAGMNet.

Lastly, we evaluated the association between the lesion volumes estimated by *DeepISLES* with the National Institutes of Health Stroke Scale (NIHSS) at patient admission (*N* = 999) and with the modified ranking scales (mRS) at 90-day follow-up (*N* = 782). We observe comparable correlations between lesion volumes and clinical scores when using *DeepISLES*-predicted masks (NIHSS: *r* = 0.55; 90-day mRS: *r* = 0.41; Pearson correlation coefficients) and manually delineated lesions (NIHSS: *r* = 0.54; 90-day mRS: *r* = 0.39). These findings show that the proposed algorithm can derive downstream clinical scores at a level comparable to those derived by radiologists.

### DeepISLES is readily available to use

We have made *DeepISLES* publicly accessible to support its adoption by physicians and researchers alike. The tool is available as a Docker image, a web service, a standalone software with a graphical user interface, and through its Git repository in source form. *DeepISLES* supports native MR series in both DICOM and NIfTI formats, directly exportable from clinical healthcare imaging centers. It enables end-to-end processing, including image skull-stripping and registration to MNI atlas. Detailed information about the tool, its features, and access instructions can be found at https://github.com/ezequieldlrosa/DeepIsles^26^.

## Discussion

Accurate segmentation of ischemic stroke lesions from brain MRI is crucial for timely diagnosis, treatment planning, and patient follow-up. Deep learning offers a promising avenue to support radiologists by enabling faster, more objective, and potentially more accurate MRI analysis. This study addresses this challenge by proposing a clinically meaningful and generalizable deep learning algorithm for ischemia segmentation. To foster development and rigorously assess candidate solutions, we organized the international ISLES’22 medical segmentation challenge. ISLES’22 served as a powerful platform for rapid algorithm benchmarking and identification of promising approaches, including the one presented here.

The following discussion focuses on two critical aspects. First, we examine how ISLES’22 served as a platform for identifying strong deep learning algorithms, culminating in the development of a single robust solution: *DeepISLES*. Second, we evaluate the real-world applicability of the algorithm, emphasizing its robustness, generalization to unseen data domains, and potential impact in clinical and research settings.

### DeepISLES: an outcome from the ISLES’22 challenge

The ISLES’22 challenge yielded fascinating insights into the landscape of stroke segmentation algorithms. Interestingly, the challenge leaderboard revealed that even algorithms based on similar CNN architectures and optimization strategies can exhibit variable performance. This reinforces the notion that factors beyond architecture, like hyper-parameter tuning, stochastic optimization, and training data sub-splitting (as in cross-validation), all contribute to model variability, even with a consistent dataset like ISLES’22^35^. However, the challenge also showcased the effectiveness of diverse algorithmic approaches. While achieving similar performance on most metrics, the top three ranked solutions employed different methodologies. The leading two teams utilized distinct CNN architectures (nnU-Net^27^ and SegResNet^36^) and loss functions (Dice with binary cross-entropy vs. Dice with focal loss). Notably, the third-ranked solution adopted a completely different approach based on non-negative matrix factorization operations^37^. This solution also leveraged the FLAIR modality (discarded by the top two teams), necessitating additional FLAIR-to-DWI co-registration. Some submissions also demonstrated innovative transfer learning strategies -for example, the PAT team fine-tuned models pre-trained on brain tumor segmentation tasks for ischemic stroke and subsequently validated them on private external datasets, as detailed in their post-challenge work^38^.

This remarkable diversity in algorithmic solutions also highlights the power of the ISLES’22 challenge in fostering innovation and creativity among participants: for a single research team, coming up with such a variety of methods is hardly possible. However, this variety is the basis for the strong ensemble built into *DeepISLES*. Our findings, therefore, highlight the unique potential of biomedical challenges to create (ensemble) solutions whose clinical utility extends beyond the challenge setting. To enable this, ISLES’22 offered significant advancements over prior iterations by incorporating a large, multi-centric dataset with over 6 times more scans than in a similar previous edition (ISLES’15^21^). This data reflects the real-world heterogeneity of stroke lesions, promoting generalizability. Notably, minimal data preprocessing was applied, focusing solely on patient de-identification. This challenged participants to develop end-to-end solutions encompassing all necessary processing steps (e.g., modality selection, registration, normalization), mimicking real-world clinical workflows. This, in turn, discouraged the convergence towards a single, potentially overfitted solution, as can occur with highly curated datasets. Furthermore, the challenge fostered robust evaluation by employing hidden data for testing. Participant models were presented with unseen MRI scans, preventing both model overfitting and intentional calibration towards specific images. Furthermore, the proper selection of evaluation metrics seems crucial. ISLES’22 addressed this by incorporating expert recommendation guidelines^31,32^ and by balancing technical metrics commonly found in the computer vision community (e.g., Dice scores) with clinically relevant and task-specific ones (e.g., number and volume of predicted ischemic lesions). This comprehensive approach allowed for a broader assessment of solutions’ performance and their readiness for real-world clinical applications, thus helping to bring artificial intelligence methods closer to clinical settings.

A key output of this work is *DeepISLES*. It was devised in a post-challenge scenario in collaboration with the top-ranked teams identified in the challenge. DeepISLES integrates the strengths of the individual solutions through consensus voting, thus providing a comprehensive solution to ischemic stroke segmentation robust in challenging scenarios. Besides, with the aim of turning *DeepISLES* into a really usable software tool for the clinical and research communities, it is fully standalone so that it can handle real-world scans directly after image acquisition and without requiring prior data processing.

### Beyond ISLES’22: towards automatic ischemic stroke segmentation in the clinical setting

In order to ensure the development of truly reliable AI solutions, a deeper understanding of the algorithms’ strengths and limitations is paramount. We addressed this need by extending our analysis beyond the challenge benchmarking and beyond the initial challenge dataset. A detailed evaluation of *DeepISLES* across various axes of generalization was conducted, including imaging center, ischemic lesion size, stroke phase, type of stroke pattern/configuration, and anatomical location of the ischemia.

*DeepISLES* demonstrates robust performance in handling a wide range of image and disease variations. This is evident from the successful generalization to unseen (ISLES’22) data from a new center, which achieved results similar to those of the trained center. Interestingly, while centers *#*1 and *#*3 (seen and unseen, respectively) showed similar metric distributions, a significant difference in Dice scores arose between centers *#*1 and *#*2 (both seen during training). This discrepancy can be attributed to two key factors. First, scans from center *#*2 had considerably smaller stroke lesions. Second, these scans were acquired during the acute stroke phase. This observation highlights the fact that the timing of brain imaging relative to stroke onset could significantly impact model performance. Despite the observed difference in Dice scores between the acute and sub-acute stroke groups, the observed volumetric lesion agreement was exceptionally high for both groups (Pearson’s *r* = 0.99 and 0.98 for acute and sub-acute, respectively). Ischemia detectability, as measured by lesion-wise F1 scores, also remained statistically similar between groups. *DeepISLES* also demonstrates robustness across varying lesion sizes, providing reliable volume estimates in strong agreement with ground-truth data. It also maintains high detectability for both small emboli and large infarcts, ensuring consistent performance across heterogenous brain ischemia. These results derived from the ISLES’22 challenge lead to some key conclusions: *i*) The model demonstrates remarkable generalizability across different stroke phases (acute and sub-acute), lesion sizes, and imaging centers. Thus suggesting the successful capture of stroke lesion variability, avoiding reliance on center-specific MRI features. *ii*) The stroke phase at scan acquisition influences performance. This is understandable as early (acute) scans exhibit different MR characteristics compared to later ones (sub-acute) due to evolving tissue changes. This aligns with established knowledge about how DWI and ADC values fluctuate with stroke progression^12^. Similarly, DWI sensitivity (specificity) ranges between 73% (92%) 3 hours from the stroke event to 92% (97%) 12 h from the stroke event^4,11^. Furthermore, false negatives may also increase with early DWI acquisition^4,39^.

*DeepISLES* also sheds light on stroke etiology. Traditionally, stroke type and affected vascular territories are crucial for determining the underlying cause, impacting treatment decisions and prevention strategies (e.g., Merino et al.^4^, Kim et al.^40^). Existing research establishes associations between DWI lesion patterns and stroke causes (e.g. Kang et al.^13^). For instance, scattered infarcts across multiple territories, single cortico-subcortical lesions, or multiple lesions in the anterior and posterior circulation often indicate cardioembolism^4,13,40,41^. Conversely, large artery atherosclerosis typically presents with lesions in a single vascular territory^4,13^. In this context, our deep learning algorithm stands out by accurately characterizing DWI images from a multi-faceted perspective. The model effectively segments ischemia in different stroke sub-groups (SVI, scattered infarcts, and mixed SVI/scattered) with consistent performance. Importantly, the model tackles even small ischemic volumes (e.g., embolic showers) with high detectability (lesion-wise F1 scores comparable to larger lesions). Furthermore, it accurately identifies stroke subgroups and affected vascular territories using predicted lesion masks (multi-class balanced accuracies of  ~87% and  ~98%, respectively, Table 1). These findings suggest that the algorithm’s outputs extend beyond lesion volume, providing valuable clinical insights into stroke type and underlying cause, ultimately aiding downstream clinical assessments.

The strength of *DeepISLES* lies in its competitiveness, robustness, and clinical utility. Validation in the Johns Hopkins stroke dataset—a large, unseen, and highly heterogeneous patient cohort - demonstrates that our approach overcomes the limitations of individual algorithms, resulting in more reliable stroke lesion detection and segmentation. *DeepISLES* effectively combines the segmentation accuracy of NVAUTO—reflected in high Dice scores and low absolute volume differences—with the lesion-wise detection capabilities reflected in SEALS F1 scores and lesion counts difference. Moreover, the segmentation results closely mirror those from the ISLES’22 challenge hidden test set, further validating *DeepISLES* and the effectiveness of the challenge design. Importantly, when comparing our solution against DAGMNet, an algorithm specifically designed and trained on the unseen Johns Hopkins dataset, our solution outperforms DAGMNet by a significant margin (7.4% higher mean Dice and 12.6% higher F1 scores), underscoring its generalizability and exceptional performance on new scans. When assessing lesion volumes predicted by the proposed algorithm over the Johns Hopkins dataset, a high agreement with the expert’s delineations was obtained. The algorithm-derived lesion volumes explain clinical stroke scores (admission NIHSS and 90-day mRS) at least as effectively, and potentially even better, than those obtained through manual expert delineation, thus showcasing the downstream clinical utility of such a tool. Qualitative analysis performed in a Turing-like test showcased that experienced neuroradiologists preferred *DeepISLES* outputs over manual lesion tracing in terms of segmentation *completeness* and *correctness*, thus suggesting that the proposed algorithm can match or even surpass human experts in identifying brain infarcts on MRI scans.

*DeepISLES* has the potential to significantly transform clinical stroke research and practice. It distinguishes itself from existing tools^7,9,38,42–46^ through its broad implementation across multiple formats, creating unprecedented accessibility for diverse user groups—from research scientists to clinicians. This flexibility enables various deployment pathways while fully embracing FAIR principles. Moreover, *DeepISLES* establishes itself as the leading stroke segmentation solution through rigorous validation on the largest public stroke dataset available to date. It consistently surpasses state-of-the-art methods in both ischemia identification and segmentation, achieving statistically significant and clinically meaningful performance advantages with substantial margins across all evaluation metrics. Notably, *DeepISLES* exhibits robust generalization capabilities when applied to out-of-domain data, further validating its reliability and highlighting its practical utility in real-world clinical environments.

Our study presents two significant findings. First, we introduce *DeepISLES*, an innovative algorithm designed to detect and segment ischemic stroke lesions across a variety of scenarios, achieving performance levels comparable to those of expert (neuro)radiologists. Second, we illustrate the potential of biomedical challenges to foster the development of models that extend beyond the confines of the challenge itself, underscoring their real-world clinical applicability and bridging the gap between biomedical research and clinical practice. *DeepISLES* is accessible in multiple formats at https://github.com/ezequieldlrosa/DeepIsles^26^.

### Limitations and outlook

Despite encouraging results in the Johns Hopkins dataset, further validation on additional external datasets is required to comprehensively assess the generalizability of our model. We systematically introduced variability to the data to assess the algorithm’s performance across different patient populations and stroke scenarios. While we focused on major sources of variation, limitations remain. First, the external testing dataset lacked multi-center representation. Second, the ISLES’22 dataset consisted solely of European cohorts, while the external dataset, although offering more patient race variability, was limited to the US population. In addition, users should be aware that sub-optimal performance may occur in cases with small infarcts located in artifact-prone regions (such as posterior circulation territories, and cortical sulci) or in the presence of chronic brain lesions, which can confound model predictions. Addressing these limitations, we believe the ensemble algorithm would significantly benefit from validation on external cohorts collected from diverse medical centers worldwide. Moreover, the inclusion of under-represented patient groups is crucial. We encourage clinical research groups to participate in validating and refining our model to enhance its generalizability and clinical impact.

## Methods

In order to devise a robust algorithm that can identify brain ischemia under heterogeneous *real-world* imaging scenarios, we organized the ISLES’22 challenge to collect diverse solutions from leading research teams. The challenge enables a fast and extended benchmarking of algorithmic strategies for tackling the task. This section is organized in two parts. The first section explains how the challenge is structured. The BIAS (Biomedical Image Analysis ChallengeS)^23^ guideline is followed. The second section details how the identified algorithms are evaluated and integrated into *DeepISLES*, a robust and clinically useful solution.

### The ISLES’22 challenge

The ISLES challenge (https://www.isles-challenge.org/) is a collaborative, multi-institutional, non-profit initiative uniting leading neurointerventionalists, radiologists, and researchers in clinical and medical imaging with the aim of enhancing the accuracy, fairness, and reproducibility of ischemic stroke algorithms. In the ISLES’22 edition, participants were tasked with developing fully automated algorithmic solutions for segmenting ischemic lesions across hyperacute, acute, and early subacute stroke stages using MRI modalities, including DWI, ADC, and FLAIR. The algorithms produce a binary stroke segmentation mask as their output.

#### Challenge organization

The aims, structure, and organization of the challenge are available for the teams several weeks before the dataset is released. A detailed description of the challenge organization is available in de la Rosa et al.^25^. The challenge is organized in three phases: a *train*, a *sanity-check*, and a *test* phase. In the train phase, participating teams have six weeks to develop a solution to the task using a labeled MRI dataset. All teams have access to the data at the same time. There are no technical constraints on the employed algorithmic solution. In the sanity-check phase, participants can test their devised Docker solutions over a few train set scans in order to ensure that their algorithms properly work in the challenge organizers’ servers. In the test phase, participating teams are requested to submit a Docker containing their final algorithmic solution. Teams can submit just a single time to this phase. The algorithm is later run by the challenge organizers over the hidden test set. Algorithmic performance is measured by computing relevant metrics (below detailed) using the predicted segmentations and the ground truth masks. Later, teams are ranked based on their yield performance metrics.

#### Dataset

The dataset used in this study is customarily devised for the purposes of the challenge. It contains multi-center and multi-scanner data, and it consists of MRI scans (*n* = 400) acquired during the early/late acute and the early sub-acute stroke phase of patients across three European health centers. The train (*N* = 250) and test (*n* = 150) sets include scans from two and three healthcare centers, respectively. Most of the datasets have been acquired in the subacute post-stroke stage, mainly three days after treatment (#post-RT). Furthermore, as a proof of concept, we aim to test the generalization capability of the devised algorithms over a small, single-center subset of hyper-acute/early acute stroke scans (12.5%, *N* = 50) before intervention (#pre-RT), which represents a third part of the test set. Table 3 provides a summary of the ISLES’22 dataset. All cases include DWI (*b*-value = 1000 s/mm^2^), FLAIR, and apparent diffusion coefficient (ADC) MRI. The ischemic stroke segmentation ground truths are obtained using an algorithm-human hybrid iterative method and are later revised and refined by one out of three experienced neuroradiologists with more than 10 years of experience (RW, JSK, and BW, who reviewed 150, 125, and 125 scans, respectively). The MRI images are released in their native acquisition space (ground truth masks are released in the DWI/ADC space) after minimal pre-processing. Thus, pre-processing is solely performed with the purpose of patient de-identification and therefore consists of MRI skull-stripping. The reason for releasing the dataset ‘as raw as possible’ is to encourage the development of algorithms that could deal with real-world raw imaging data, which suffers from a large variability (signal-to-noise, resolutions, variable parameter settings, etc.) and therefore has its own technical limitations (e.g., different MR modalities, as FLAIR and DWI, are not co-registered). In this sense, participants are also challenged to devise end-to-end algorithms that can deal with the pre-processing and curation of the images. For more detailed information about this dataset, including the ground-truth annotation process, please refer to the corresponding data descriptor^30^.

**Table 3:** Data summary

Dataset	Phase	# Scans	# Scans by center (1 / 2 / 3)	RT	# Scans pre-RT	# Scans post-RT	Stroke phase
ISLES’22^30^	Train	250	200 / 50 / 0	MT	0	250	Sub-acute
	Test	150	50 / 50 / 50	MT	50	100	Acute and sub-acute
JHCSC^34^	Test	1685	–	ivtPA	810	876	Acute and sub-acute

The ISLES’22 dataset was used in the challenge competition, while the JHCSC was used as an external testing dataset in a post-challenge setting. Further details about the datasets are available in the corresponding data descriptors^30,34^.

*JHCSC* Johns Hopkins Comprehensive Stroke Center, *RT* reperfusion treatment, *MT* mechanical thrombectomy, *ivtPA* intravenous tissue plasminogen activator.

#### Algorithms’ evaluation: metrics and ranking

The algorithmic results are evaluated by comparing the predicted lesions masks with the (manually traced) ground truth masks. Metrics are chosen following current recommendation guidelines^31,32^. Metrics that are well known in the literature for both the (medical imaging) research and radiological communities were included: Dice score^47^ (DSC), the absolute volume difference (AVD = ∣Volume_predicted_ − Volume_ground truth_∣), lesion-wise F1 score (defined as in Section *Statistical analysis* by considering instance lesions), and the absolute lesion count difference (ALD = ∣*#* Lesions_predicted_ − *#* Lesions_ground truth_∣). Lesion-wise metrics were computed after isolating disconnected ischemias in the binary masks through connected-component analysis. Note that including complementary segmentation and detection metrics is beneficial and helps evaluate models in a robust, subject, and lesion-wise unbiased scheme^48^. Implementation details of the four metrics can be found in the challenge Python notebooks^49,50^.

The final competition ranking is obtained from the *test* phase, which is *blind* (there is no access for the teams to the MRI images to be predicted) and *single-shoot* (one submission per team is solely allowed), thus completely precluding participants from any sort of overfitting strategy. As done in previous ISLES editions^21,51,52^, the ranking is performed in a ‘rank then aggregate’ fashion^14^. A thousand bootstraps are conducted by repeatedly drawing samples with replacements and recalculating the rankings in each iteration. Furthermore, as a complementary analysis, we calculated the challenge ranking through a thousand bootstraps ‘aggregate then rank’ scheme.

### DeepISLES: a collaboratively developed solution

*DeepISLES* is a comprehensive, end-to-end software solution designed to handle real-world datasets immediately after image acquisition. It integrates advanced preprocessing capabilities, enabling it to process images in their native space, perform skull-stripping, and register scans to the MNI-152 atlas. The framework supports standard medical imaging formats, including DICOM and NIfTI (.nii, .nii.gz, .mha). On a system with an 8-core CPU, the Docker image takes on average 2 min on a GeForce RTX 3090 (24GB) and 5 minutes on a GeForce GTX 1080 Ti (12GB), while the web service execution time is approximately 10 minutes. When run directly via the source Git repository in Python on a GPU-enabled machine, the model generates segmentation outputs in approximately 1.5 minutes.

The development of *DeepISLES* leveraged insights from the top three methods on the ISLES’22 challenge leaderboard. *DeepISLES* integrates the solutions from the teams SEALS, NVAUTO, and SWAN using an ensembling, majority voting strategy. For each voxel in an MRI scan, the predicted output (lesion or no lesion) is determined by the consensus of at least two of the three methods. This ensemble approach ensures resilience to challenging cases, allowing accurate lesion detection even when an individual algorithm fails. The individual methodologies employed by each team are described below.

*Algorithm SEALS*. The participants utilized DWI and ADC images as input for their algorithm. Image pre-processing involved the resampling of the scans to a 1 × 1 × 1 mm^3^ voxel resolution, followed by a z-score image normalization. The nnU-Net pipeline^27^ was employed for training a 3D full-resolution U-Net. A 1/7 subset of the training dataset was held out to evaluate the performance of the model. The remaining 6/7 parts of the dataset were used to train models through 5-fold cross-validation. A combined Dice loss with categorical cross-entropy was used. Data augmentation transforms were used, including image flipping and Gaussian noise addition. The final submission to the challenge was an ensemble of the five trained models.

*Algorithm NVAUTO*. The team proposed an automated 3D semantic segmentation solution implemented with Auto3DSeg^53^. The algorithm automated most deep learning steps and decision choices. DWI and ADC images were used as input to the model after voxel resampling to 1 × 1 × 1 mm^3^ and z-score normalization. SegResNet^36^ models were trained through 5-fold cross-validation. Several augmentation transforms were used, including flipping, rotation, scaling, smoothing, intensity-scaling and -shifting. Random cropped patches of dimensions 192 × 192 × 128 were adopted. The models were trained on an 8-GPU NVIDIA V100 machine with an AdamW optimizer and unitary batch size. Dice loss with focal loss using deep supervision was used as a loss function. The model was first pre-trained on the BRATS 2021 dataset^54^. The final algorithm was an ensemble of 15 models obtained through a 3-time 5-fold cross-validation.

*Algorithm SWAN*. The participants used the *Factorizer*^37^ algorithm to construct an end-to-end, linearly scalable model for stroke lesion segmentation. Factorizer is a family of models that leverage the power of Non-negative Matrix Factorization (NMF) to extract discriminative and meaningful feature maps. The algorithm uses a differentiable NMF layer that can be back-propagated during the training of deep learning models. A *Factorizer* block is constructed by replacing the self-attention layer of a vision transformer block^55^ with an NMF-based module and then integrating them into a U-shaped architecture with skip connections. The participants used a Swin Factorizer, which combines NMF with the shifted-window idea inspired by Liu et al.^56^ to effectively exploit local context. Preprocessing involves FLAIR-to-DWI image registration using Elastix^57^ and z-score normalization. Various data augmentation techniques were performed, including random affine transforms, flips, and random intensity scalings. Deep supervision was used at the three highest decoder resolution levels for training the models. The final challenge submission was an ensemble of Swin Factorizers and UNet models with residual blocks^58^ (a.k.a ResU-Net) obtained through 5-fold cross-validation.

### Towards real-world clinical performance

#### Stress-testing the model: Which (and how) real-world variables impact it?

With the aim of understanding the potential clinical utility of the deep learning solution, we evaluate whether *DeepISLES* can detect ischemia under diverse disease and imaging scenarios, thus providing insights about its robustness and generalization capability when dealing with diverse ischemic stroke events. With this aim, the test-phase predictions of the ensemble algorithm are evaluated over *i*) scans coming from an external healthcare center, unseen during model development, *ii*) scans with diverse ischemic lesion size, *iii*) scans with ischemia located in diverse vascular territories of the brain, *iv*) scans with diverse lesion configurations and patterns, and *v*) scans with heterogenous image contrast due to different stroke phases.

##### Multi-center data

We test *DeepISLES* performance over scans acquired in an external imaging center unseen during the development (train phase) of the models. To this end, 50 test-phase scans from center #3 (University Medical Center Hamburg-Eppendorf), a center not included in the train phase, are evaluated and compared to 100 unseen test-phase scans from centers #1 (University Hospital of the Technical University Munich) and #2 (University Hospital of Bern). While all test-phase scans are unseen, centers #1 and #2 were part of the training phase, making their data distribution familiar to the model.

##### MRI acquisition time

*DeepISLES* is evaluated over two sub-groups of the test set data clustered based on the stroke phase. The first group considers scans (*N* = 100) acquired during the late acute or early sub-acute course of the disease. In these cases, MRI is acquired after treating the patient with mechanical thrombectomy. The second group considers patients (*N* = 50) imaged during the early acute phase of the disease and, therefore, MRI is acquired before treating the patient with mechanical thrombectomy.

##### Lesion size

Ischemic stroke spans from minor brain lesions of a few milliliters to large-vessel occlusions involving over a hundred milliliters of brain tissue. Therefore, to understand the algorithm performance when dealing with different ischemic lesion sizes, the test-phase data is split into three stroke sub-groups: lesions smaller than 5 ml, lesions bigger or equal to 5 ml but smaller than 20 ml, and lesions greater than or equal to 20 ml.

##### Vascular brain territory

In this experiment, we evaluate if *DeepISLES* can identify the affected brain vascular territory in the MRI scans. For doing so, the test-phase scans are linearly registered to a FLAIR MNI template with vascular territory annotations^59^ using NiftyReg^60^. Later, the lesion load over each vascular territory is quantified using the ground truth annotations and each scan receives a label of the vascular territory that yields the largest lesion load. The considered vascular territories are the ones covered by the middle cerebral artery, the anterior cerebral artery, the posterior cerebral artery, the arteries perfusing the cerebellum, and the ones perfusing the pons and medulla. The deep learning predictions of the vascular territories are generated by finding the vascular territory with the largest (predicted) lesion volume. Then, we assess through classification metrics how well the algorithms identify the stroke vascular territory.

##### Stroke pattern

The test-phase scans are assigned to one of the  four following clinical sub-groups depending on the type of lesion and stroke pattern:

- No ischemia
  Scans with no ischemic lesions (lesion volume of 0 ml, *N* = 2).
- Single vessel infarct
  Scans with the largest lesion accounting for >95% of the total lesion volume (*N* = 62).
- Scattered infarcts based on micro-occlusions
  Scans with ≥ three single lesions where either the largest lesion represents < 60% of the total lesion volume or the total lesion volume is < 5 ml (*N* = 48).
- Single vessel infarct with accompanying scattered infarcts
  All remaining scans (*N*=  38).

To label the scans, we perform an iterative computer-human approach. First, using prior knowledge from experienced neuroradiologists (BW and JSK) we define heuristic classification rules that assign each scan to one of the subgroups. Later, the same neuroradiologists evaluated the labels assigned to the scans and updated the heuristic rule, improving its labeling performance. After some iterations, the heuristic rule that suffix the stroke pattern grouping are the ones mentioned above. In order to evaluate if the algorithms can predict the stroke lesion subgroup, these heuristic rules are applied to each (predicted) stroke mask. Then, the stroke subgroup predictions are compared against the “real” labels obtained through the ground truth stroke masks. Conventional classification metrics are used to evaluate the algorithm’s performance.

#### DeepISLES versus experts in a Turing-like test

Nine radiologists from three healthcare centers (University Hospital of the Technical University Munich, University Medical Center Hamburg-Eppendorf, and University Hospital of Bern) blindly rated the quality of the ischemic stroke masks generated either by experts or by the devised algorithm. Forty or forty-one scans with three annotated slices each (two axial, one sagittal) were provided to each radiologist. All images were randomized, and no information about the annotator (human or algorithm) was provided. Radiologists were asked to rate the *completeness* of the segmented lesion and the correctness of their contours on a 1–6 scale as similarly done in Kofler et al.^33^ (see supplementary material section 6 for the criteria used).

#### Validating DeepISLES in external data

The algorithm is tested over a public, external, ischemic stroke cohort (*N* = 1685)^34^ including raw MRI (such as DWI, ADC, FLAIR, etc.), patient (e.g., age, sex, race) and clinical data (e.g., treatment, NIHSS and mRS scores, etc.). Table 3 summarises the dataset characteristics. Images were acquired over ten years using eleven 1.5T or 3T scanners from the four major machine vendors (Siemens, GE, Toshiba, and Philips). NIHSS and mRS scores were respectively performed at patient admission and at 90-day follow-up. Moreover, the time from symptom onset to MRI acquisition was recorded when the patient or the caregiver was confident about symptom onset. MRI was mostly performed six or more hours from symptom onset, before or after administration of intravenous tissue plasminogen activator. To predict ischemic lesions with the ensemble algorithm, all scans were priorly skull-stripped using HD-BET^61^.

#### Statistical analysis

Data are compared using two-sided non-parametric, Wilcoxon unpaired rank-sum, or paired signed-rank tests after observing that data is heteroscedastic and does not follow a Gaussian distribution. The significance level is set at *α* = 0.05. Bland-Altman^62^ analysis is used to evaluate the volumetric bias between the manually-traced and the algorithm-predicted lesion volumes. Classification metrics used to evaluate the algorithms are per-class F1 scores (${{{\rm{F}}}}1\,{{{{\rm{score}}}}}_{{{{\rm{c}}}}}=\frac{2*{{{{\rm{TP}}}}}_{{{{\rm{c}}}}}}{2*{{{{\rm{TP}}}}}_{{{{\rm{c}}}}}+{{{{\rm{FP}}}}}_{{{{\rm{c}}}}}+{{{{\rm{FN}}}}}_{{{{\rm{c}}}}}}$) and the balanced accuracy computed as the macro-average of the recall scores per class (${{{\rm{Balanced}}}}\,{{{\rm{Accuracy}}}}=\frac{1}{C}\,\mathop{\sum }_{c=1}^{C}{{\mbox{Recall}}}_{{{{\rm{c}}}}}$, with ${{{{\rm{Recall}}}}}_{{{{\rm{c}}}}}=\frac{{{{{\rm{TP}}}}}_{{{{\rm{c}}}}}}{{{{{\rm{TP}}}}}_{{{{\rm{c}}}}}+{{{{\rm{FN}}}}}_{{{{\rm{c}}}}}}$, TP are true positives, FP the false positives, FN the false negatives and C the number of classes). The scikit-learn Python library^63^ is used to compute the classification metrics.

### Reporting summary

Further information on research design is available in the Nature Portfolio Reporting Summary linked to this article.

## Supplementary information

Supplementary Information

Reporting Summary

## Source data

Source Data

## Data Availability

*Images* The ISLES’22 dataset used for the challenge is open and freely available under the Creative Commons CC BY 4.0 license. The train dataset is available in www.zenodo.org/^64^. The external stroke dataset used for validating the ensemble algorithm is available through ICPSR^34,65^. Source data are provided with this paper. *Challenge results* Performance metrics are available in Table S2.1 and Figure S2.1 (supplementary material section 2) and also through https://grand-challenge.org/^24^. Note that this challenge continues accepting submissions and, therefore, the online leaderboard is constantly getting updated. In this study, only the solutions received for the MICCAI’2022 challenge are evaluated. Source data are provided with this paper.

## Code availability

The devised algorithm *DeepISLES* is freely available in four versions: stand-alone software tool with integrated GUI, web-service, Docker image, and through the main GitHub repository. For details, check https://github.com/ezequieldlrosa/DeepIsles^26^. To help participants get familiar with the data and with the challenge performance metrics a Python notebook^49^ was made available in advance. Moreover, in order to facilitate the teams during the algorithms’ submission process, a Docker template and a Docker creation tutorial were shared with the teams^66^. Challenge rankings were obtained using challengeR v1.0.5^67^ Figures of this work were generated in R (v.4.4.3)^68^ using the ggplot2^69^ and the patchwork^70^ software packages. Figure 3 was generated with 3D Slicer (v4.8.0) (https://www.slicer.org/)^71^.
