# Supplementary material for: DeepISLES: a clinically validated ischemic stroke segmentation model from the ISLES'22 challenge
Source: Nat Commun. 2025 Aug 9;16:7357. doi: 10.1038/s41467-025-62373-x (PMC12335569; doi:10.1038/s41467-025-62373-x)
Supplement: Supplementary file 1 — Supplementary Information [file 41467_2025_62373_MOESM1_ESM.pdf]

Supplementary Material for

DeepISLES: A Clinically Validated Ischemic Stroke Segmentation Model from the ISLES'22 Challenge

Index of Figures and Tables.

Supplementary Material 1: ISLES’22 Summary of participating algorithms. p1
Supplementary Material 2: Extended DeepISLES performance analysis. p9
Table S2.1: Final ISLES'22 test-set ranking. p10
Figure S2.1: Boxplots of ISLES’22 test-set performance. p11
Figure S2.2: DeepISLES Dice vs lesion volumes on the ISLES’22 test set. p12
Figure S2.3: Volumetric lesion agreement (DeepISLES vs. ground truth). p13
Supplementary Material 3: ISLES’22 challenge report (‘*Rank then Aggregate*). p14
Table S3.1: AVD Ranking. p15
Table S3.2: Dice Ranking. p15
Table S3.3: F1 Score Ranking. p16
Table S3.4: LCD Ranking. p16
Table S3.5: Consensus Ranking Across Tasks. p17
Table S3.6: Kendall’s Tau Summary for Ranking Stability. p31
Figure S3.1: AVD Boxplots. p18
Figure S3.2: Dice BoxplotS. p19
Figure S3.3: F1 Boxplots. p19
Figure S3.4: LCD Boxplots. p20
Figure S3.5: AVD Podium Plot. p21
Figure S3.6: Dice Podium Plot. p21
Figure S3.7: F1 Podium Plot. p22
Figure S3.8: LCD Podium Plot. p22
Figure S3.9: AVD Ranking Heatmap. p23
Figure S3.10: Dice Ranking Heatmap. p24
Figure S3.11: F1 Ranking Heatmap. p25
Figure S3.12: LCD Ranking Heatmap. p26
Figure S3.13: AVD Ranking Stability – Blob Plot. p27
Figure S3.14: Dice Ranking Stability – Blob Plot. p28
Figure S3.15: F1 Ranking Stability – Blob Plot. p29
Figure S3.16: LCD Ranking Stability – Blob Plot. p30
Figure S3.17: Kendall’s Tau Violin Plot for All Metrics. p31
Figure S3.18: AVD Significance Map. p32
Figure S3.19: Dice Significance Map. p33
Figure S3.20: F1 Significance Map. p34
Figure S3.21: LCD Significance Map. p35
Figure S3.22: AVD Ranking Robustness Across Methods. p36
Figure S3.23: Dice Ranking Robustness Across Methods. p37
Figure S3.24: F1 Ranking Robustness Across Methods. p38
Figure S3.25: LCD Ranking Robustness Across Methods. p39
Figure S3.26: Cross-Task Ranking Stability – Blob Plot. p40
Figure S3.27: Bootstrap Ranking Stability – SEALS. p41
Figure S3.28: Bootstrap Ranking Stability – NVAUTO. p42
Figure S3.29: Bootstrap Ranking Stability – PAT. p43
Figure S3.30: Bootstrap Ranking Stability – SWAN. p44
Figure S3.31: Bootstrap Ranking Stability – CTRL. p45
Figure S3.32: Bootstrap Ranking Stability – VICOROB. p46
Figure S3.33: Bootstrap Ranking Stability – PLORAS. p47
Figure S3.34: Bootstrap Ranking Stability – MIRC. p48
Figure S3.35: Bootstrap Ranking Stability – CREATIS. p49
Figure S3.36: Bootstrap Ranking Stability – Dolphins. p50
Figure S3.37: Bootstrap Ranking Stability – AICONS. p51
Figure S3.38: Bootstrap Ranking Stability – A55972. p52
Figure S3.39: Stacked Rank Frequencies – SEALS. p53
Figure S3.40: Stacked Rank Frequencies – NVAUTO. p54

Figure S3.41: Stacked Rank Frequencies – PAT. p55
Figure S3.42: Stacked Rank Frequencies – SWAN. p56
Figure S3.43: Stacked Rank Frequencies – CTRL. p57
Figure S3.44: Stacked Rank Frequencies – VICOROB. p58
Figure S3.45: Stacked Rank Frequencies – PLORAS. p59
Figure S3.46: Stacked Rank Frequencies – MIRC. p60
Figure S3.47: Stacked Rank Frequencies – CREATIS. p61
Figure S3.48: Stacked Rank Frequencies – Dolphins. p62
Figure S3.49: Stacked Rank Frequencies – AICONS. p63
Figure S3.50: Stacked Rank Frequencies – A55972. p64
Figure S3.51: AVD Task-Level Ranking Stability – Blob Plot. p66
Figure S3.52: Dice Task-Level Ranking Stability – Blob Plot. p67
Figure S3.53: F1 Task-Level Ranking Stability – Blob Plot. p68
Figure S3.54: LCD Task-Level Ranking Stability – Blob Plot. p69
Figure S3.55: Cluster Dendrogram – Complete Linkage. p70
Figure S3.56: Cluster Dendrogram – Average Linkage. p70
Supplementary Material 4: ISLES'22 challenge report ('*Aggregate then Rank*'). p72
Table S4.1: AVD Ranking. p73
Table S4.2: Dice Ranking. p74
Table S4.3: F1 Score Ranking. p74
Table S4.4: LCD Ranking. p74
Table S4.5: Consensus Ranking. p75
Table S4.6: Kendall's Tau Summary for Ranking Stability. p89
Figure S4.1: AVD Boxplots. p76
Figure S4.2: Dice Boxplots. p77
Figure S4.3: F1 Boxplots. p77
Figure S4.4: LCD Boxplots. p78
Figure S4.5: AVD Podium Plot. p79
Figure S4.6: Dice Podium Plot. p79
Figure S4.7: F1 Podium Plot. p80
Figure S4.8: LCD Podium Plot. p80
Figure S4.9: AVD Ranking Heatmap. p81
Figure S4.10: Dice Ranking Heatmap. p82
Figure S4.11: F1 Ranking Heatmap. p83
Figure S4.12: LCD Ranking Heatmap. p84
Figure S4.13: AVD Ranking Stability – Blob Plot. p85
Figure S4.14: Dice Ranking Stability – Blob Plot. p86
Figure S4.15: F1 Ranking Stability – Blob Plot. p87
Figure S4.16: LCD Ranking Stability – Blob Plot. p88
Figure S4.17: Kendall's Tau Violin Plot for All Metrics. p89
Figure S4.18: AVD Significance Map. p90
Figure S4.19: Dice Significance Map. p91
Figure S4.20: F1 Significance Map. p92
Figure S4.21: LCD Significance Map. p93
Figure S4.22: AVD Ranking Robustness Across Methods. p94
Figure S4.23: Dice Ranking Robustness Across Methods. p95
Figure S4.24: F1 Ranking Robustness Across Methods. p96
Figure S4.25: LCD Ranking Robustness Across Methods. p97
Figure S4.26: Cross-Task Ranking Stability – Blob Plot. p98
Figure S4.27: Bootstrap Ranking Stability – SEALS. p99
Figure S4.28: Bootstrap Ranking Stability – NVAUTO. p100
Figure S4.29: Bootstrap Ranking Stability – SWAN. p101
Figure S4.30: Bootstrap Ranking Stability – PAT. p102
Figure S4.31: Bootstrap Ranking Stability – PLORAS. p103
Figure S4.32: Bootstrap Ranking Stability – VICOROB. p104
Figure S4.33: Bootstrap Ranking Stability – CTRL. p105
Figure S4.34: Bootstrap Ranking Stability – MIRC. p106
Figure S4.35: Bootstrap Ranking Stability – CREATIS. p107
Figure S4.36: Bootstrap Ranking Stability – Dolphins. p108
Figure S4.37: Bootstrap Ranking Stability – A55972. p109
Figure S4.38: Bootstrap Ranking Stability – AICONS. p110

Figure S4.39: Stacked Rank Frequencies – SEALS. p111
Figure S4.40: Stacked Rank Frequencies – NVAUTO. p112
Figure S4.41: Stacked Rank Frequencies – SWAN. p113
Figure S4.42: Stacked Rank Frequencies – PAT. p114
Figure S4.43: Stacked Rank Frequencies – PLORAS. p115
Figure S4.44: Stacked Rank Frequencies – VICOROB. p116
Figure S4.45: Stacked Rank Frequencies – CTRL. p117
Figure S4.46: Stacked Rank Frequencies – MIRC. p118
Figure S4.47: Stacked Rank Frequencies – CREATIS. p119
Figure S4.48: Stacked Rank Frequencies – Dolphins. p120
Figure S4.49: Stacked Rank Frequencies – A55972. p121
Figure S4.50: Stacked Rank Frequencies – AICONS. p122
Figure S4.51: AVD Task-Level Ranking Stability – Blob Plot. p124
Figure S4.52: Dice Task-Level Ranking Stability – Blob Plot. p125
Figure S4.53: F1 Task-Level Ranking Stability – Blob Plot. p126
Figure S4.54: LCD Task-Level Ranking Stability – Blob Plot. p127
Figure S4.55: Cluster Dendrogram – Complete Linkage. p128
Figure S4.56: Cluster Dendrogram – Average Linkage. p128
Supplementary Material 5: Emulated ISLES’22 challenge report with DeepISLES. p130
Table S5.1: AVD Ranking. p131
Table S5.2: Dice Ranking. p132
Table S5.3: F1 Score Ranking. p132
Table S5.4: LCD Ranking. p132
Table S5.5: Consensus Ranking Across Tasks. p148
Table S5.6: Kendall’s Tau Summary for Ranking Stability. p134
Figure S5.1: AVD Boxplots. p134
Figure S5.2: Dice Boxplots. p135
Figure S5.3: F1 Boxplots. p135
Figure S5.4: LCD Boxplots. p136
Figure S5.5: AVD Podium Plot. p137
Figure S5.6: Dice Podium Plot. p138
Figure S5.7: F1 Podium Plot. p138
Figure S5.8: LCD Podium Plot. p139
Figure S5.9: AVD Ranking Heatmap. p140
Figure S5.10: Dice Ranking Heatmap. p141
Figure S5.11: F1 Ranking Heatmap. p142
Figure S5.12: LCD Ranking Heatmap. p143
Figure S5.13: AVD Ranking Stability – Blob Plot. p144
Figure S5.14: Dice Ranking Stability – Blob Plot. p145
Figure S5.15: F1 Ranking Stability – Blob Plot. p146
Figure S5.16: LCD Ranking Stability – Blob Plot. p147
Figure S5.17: Kendall’s Tau Violin Plot for All Metrics. p148
Figure S5.18: AVD Significance Map. p149
Figure S5.19: Dice Significance Map. p150
Figure S5.20: F1 Significance Map. p151
Figure S5.21: LCD Significance Map. p152
Figure S5.22: AVD Ranking Robustness Across Methods. p153
Figure S5.23: Dice Ranking Robustness Across Methods. p154
Figure S5.24: F1 Ranking Robustness Across Methods. p155
Figure S5.25: LCD Ranking Robustness Across Methods. p156
Figure S5.26: Cross-Task Ranking Stability – Blob Plot. p157
Figure S5.27: Bootstrap Ranking Stability – DeepISLES. p158
Figure S5.28: Bootstrap Ranking Stability – SEALS. p159
Figure S5.29: Bootstrap Ranking Stability – NVAUTO. p160
Figure S5.30: Bootstrap Ranking Stability – PAT. p161
Figure S5.31: Bootstrap Ranking Stability – SWAN. p162
Figure S5.32: Bootstrap Ranking Stability – CTRL. p163
Figure S5.33: Bootstrap Ranking Stability – VICOROB. p164
Figure S5.34: Bootstrap Ranking Stability – PLORAS. p165
Figure S5.35: Bootstrap Ranking Stability – MIRC. p166
Figure S5.36: Bootstrap Ranking Stability – CREATIS. p167
Figure S5.37: Bootstrap Ranking Stability – Dolphins. p168

Figure S5.38: Bootstrap Ranking Stability – AICONS. p169
Figure S5.39: Bootstrap Ranking Stability – A55972. p170
Figure S5.40: Stacked Rank Frequencies – DeepISLES. p171
Figure S5.41: Stacked Rank Frequencies – SEALS. p172
Figure S5.42: Stacked Rank Frequencies – NVAUTO. p173
Figure S5.43: Stacked Rank Frequencies – PAT. p174
Figure S5.44: Stacked Rank Frequencies – SWAN. p175
Figure S5.45: Stacked Rank Frequencies – CTRL. p176
Figure S5.46: Stacked Rank Frequencies – VICOROB. p177
Figure S5.47: Stacked Rank Frequencies – PLORAS. p178
Figure S5.48: Stacked Rank Frequencies – MIRC. p179
Figure S5.49: Stacked Rank Frequencies – CREATIS. p180
Figure S5.50: Stacked Rank Frequencies – Dolphins. p181
Figure S5.51: Stacked Rank Frequencies – AICONS. p182
Figure S5.52: Stacked Rank Frequencies – A55972. p183
Figure S5.53: AVD Task-Level Ranking Stability – Blob Plot. p185
Figure S5.54: Dice Task-Level Ranking Stability – Blob Plot. p186
Figure S5.55: F1 Task-Level Ranking Stability – Blob Plot. p187
Figure S5.56: LCD Task-Level Ranking Stability – Blob Plot. p188
Figure S5.57: Cluster Dendrogram – Complete Linkage. p189
Figure S5.58: Cluster Dendrogram – Average Linkage. p189
Supplementary Material 6: Criteria for the Turink-like test scoring. p191
Table S6.1. Completeness criteria. p192
Table S6.2. Correctness criteria. p192
Supplementary Material 7: DeepISLES failures. p193
Figure 7.1. DeepISLES examples of suboptimal performance. p194

Supplementary material #1.

ISLES'22 participating algorithms:
Description of methods

This section includes short descriptions of the challenge participating algorithms. For the three top ranking solutions, we refer the reader to the main body of the manuscript. Methods are described in order based on their final challenge rank.

TEAM: PAT

Ensemble 3D and 2D nn-UNet to automatically segment ischemic stroke

Hyun-su Jeong, Chi-ho Yoon, Chul-hong Kim

Ischemic strokes narrowing or blocking arteries in brains are life-threatening diseases. Therefore, it is crucial to identify ischemic lesions early. Recently, deep learning models play essential roles to fully and automatically segment the lesions. In this work, we use an nn-UNet (Isensee et al., 2021) that considers both 2D and 3D information through an ensemble method. Initially, 3D and 2D nn-UNet models are separately trained. To avoid overfitting, we constructed five sub-folds for bagging at inference. Although the 3D model can capture 3D information, it requires heavy GPU computation allowing only the reduced path size as input. Therefore, it is difficult to extract the features of the entire area. The 2D model considers the whole areas, but the 3D information is still missing due to the nature of 2D data. To compensate for each shortcoming, we make use of an ensemble method. For training we employ the Dice + cross-entropy loss function. Our code is available here: https://github.com/poboai/ISLES22_PAT.

TEAM: CTRL-KCL

Multi-resolution Model Fusion for Ischemic Stroke Lesion Segmentation

Jiayu Huo, Sébastien Ourselin, Rachel Sparks

We proposed a multi-resolution model fusion method. Within the dataset there are two different image resolutions: high resolution, $2.0 \times 2.0 \times 2.0$ mm^3, and low resolution, $1.8 \times 1.8 \times 4.8$ mm^3. Therefore, we decided to train two segmentation models independently for each image resolution. The training dataset comprises 193 high-resolution cases which we use for training. For the low-resolution training dataset, we manually resample the high-resolution cases to low-resolution, and combine them with low-resolution cases which are in the training dataset. We split each training dataset into five folds according to the size of the stroke lesion area. Prior to training we first apply an affine transformation to the FLAIR image to align it with both DWI and ADC images. The three modalities are then concatenated and input to the model. We use nnU-Net (Isensee et al., 2021) for the model architecture and training. For each image set, we design two different training schemes. The first training scheme is using the TopK10 loss function to train the generic U-Net, because most lesions are small. By introducing the TopK10 loss function, the model will pay more attention to small foreground areas. In the second training scheme, we add a residual block (He et al., 2016) into the generic U-Net, and use the default compound loss function (dice + cross-entropy) to train the network. For each setting, we use the stochastic gradient descent (SGD) as the optimizer and set the initial learning rate as 0.01. The batch size is 2, and z-score intensity normalization is performed to normalize image patches. Due to the time limit, we train each setting for 500 epochs. The patch sizes used for high-resolution and low-resolution model training are 72×112×112 and 32×112×112,

respectively. The proposed solution is available here: https://github.com/King-HAW/ISLES22-Docker-Submission.

TEAM: VICOROB

Stroke lesion segmentation from multimodal MRI using a residual U-Net and bilateral modality augmentation

Albert Clèrigues, Arnau Oliver, Xavier Lladó

We adopt a patch-based deep learning approach using a residual 3D U-Net and small 24×24×24 patches. First, ADC and DWI images are resampled to a $1 \times 1 \times 2$ mm^3 voxel size through *sinc* interpolation, ground truth masks through nearest neighbor and the FLAIR scan is linearly registered to the resampled DWI. Then, bilateral modality augmentation is performed by flipping each modality along the sagittal plane and then registering back to the original one. These are stacked along the original modalities into a six-channel input. Input normalization is performed by remapping and clamping intensities within the 0.05% and 99.95% percentiles to the [-1, 1] interval.

For training, we use 200,000 training and 50,000 validation patches extracted with a deliberate class and lesion size balancing strategy where, half of the patches are extracted from healthy voxels and the other half from lesioned ones. For lesion patches, we first sample 10 from each individual lesion and the rest are randomly sampled. We use the DiceCELoss function from MONAI with a batch size of 16 along with the Adadelta optimizer with a 0.05 learning rate and early stopping with 5 epoch patience.

Inference is performed on highly overlapping patches extracted with a 9 × 9 × 9 step, which are later recombined into a whole image segmentation. The challenge submission used a patch-wise average ensemble of all models trained on the 5-fold cross-validation used for evaluation, which can be found at https://github.com/NIC-VICOROB/isles22-vicorob.

TEAM: PLORAS

Large-kernel Attention U-Net for Lesion Segmentation

Liam Chalcroft, Ioannis Pappas

We propose a hybrid U-Net model with a convolution-based transformer encoder consisting of 6 consecutive blocks of decreasing resolution using attention layers equivalent to a 21^3 convolution kernel based on the matrix decomposition method proposed by Guo et al. (Guo et al., 2022). The 6 blocks have output channels of (32, 64, 128, 256, 320, 320) respectively. The 5th block has 2 transformer layers, whilst all others have 1. The decoder follows the typical CNN layout of nnUNet (Isensee et al., 2018) with symmetrical channels to the encoder. Images are preprocessed via reslicing the DWI and ADC images to the FLAIR, reslicing to 1 mm, foreground cropping and z-score normalization. Training data is augmented using lesion-weighted random crop to 128^3, random flip, gaussian noise, gaussian blur and intensity shift. Training is performed for 1000 epochs with an Adam optimiser using a 5-fold ensemble, with each model trained using deep supervision for the 2 highest resolution blocks of the decoder. The dice + cross-entropy loss function is minimized in training. Final inference is performed across the ensemble using flip-based test-time augmentation. Predicted images are post-processed using a fully-connected conditional random field, before removing small holes and artefacts based on morphological operations. All training was performed using NVIDIA DALI and Auto-Mixed Precision in Pytorch Lightning, and can be trained on new data using the

implementation available at https://github.com/liamchalcroft/MDUNet. For a detailed description of the method the reader is referred to Chalcroft et al. 2023.

TEAM: MIRC

3CNN: an efficient contralateral combination CNN for automated stroke lesion segmentation on multimodal MRI

Jeroen Bertels, Ewout Heylen

The Contralateral Combination CNN (3CNN) makes a prediction for each voxel based upon a combination of features from the ipsi- and contra-lateral hemisphere. To a certain extent, 3CNN can be viewed as the natural coalescence of the contralateral information CNN (Bertels et al., 2019) and siamese network architectures (Bromley et al., 1993). The ipsi- and contralateral features target the same patterns and are extracted only once to produce the prediction of the ipsi- and contralateral voxel simultaneously. Preprocessing included aligning the FLAIR and ADC maps with the DWI map and resampling each map to an isotropic 2 mm resolution. The image intensities were projected onto [0,1] for each image individually. During training, random affine transformations, elastic distortions, and lateral flips were applied. Contralateral sampling was obtained via lateral flipping. A simplified version of the “No New-Net” (Isensee et al., 2019) served as the feature extractor. The combination of ipsi- and contralateral features was fed to three subsequent dense layers. For each ipsi- and contra-lateral voxel there were two predictions, one that was optimized with respect to cross-entropy, the other that was optimized with respect to soft Dice. Due to the use of both ipsi- and contra-lateral features, a smaller receptive field was possible, and, in combination with a smaller patch size, a virtual batch size of 16 could be obtained and batch normalization became feasible. When testing, the patch size was increased such that only a single patch was needed, and the predictions were averaged across the ipsi- and contra-lateral prediction of each voxel. All implementation details can be consulted at https://github.com/JeroenBertels/isles22.

TEAM: CREATIS

Curriculum learning on late fusion U-Net for multimodal stroke lesion segmentation

Juliette Moreau, Nima Hatami, Carole Frindel

We proposed a late fusion U-Net (Ronneberger et al., 2015) to exploit the three available modalities. To address the difficulties of the dataset we used curriculum learning (Bengio et al., 2009); scans were presented to the algorithm from easiest to more difficult based on the ratio between the volume of the bounding box and the volume of the lesion (BE) (Frindel et al., 2015). The bigger the ratio is the more the lesion is limited and/or fragmented and so considered as difficult. The curriculum based learning enables our 2D model to compete with 3D models. Training parameters were set as follows: learning rate = 0.001, decay = 0.0005, batch size = 12 and max epochs = 200. We used multiclass dice loss and Adam optimizer. Before any training, images underwent some preprocessing: first, they were all resized to the same size fixed to the most common and smallest of the dataset, i.e., 112x112 pixels; then FLAIR images were registered to DWI images and ADC maps thanks to affine and elastic transformations and finally images were normalized. For training, patients were separated in five equal random groups to proceed to 5-fold cross-validation. Each time, the training set was separated into four groups considering BE value. Thresholds were not set

regularly, namely 30, 8 and 3.5, as the patients were not evenly distributed through BE values. During training, the first fifty epochs were run with the first group, and then the second group was added to the first for the next fifty epochs, continuing until the last fifty epochs with the entire training set. The final model is an ensemble of the five models obtained from cross-validation. The final model is available here: https://github.com/JulietteMoreau/isles22_UnetLateFusion.

TEAM: DOLPHINS

Pre-Training 3D-Autoencoder based Segmentation of Ischemic Stroke Lesion using 3D-ResUnet with Deep Supervision Approach

Abdul Qayyum, Moona Mazher, Domenec Puig

We have proposed a two-stage solution for Ischemic Stroke Lesions. In a first stage, the 3D-Autoencoder model has been trained on challenge training datasets. In a second stage, a 3D-ResUnet with deep supervision has been proposed for Ischemic Stroke Lesion segmentation. The 3D-ResUnet with deep supervision is presented as an encoder, a decoder, and a baseline module. The convolutional block consists of convolutional layers with Batch-Normalization and ReLU activation function is used to extract the different feature maps from each encoder block. The input features' maps from every encoder block are concatenated with every decoder block feature maps to reconstruct the semantic information. The three-level deep-supervision techniques are applied to get the aggregate loss between ground truth and prediction. The unlabeled training ISLES'22 (Hernandez Petzsche et al., 2022) dataset is used in the 3D-Autoencoder to train the encoder. The pre-trained encoder weights obtained from the first stage 3D-Autoencoder are then used in the proposed 3D-ResUNet encoder. The random generated patches with size 96x96x96 from input volume using different augmentations are used to train the encoder of the proposed 3D-Autoencoder as well as the 3D-ResUnet. A sliding window with 8 strides is used to generate the prediction volume. The training transforms such as RandCrop, RandGaussianNoise, RandFlipping, RandShiftIntensity, RandAdjustContrast, and RandZoomd are used to train the proposed model (Cardoso et al., 2022). A learning rate of 0.0004 is used along with Adam optimizer and the cross-entropy + dice loss as the loss function. 200 epochs are used to train the proposed models. The Pytorch library using V100 tesla NVidia-GPU machine is used for model development, training, optimization, and testing. Code is publicly available at: https://github.com/RespectKnowledge/3Dsegmentation_isles2022.

TEAM: A55972-UrStroke

3D Convolutional Neural Networks for Lesion Segmentation in Multi-MR Sequence Imaging

Shao-Chieh Lin, Chun-Jung Juan

In the proposed solution, image preprocessing consisted of 1) point-by-point transforming the FLAIR image via co-registration to the DWI image and 2) applying a brain mask, extracted from the ADC maps, to remove signal and noise from CSF and outside of the brain. A multi-channel ensemble comprising DWI ('D'), ADC ('A') maps and FLAIR ('F') images with different weightings served as the input data. The original images were resampled to size 128 × 128 × 96 × N, with N (3 or 4) the number of channels, and cropped into several small volume patches (64 × 64 × 64 × N). Each patch was intentionally overlapped by a factor of 0.5 on each of three orthogonal axes for data augmentation of the training data and reduction of potential false positive prediction.

A 3D 4-layer U-Net (Ronneberger et al., 2015) was built using TensorFlow and Keras to complete the current task. The network parameters were optimized using the ADAM algorithm with BCE loss function. Learning rate scheduling and early stopping function were applied for better convergence and avoiding overfitting. Finally, a total of three models, including model-ADF, model-DDF, and model ADDF, were trained using 250 cases provided by ISLES'22 (Hernandez Petzsche et al., 2022), including 150 cases in the training stage and another 100 cases in the validation stage.
The predicted patches were reconstructed back into the full volume in each case. Dice similarity coefficient (DSC) was calculated to select the best probability threshold of each model and to generate the binary segmentation. Finally, majority voting was applied to the predictions of the three models to obtain the final prediction of stroke lesions.
The proposed solution is publicly available at: https://github.com/m0705327/ISLES-22-UrStroke.git.

TEAM: AICONS

Deep-Learning Based Framework for Automated Stroke Lesion Segmentation in Multimodal MRI Data

Tianxi Hu*, Lyndon Boone*, Maged Goubran
* Equal contributions.

In our solution, the ISLES'22 dataset is used with an 80% / 20% split between training and validation. ADC and DWI images are used as model inputs and preprocessed with channel-wise intensity normalization. Images are then flipped across the left-right axis and concatenated with the original ones to add a second channel as input. As many stroke lesions are unilateral compared to normal brain tissues which are more symmetric, this enhances the contrast between the two (Li et al., 2020). Image contrast and zoom are randomly adjusted for data augmentation, and 96 mm^3 patches are randomly sampled to be used as inputs. For our model architecture, a 5-layer 3D residual U-Net deep neural network (Kerfoot et al., 2019) is used with 2 convolution layers per residual block (each followed by activation, normalization, and dropout), and a dropout rate of 0.2. Training is performed over 500 epochs with batch size of 8 image patches and early stopping at 125 epochs to prevent overfitting. Dice Focal Loss is used along with ADAM optimizer and a learning rate of 1×10^{-4}. Model performance is evaluated using Dice Similarity Score (DSC) and Hausdorff Distance. Validation is performed on the entire validation set every 2 training epochs which computes a mean DSC and saves the model parameters if a best new DSC is recorded. The proposed solution is available at https://github.com/tianxic-hu/isles22_aicons_algorithm.

References

Bengio, Y., Louradour, J., Collobert, R. and Weston, J., 2009, June. Curriculum learning. In *Proceedings of the 26th annual international conference on machine learning* (pp. 41-48).

Chalcroft, L., Pereira, R.L., Brudfors, M., Kayser, A.S., D'Esposito, M., Price, C.J., Pappas, I. and Ashburner, J., 2023. Large-kernel Attention for Efficient and Robust Brain Lesion Segmentation. *arXiv preprint* arXiv:2308.07251.

Bertels, J., Robben, D., Vandermeulen, D. and Suetens, P., 2019. Contra-lateral information CNN for core lesion segmentation based on native CTP in acute stroke. In *Brainlesion: Glioma, Multiple Sclerosis, Stroke and*

Traumatic Brain Injuries: 4th International Workshop, BrainLes 2018, Held in Conjunction with MICCAI 2018, Granada, Spain, September 16, 2018, Revised Selected Papers, Part I 4 (pp. 263-270). Springer International Publishing.

Bromley, J., Guyon, I., LeCun, Y., Säckinger, E. and Shah, R., 1993. Signature verification using a “siamese” time delay neural network. *Advances in neural information processing systems*, *6*.

Cardoso, M.J., Li, W., Brown, R., Ma, N., Kerfoot, E., Wang, Y., Murrey, B., Myronenko, A., Zhao, C., Yang, D. and Nath, V., 2022. MONAI: An open-source framework for deep learning in healthcare. *arXiv preprint arXiv:2211.02701*.

Frindel, C., Rouanet, A., Giacalone, M., Cho, T.H., Østergaard, L., Fiehler, J., Pedraza, S., Baron, J.C., Wiart, M., Berthezène, Y. and Nighoghossian, N., 2015. Validity of shape as a predictive biomarker of final infarct volume in acute ischemic stroke. *Stroke*, *46*(4), pp.976-981.

Guo, M.H., Lu, C.Z., Liu, Z.N., Cheng, M.M. and Hu, S.M., 2023. Visual attention network. *Computational Visual Media*, *9*(4), pp.733-752.

He, K., Zhang, X., Ren, S. and Sun, J., 2016. Deep residual learning for image recognition. In *Proceedings of the IEEE conference on computer vision and pattern recognition* (pp. 770-778).

Hernandez Petzsche, M.R., de la Rosa, E., Hanning, U., Wiest, R., Valenzuela, W., Reyes, M., Meyer, M., Liew, S.L., Kofler, F., Ezhov, I. and Robben, D., 2022. ISLES 2022: A multi-center magnetic resonance imaging stroke lesion segmentation dataset. *Scientific data*, *9*(1), p.762.

Isensee, F., Petersen, J., Klein, A., Zimmerer, D., Jaeger, P.F., Kohl, S., Wasserthal, J., Koehler, G., Norajitra, T., Wirkert, S. and Maier-Hein, K.H., 2018. nnU-Net: Self-adapting framework for U-Net-based medical image segmentation. *arXiv preprint arXiv:1809.10486*.

Isensee, F., Jaeger, P.F., Kohl, S.A., Petersen, J. and Maier-Hein, K.H., 2021. nnU-Net: a self-configuring method for deep learning-based biomedical image segmentation. *Nature methods*, *18*(2), pp.203-211.

Isensee, F., Kickingereder, P., Wick, W., Bendszus, M. and Maier-Hein, K.H., 2019. No New-Net. In *Brainlesion: Glioma, Multiple Sclerosis, Stroke and Traumatic Brain Injuries: 4th International Workshop, BrainLes 2018, Held in Conjunction with MICCAI 2018, Granada, Spain, September 16, 2018, Revised Selected Papers, Part II 4* (pp. 234-244). Springer International Publishing.

Kerfoot, E., Clough, J., Oksuz, I., Lee, J., King, A.P. and Schnabel, J.A., 2019. Left-ventricle quantification using residual U-Net. In *Statistical Atlases and Computational Models of the Heart. Atrial Segmentation and LV Quantification Challenges: 9th International Workshop, STACOM 2018, Held in Conjunction with MICCAI 2018, Granada, Spain, September 16, 2018, Revised Selected Papers 9* (pp. 371-380). Springer International Publishing.

Li, L., Wei, M., Liu, B., Atchaneeyasakul, K., Zhou, F., Pan, Z., Kumar, S.A., Zhang, J.Y., Pu, Y., Liebeskind, D.S. and Scalzo, F., 2020. Deep learning for hemorrhagic lesion detection and segmentation on brain CT images. *IEEE journal of biomedical and health informatics*, *25*(5), pp.1646-1659.

Ronneberger, O., Fischer, P. and Brox, T., 2015. U-net: Convolutional networks for biomedical image segmentation. In *Medical Image Computing and Computer-Assisted Intervention–MICCAI 2015: 18th*

International Conference, Munich, Germany, October 5-9, 2015, Proceedings, Part III 18 (pp. 234-241). Springer International Publishing.

Supplementary material #2.

Supplementary Material for

DeepISLES: A Clinically Validated Ischemic Stroke Segmentation Model from the ISLES'22 Challenge

de la Rosa, E. et al.

Rank	Team	DSC ↑	AVD (ml) ↓	F1 ↑	ALD ↓
*1	DeepISLES	0.82 ± 0.12	1.59 ± 4.40	0.86 ± 0.21	1.00 ± 3.00
1	SEALS	0.82 ± 0.12	1.63 ± 5.43	0.86 ± 0.19	1.00 ± 3.00
2	NVAUTO	0.82 ± 0.12	1.63 ± 4.27	0.80 ± 0.23	2.00 ± 2.75
3	SWAN	0.81 ± 0.15	1.94 ± 4.40	0.80 ± 0.24	2.00 ± 2.75
3	PAT	0.82 ± 0.15	1.95 ± 4.71	0.82 ± 0.30	1.00 ± 3.00
5	CTRL	0.80 ± 0.14	2.15 ± 5.32	0.79 ± 0.23	1.50 ± 3.00
6	VICOROB	0.78 ± 0.18	2.04 ± 5.29	0.74 ± 0.31	2.00 ± 3.00
7	PLORAS	0.76 ± 0.16	1.69 ± 4.71	0.73 ± 0.33	2.00 ± 3.00
8	MIRC	0.76 ± 0.21	2.78 ± 6.12	0.67 ± 0.27	2.00 ± 4.00
9	CREATIS	0.71 ± 0.18	3.30 ± 7.16	0.67 ± 0.25	3.00 ± 5.00
10	Dolphins	0.74 ± 0.21	2.44 ± 6.15	0.60 ± 0.38	3.00 ± 5.75
11	A55972	0.06 ± 0.22	10.49 ± 29.17	0.44 ± 0.44	4.00 ± 6.78
12	AICONS	0.40 ± 0.50	18.86 ± 31.59	0.26 ± 0.19	5.00 ± 6.00

Table S2.1. Final ISLES'22 ranking obtained over the unseen, test-phase data. Median ± interquartile range values are displayed. *Result obtained in an emulated post-challenge setting by including an ensemble algorithm with the submissions of teams SEALS, NVAUTO and SWAN and by re-computing the challenge ranking. The best median value of each metric is displayed in bold. DSC: Dice Similarity Coefficient (DSC); F1 score: lesion-wise F1 score; AVD: absolute volume difference; ALD: absolute lesion count difference.

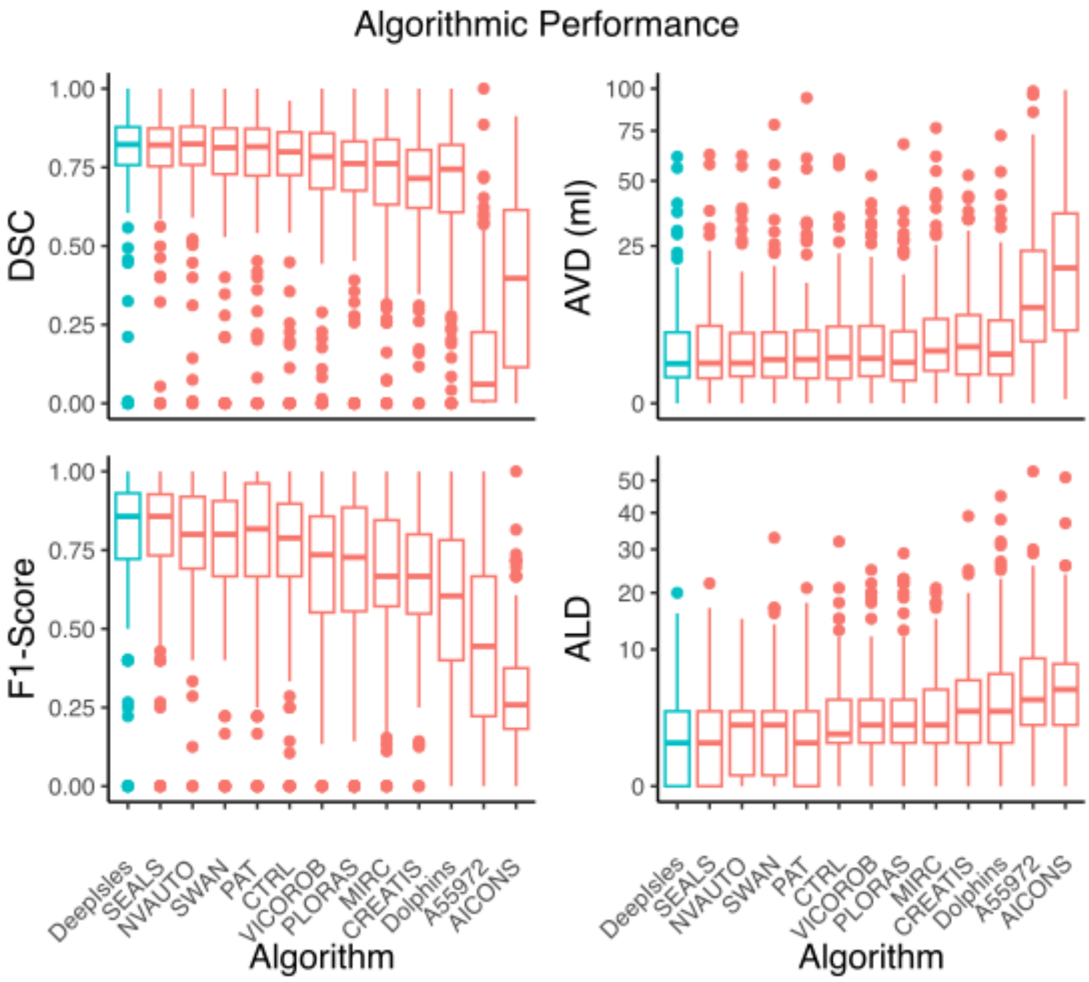

Figure S2.1. Performance for the participating teams in the unseen test phase of the challenge. Teams are displayed in red and in decreasing order based on their final rank. DSC: Dice Similarity Coefficient; F1 score: lesion-wise F1 score; AVD: absolute volume difference; ALD: absolute lesion count difference. y-axis for AVD and ALD boxplots are displayed using a non-linear scale to enhance data visibility.

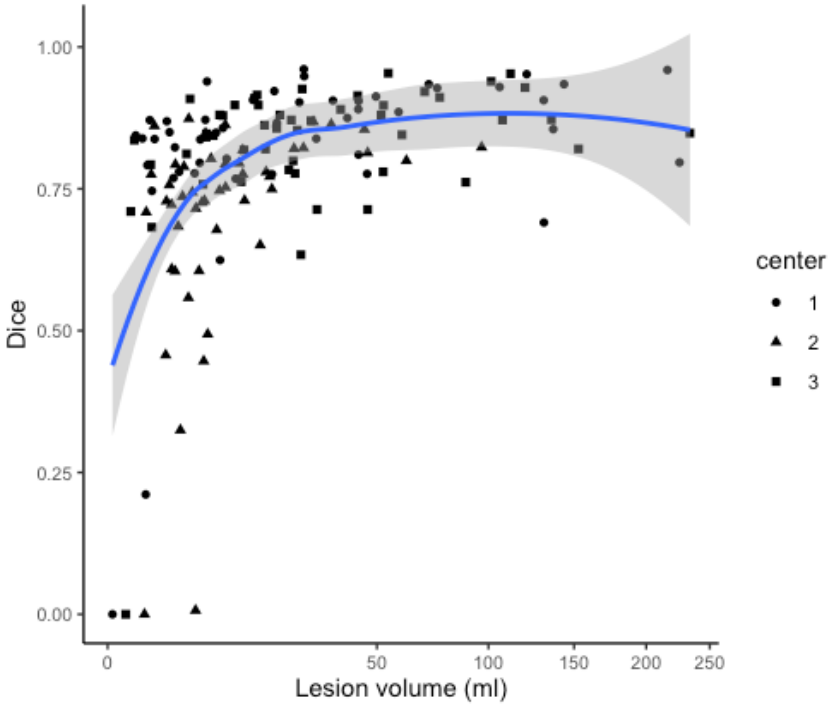

Figure S2.2. DeepISLES Dice vs lesion volumes on the ISLES'22 test set. There is a non-linear correlation between lesion size and Dice scores.

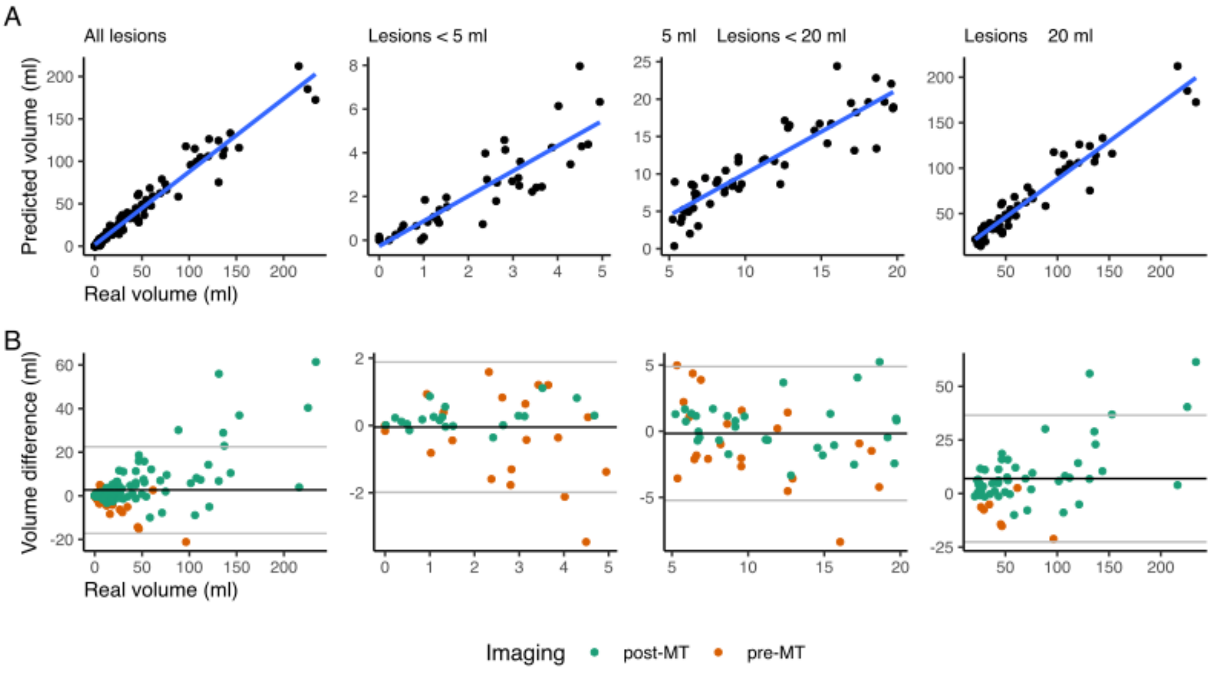

Figure S2.3. Volumetric lesion agreement (DeepISLES vs. ground truth) across different ischemic lesion sizes. There is a high volumetric agreement for all lesion sizes, with Pearson r = 0.98 when considering all lesion sizes, r = 0.87 for lesions smaller than 5 ml, r = 0.90 for lesions equal or larger than 5 ml but smaller than 20 ml, and r = 0.96 for lesions larger or equal than 20 ml. A) Scatter plots (predicted volumes vs. ground truth volumes). In blue, a linear regression line is shown. B) Bland-altman plots. The y-axis (volume difference) is calculated as the difference between the real (ground truth) volume and DeepISLES -predicted volume. The black line is plotted at the mean of the volume difference. The gray lines are plotted at 1.96 times the standard deviation of the volume difference. MT: mechanical thrombectomy.

Supplementary material #3.

Benchmarking report for ISLES'22 - MICCAI Challenge

created by challengeR v1.0.5

06 December, 2024

This document presents a systematic report on the benchmark study "ISLES'22 - MICCAI Challenge". Input data comprises raw metric values for all algorithms and cases. Generated plots are:

- Visualization of assessment data: Dot- and boxplots, podium plots and ranking heatmaps
- Visualization of ranking stability: Blob plots, violin plots and significance maps, line plots
- Visualization of cross-task insights: Blob plots, stacked frequency plots, dendrograms

Details can be found in Wiesenfarth et al. (2021).

1 Rankings

Algorithms within a task are ranked according to the following ranking scheme:

rank then aggregate using function ("mean")

Ranking for each task:

AVD : The analysis is based on 12 algorithms and 150 cases. 0 missing cases have been found in the data set.

	rank_mean	rank
NVAUTO	4.733333	1
SEALS	4.900000	2
SWAN	5.020000	3
PLORAS	5.306667	4
PAT	5.460000	5
VICOROB	5.540000	6
CTRL	5.633333	7
Dolphins	6.006667	8
CREATIS	6.440000	9
MIRC	7.206667	10
AICONS	9.953333	11
A55972	11.020000	12

Table S3.1: AVD Ranking.

Dice : The analysis is based on 12 algorithms and 150 cases. 0 missing cases have been found in the data set.

	rank_mean	rank
NVAUTO	2.920000	1

	rank_mean	rank
SEALS	3.260000	2
SWAN	3.766667	3
PAT	4.213333	4
CTRL	4.546667	5
VICOROB	5.573333	6
PLORAS	7.093333	7
MIRC	7.393333	8
Dolphins	7.546667	9
CREATIS	7.846667	10
AICONS	10.706667	11
A55972	11.380000	12

Table S3.2: Dice Ranking.

F1 : The analysis is based on 12 algorithms and 150 cases. 0 missing cases have been found in the data set.

	rank_mean	rank
SEALS	2.293333	1
NVAUTO	3.220000	2
PAT	3.253333	3
SWAN	3.646667	4
CTRL	3.720000	5
VICOROB	5.060000	6
PLORAS	5.313333	7
MIRC	5.473333	8
CREATIS	6.493333	9
Dolphins	7.420000	10
A55972	8.893333	11
AICONS	10.860000	12

Table S3.3: F1 Score Ranking.

LCD : The analysis is based on 12 algorithms and 150 cases. 0 missing cases have been found in the data set.

	rank_mean	rank
SEALS	3.366667	1
PAT	3.866667	2
NVAUTO	3.980000	3
SWAN	4.080000	4
CTRL	4.506667	5
VICOROB	5.193333	6
PLORAS	5.353333	7
MIRC	5.393333	8
CREATIS	6.173333	9
Dolphins	6.246667	10
AICONS	8.006667	11
A55972	8.173333	12

Table S3.4: LCD Ranking.

Consensus ranking across tasks according to chosen method "euclidean":

	value	rank
SEALS	1.50	1
NVAUTO	1.75	2
PAT	3.50	3
SWAN	3.50	3
CTRL	5.50	5
VICOROB	6.00	6
PLORAS	6.25	7
MIRC	8.50	8
CREATIS	9.25	9
Dolphins	9.25	9
AICONS	11.25	11
A55972	11.75	12

Table S3.5: Consensus Ranking.

2 Visualization of raw assessment data

The algorithms are ordered according to the computed ranks for each task.

2.1 Dot- and boxplot

Dot- and boxplots for visualizing raw assessment data separately for each algorithm. Boxplots representing descriptive statistics over all cases (median, quartiles and outliers) are combined with horizontally jittered dots representing individual cases.

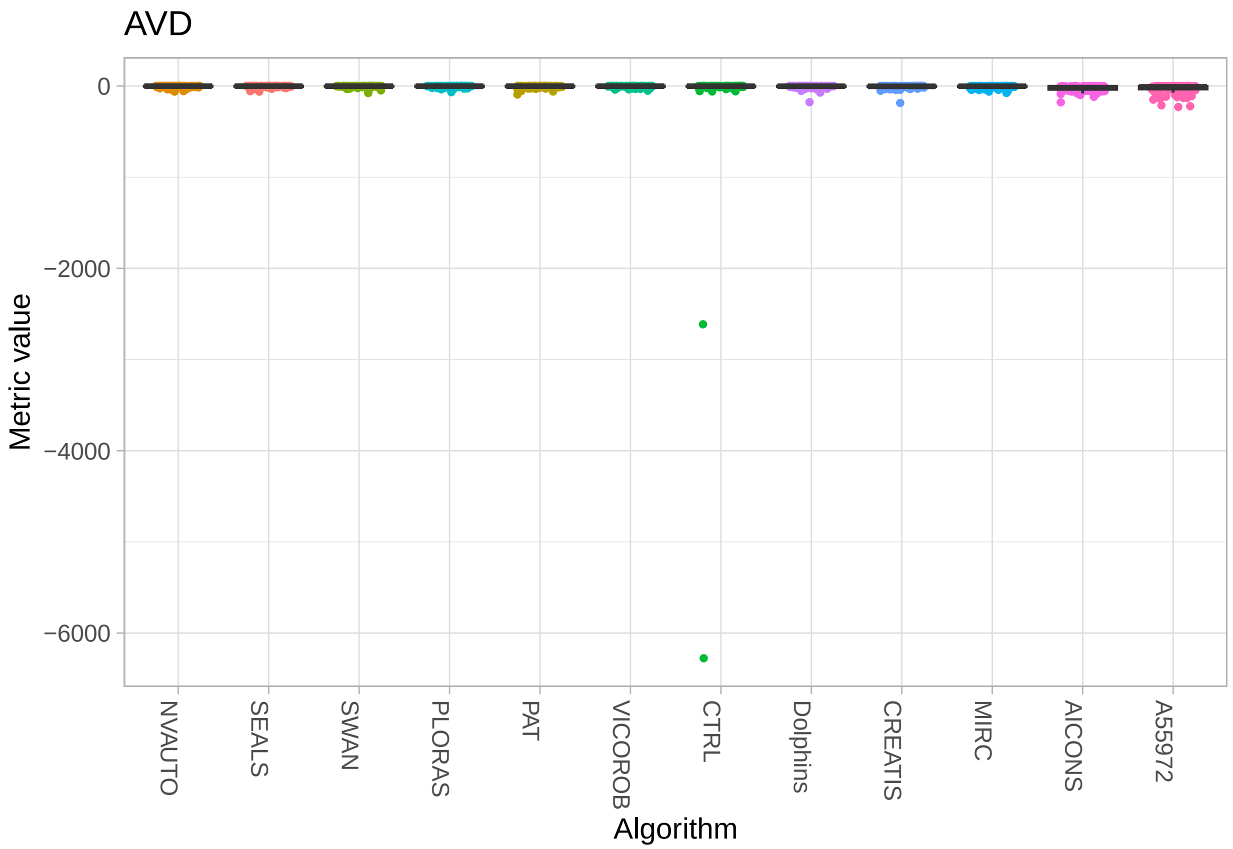

Figure S3.1: AVD Boxplots.

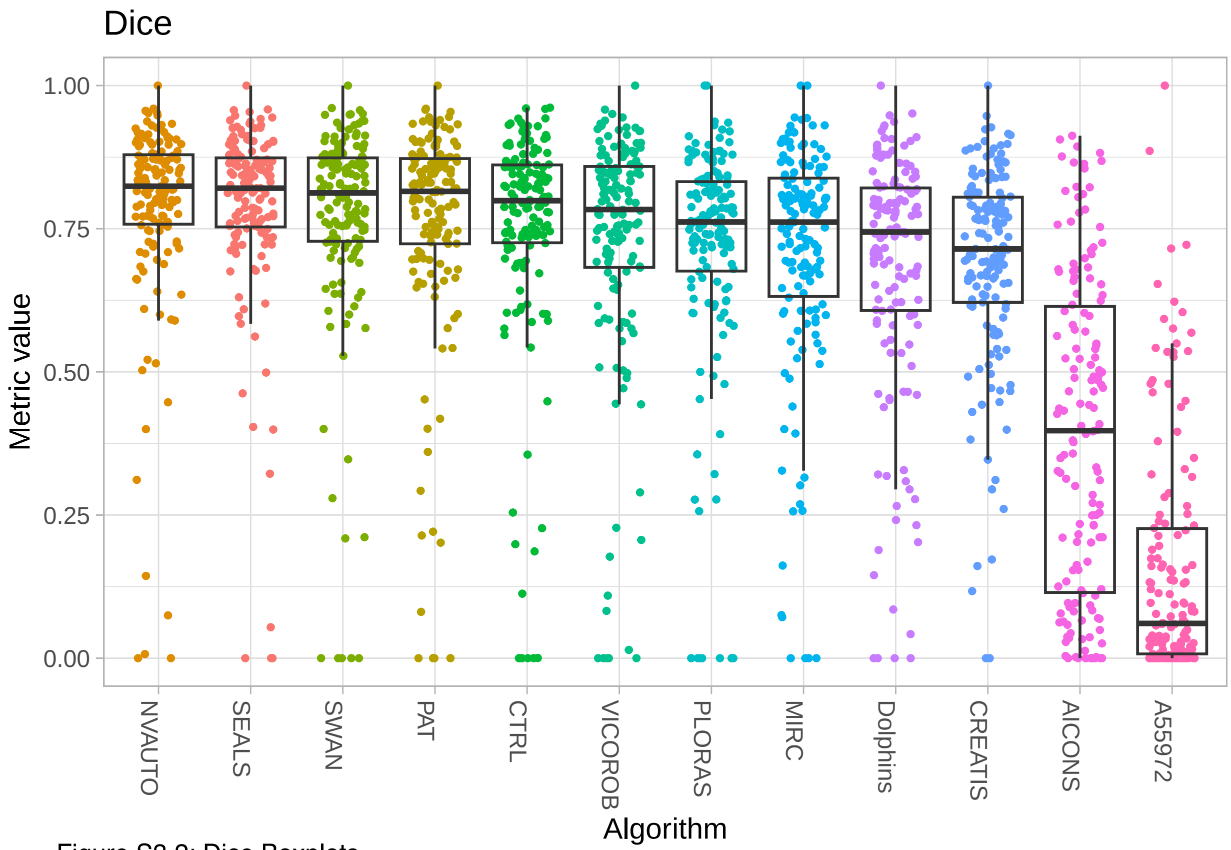

Figure S3.2: Dice Boxplots.

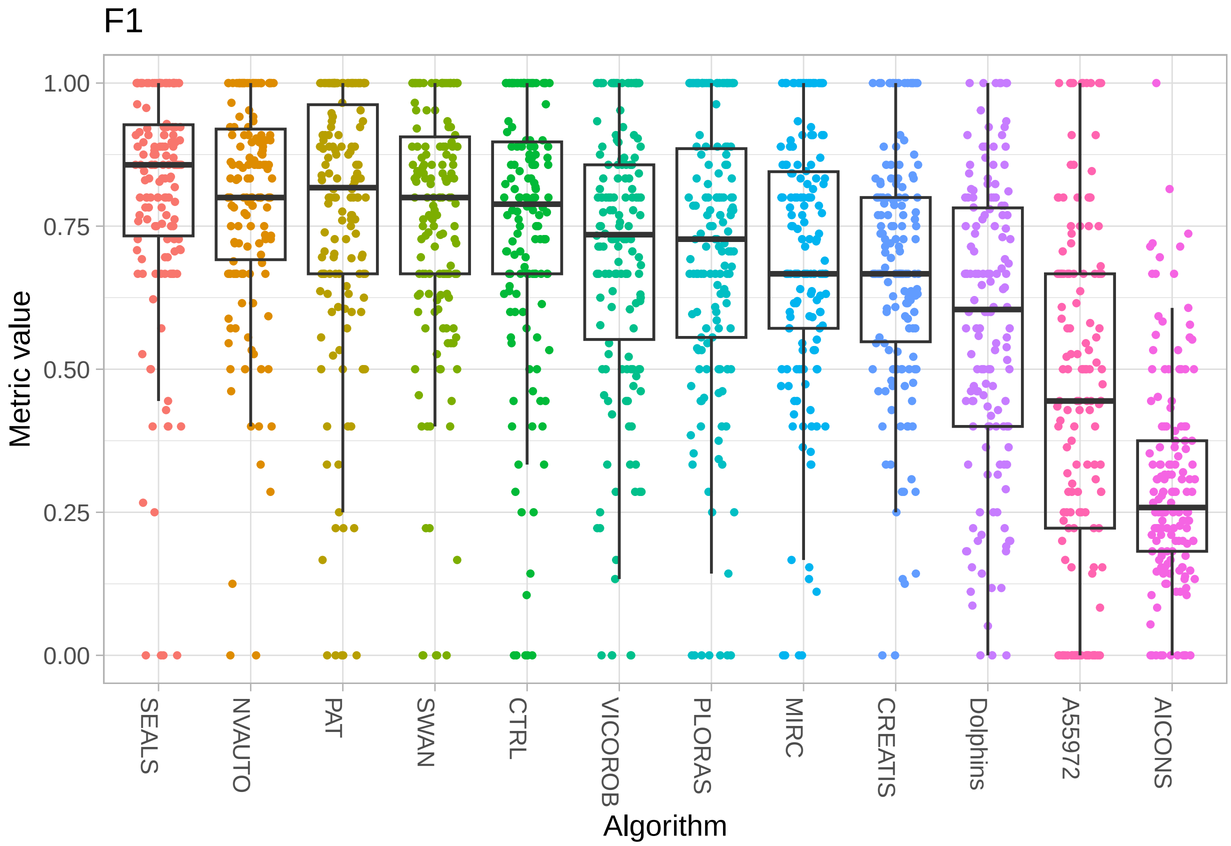

Figure S3.3: F1 Boxplots.

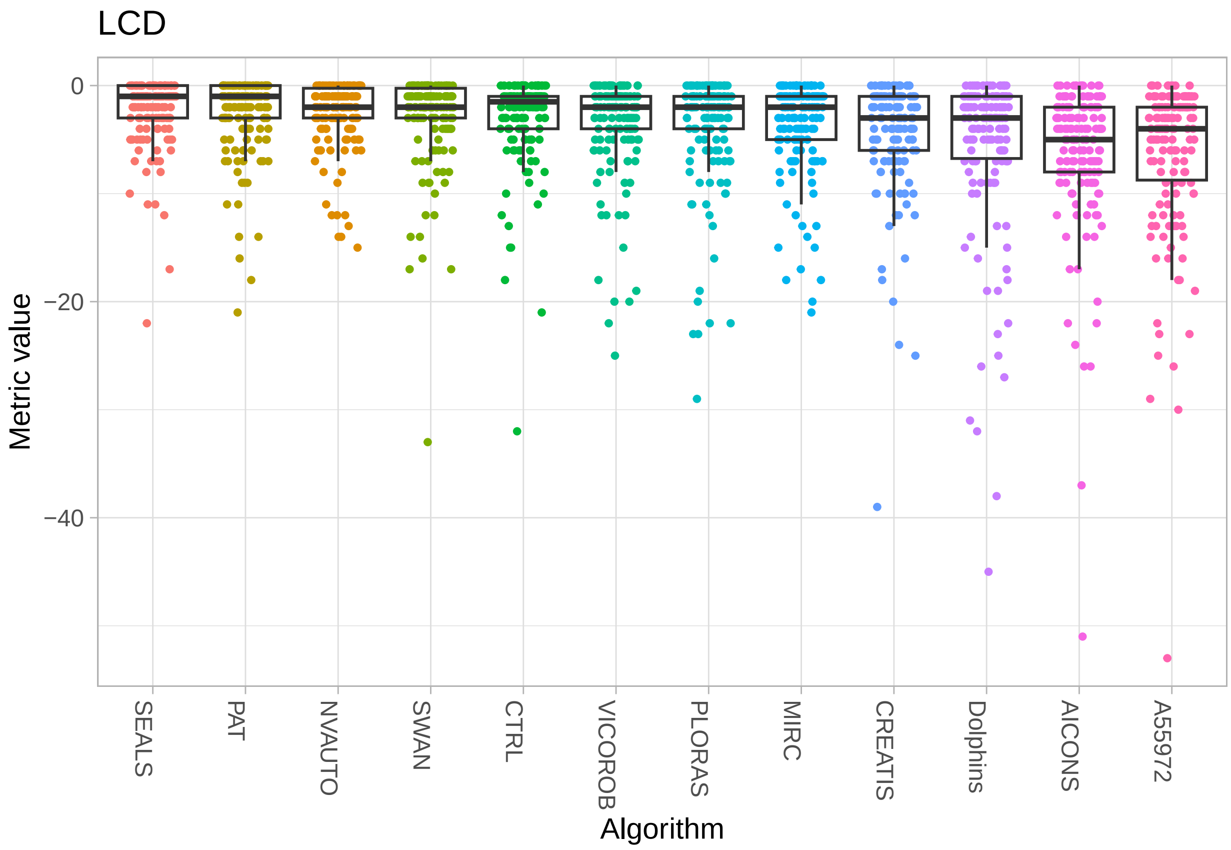

Figure S3.4: LCD Boxplots.

2.2 Podium plot

Podium plots (see also Eugster et al., 2008) for visualizing raw assessment data. Upper part (spaghetti plot): Participating algorithms are color-coded, and each colored dot in the plot represents a metric value achieved with the respective algorithm. The actual metric value is encoded by the y-axis. Each podium (here: p=12) represents one possible rank, ordered from best (1) to last (here: 12). The assignment of metric values (i.e. colored dots) to one of the podiums is based on the rank that the respective algorithm achieved on the corresponding case. Note that the plot part above each podium place is further subdivided into p "columns", where each column represents one participating algorithm (here: p = 12). Dots corresponding to identical cases are connected by a line, leading to the shown spaghetti structure. Lower part: Bar charts represent the relative frequency for each algorithm to achieve the rank encoded by the podium place.

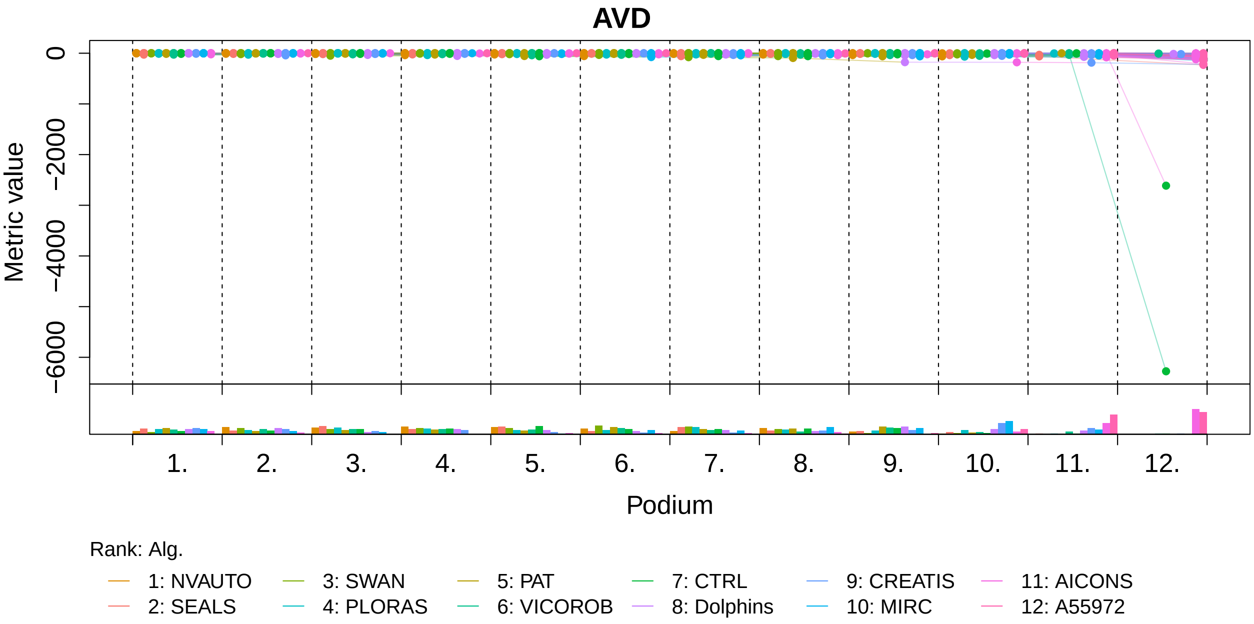

Figure S3.5: AVD Podium Plot.

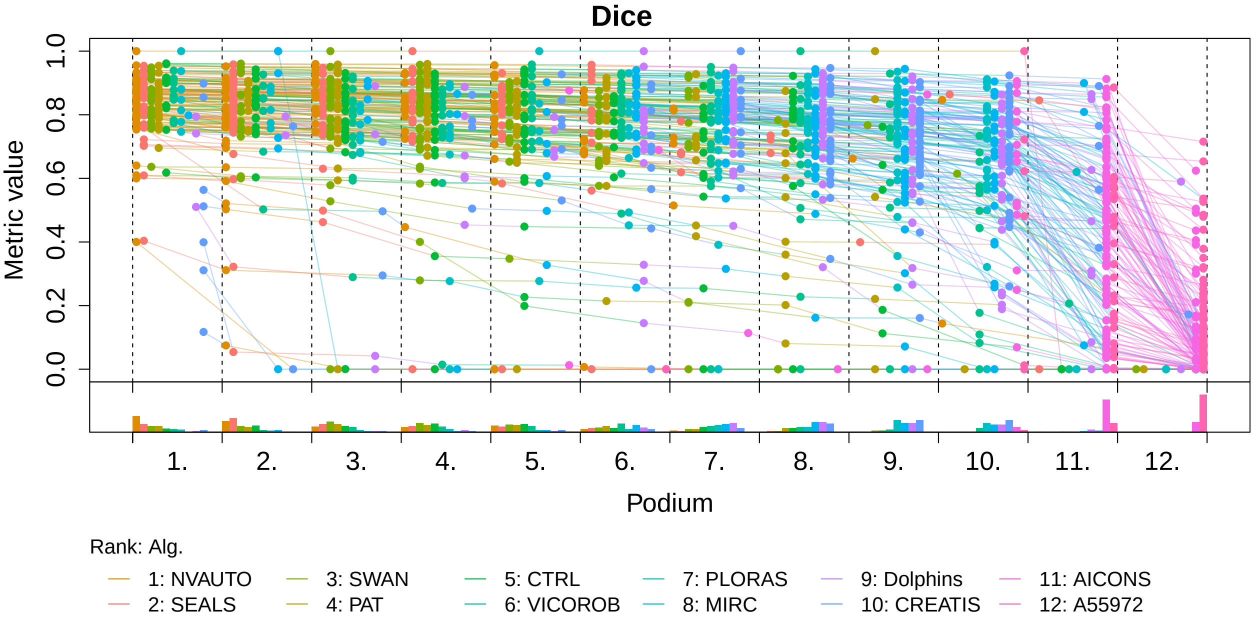

Figure S3.6: Dice Podium Plot.

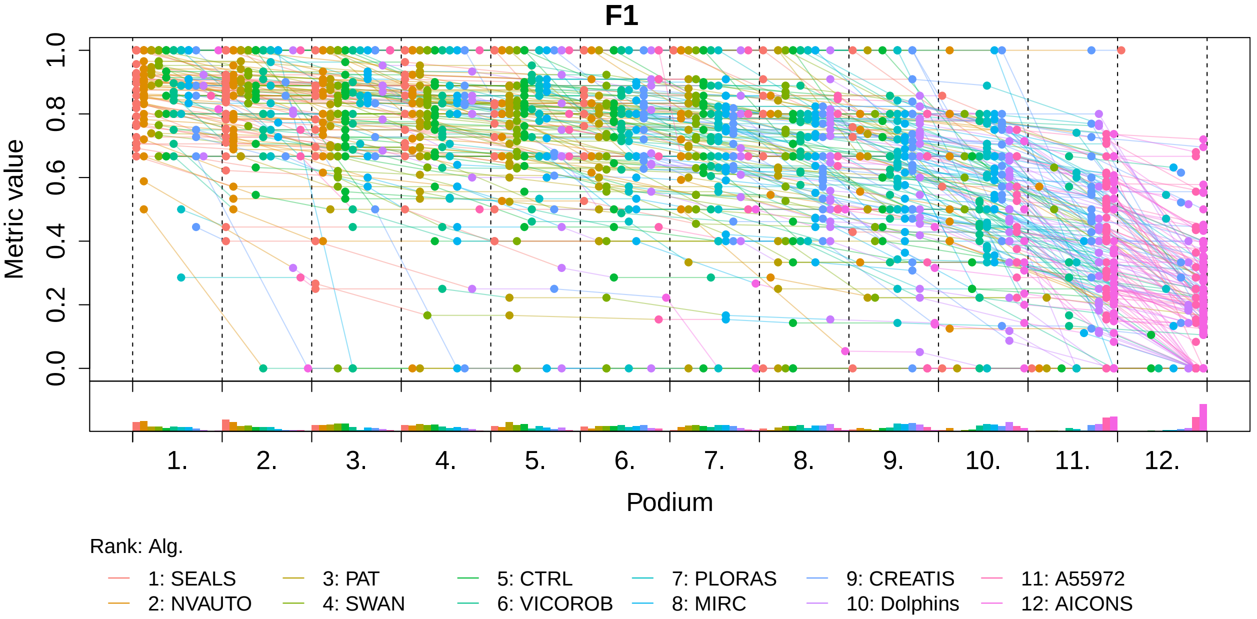

Figure S3.7: F1 Podium Plot.

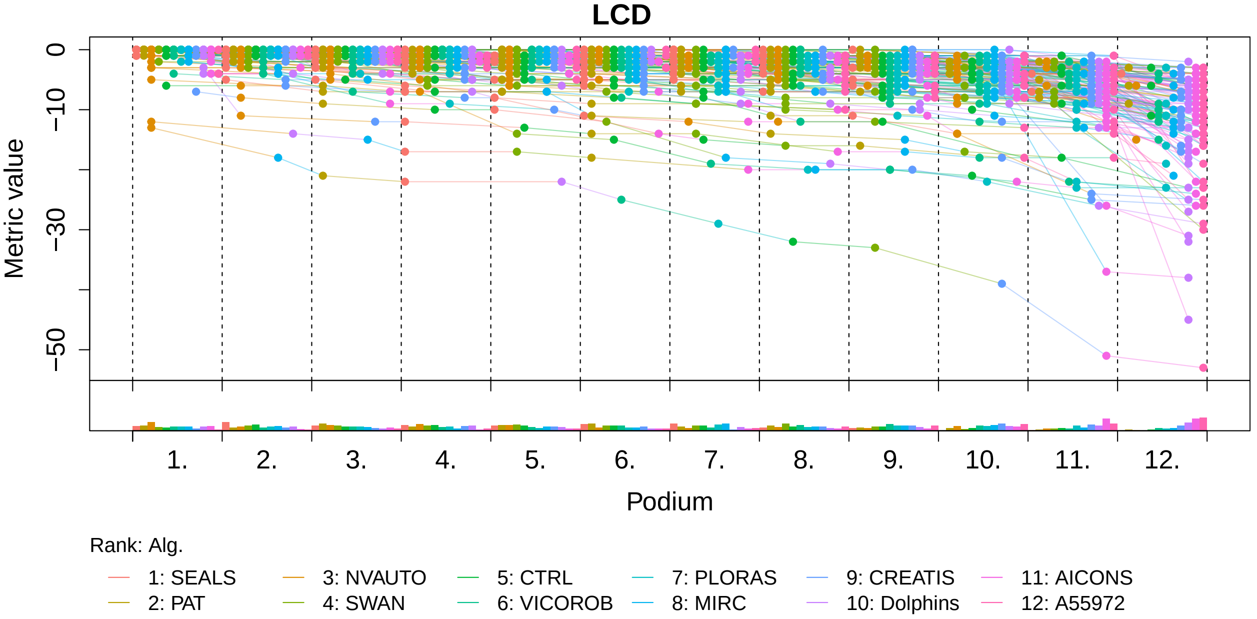

Figure S3.8: LCD Podium Plot.

2.3 Ranking heatmap

Ranking heatmaps for visualizing raw assessment data. Each cell (i, A_j) shows the absolute frequency of cases in which algorithm A_j achieved rank i.

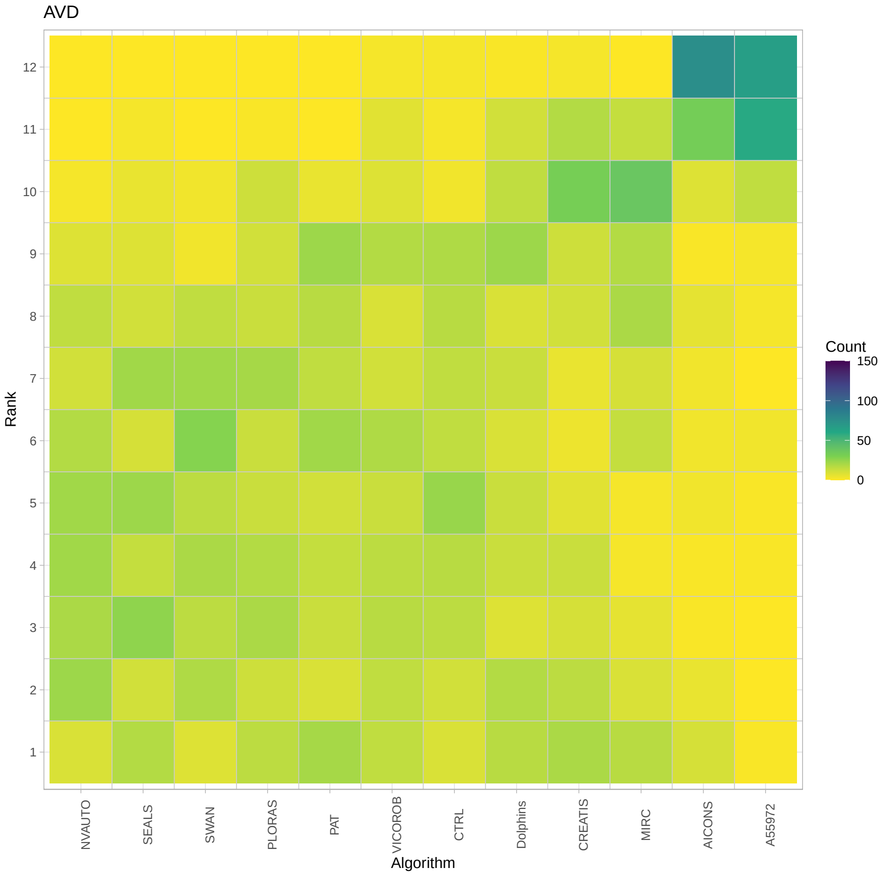

Figure S3.9: AVD Ranking Heatmap.

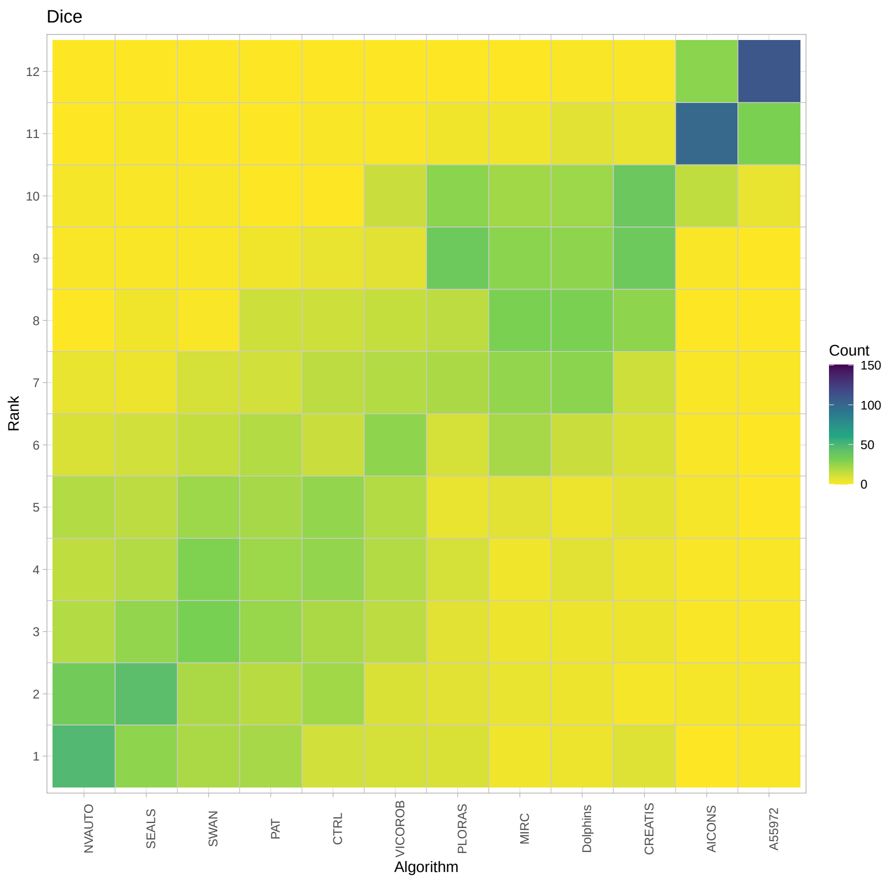

Figure S3.10: Dice Ranking Heatmap.

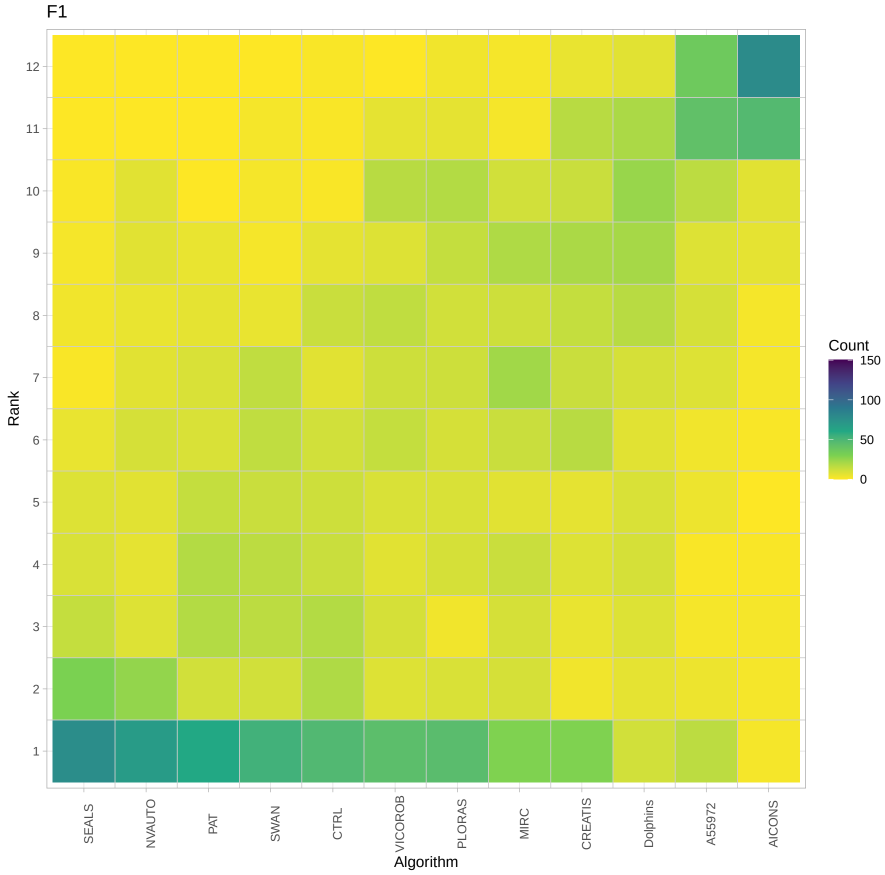

Figure S3.11: F1 Ranking Heatmap.

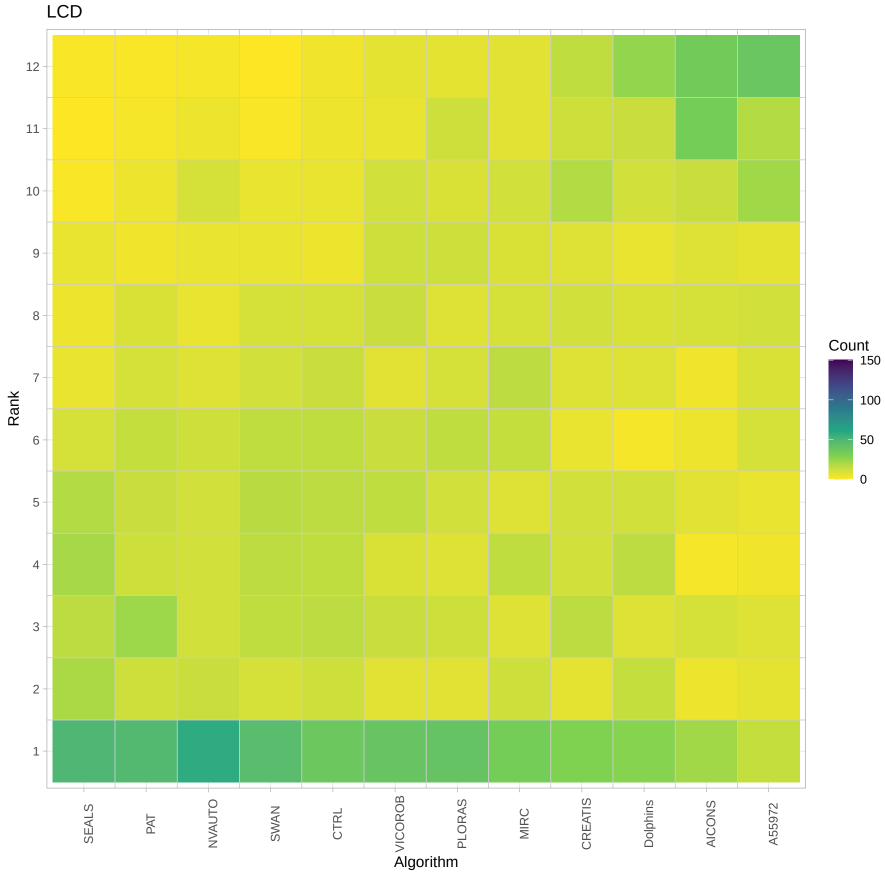

Figure S3.12: LCD Ranking Heatmap.

3 Visualization of ranking stability

3.1 *Blob plot* for visualizing ranking stability based on bootstrap sampling

Algorithms are color-coded, and the area of each blob at position $(A_i, \text{rank } j)$ is proportional to the relative frequency A_i achieved rank j across $b = 1000$ bootstrap samples. The median rank for each algorithm is indicated by a black cross. 95% bootstrap intervals across bootstrap samples are indicated by black lines.

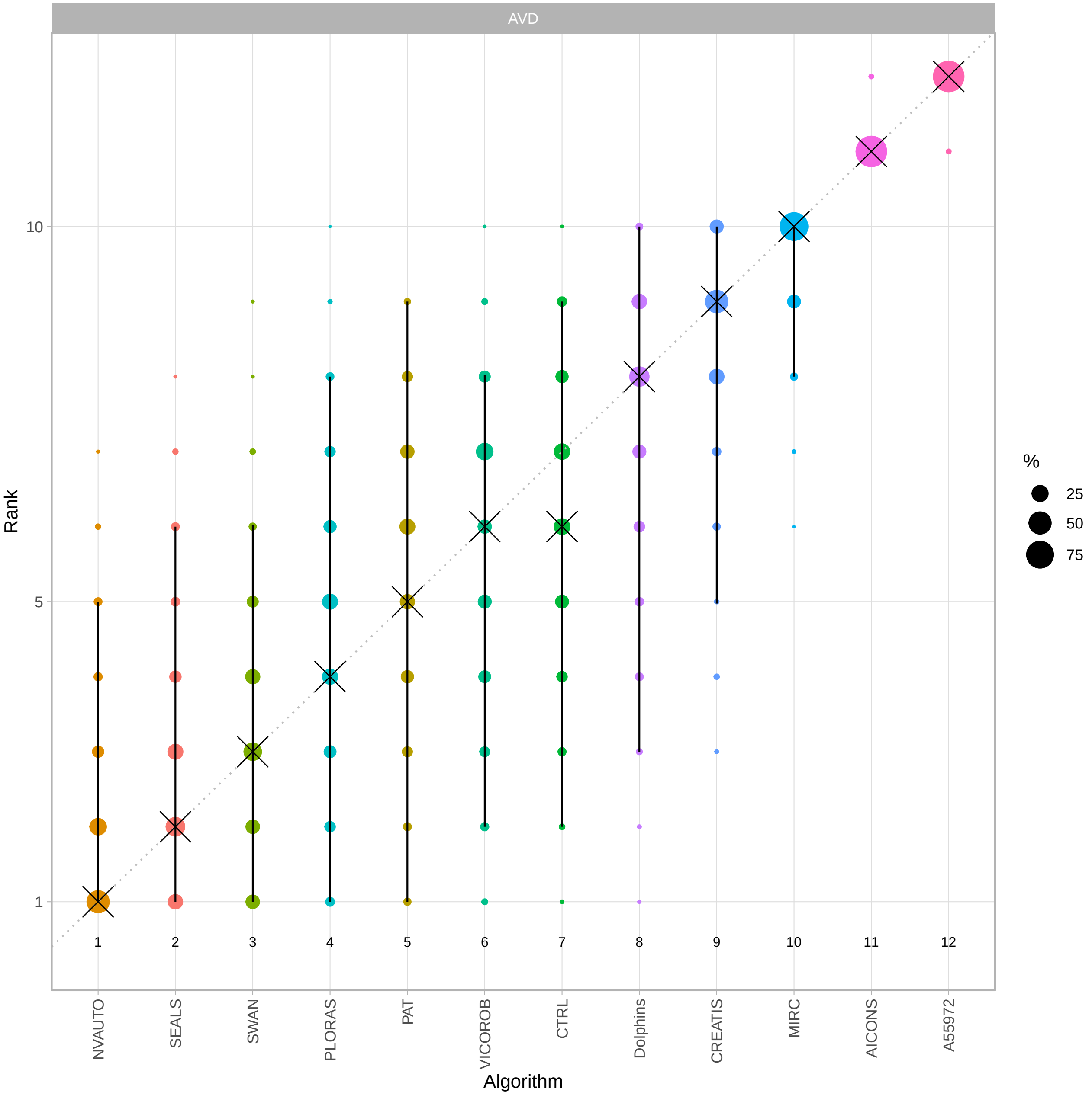

Figure S3.13: AVD Ranking Stability – Blob Plot.

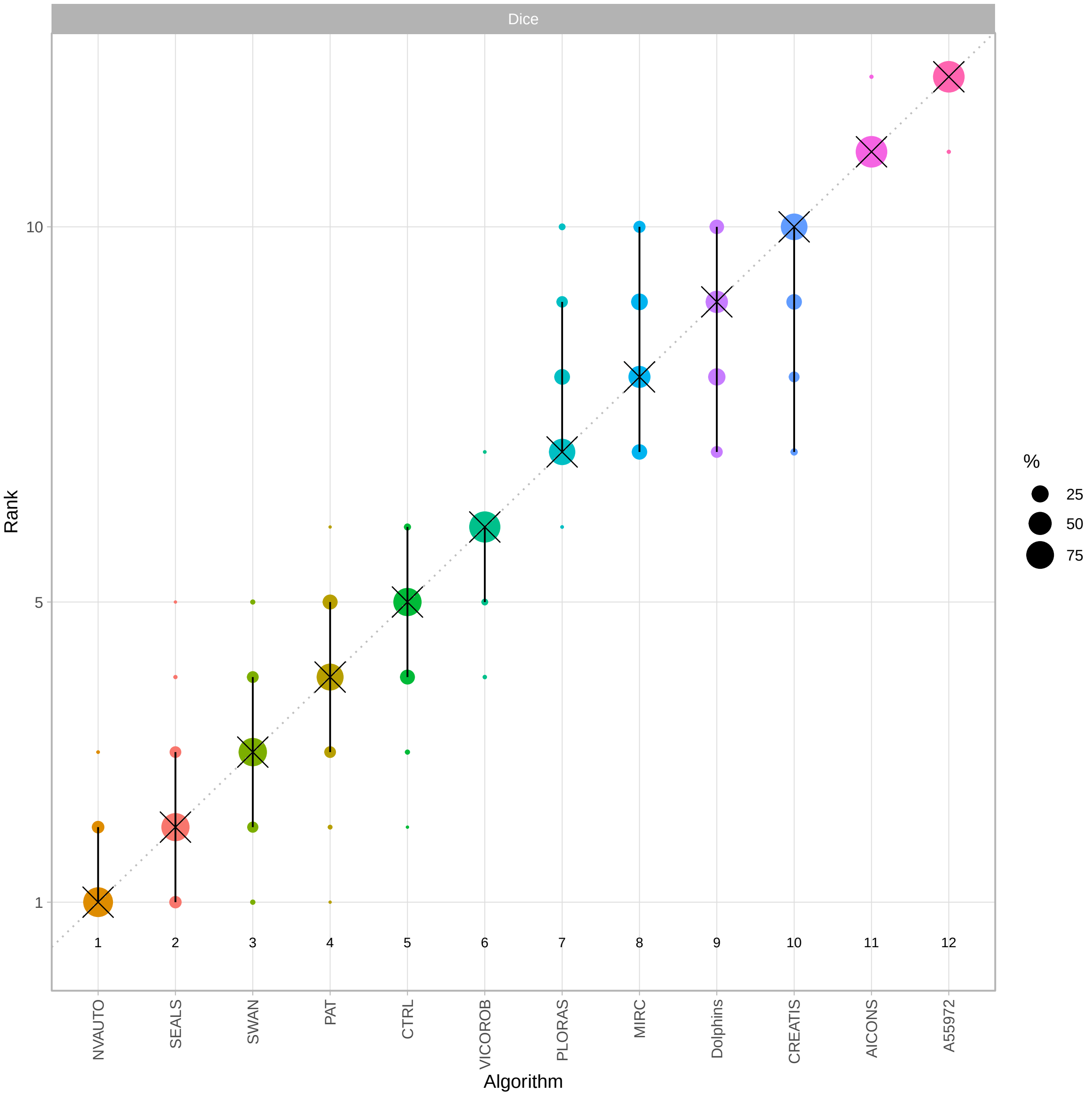

Figure S3.14: Dice Ranking Stability – Blob Plot.

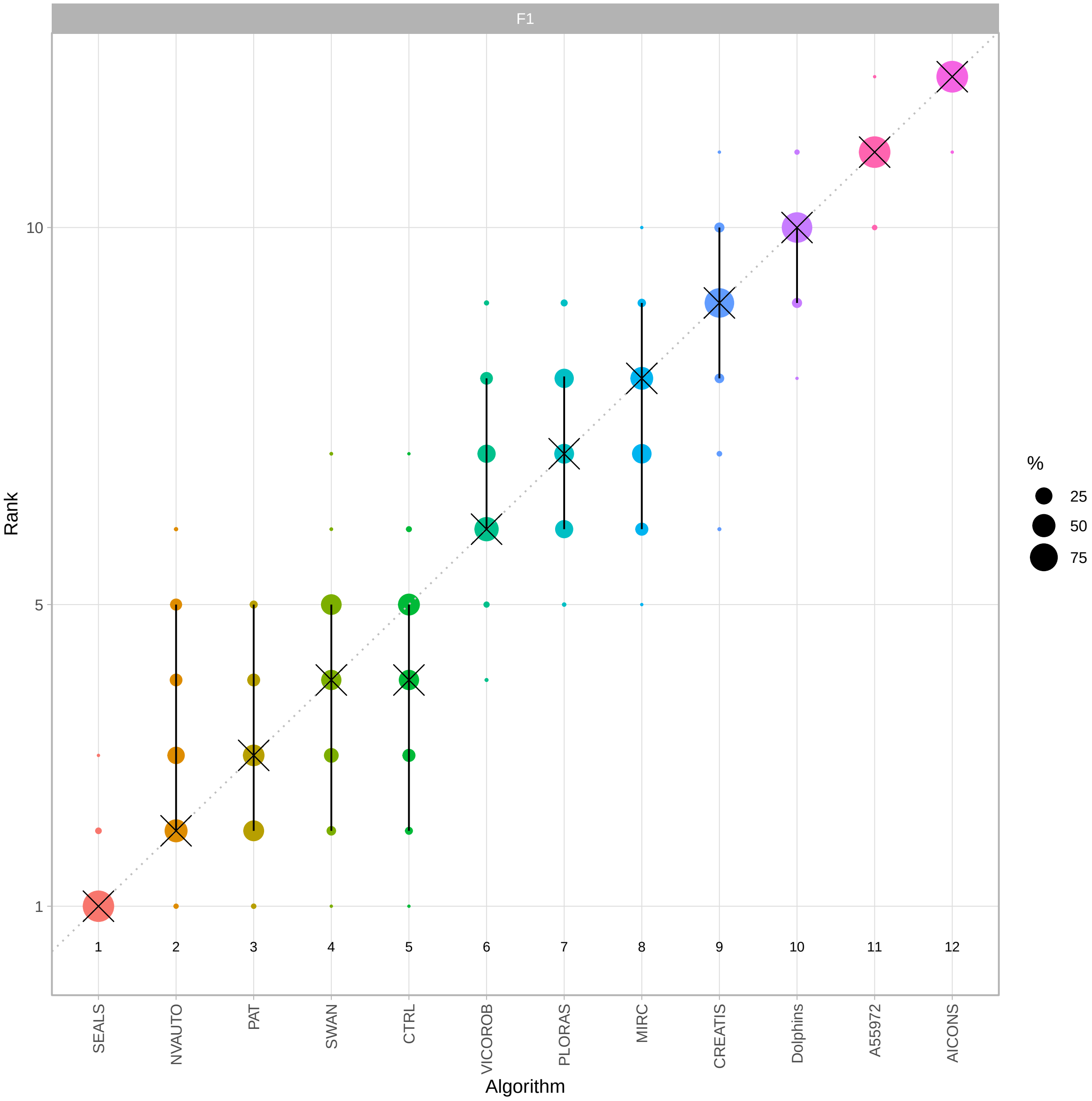

Figure S3.15: F1 Ranking Stability – Blob Plot.

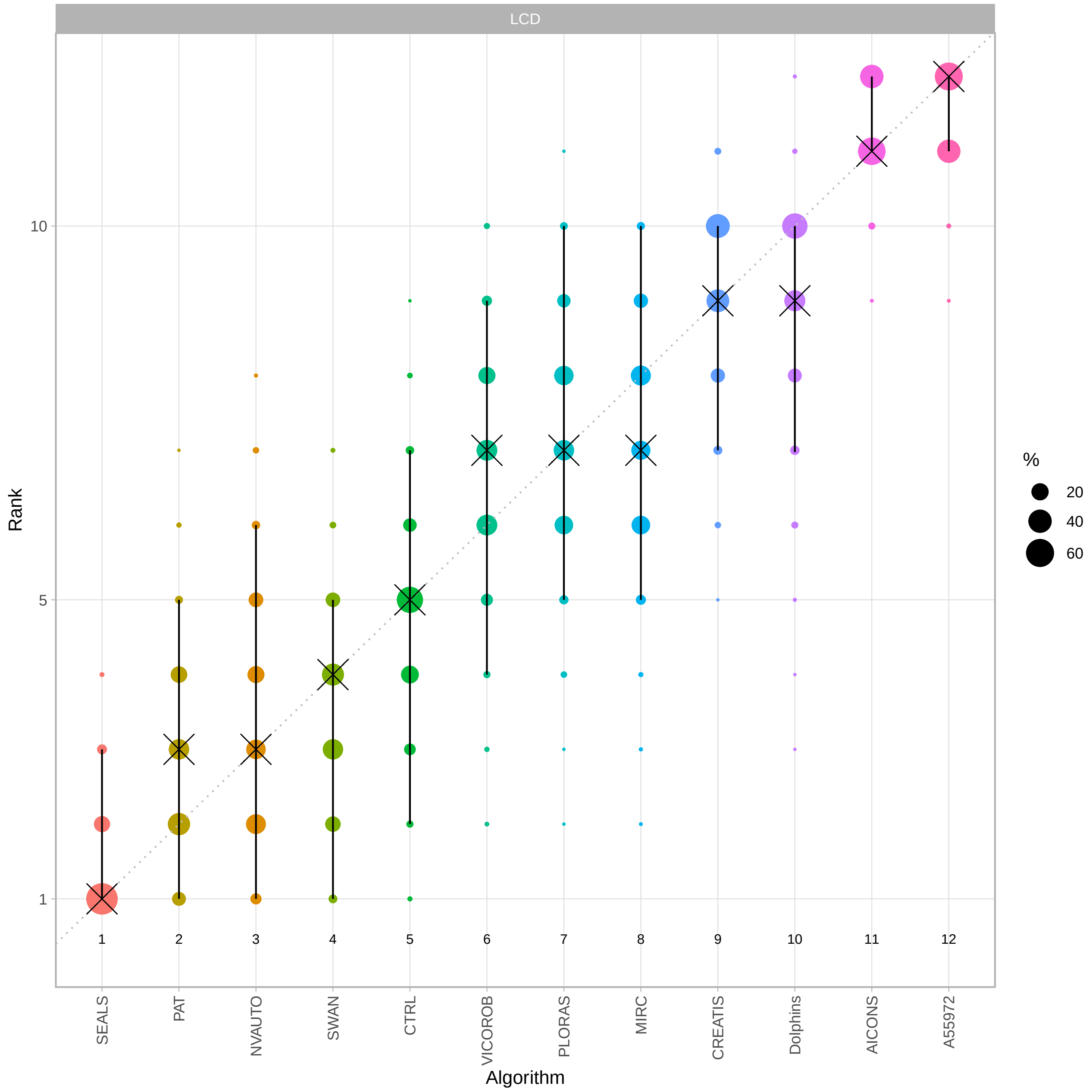

Figure S3.16: LCD Ranking Stability – Blob Plot.

3.2 *Violin plot* for visualizing ranking stability based on bootstrapping

The ranking list based on the full assessment data is pairwise compared with the ranking lists based on the individual bootstrap samples (here $b = 1000$ samples). For each pair of rankings, Kendall's τ correlation is computed. Kendall's τ is a scaled index determining the correlation between the lists. It is computed by evaluating the number of pairwise concordances and discordances between ranking lists and produces values between -1 (for inverted order) and 1 (for identical order). A violin plot, which simultaneously depicts a boxplot and a density plot, is generated from the results.

Summary Kendall's tau:

Task	mean	median	q25	q75
Dice	0.9457770	0.9393939	0.9090909	0.9696970
F1	0.9162829	0.9090909	0.8787879	0.9393939
LCD	0.8443604	0.8484848	0.8181818	0.8787879
AVD	0.7967418	0.8181818	0.7272727	0.8787879

Table S3.6: Kendall's Tau Summary for Ranking Stability.

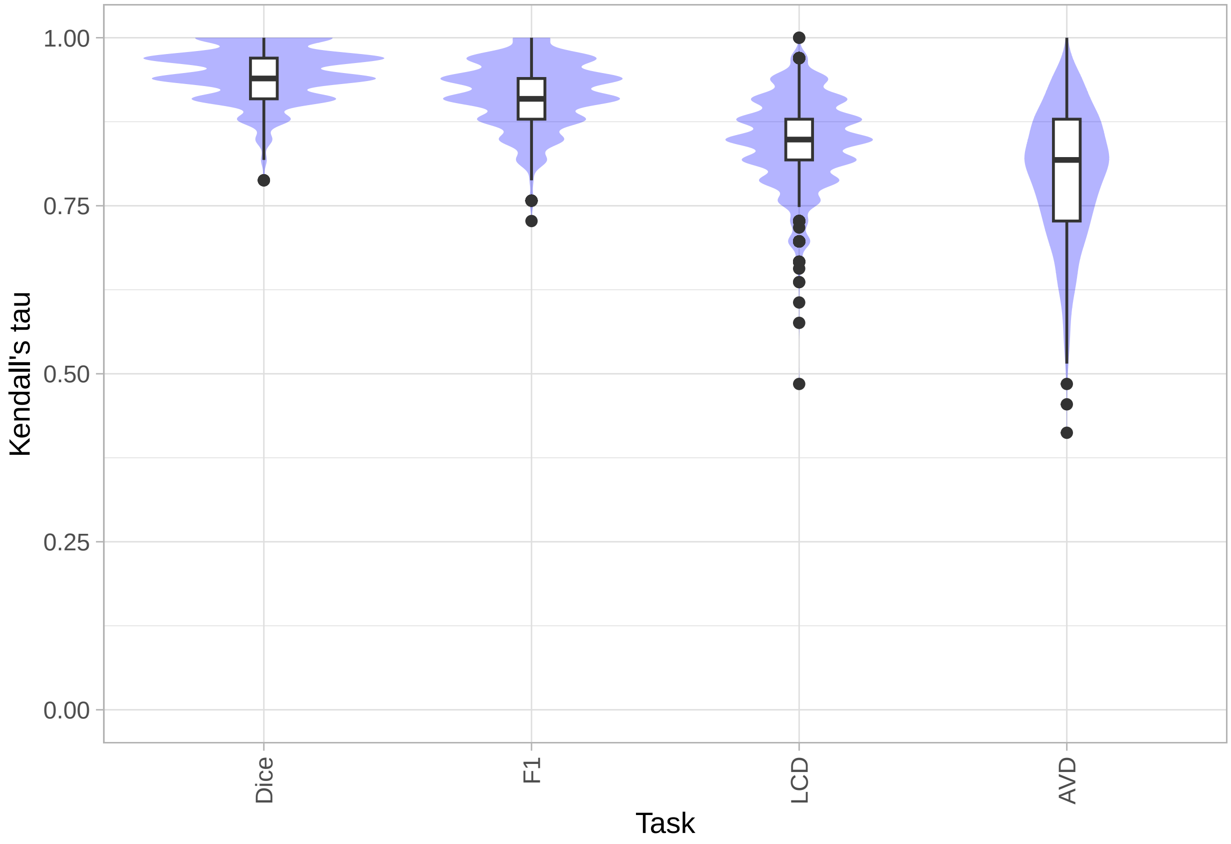

Figure S3.17: Kendall's Tau Violin Plot for All Metrics.

3.3 *Significance maps* for visualizing ranking stability based on statistical significance

Significance maps depict incidence matrices of pairwise significant test results for the one-sided Wilcoxon signed rank test at a 5% significance level with adjustment for multiple testing according to Holm. Yellow shading indicates that metric values from the algorithm on the x-axis were significantly superior to those from the algorithm on the y-axis, blue color indicates no significant difference.

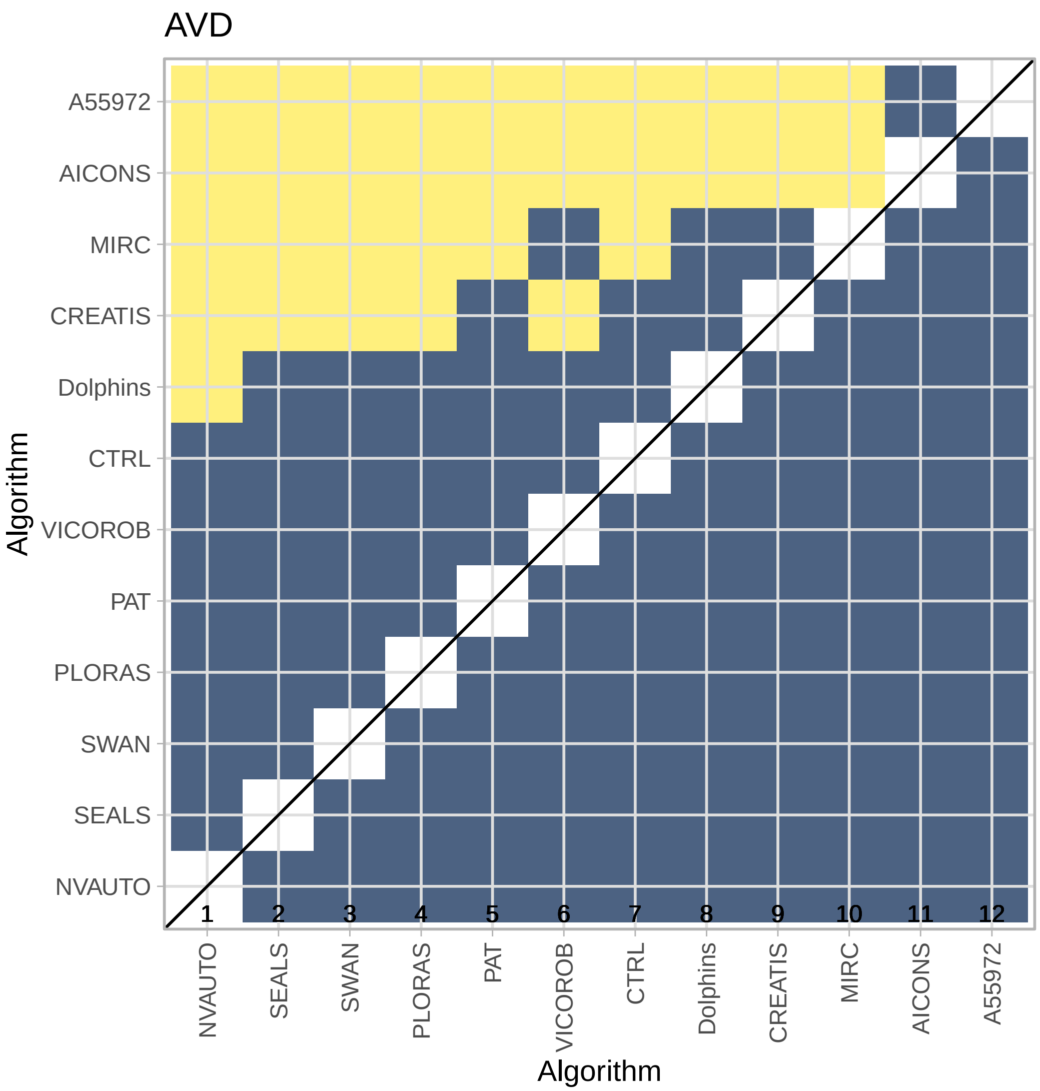

Figure S3.18: AVD Significance Map.

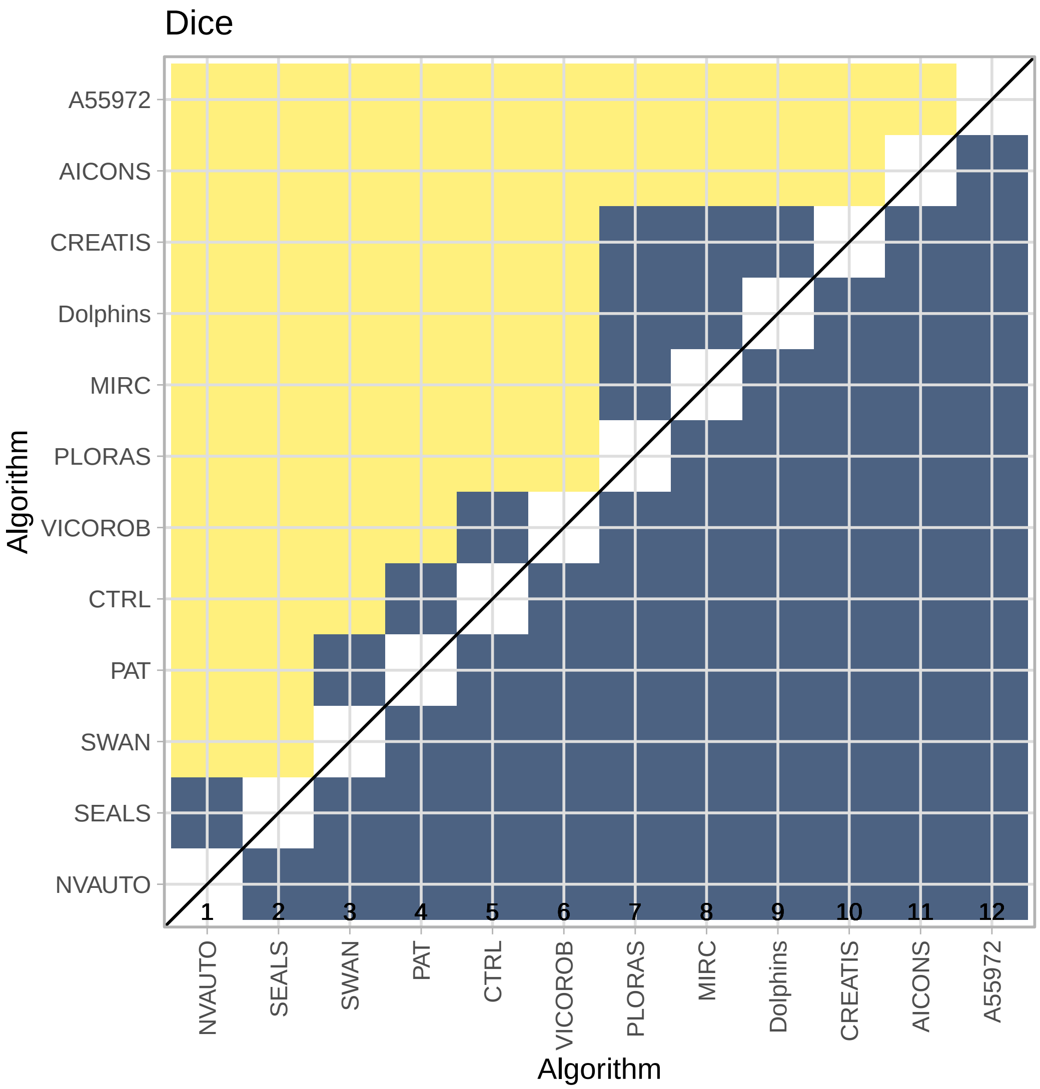

Figure S3.19: Dice Significance Map.

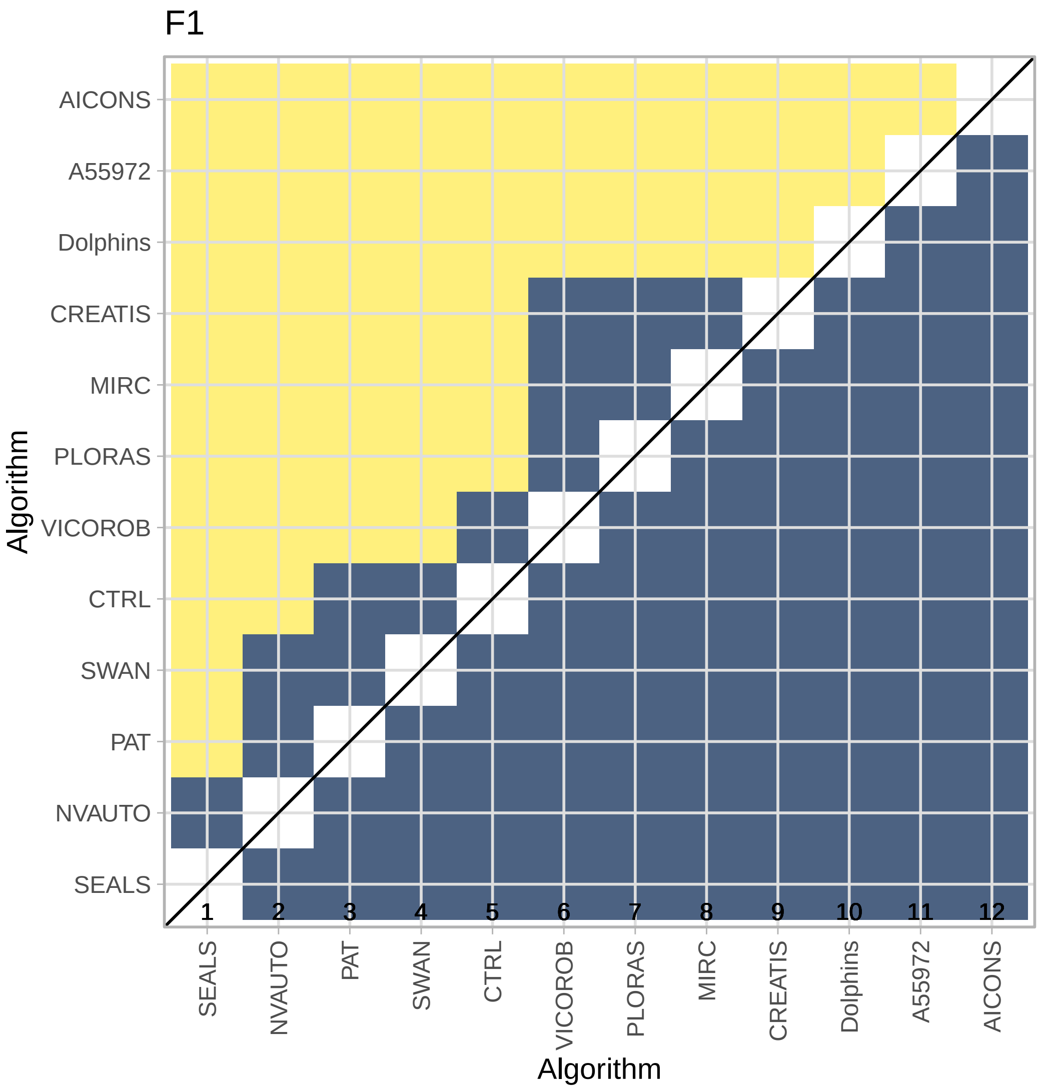

Figure S3.20: F1 Significance Map.

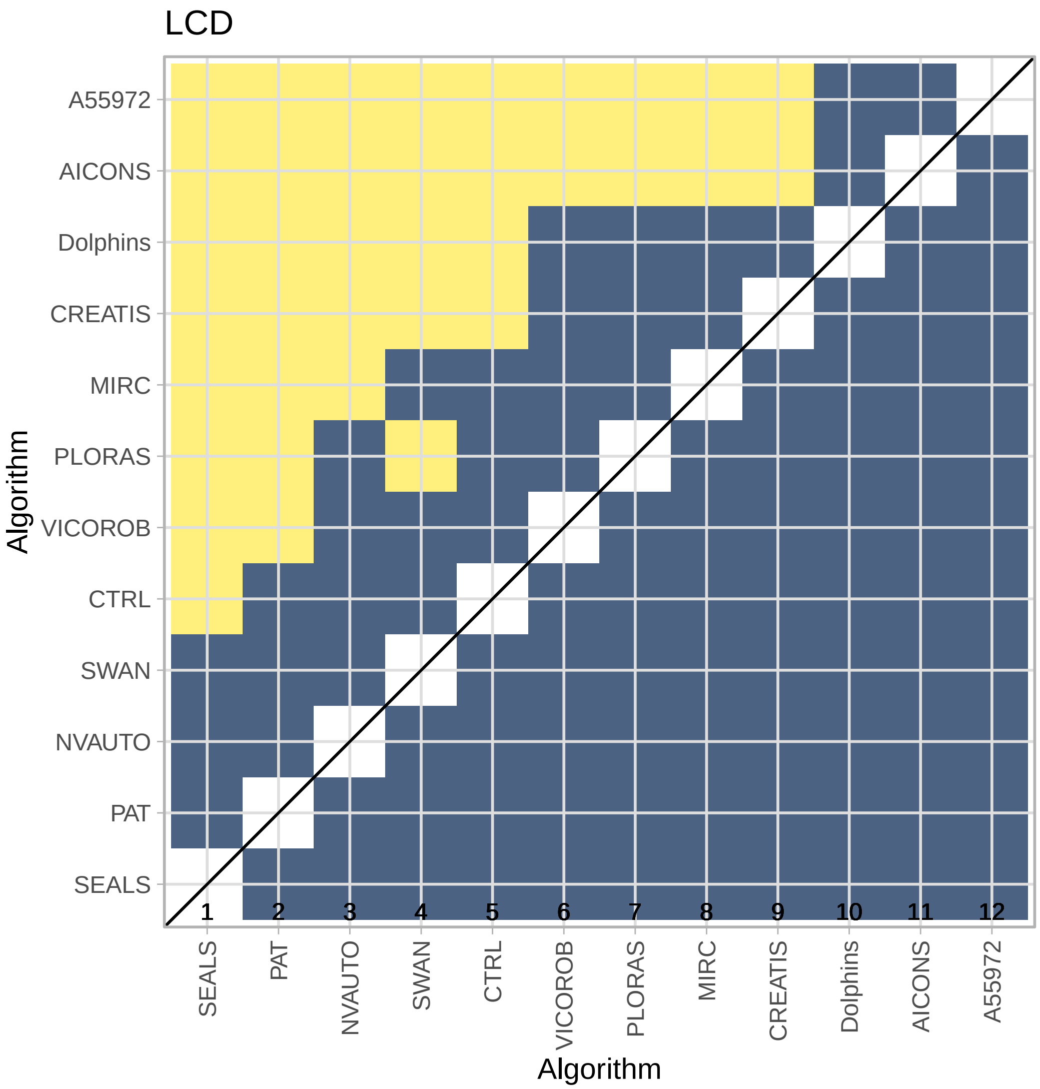

Figure S3.21: LCD Significance Map.

3.4 Ranking robustness to ranking methods

Line plots for visualizing ranking robustness across different ranking methods. Each algorithm is represented by one colored line. For each ranking method encoded on the x-axis, the height of the line represents the corresponding rank. Horizontal lines indicate identical ranks for all methods.

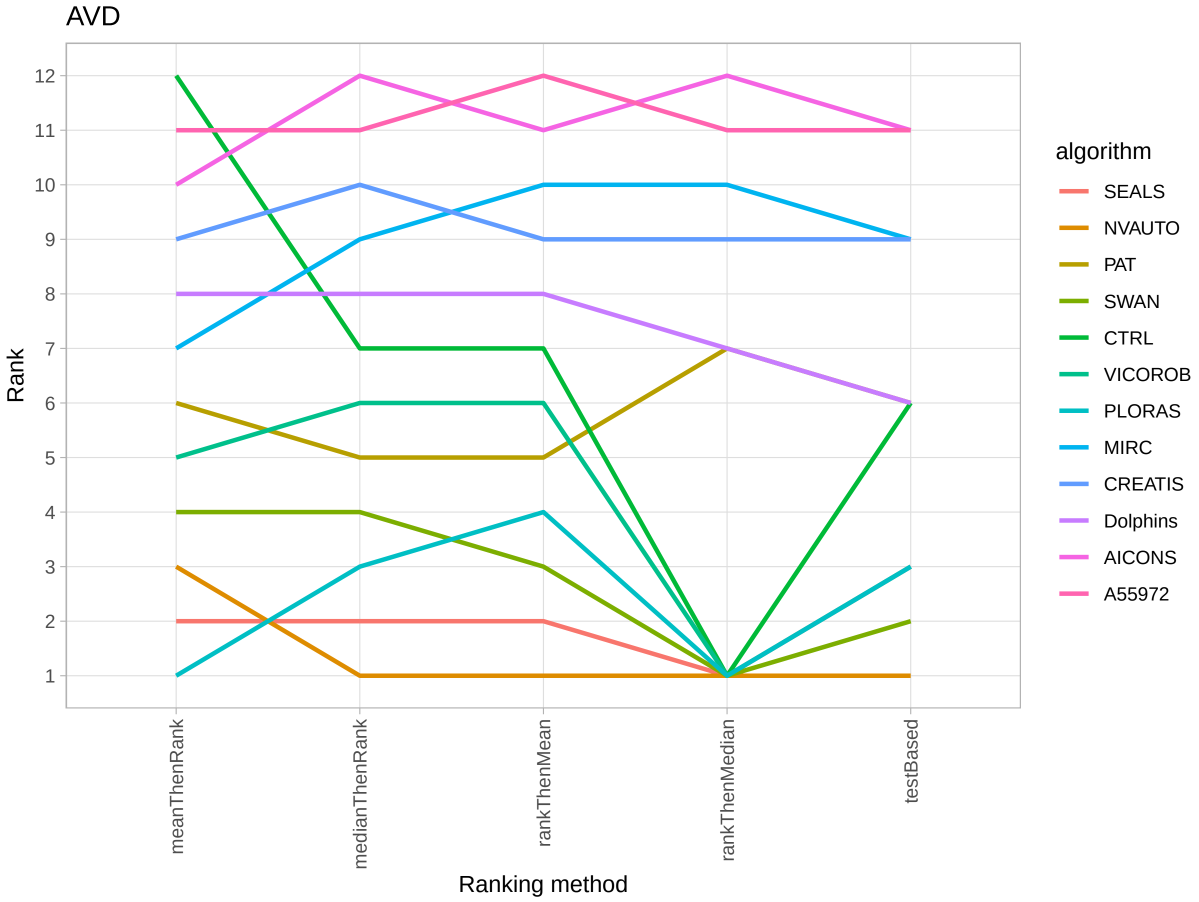

Figure S3.22: AVD Ranking Robustness Across Methods.

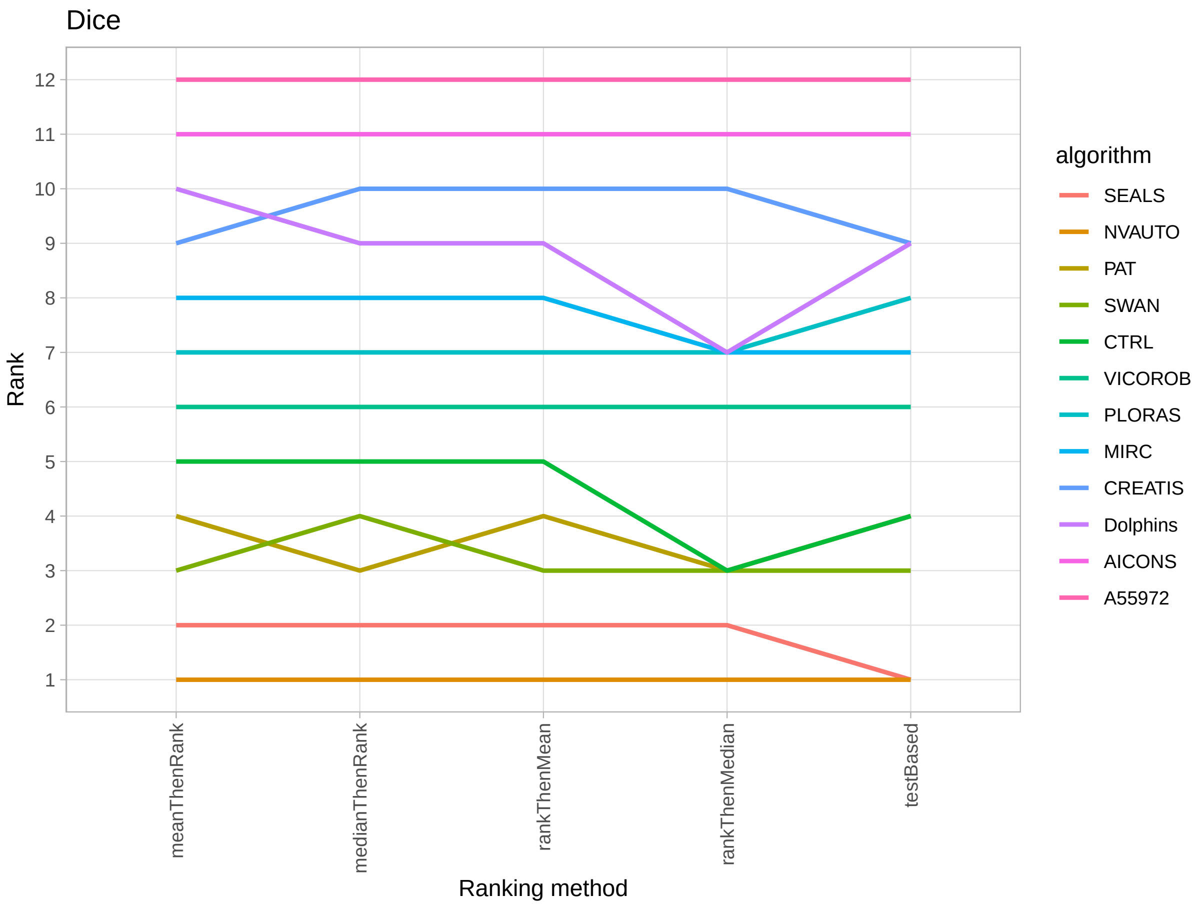

Figure S3.23: Dice Ranking Robustness Across Methods.

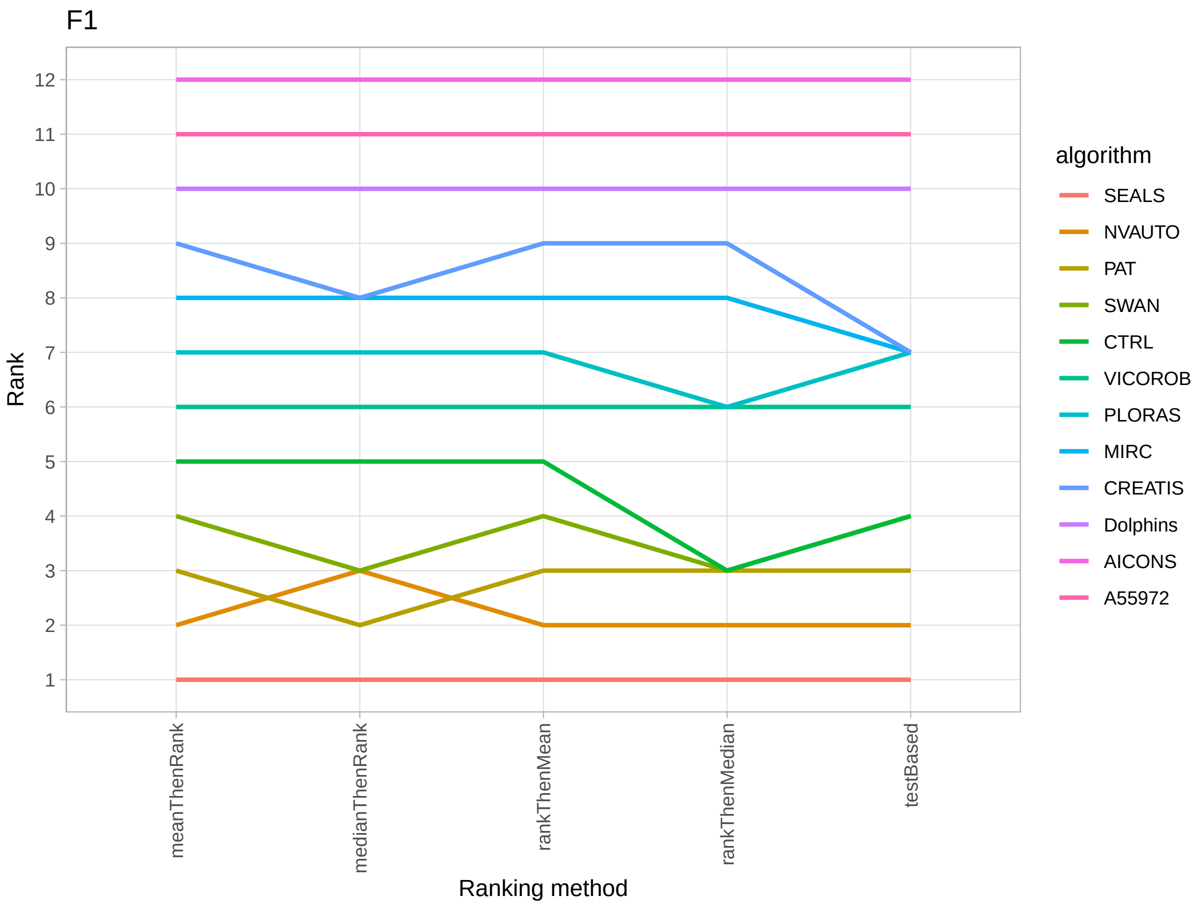

Figure S3.24: F1 Ranking Robustness Across Methods.

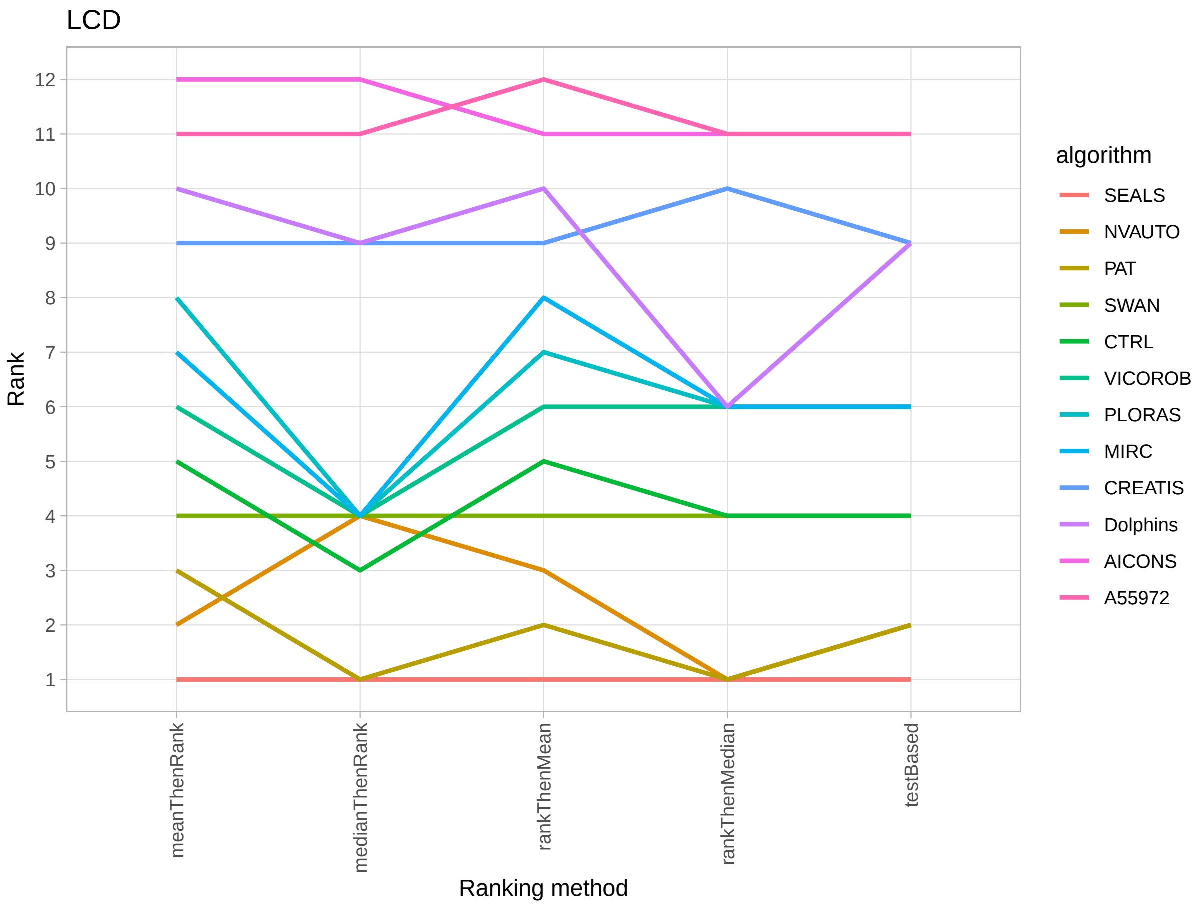

Figure S3.25: LCD Ranking Robustness Across Methods.

4 Visualization of cross-task insights

The algorithms are ordered according to consensus ranking.

4.1 Characterization of algorithms

4.1.1 Ranking stability: Variability of achieved rankings across tasks

Algorithms are color-coded, and the area of each blob at position $(A_i, \text{rank } j)$ is proportional to the relative frequency A_i achieved rank j across multiple tasks. The median rank for each algorithm is indicated by a black cross. This way, the distribution of ranks across tasks can be intuitively visualized.

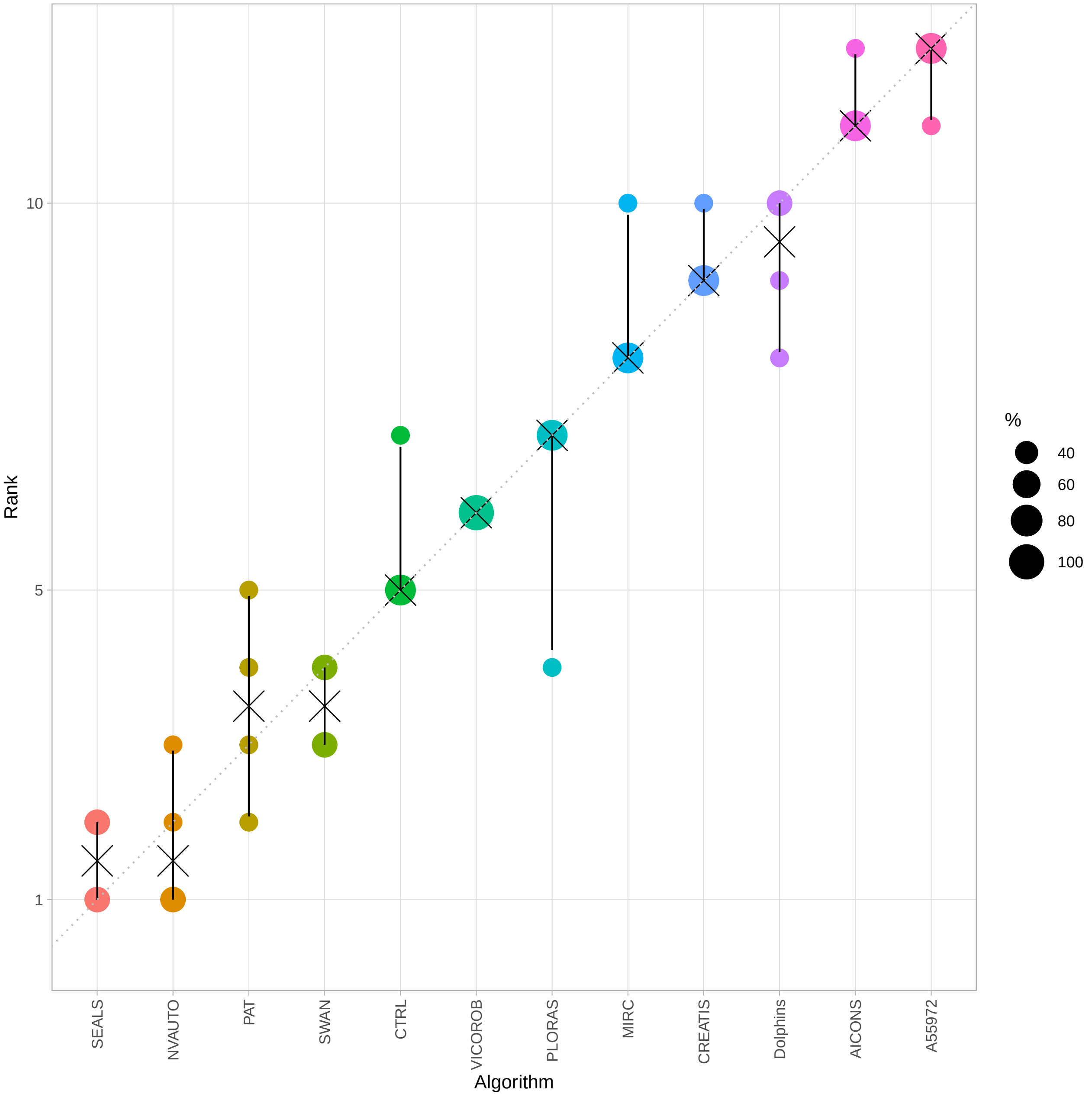

Figure S3.26: Cross-Task Ranking Stability – Blob Plot.

4.1.2 Ranking stability: Ranking variability via bootstrap approach

A blob plot of bootstrap results over the different tasks separated by algorithm allows another perspective on the assessment data. This gives deeper insights into the characteristics of tasks and the ranking uncertainty of the algorithms in each task.

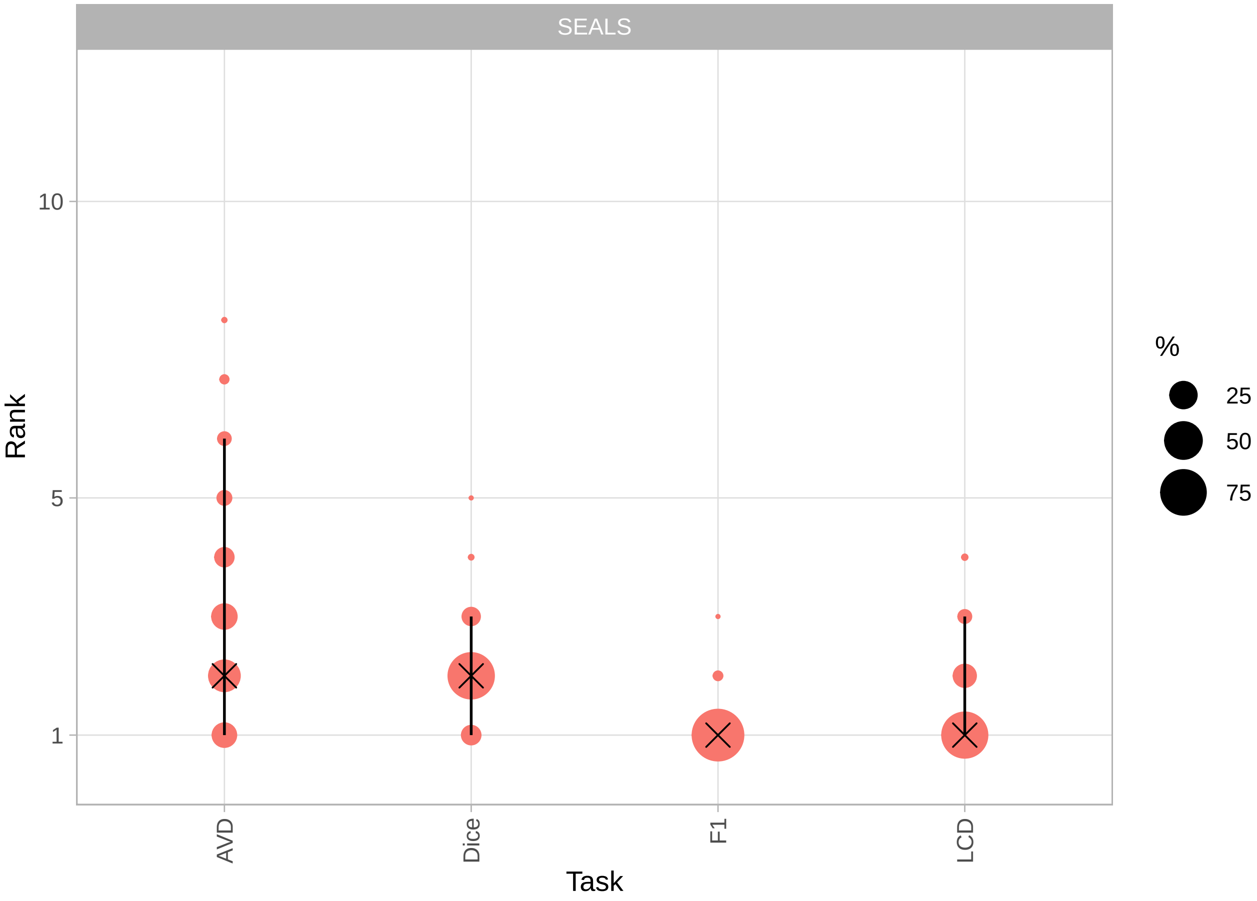

Figure S3.27: Bootstrap Ranking Stability – SEALS.

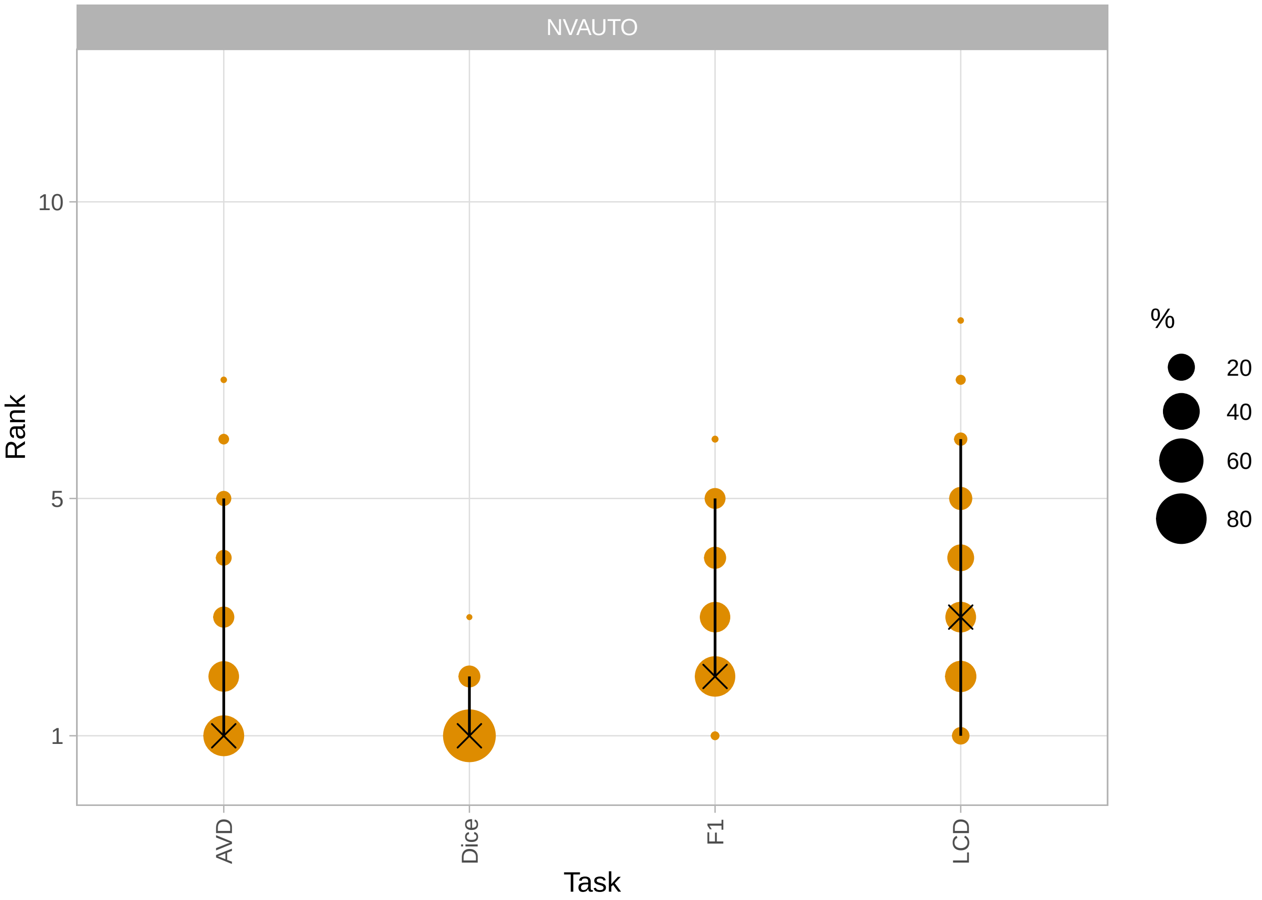

Figure S3.28: Bootstrap Ranking Stability – NVAUTO.

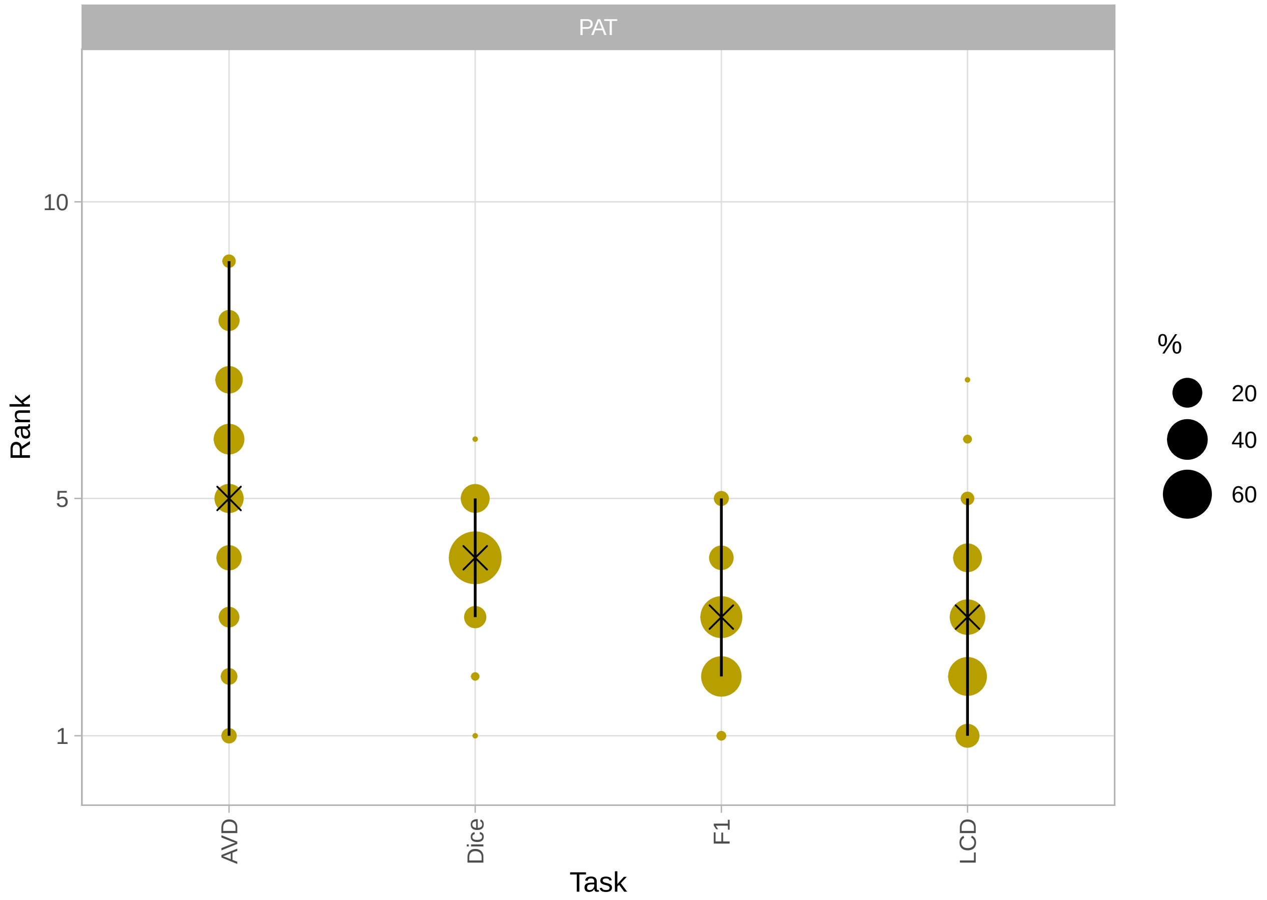

Figure S3.29: Bootstrap Ranking Stability – PAT.

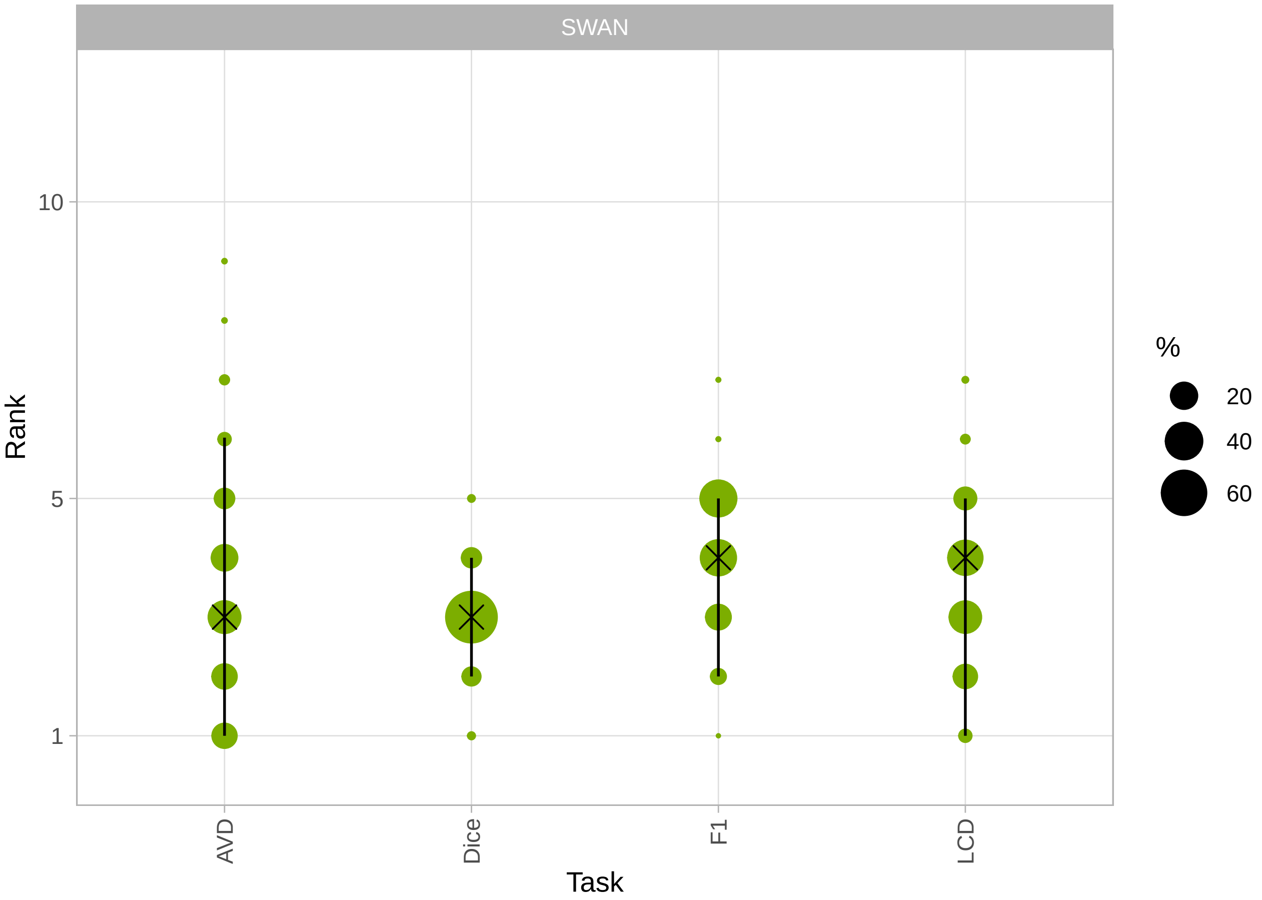

Figure S3.30: Bootstrap Ranking Stability – SWAN.

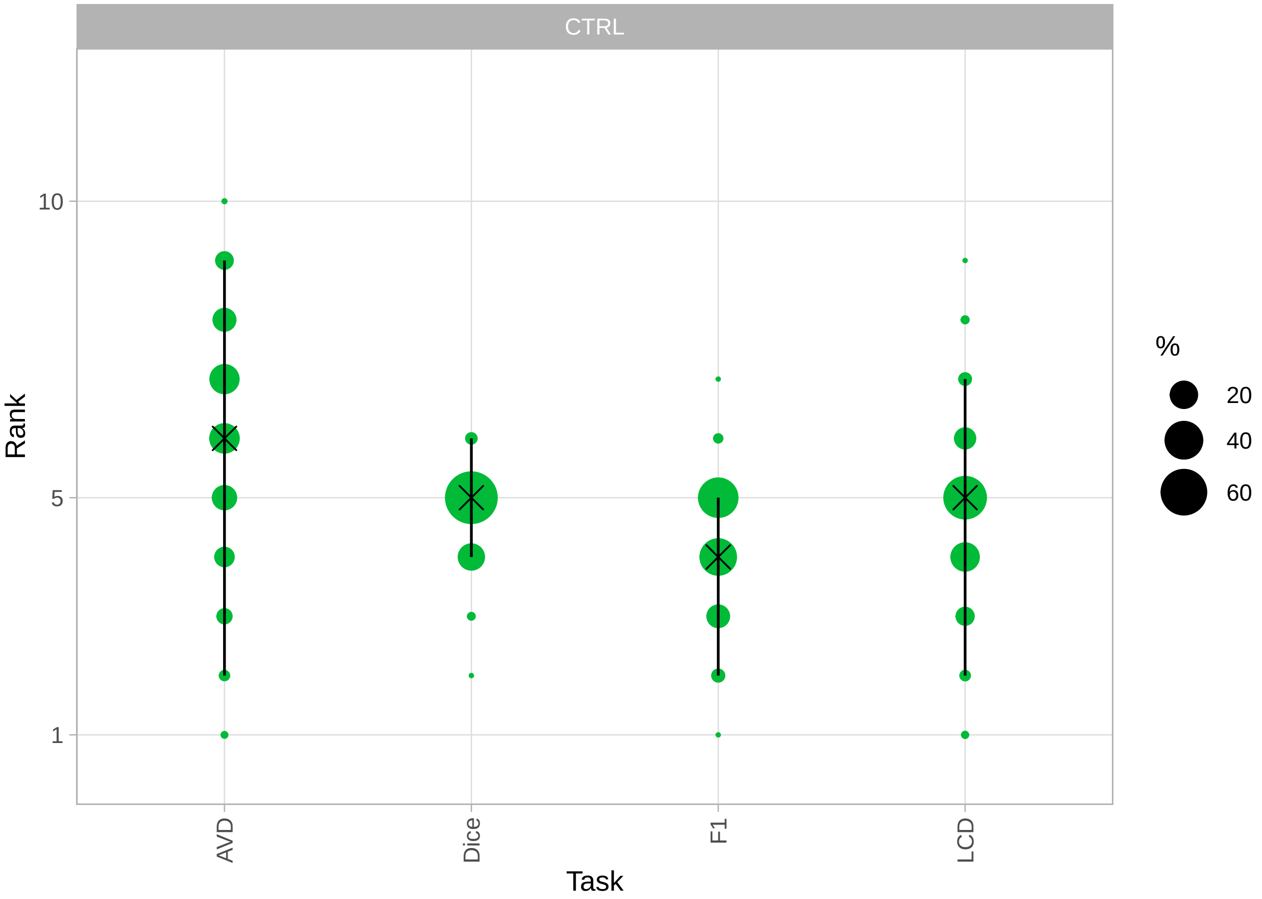

Figure S3.31: Bootstrap Ranking Stability – CTRL.

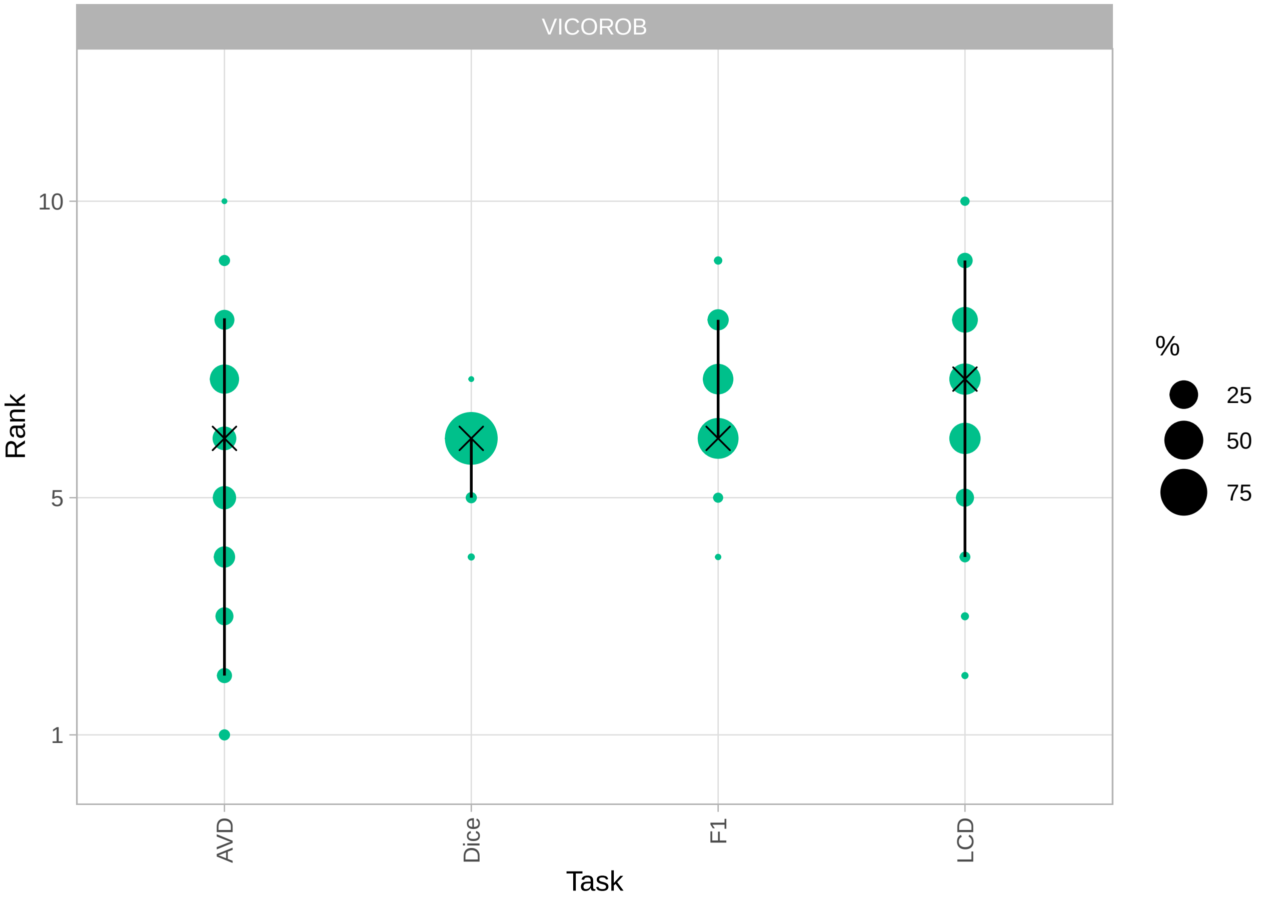

Figure S3.32: Bootstrap Ranking Stability – VICOROB.

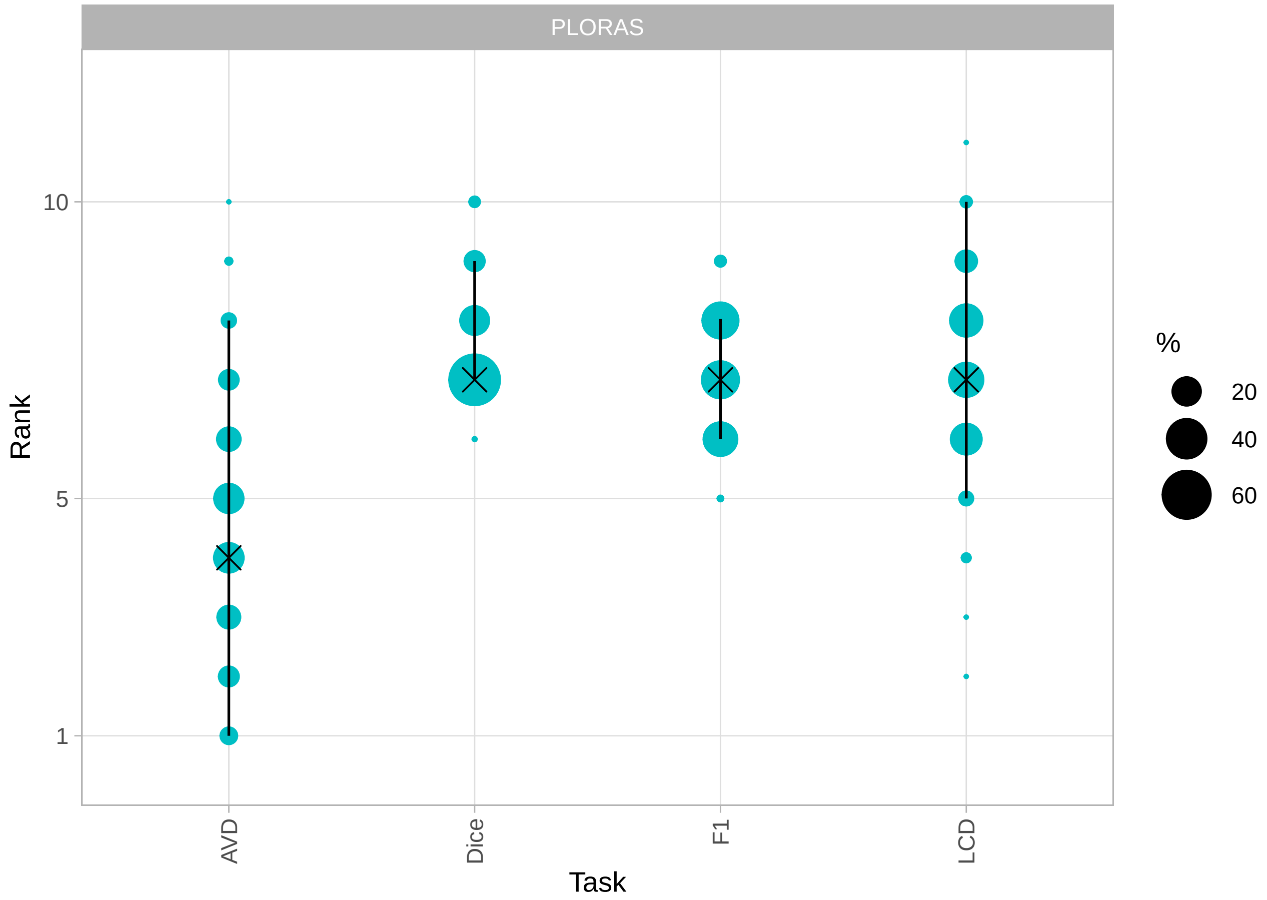

Figure S3.33: Bootstrap Ranking Stability – PLORAS.

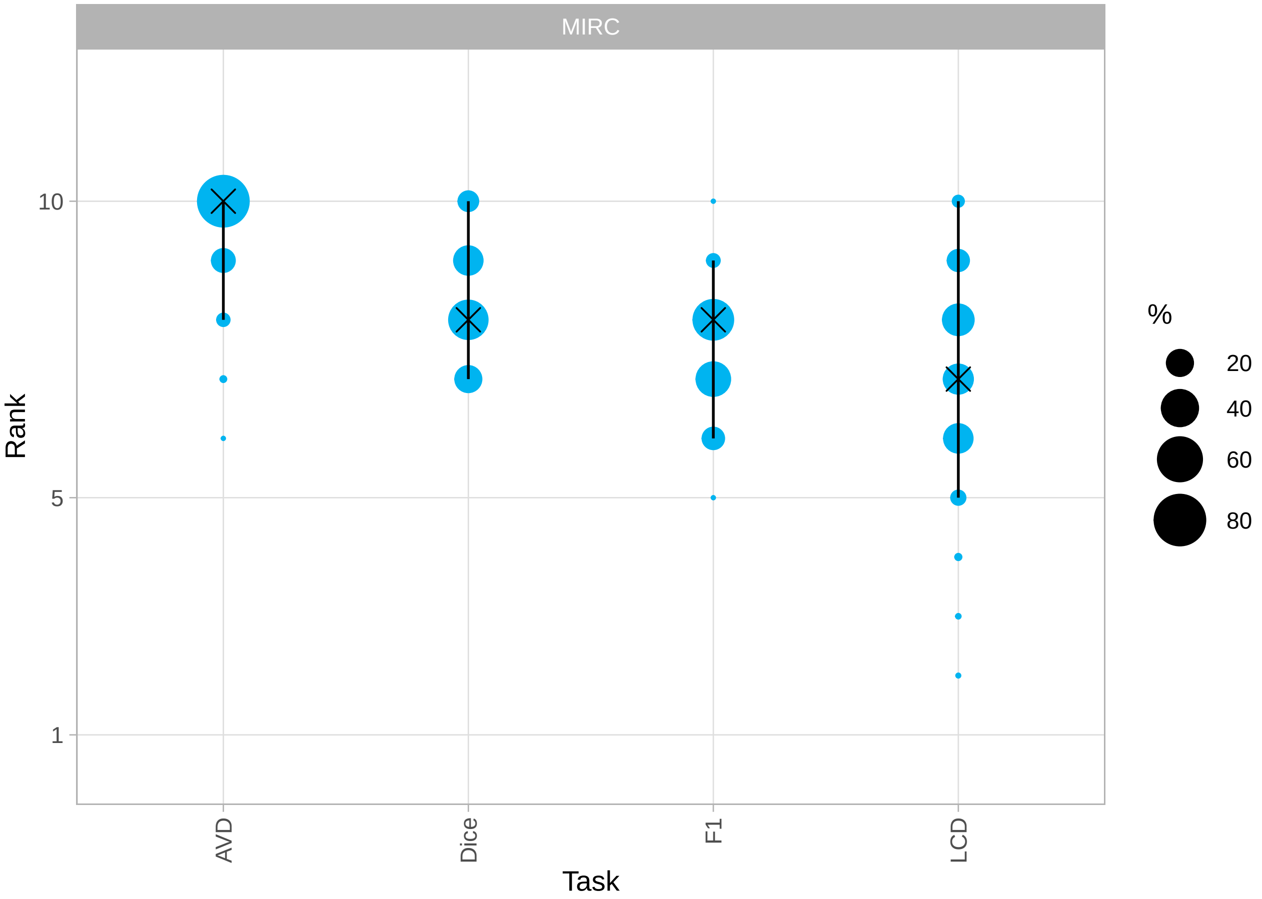

Figure S3.34: Bootstrap Ranking Stability – MIRC.

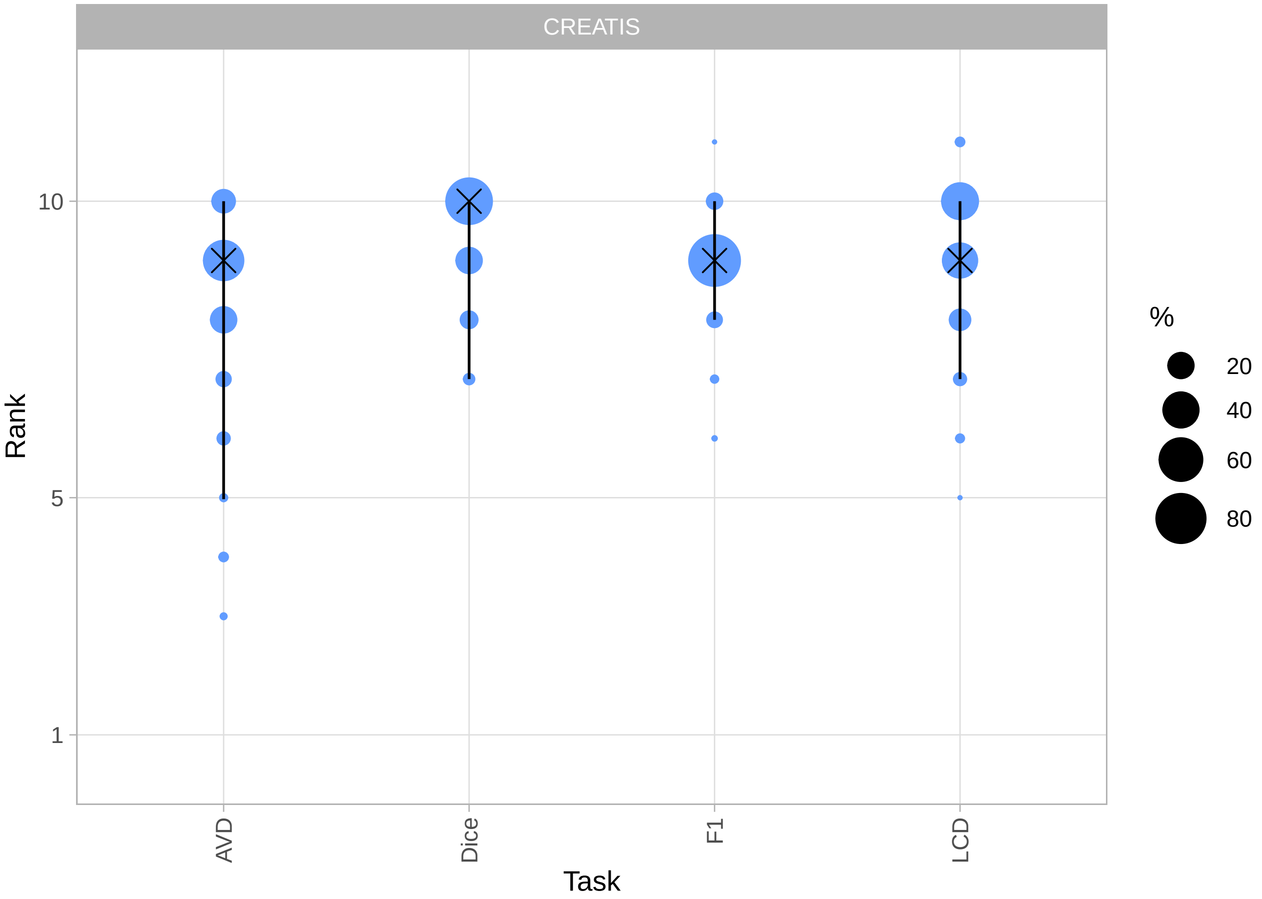

Figure S3.35: Bootstrap Ranking Stability – CREATIS.

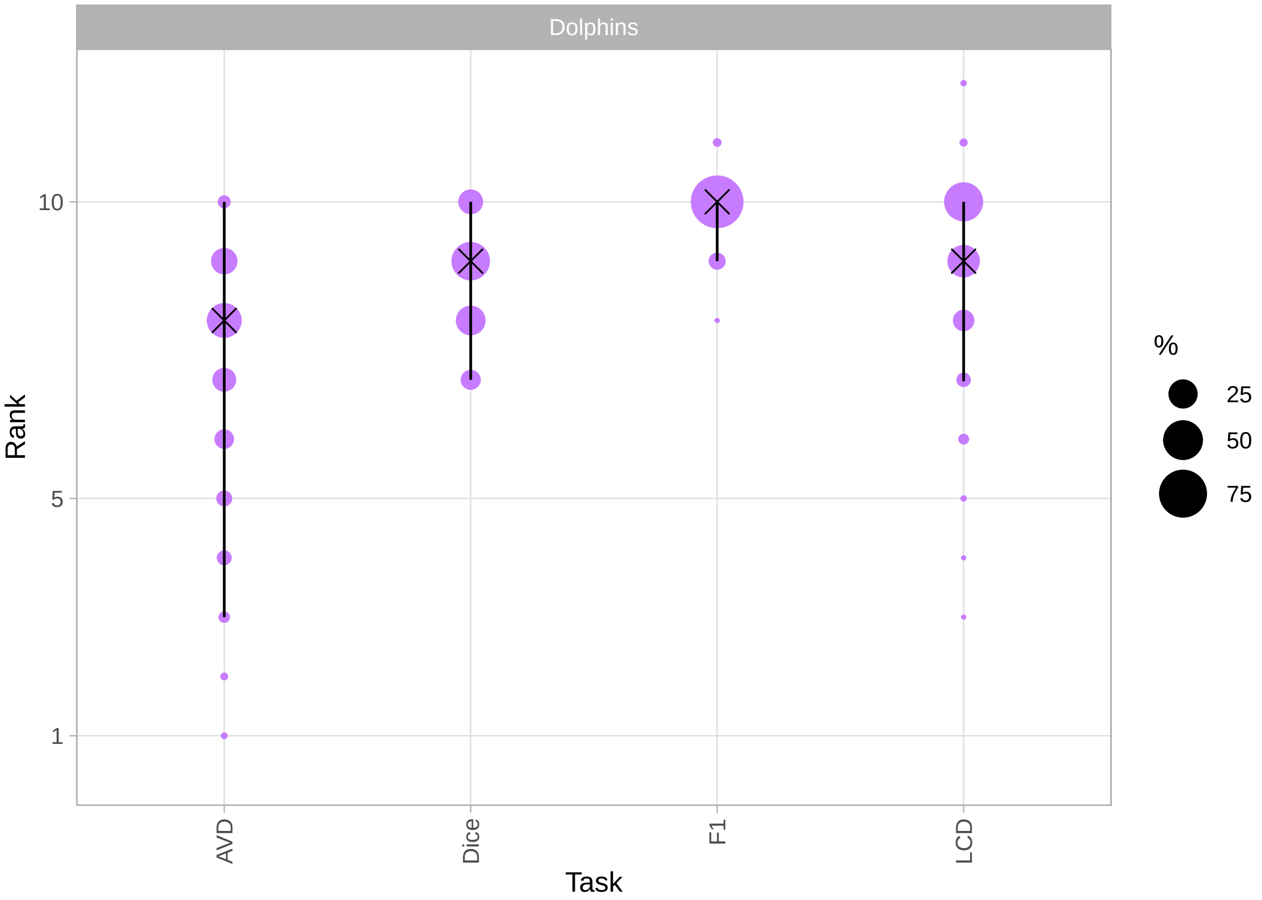

Figure S3.36: Bootstrap Ranking Stability – Dolphins.

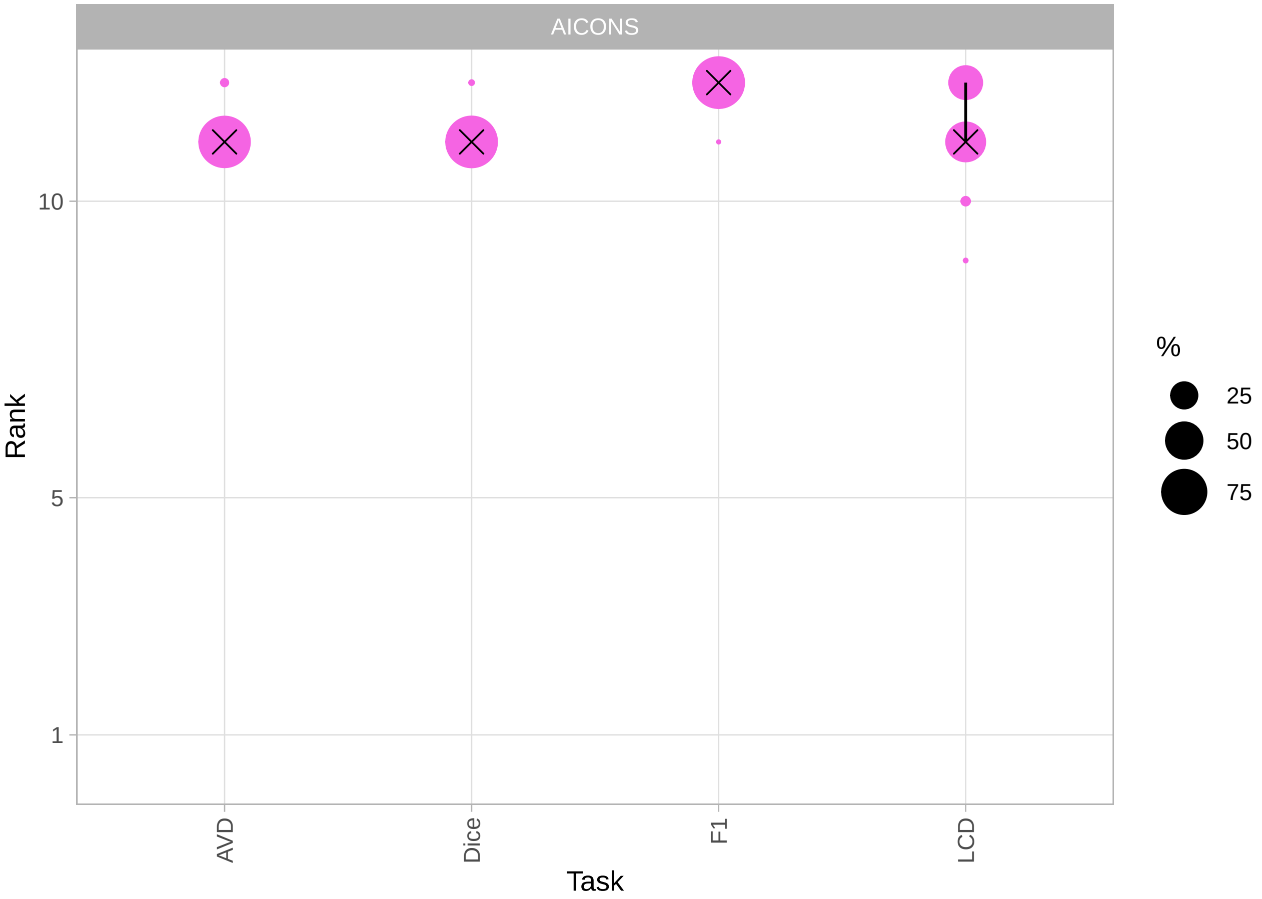

Figure S3.37: Bootstrap Ranking Stability – AICONS.

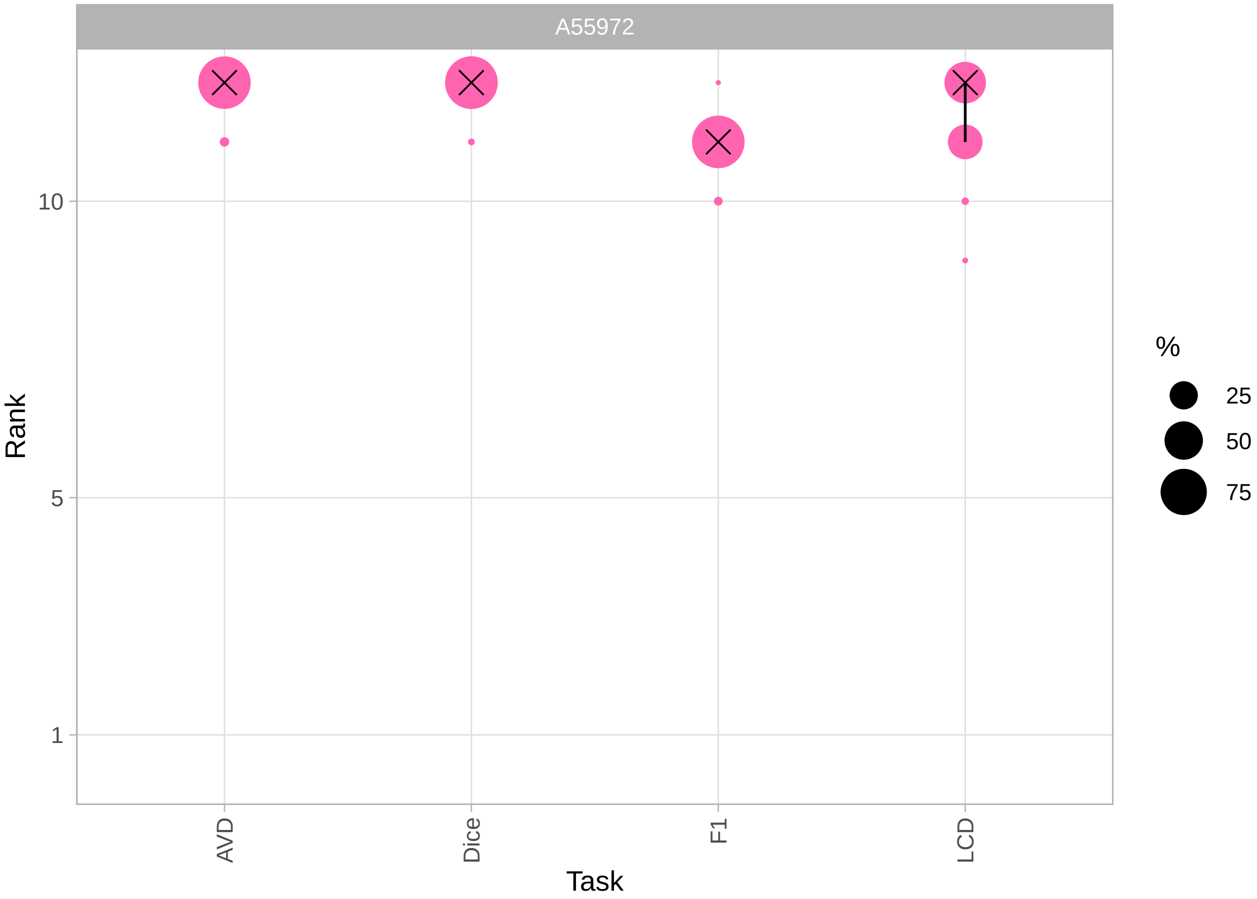

Figure S3.38: Bootstrap Ranking Stability – A55972.

An alternative representation is provided by a stacked frequency plot of the observed ranks, separated by algorithm. Observed ranks across bootstrap samples are displayed with coloring according to the task. For algorithms that achieve the same rank in different tasks for the full assessment data set, vertical lines are on top of each other. Vertical lines allow to compare the achieved rank of each algorithm over different tasks.

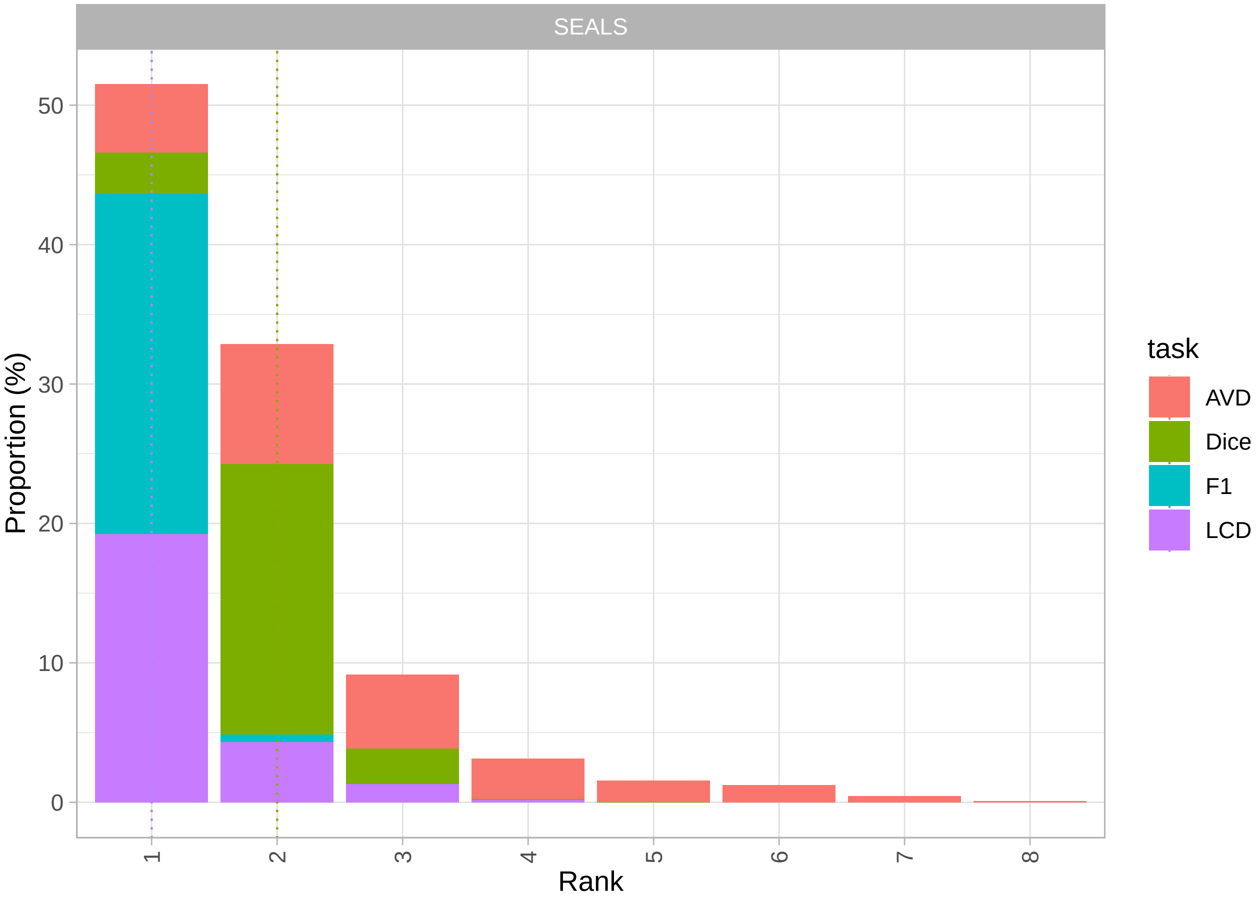

Figure S3.39: Stacked Rank Frequencies – SEALS.

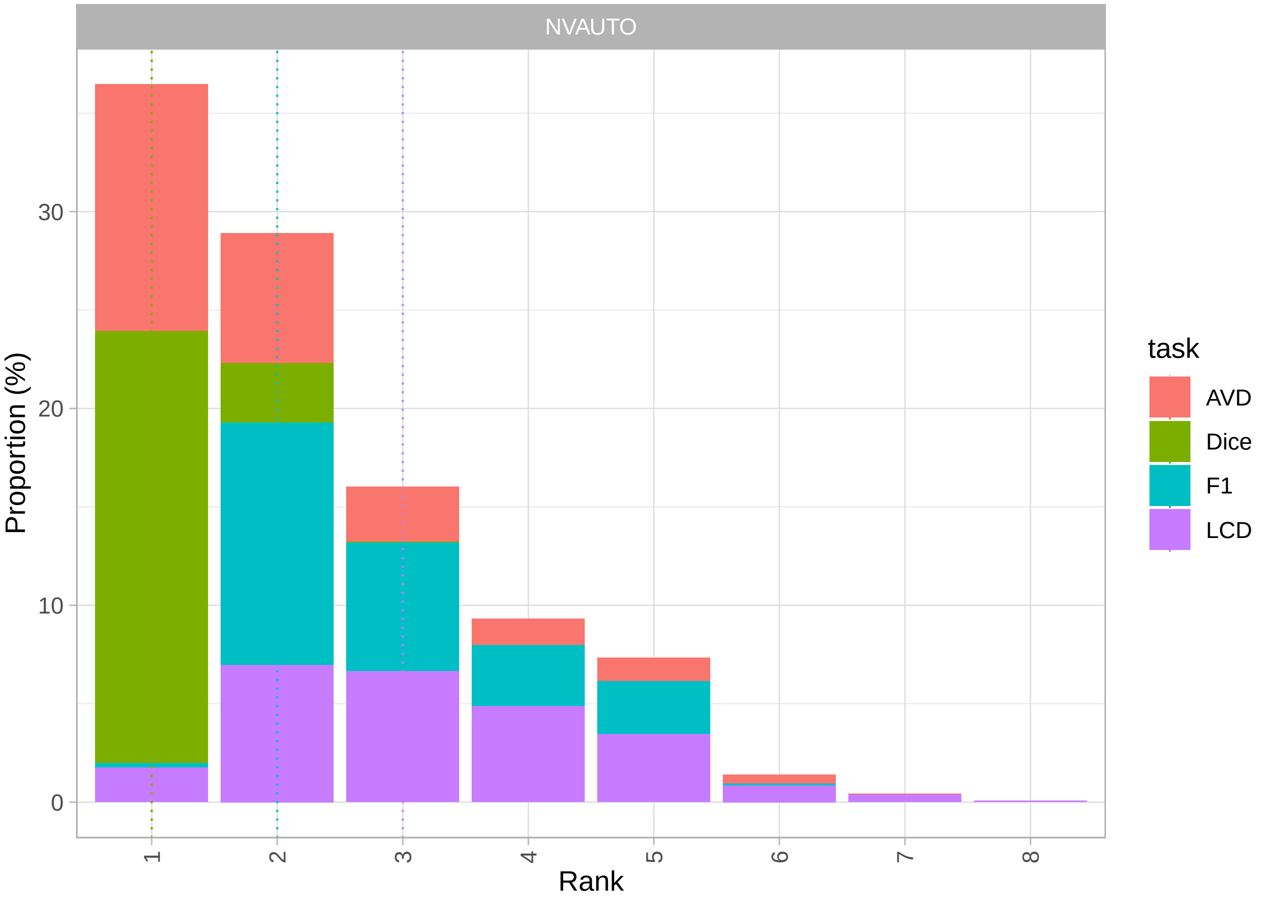

Figure S3.40: Stacked Rank Frequencies – NVAUTO.

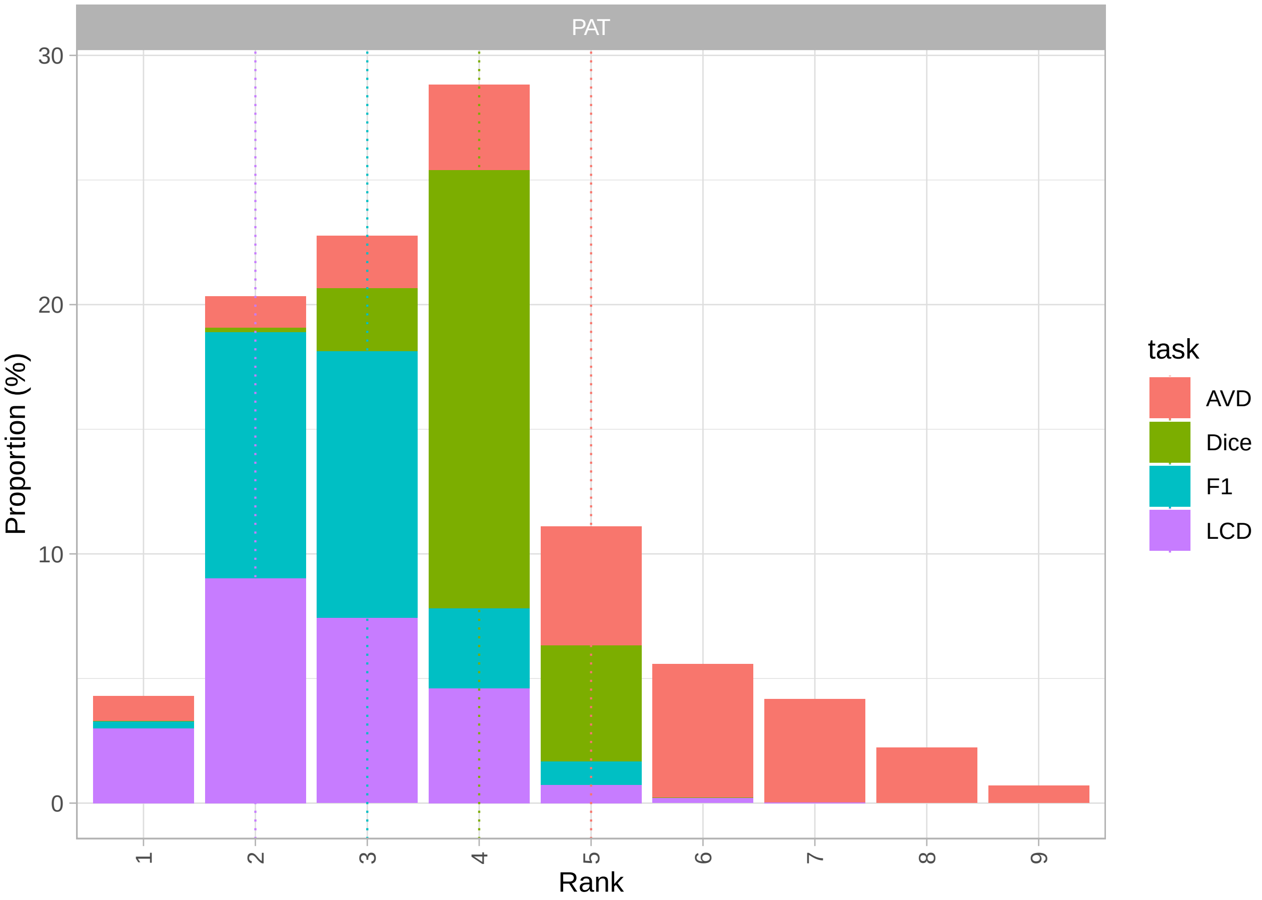

Figure S3.41: Stacked Rank Frequencies – PAT.

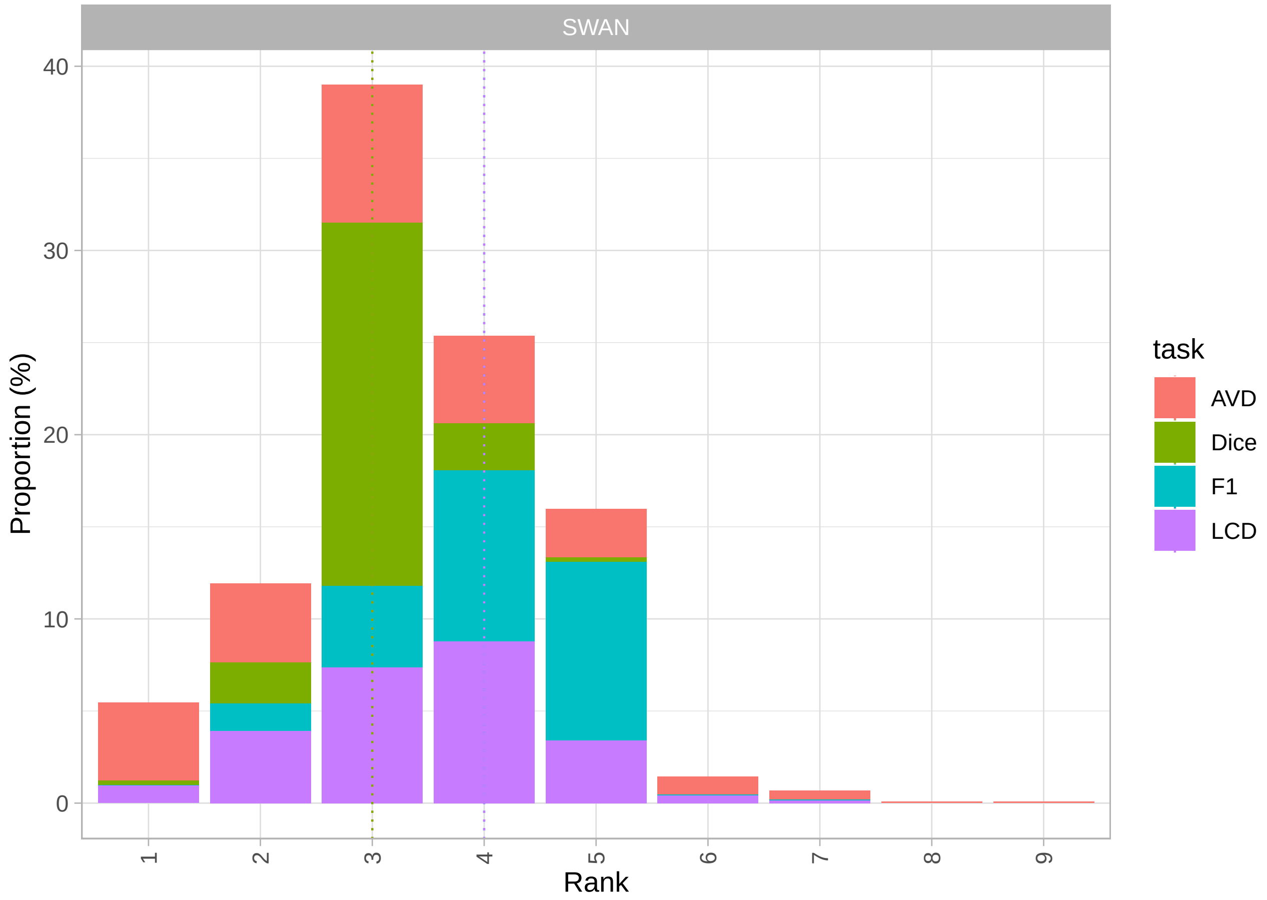

Figure S3.42: Stacked Rank Frequencies – SWAN.

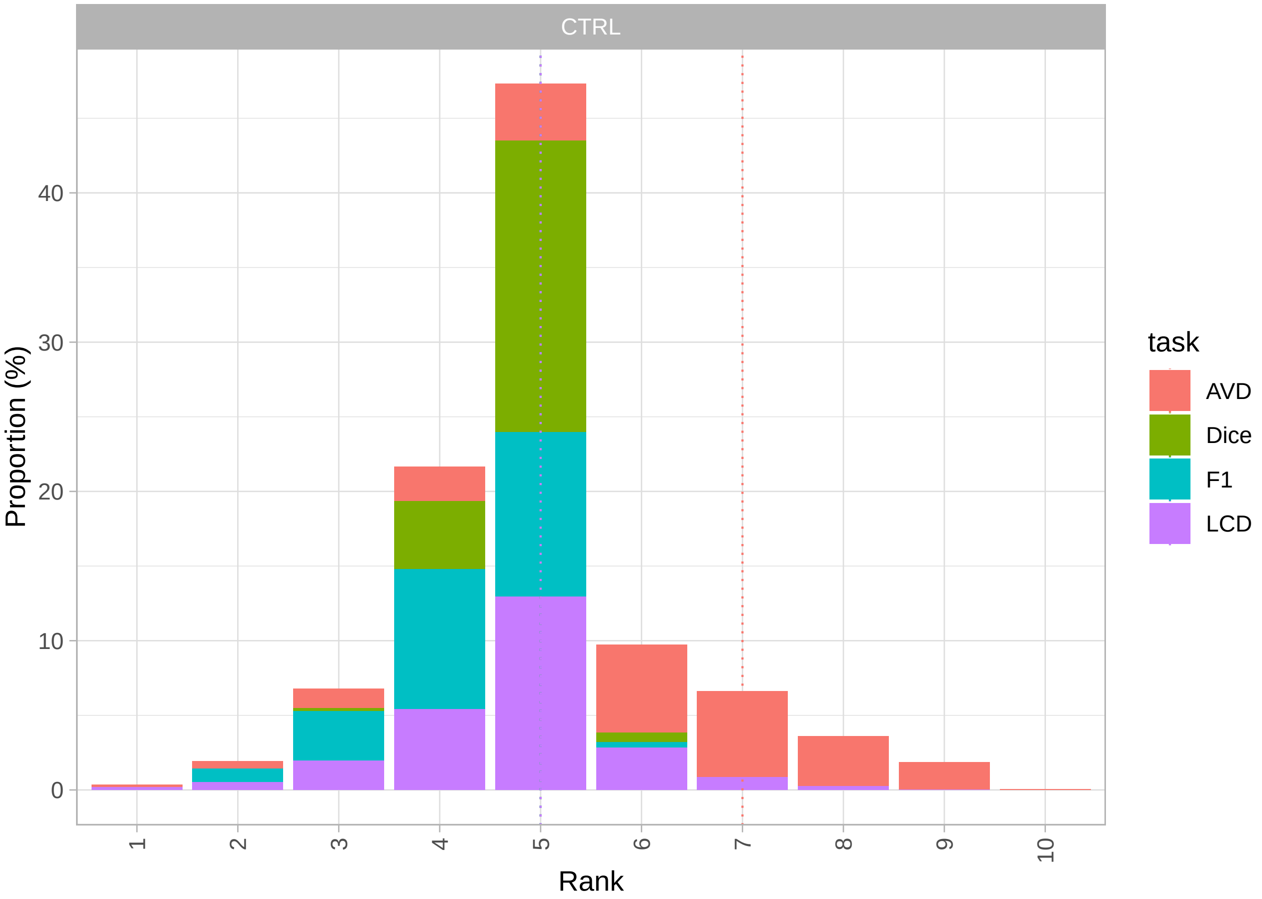

Figure S3.43: Stacked Rank Frequencies – CTRL.

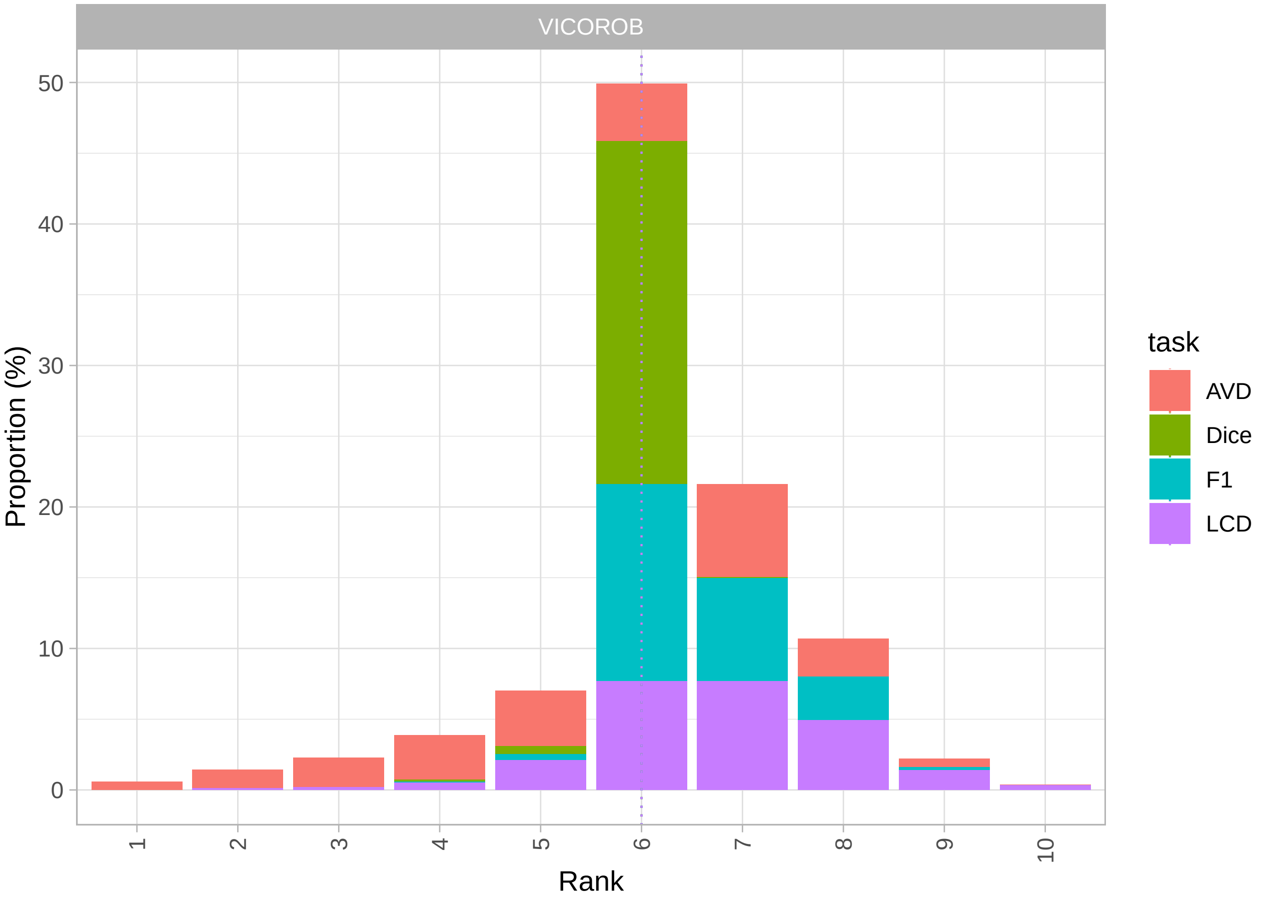

Figure S3.44: Stacked Rank Frequencies – VICOROB.

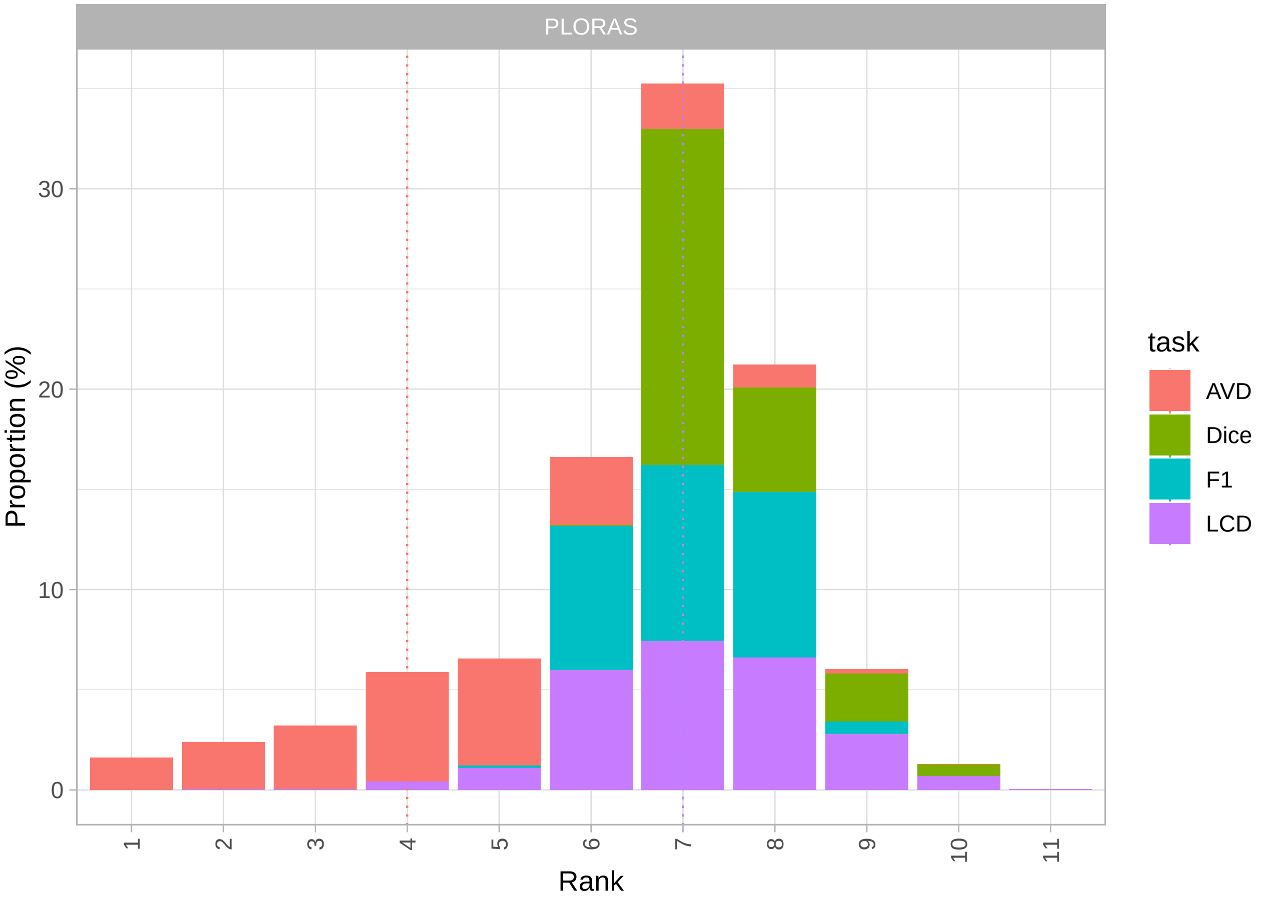

Figure S3.45: Stacked Rank Frequencies – PLORAS.

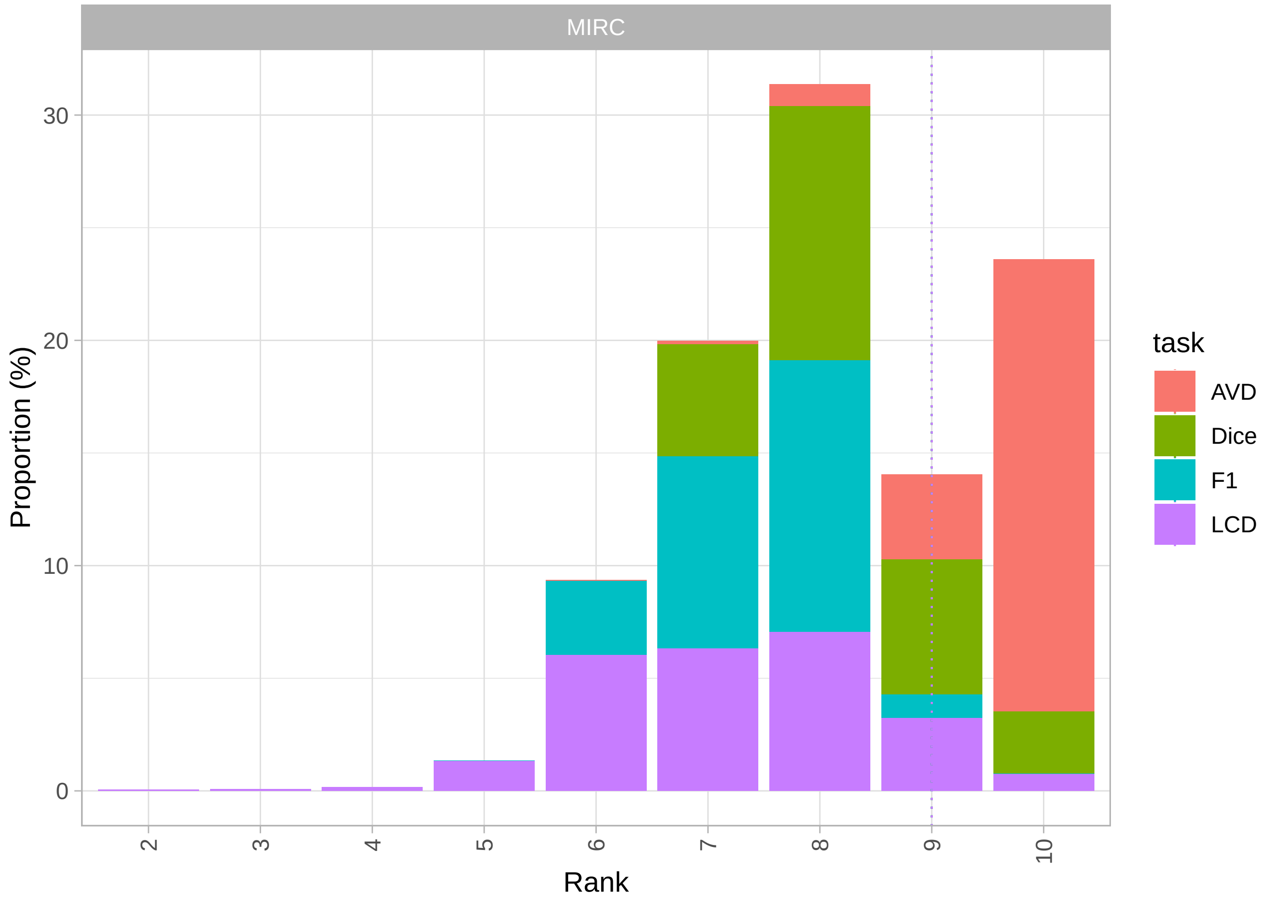

Figure S3.46: Stacked Rank Frequencies – MIRC.

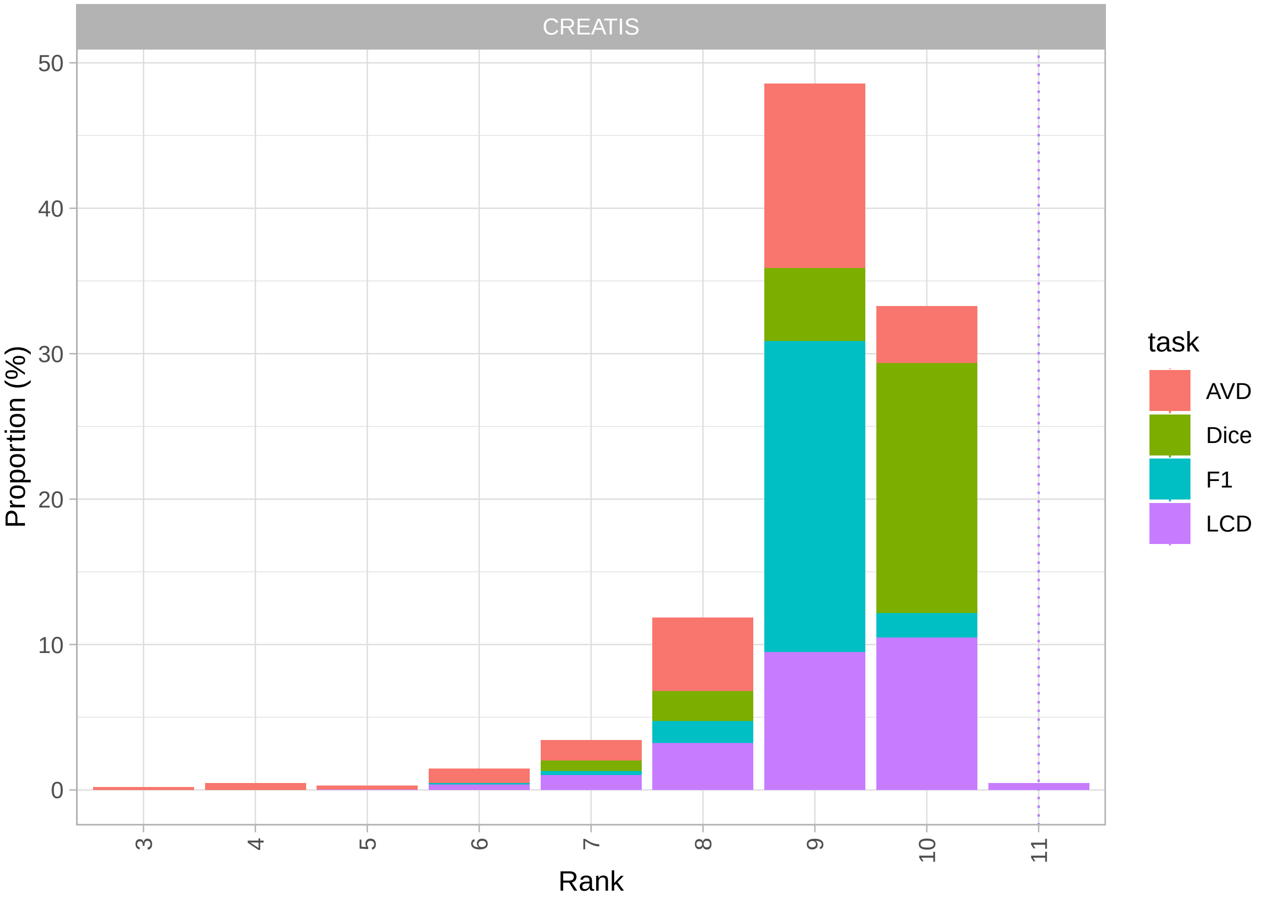

Figure S3.47: Stacked Rank Frequencies – CREATIS.

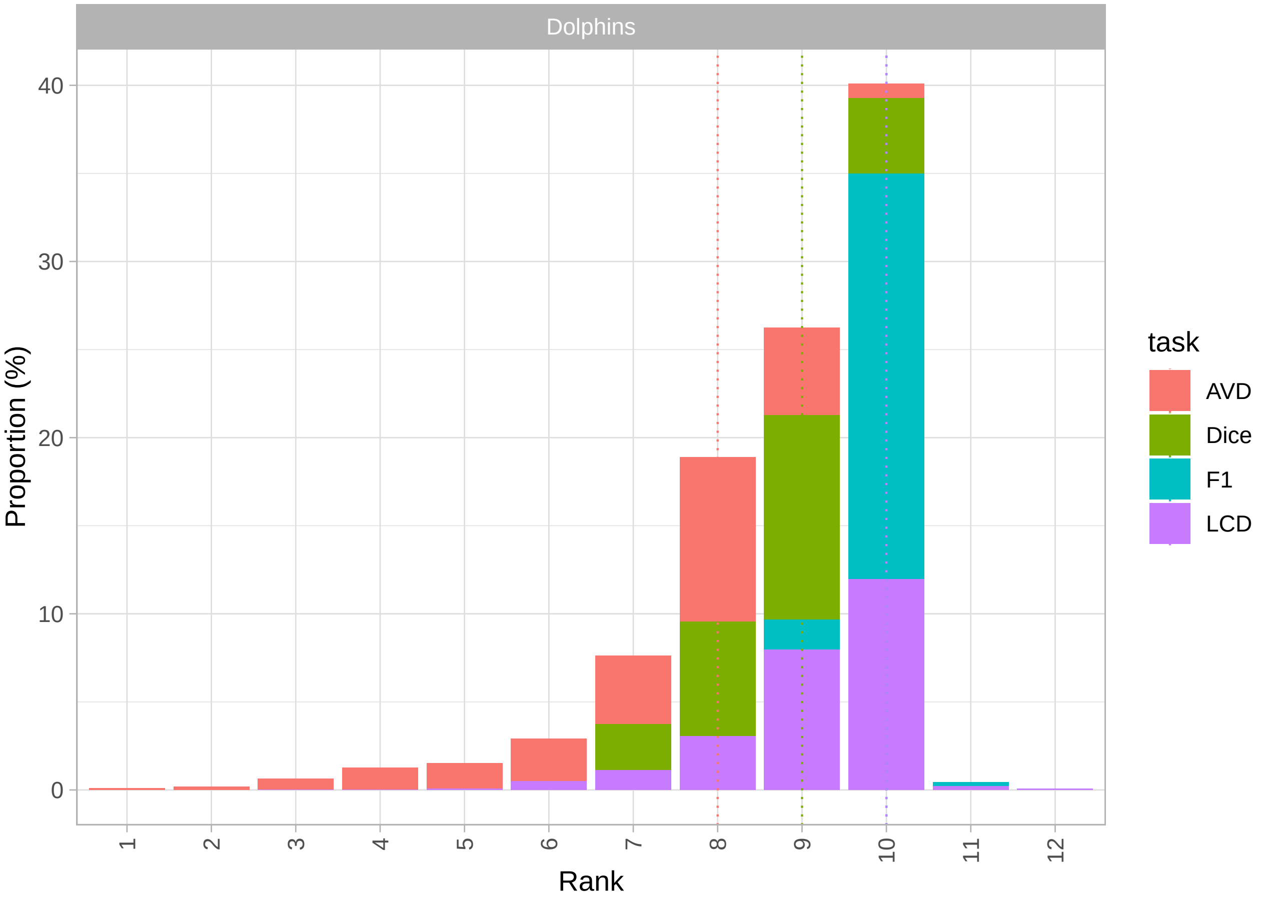

Figure S3.48: Stacked Rank Frequencies – Dolphins.

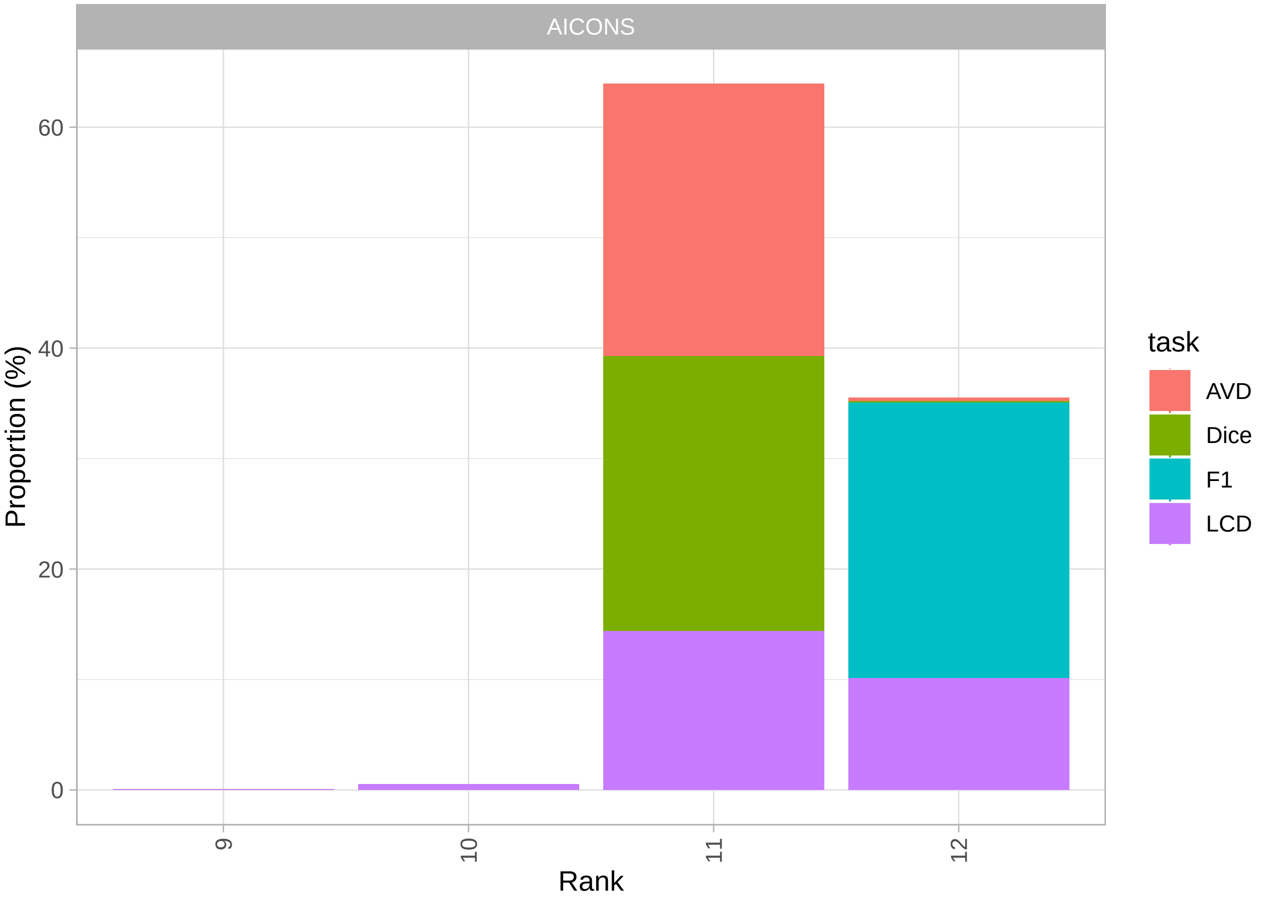

Figure S3.49: Stacked Rank Frequencies – AICONS.

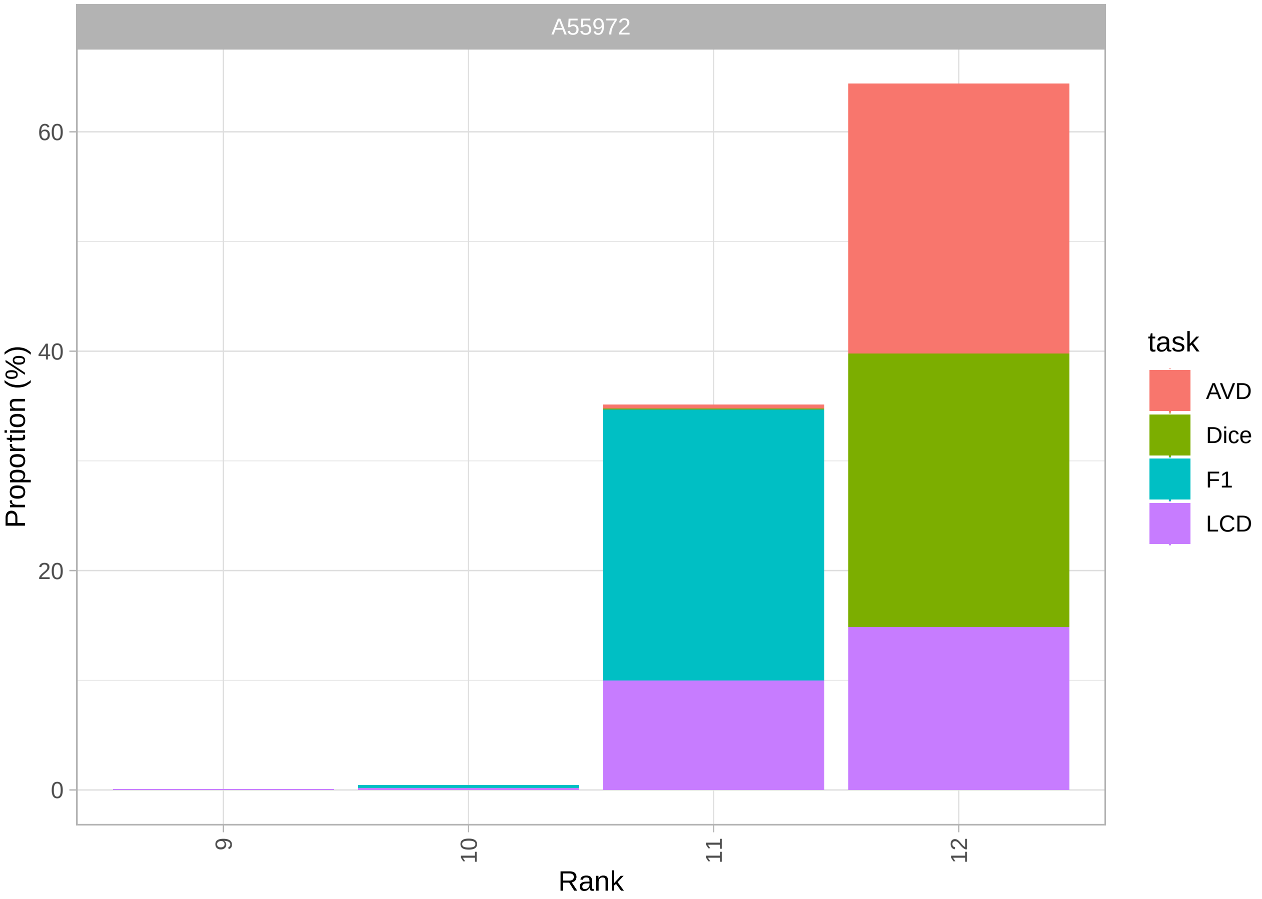

Figure S3.50: Stacked Rank Frequencies – A55972.

4.2 Characterization of tasks

4.2.1 Visualizing bootstrap results

To investigate which tasks separate algorithms well (i.e., lead to a stable ranking), a blob plot is recommended.

Bootstrap results can be shown in a blob plot showing one plot for each task. In this view, the spread of the blobs for each algorithm can be compared across tasks. Deviations from the diagonal indicate deviations from the consensus ranking (over tasks). Specifically, if rank distribution of an algorithm is consistently below the diagonal, the algorithm performed better in this task than on average across tasks, while if the rank distribution of an algorithm is consistently above the diagonal, the algorithm performed worse in this task than on average across tasks. At the bottom of each panel, ranks for each algorithm in the tasks are provided.

Same as in Section 3.1 but now ordered according to consensus.

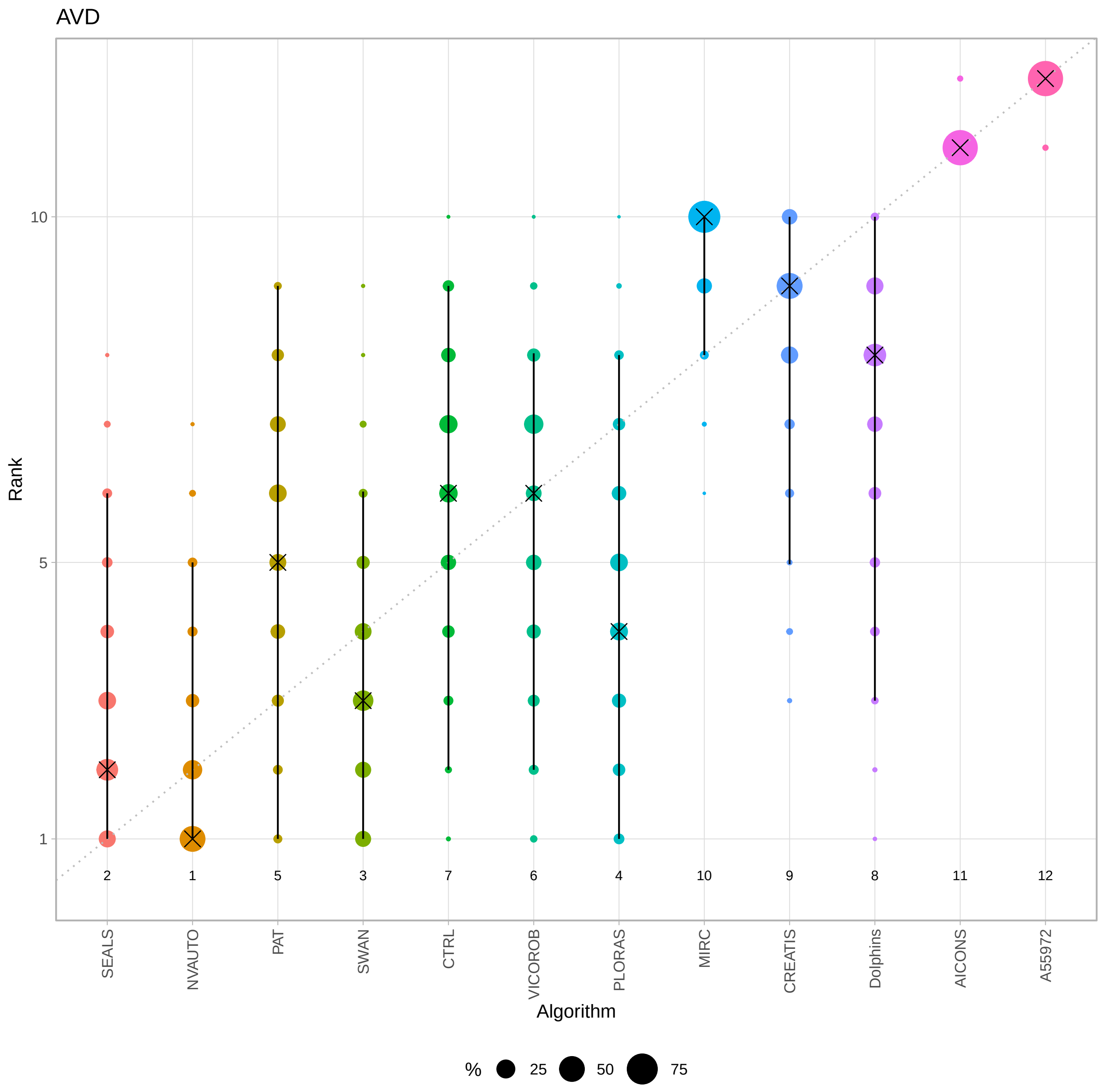

Figure S3.51: AVD Task-Level Ranking Stability – Blob Plot.

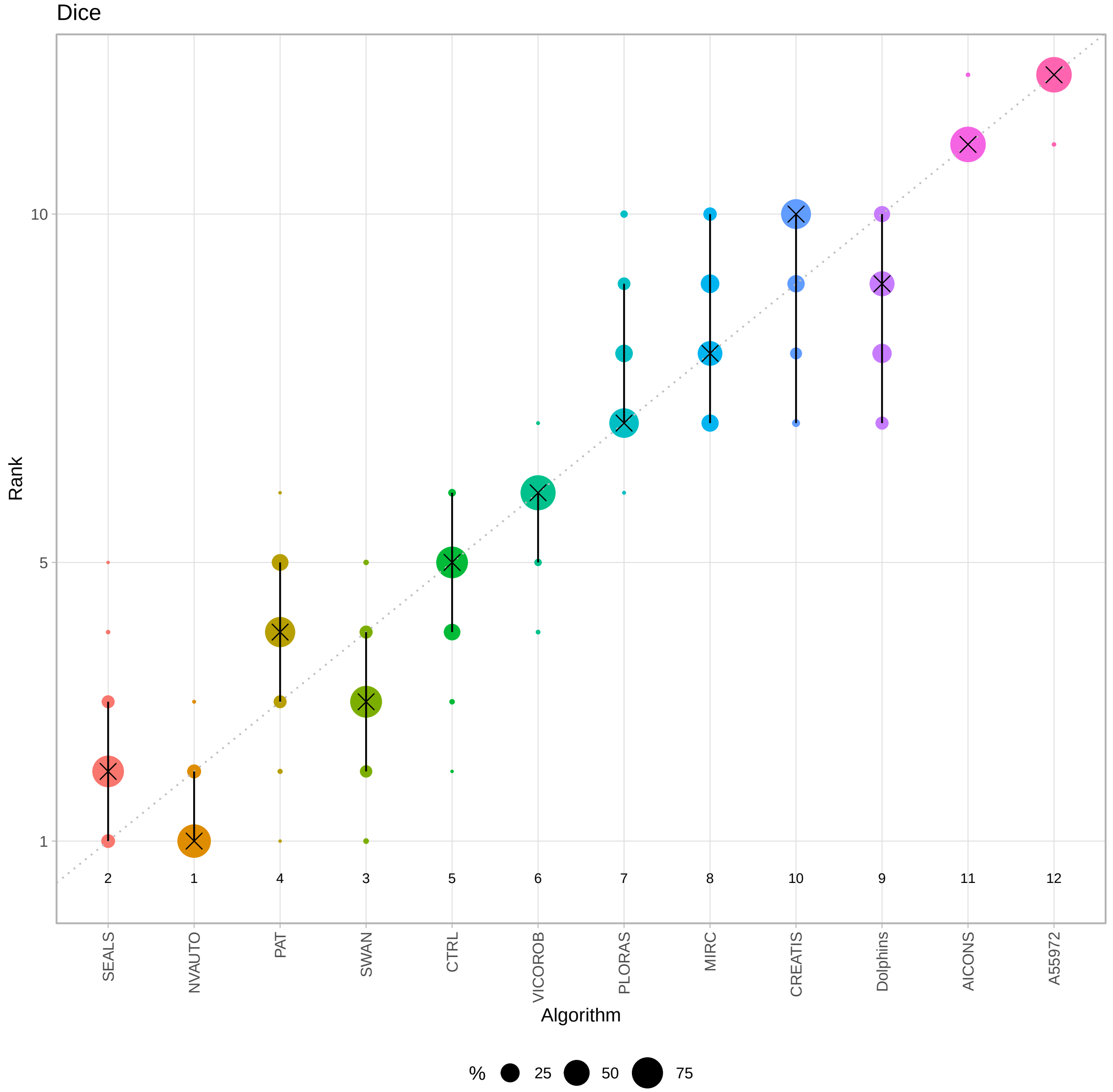

Figure S3.52: Dice Task-Level Ranking Stability – Blob Plot.

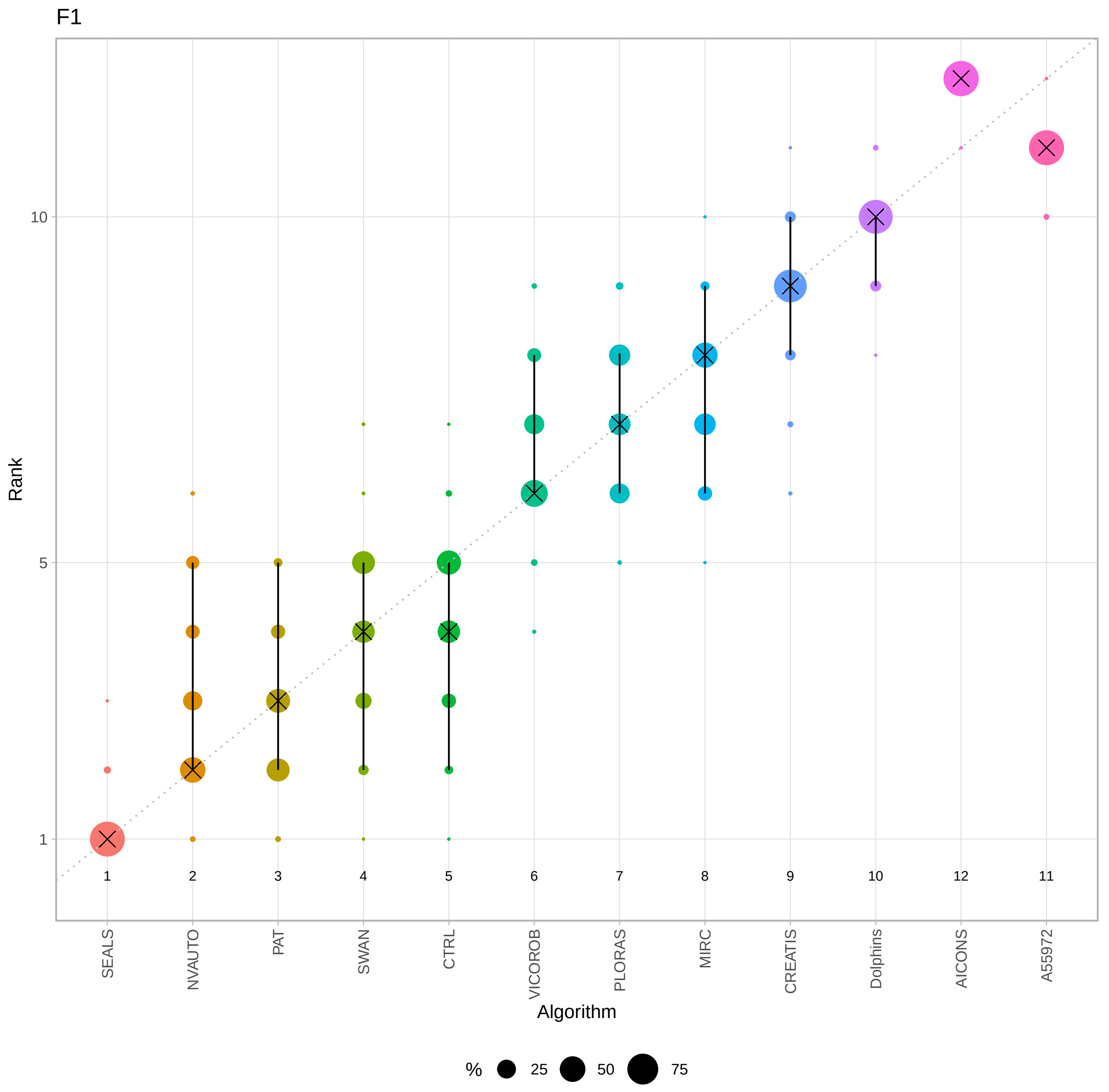

Figure S3.53: F1 Task-Level Ranking Stability – Blob Plot.

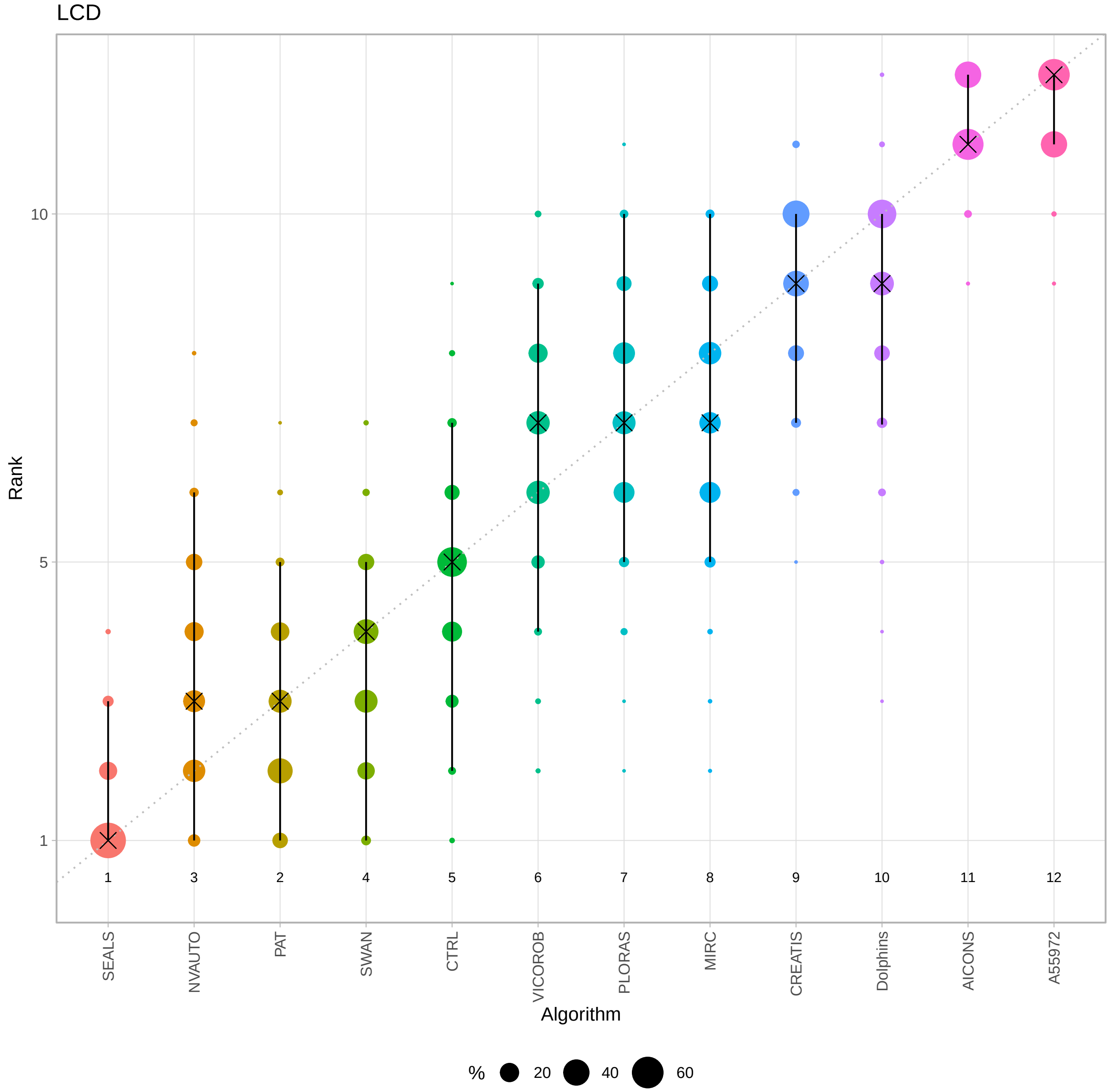

Figure S3.54: LCD Task-Level Ranking Stability – Blob Plot.

4.2.2 Cluster Analysis

Dendrogram from hierarchical cluster analysis and *network-type graphs* for assessing the similarity of tasks based on challenge rankings.

A dendrogram is a visualization approach based on hierarchical clustering. It depicts clusters according to a chosen distance measure (here: Spearman's footrule) as well as a chosen agglomeration method (here: complete and average agglomeration).

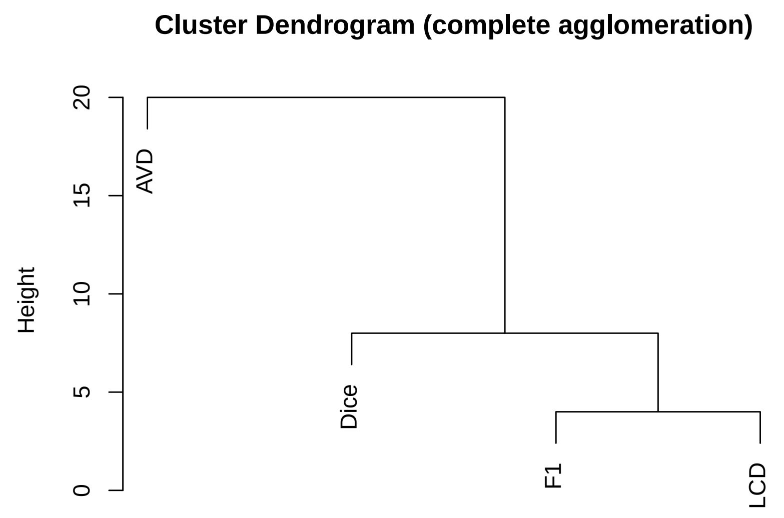

Figure S3.55: Cluster Dendrogram – Complete Linkage.

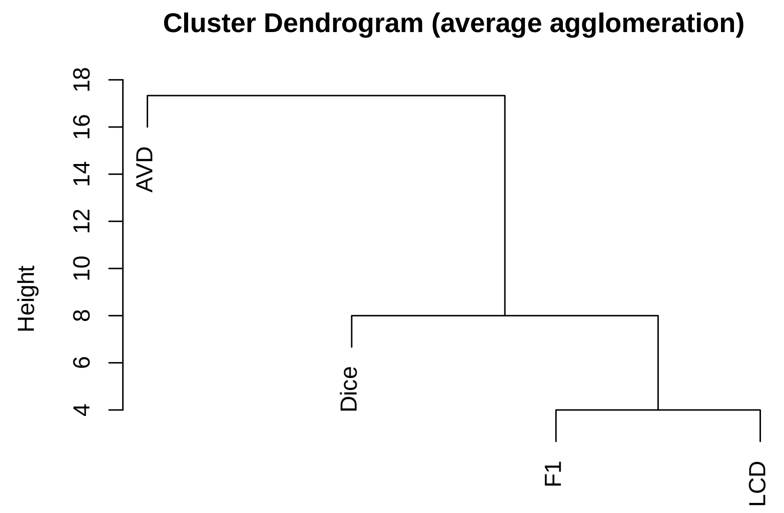

Figure S3.56: Cluster Dendrogram – Average Linkage.

5 References

Wiesenfarth, M., Reinke, A., Landman, B.A., Eisenmann, M., Aguilera Saiz, L., Cardoso, M.J., Maier-Hein, L. and Kopp-Schneider, A. Methods and open-source toolkit for analyzing and visualizing challenge results. *Sci Rep* **11**, 2369 (2021). https://doi.org/10.1038/s41598-021-82017-6

M. J. A. Eugster, T. Hothorn, and F. Leisch, "Exploratory and inferential analysis of benchmark experiments," Institut fuer Statistik, Ludwig-Maximilians-Universitaet Muenchen, Germany, Technical Report 30, 2008. [Online]. Available: http://epub.ub.uni-muenchen.de/4134/.

Supplementary material #4.

Benchmarking report for ISLES'22 - Post-challenge analysis: 'Aggregate then Rank' results.

created by challengeR v1.0.5

06 December, 2024

This document presents a systematic report on the benchmark study "ISLES'22 - Post-challenge analysis: 'Aggregate then Rank' results.". Input data comprises raw metric values for all algorithms and cases. Generated plots are:

- Visualization of assessment data: Dot- and boxplots, podium plots and ranking heatmaps
- Visualization of ranking stability: Blob plots, violin plots and significance maps, line plots
- Visualization of cross-task insights: Blob plots, stacked frequency plots, dendrograms

Details can be found in Wiesenfarth et al. (2021).

1 Rankings

Algorithms within a task are ranked according to the following ranking scheme:

aggregate using function ("mean") then rank

Ranking for each task:

AVD : The analysis is based on 12 algorithms and 150 cases. 0 missing cases have been found in the data set.

	Metric_value_mean	rank
PLORAS	-4.850038	1
SEALS	-4.950015	2
NVAUTO	-5.046373	3
SWAN	-5.145228	4
VICOROB	-5.520323	5
PAT	-5.808750	6
MIRC	-7.134733	7
Dolphins	-7.271432	8
CREATIS	-7.869850	9
AICONS	-24.921473	10
A55972	-28.698757	11
CTRL	-64.694314	12

Table S4.1: AVD Ranking.

Dice : The analysis is based on 12 algorithms and 150 cases. 0 missing cases have been found in the data set.

	Metric_value_mean	rank
NVAUTO	0.7830227	1
SEALS	0.7805508	2
SWAN	0.7632806	3
PAT	0.7521310	4
CTRL	0.7427561	5
VICOROB	0.7225404	6
PLORAS	0.7123595	7
MIRC	0.7035278	8
CREATIS	0.6881902	9
Dolphins	0.6790408	10
AICONS	0.3879143	11
A55972	0.1578497	12

Table S4.2: Dice Ranking.

F1 : The analysis is based on 12 algorithms and 150 cases. 0 missing cases have been found in the data set.

	Metric_value_mean	rank
SEALS	0.8041661	1
NVAUTO	0.7882487	2
PAT	0.7642120	3
SWAN	0.7525536	4
CTRL	0.7416228	5
VICOROB	0.6934707	6
PLORAS	0.6931600	7
MIRC	0.6851187	8
CREATIS	0.6715630	9
Dolphins	0.5735300	10
A55972	0.4414102	11
AICONS	0.2969900	12

Table S4.3: F1 Score Ranking.

LCD : The analysis is based on 12 algorithms and 150 cases. 0 missing cases have been found in the data set.

	Metric_value_mean	rank
SEALS	-2.326667	1
NVAUTO	-2.540000	2
PAT	-2.660000	3
SWAN	-2.886667	4
CTRL	-3.040000	5
VICOROB	-3.540000	6
MIRC	-3.660000	7
PLORAS	-3.766667	8
CREATIS	-4.400000	9
Dolphins	-5.513333	10
A55972	-6.313333	11
AICONS	-6.400000	12

Table S4.4: LCD Ranking.

Consensus ranking across tasks according to chosen method “euclidean”:

	value	rank
SEALS	1.50	1
NVAUTO	2.00	2
SWAN	3.75	3
PAT	4.00	4
PLORAS	5.75	5
VICOROB	5.75	5
CTRL	6.75	7
MIRC	7.50	8
CREATIS	9.00	9
Dolphins	9.50	10
A55972	11.25	11
AICONS	11.25	11

Table S4.5: Consensus Ranking Across Tasks.

2 Visualization of raw assessment data

The algorithms are ordered according to the computed ranks for each task.

2.1 Dot- and boxplot

Dot- and boxplots for visualizing raw assessment data separately for each algorithm. Boxplots representing descriptive statistics over all cases (median, quartiles and outliers) are combined with horizontally jittered dots representing individual cases.

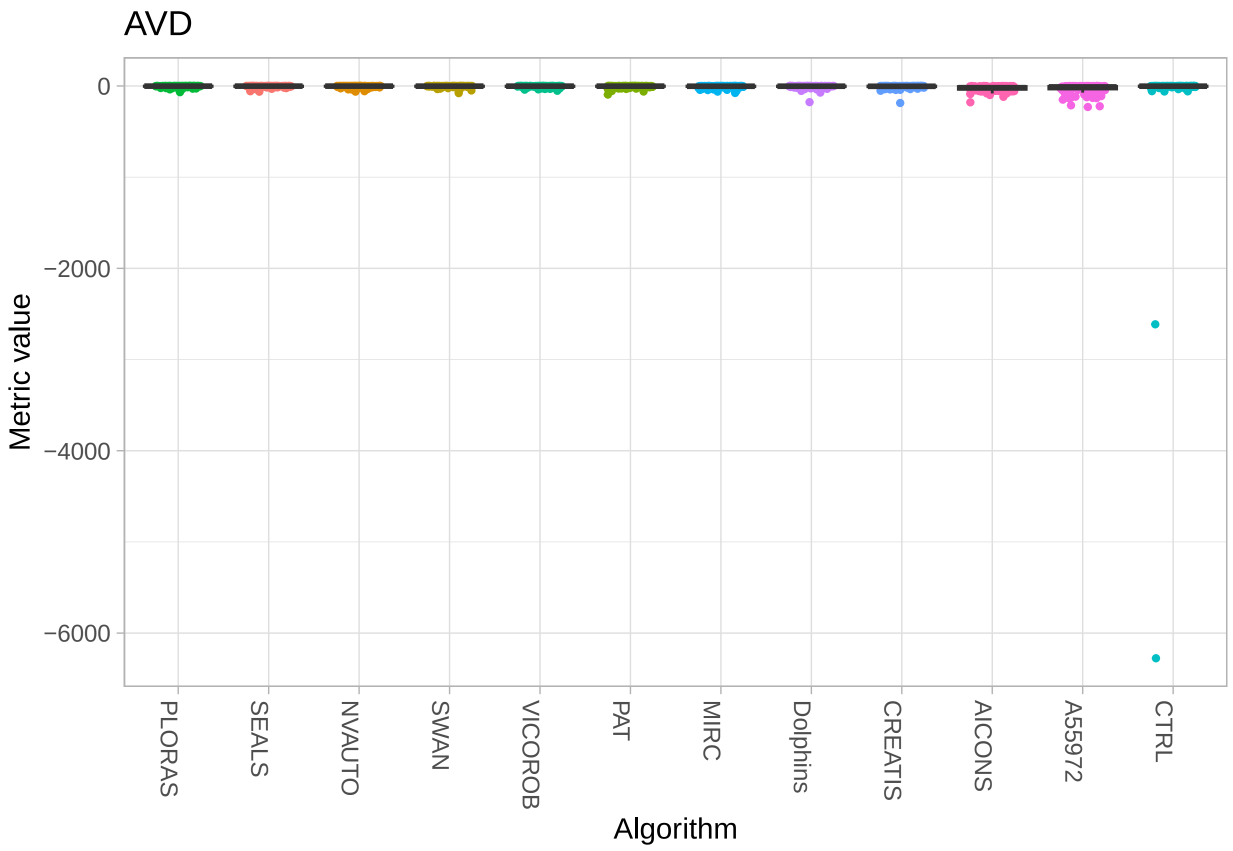

Figure S4.1: AVD Boxplots.

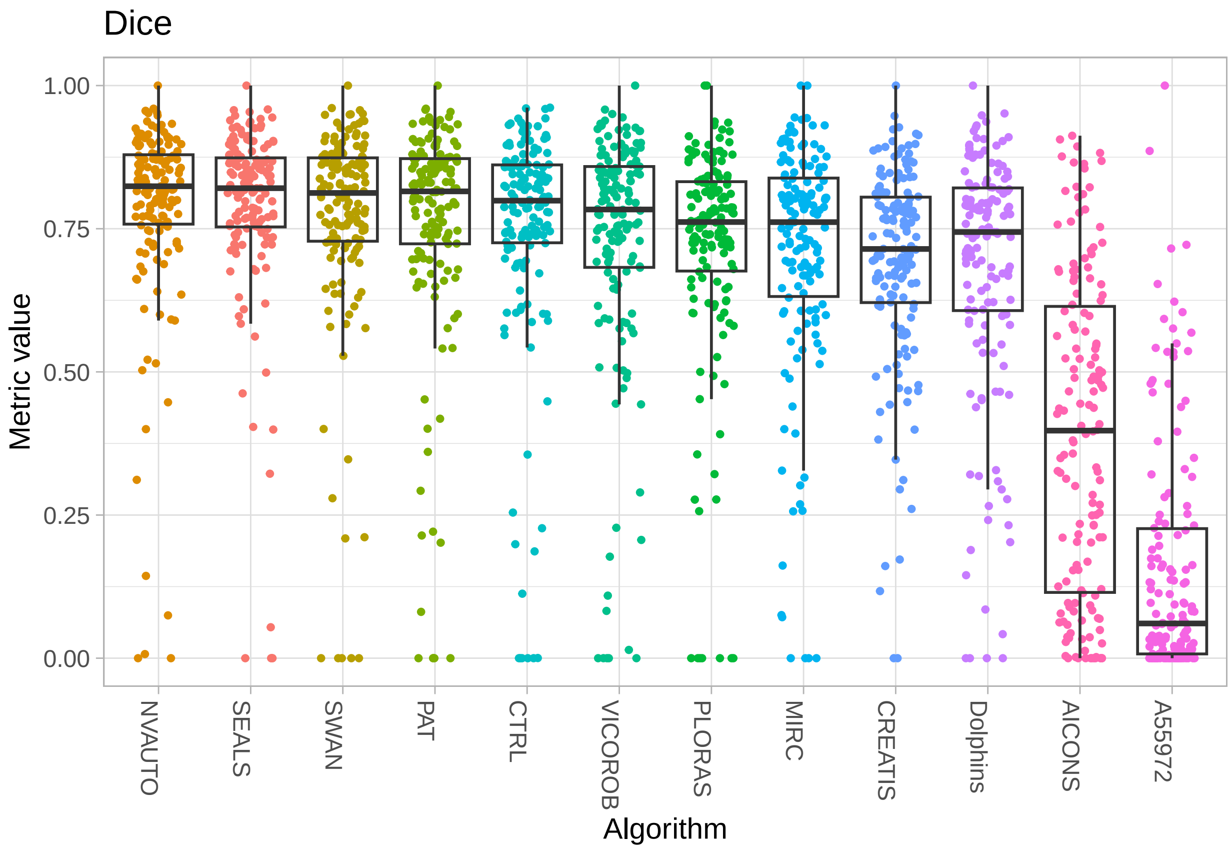

Figure S4.2: Dice Boxplots.

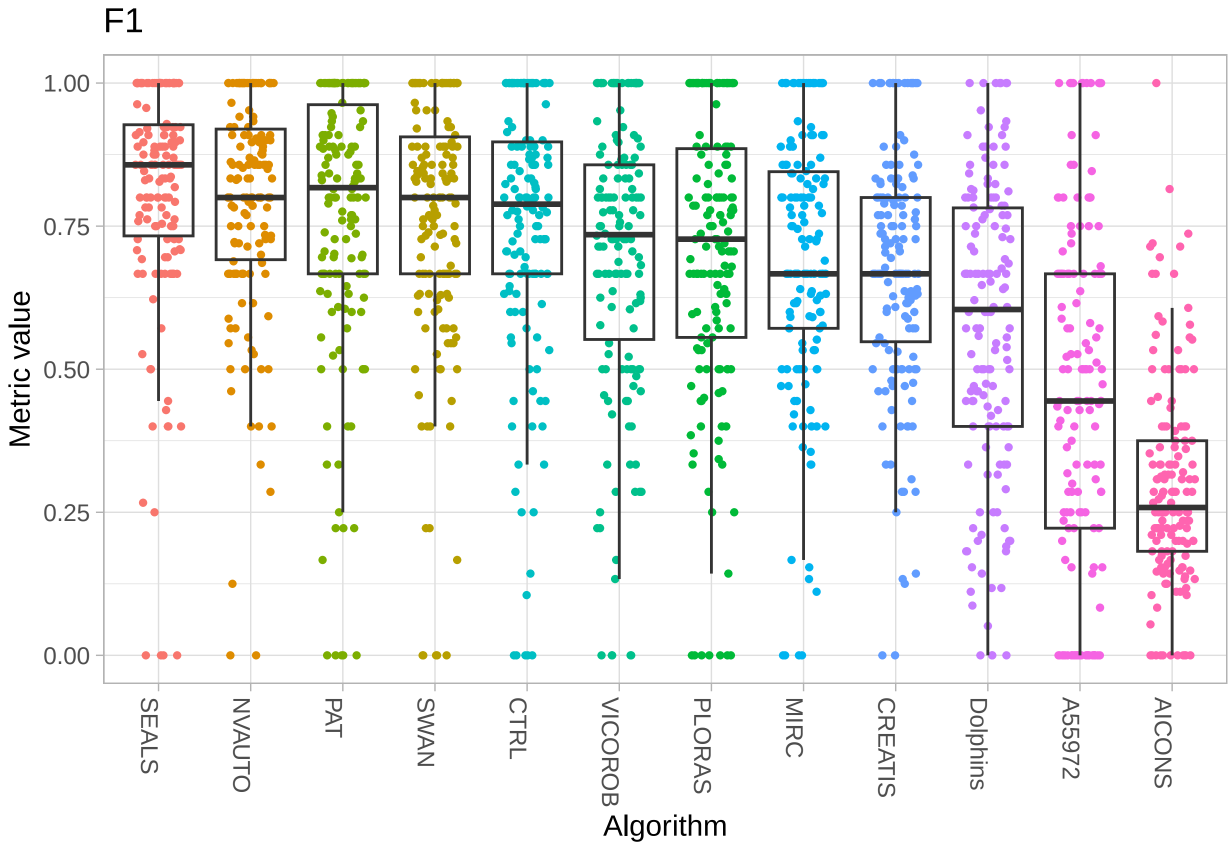

Figure S4.3: F1 Boxplots.

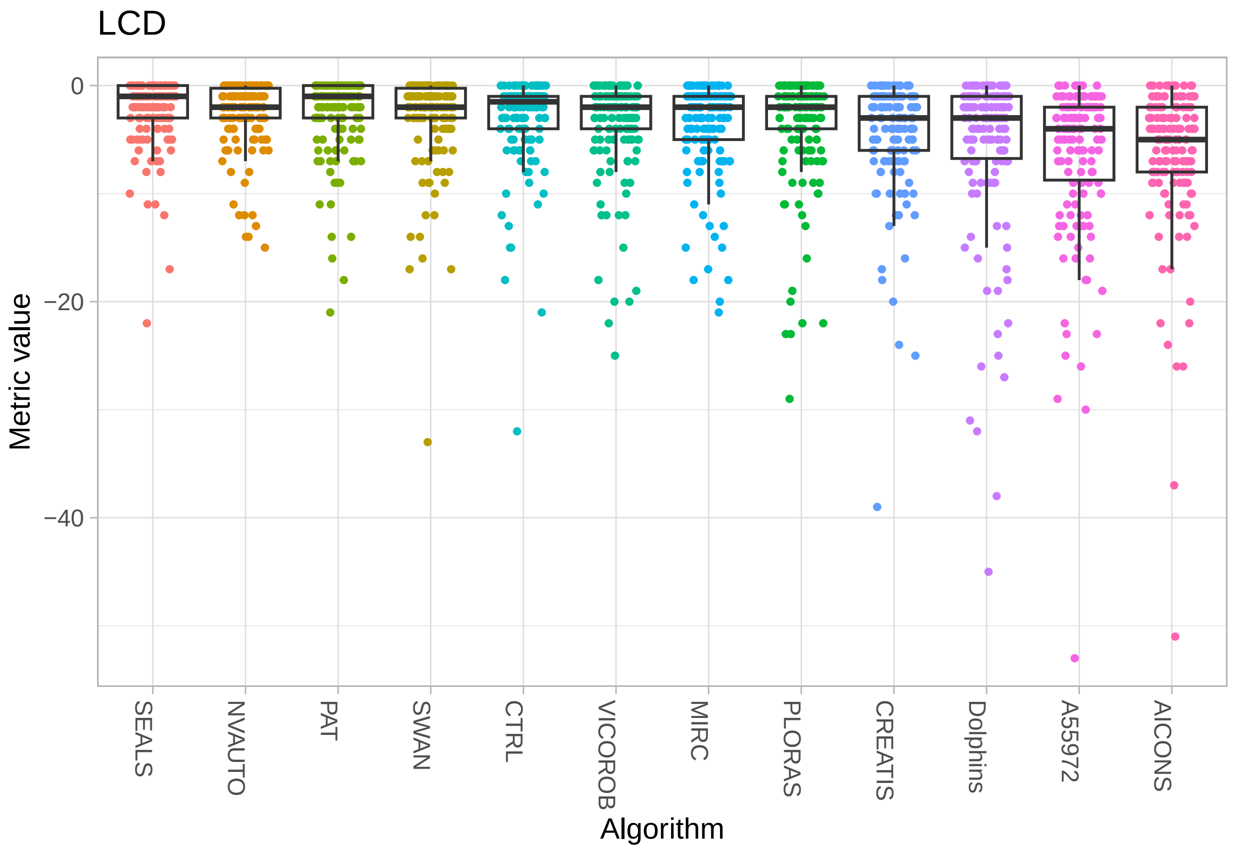

Figure S4.4: LCD Boxplots.

2.2 Podium plot

Podium plots (see also Eugster et al., 2008) for visualizing raw assessment data. Upper part (spaghetti plot): Participating algorithms are color-coded, and each colored dot in the plot represents a metric value achieved with the respective algorithm. The actual metric value is encoded by the y-axis. Each podium (here: p=12) represents one possible rank, ordered from best (1) to last (here: 12). The assignment of metric values (i.e. colored dots) to one of the podiums is based on the rank that the respective algorithm achieved on the corresponding case. Note that the plot part above each podium place is further subdivided into p "columns", where each column represents one participating algorithm (here: p = 12). Dots corresponding to identical cases are connected by a line, leading to the shown spaghetti structure. Lower part: Bar charts represent the relative frequency for each algorithm to achieve the rank encoded by the podium place.

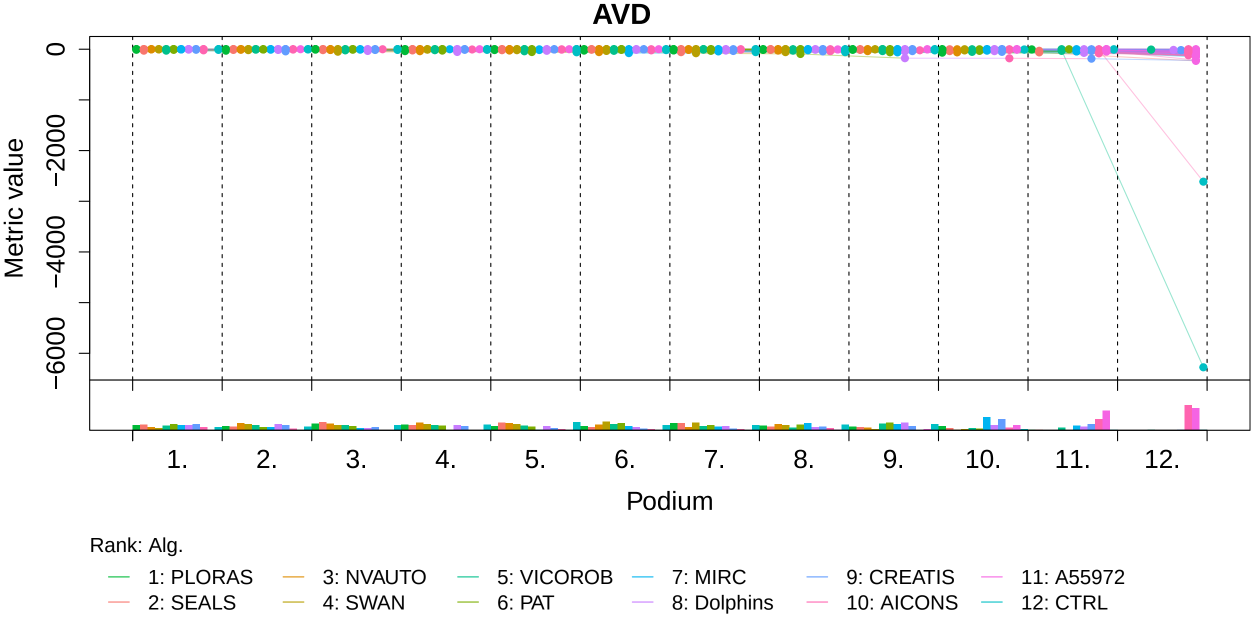

Figure S4.5: AVD Podium Plot.

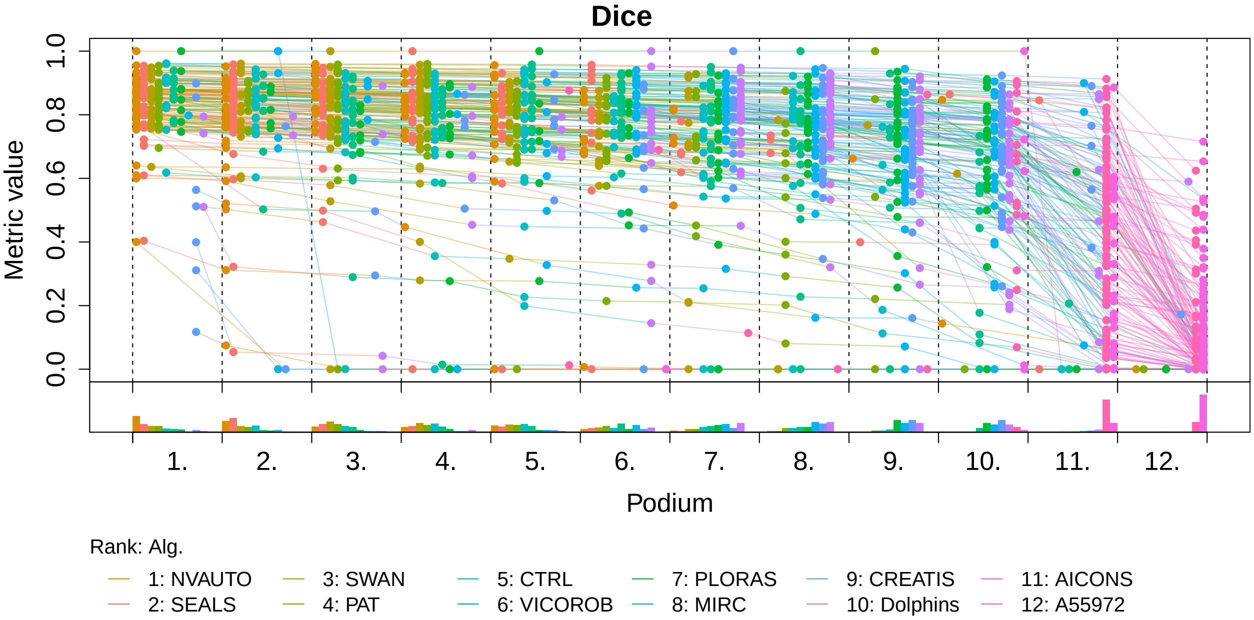

Figure S4.6: Dice Podium Plot.

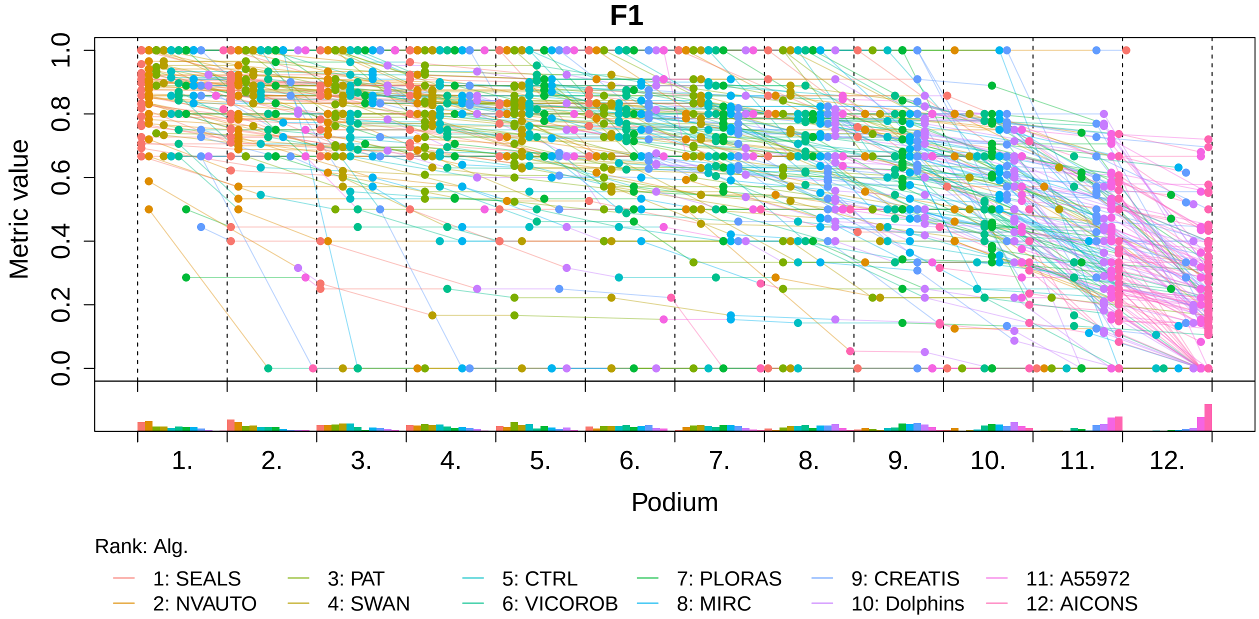

Figure S4.7: F1 Podium Plot.

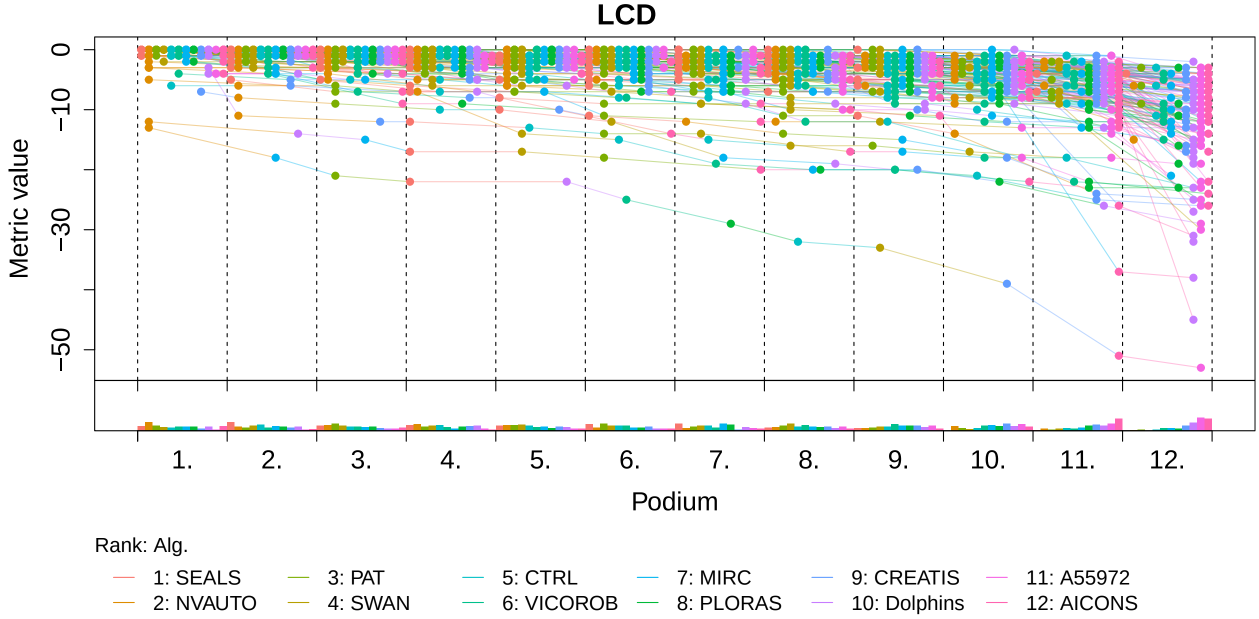

Figure S4.8: LCD Podium Plot.

2.3 Ranking heatmap

Ranking heatmaps for visualizing raw assessment data. Each cell (i, A_j) shows the absolute frequency of cases in which algorithm A_j achieved rank i.

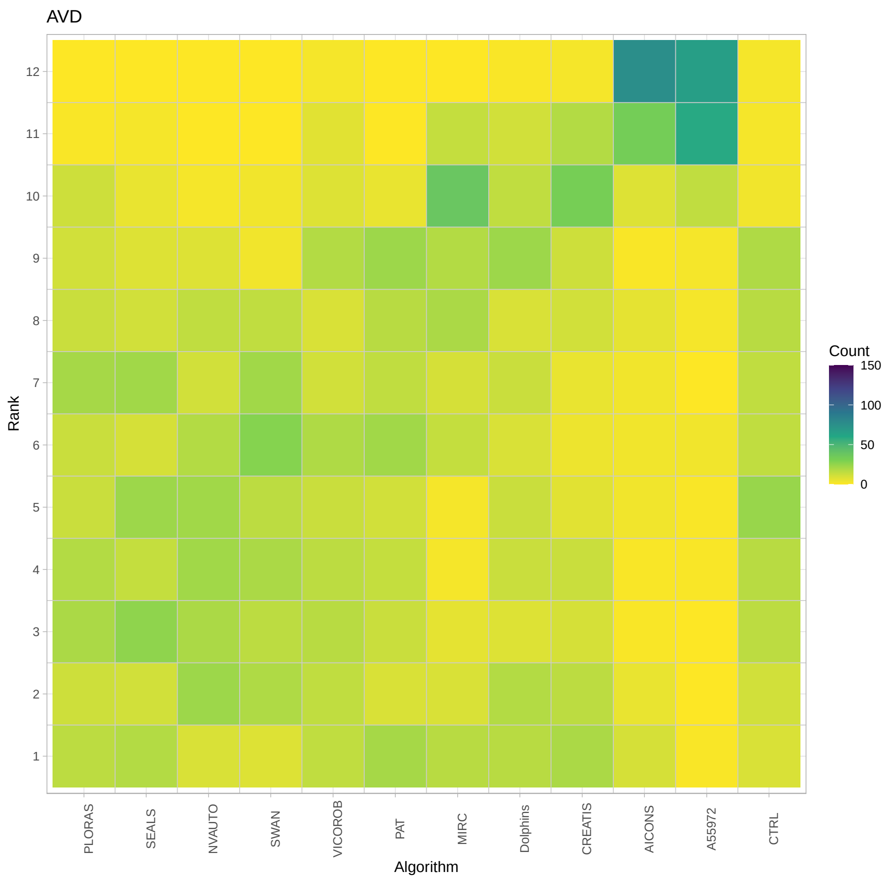

Figure S4.9: AVD Ranking Heatmap.

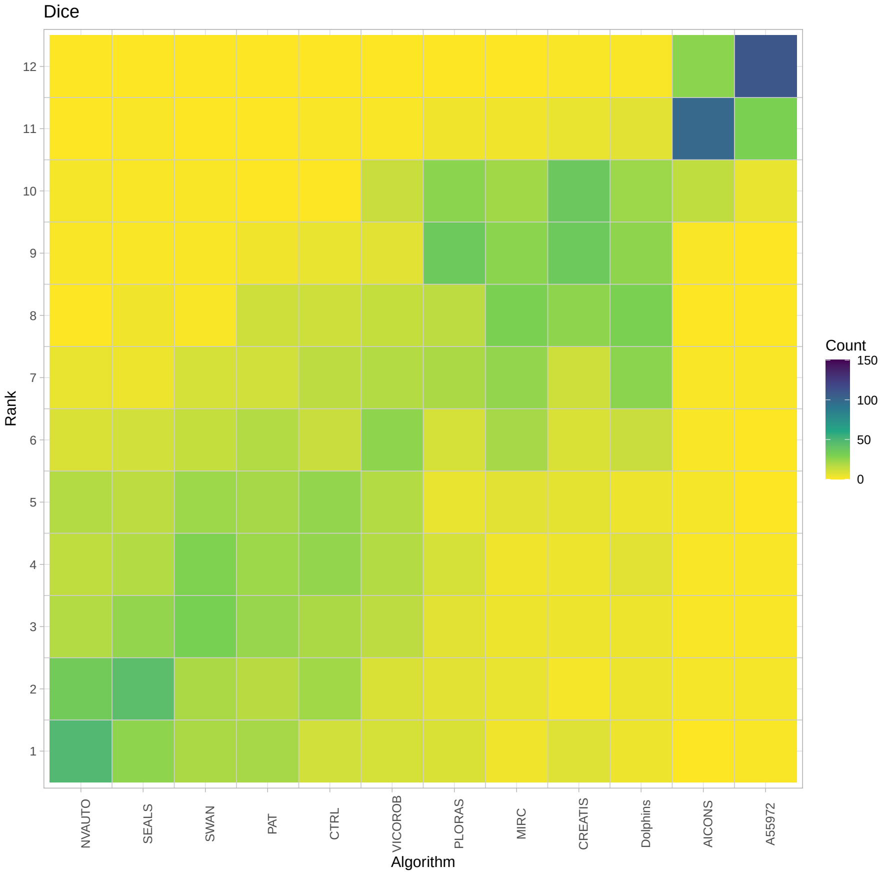

Figure S4.10: Dice Ranking Heatmap.

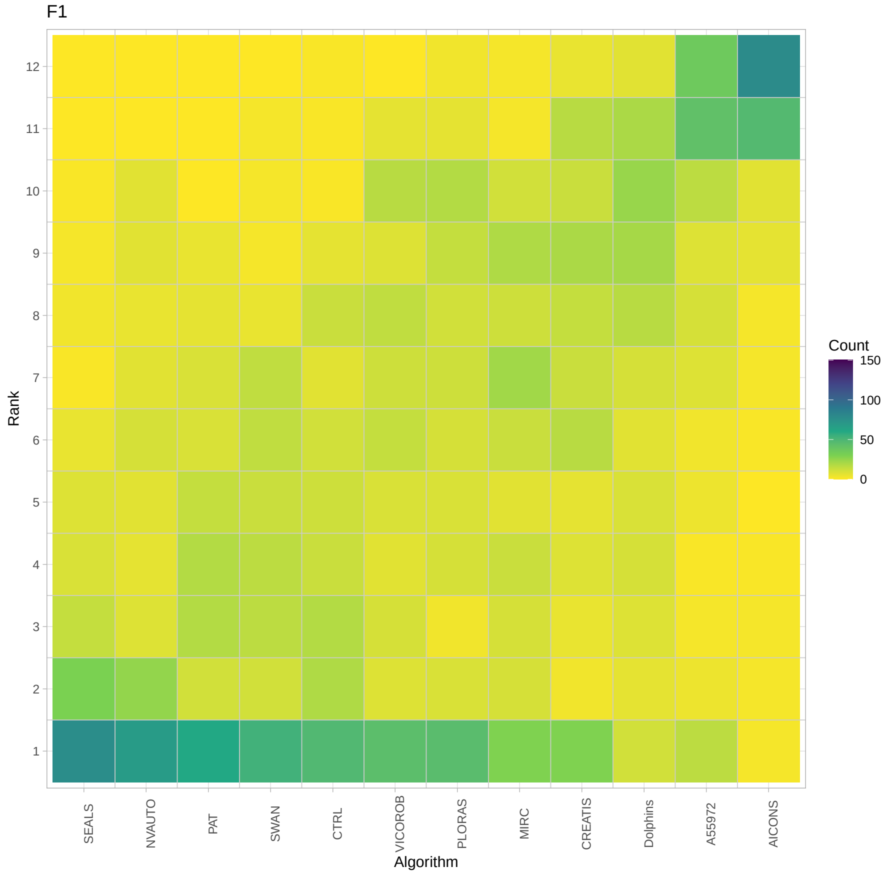

Figure S4.11: F1 Ranking Heatmap.

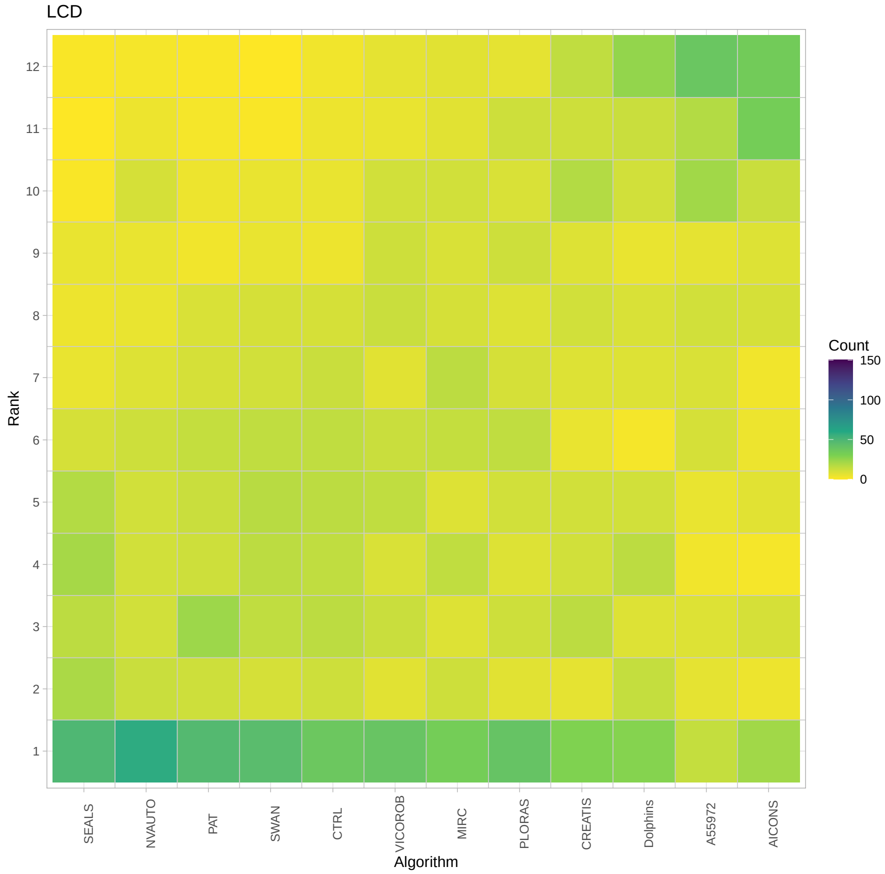

Figure S4.12: LCD Ranking Heatmap.

3 Visualization of ranking stability

3.1 *Blob plot* for visualizing ranking stability based on bootstrap sampling

Algorithms are color-coded, and the area of each blob at position $(A_i, \text{rank } j)$ is proportional to the relative frequency A_i achieved rank j across $b = 1000$ bootstrap samples. The median rank for each algorithm is indicated by a black cross. 95% bootstrap intervals across bootstrap samples are indicated by black lines.

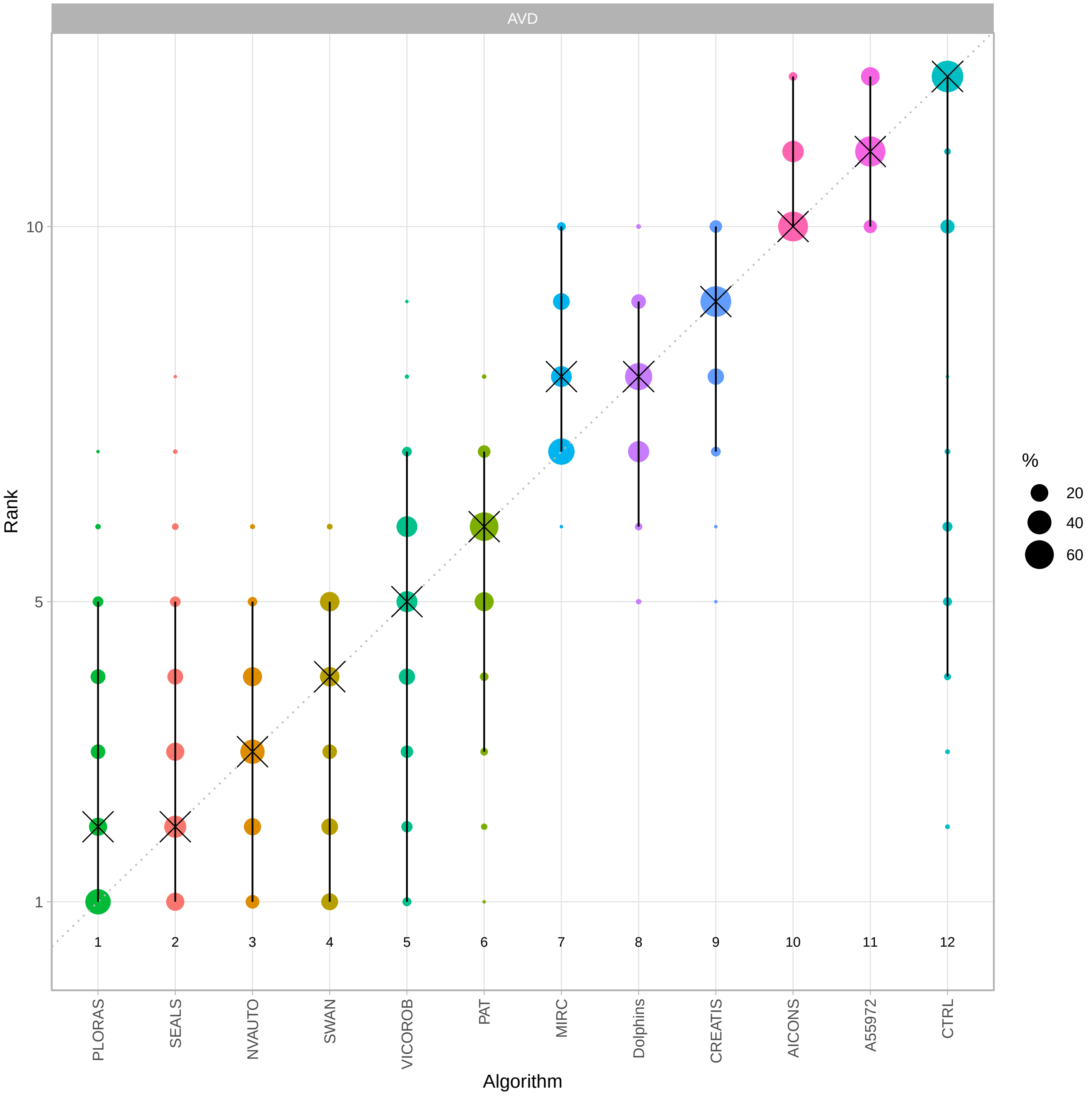

Figure S4.13: AVD Ranking Stability – Blob Plot.

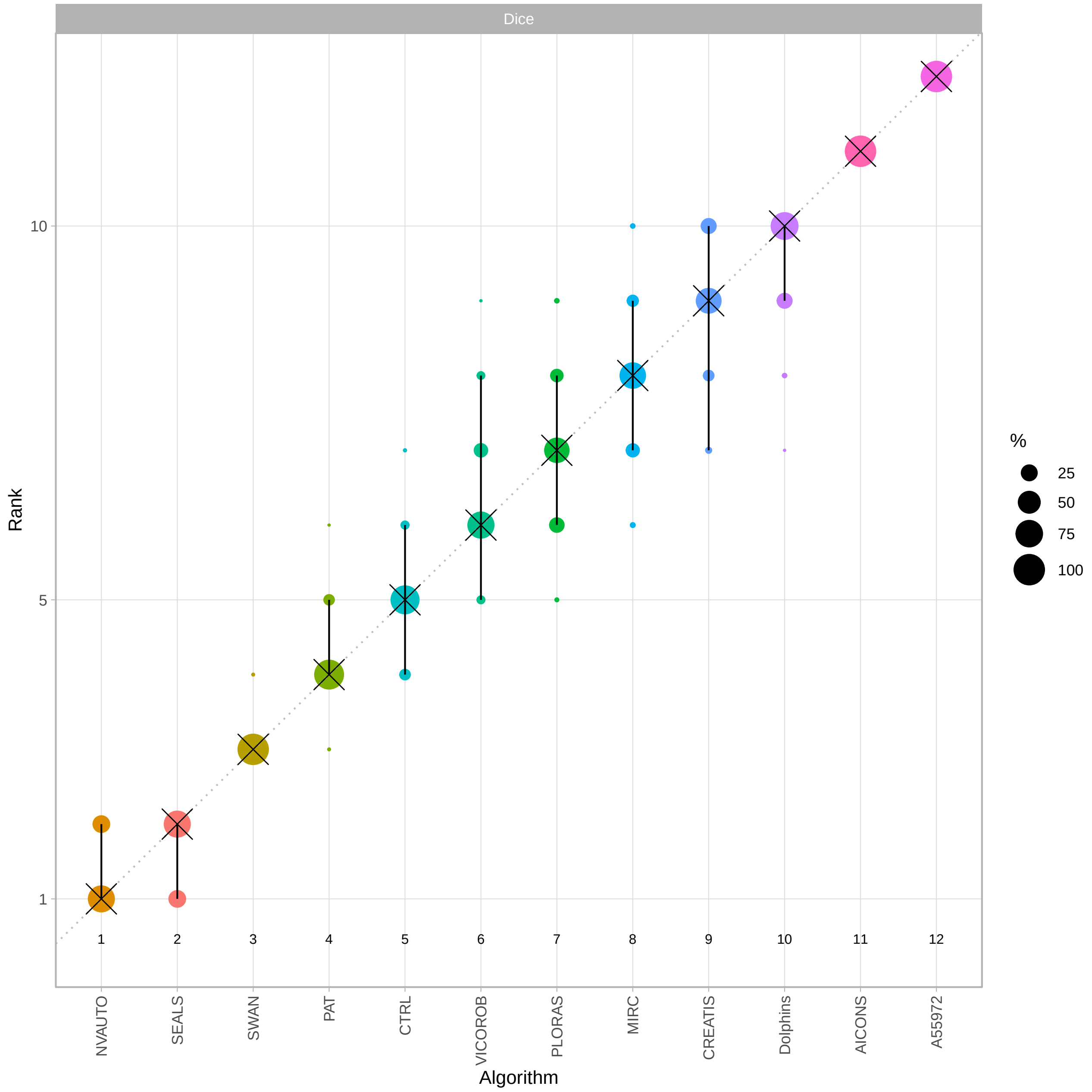

Figure S4.14: Dice Ranking Stability – Blob Plot.

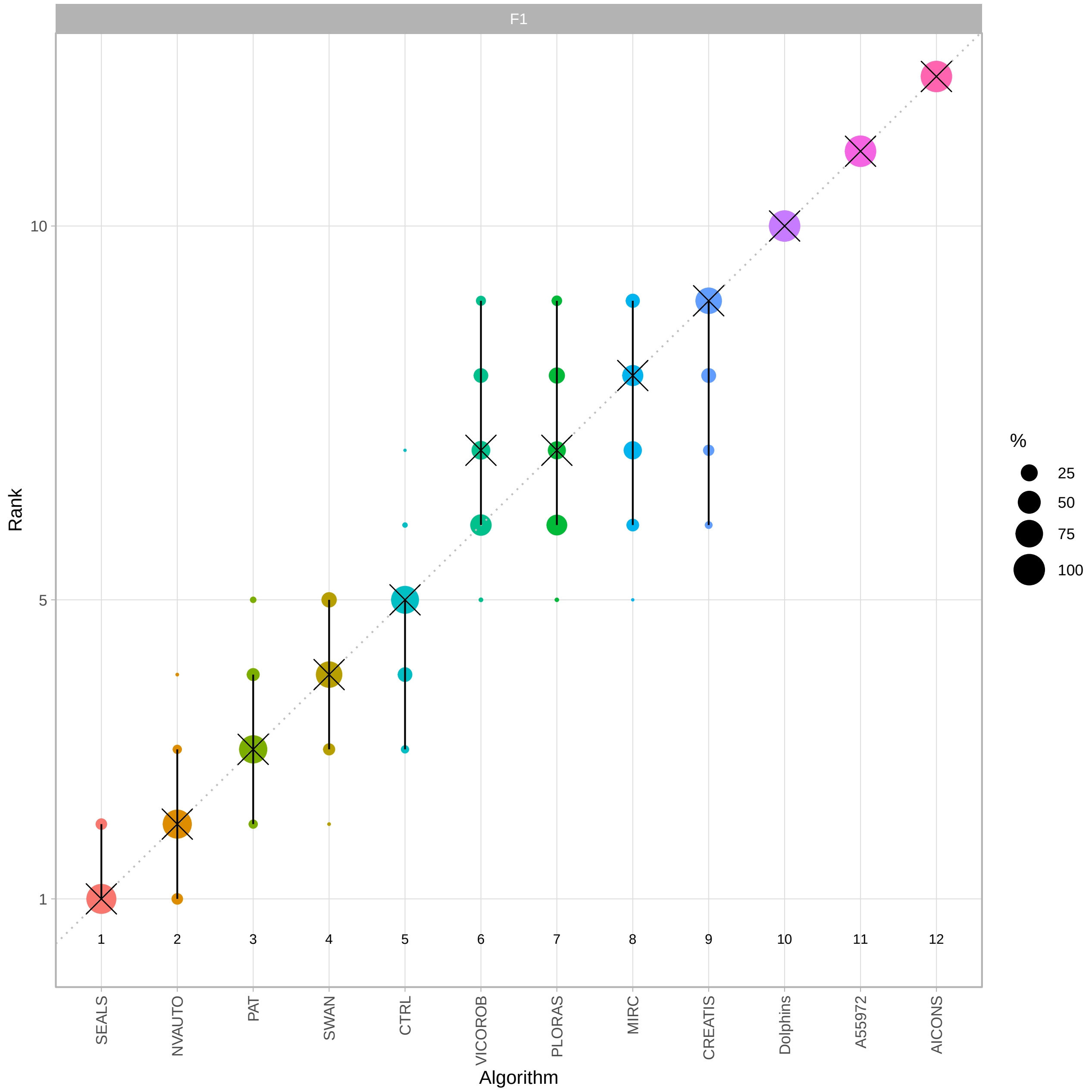

Figure S4.15: F1 Ranking Stability – Blob Plot.

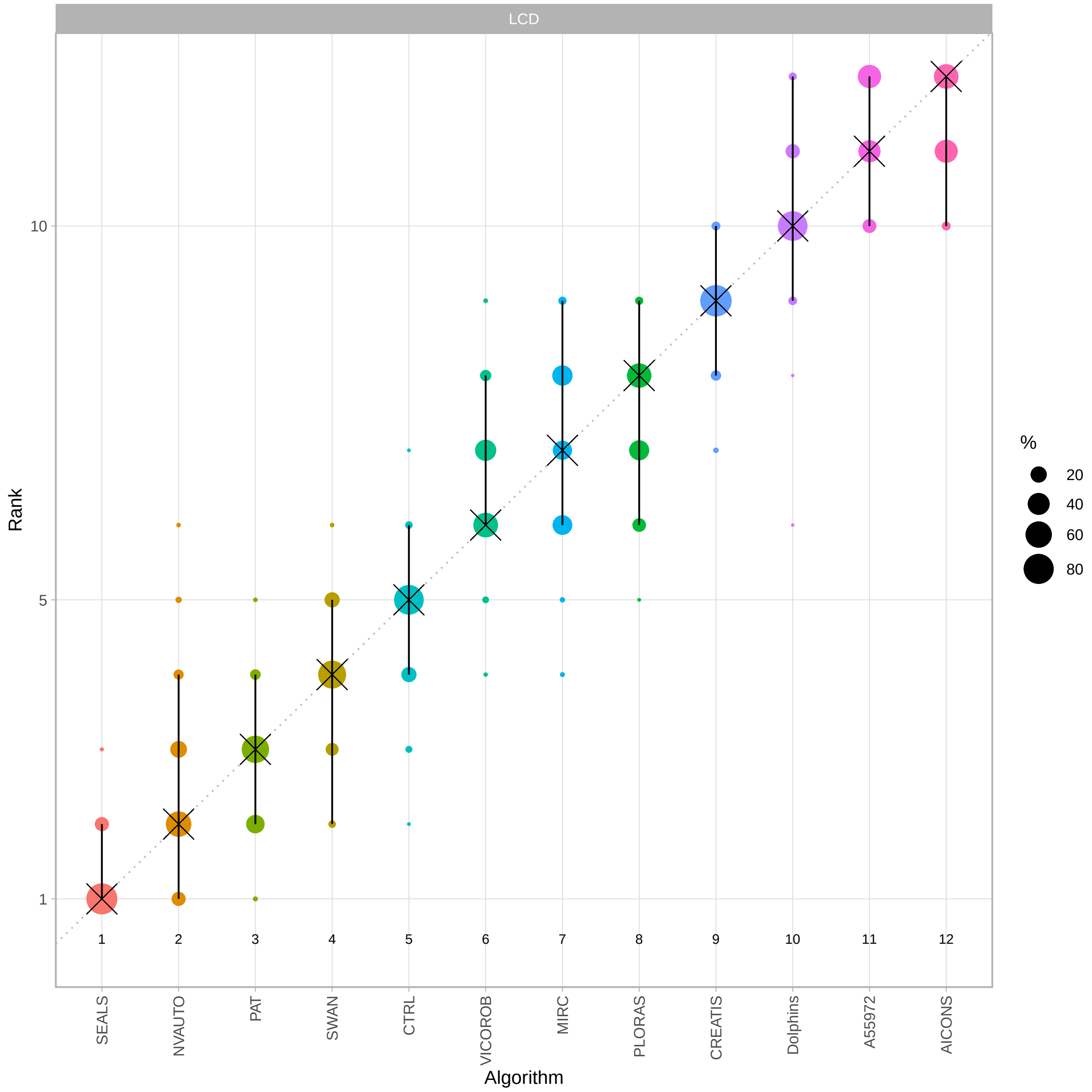

Figure S4.16: LCD Ranking Stability – Blob Plot.

3.2 *Violin plot* for visualizing ranking stability based on bootstrapping

The ranking list based on the full assessment data is pairwise compared with the ranking lists based on the individual bootstrap samples (here $b = 1000$ samples). For each pair of rankings, Kendall's τ correlation is computed. Kendall's τ is a scaled index determining the correlation between the lists. It is computed by evaluating the number of pairwise concordances and discordances between ranking lists and produces values between -1 (for inverted order) and 1 (for identical order). A violin plot, which simultaneously depicts a boxplot and a density plot, is generated from the results.

Summary Kendall's tau:

Task	mean	median	q25	q75
Dice	0.9616364	0.9696970	0.9393939	0.9696970
F1	0.9359091	0.9393939	0.9090909	0.9696970
LCD	0.9195329	0.9393939	0.8787879	0.9393939
AVD	0.8371212	0.8484848	0.7575758	0.9393939

Table S4.6: Kendall's Tau Summary for Ranking Stability.

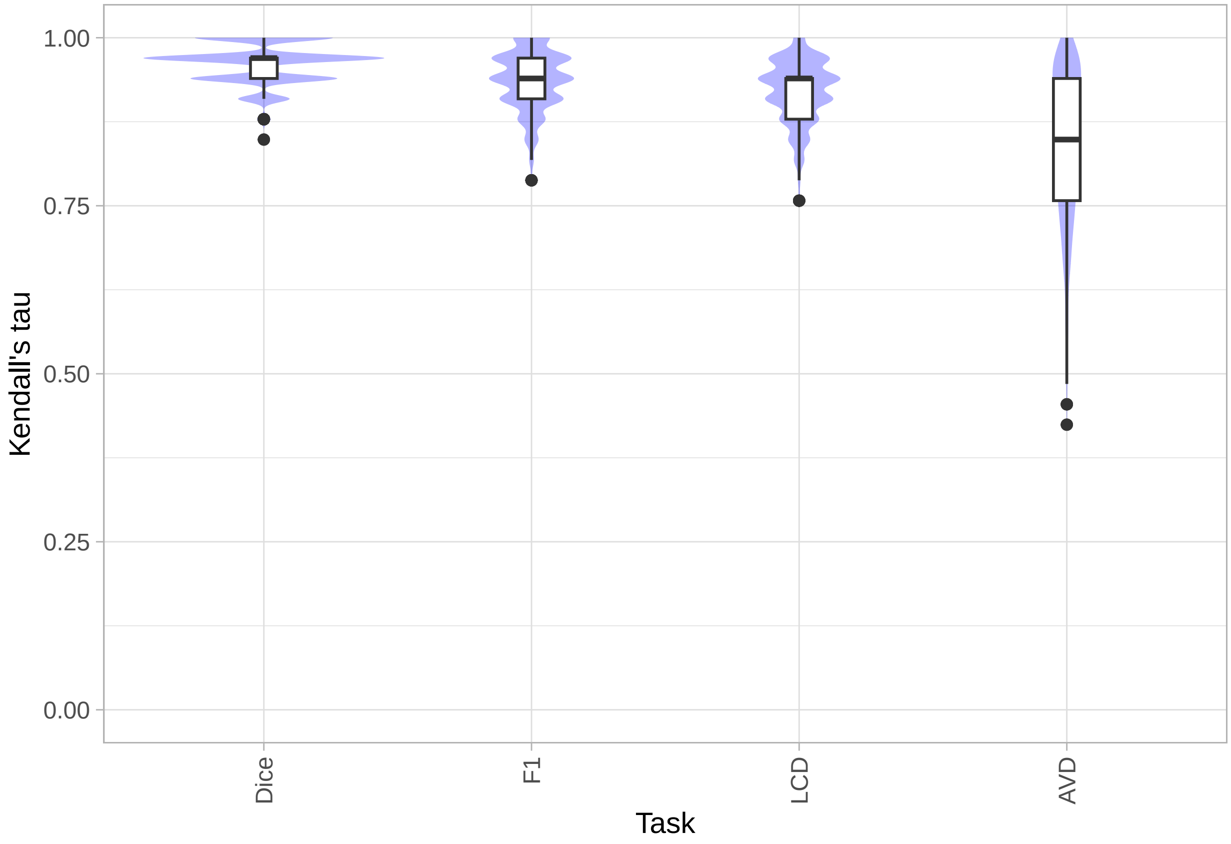

Figure S4.17: Kendall's Tau Violin Plot for All Metrics.

3.3 *Significance maps* for visualizing ranking stability based on statistical significance

Significance maps depict incidence matrices of pairwise significant test results for the one-sided Wilcoxon signed rank test at a 5% significance level with adjustment for multiple testing according to Holm. Yellow shading indicates that metric values from the algorithm on the x-axis were significantly superior to those from the algorithm on the y-axis, blue color indicates no significant difference.

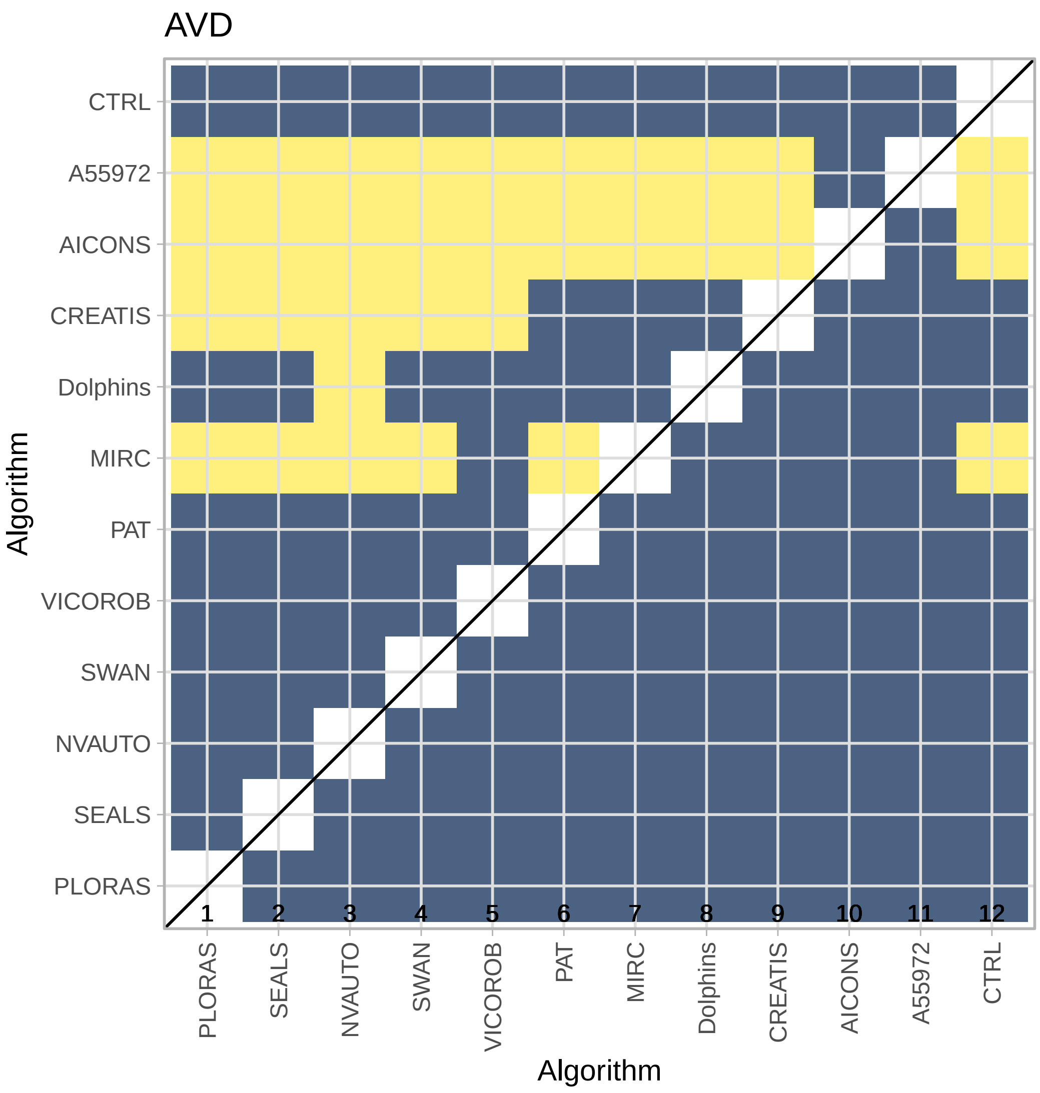

Figure S4.18: AVD Significance Map.

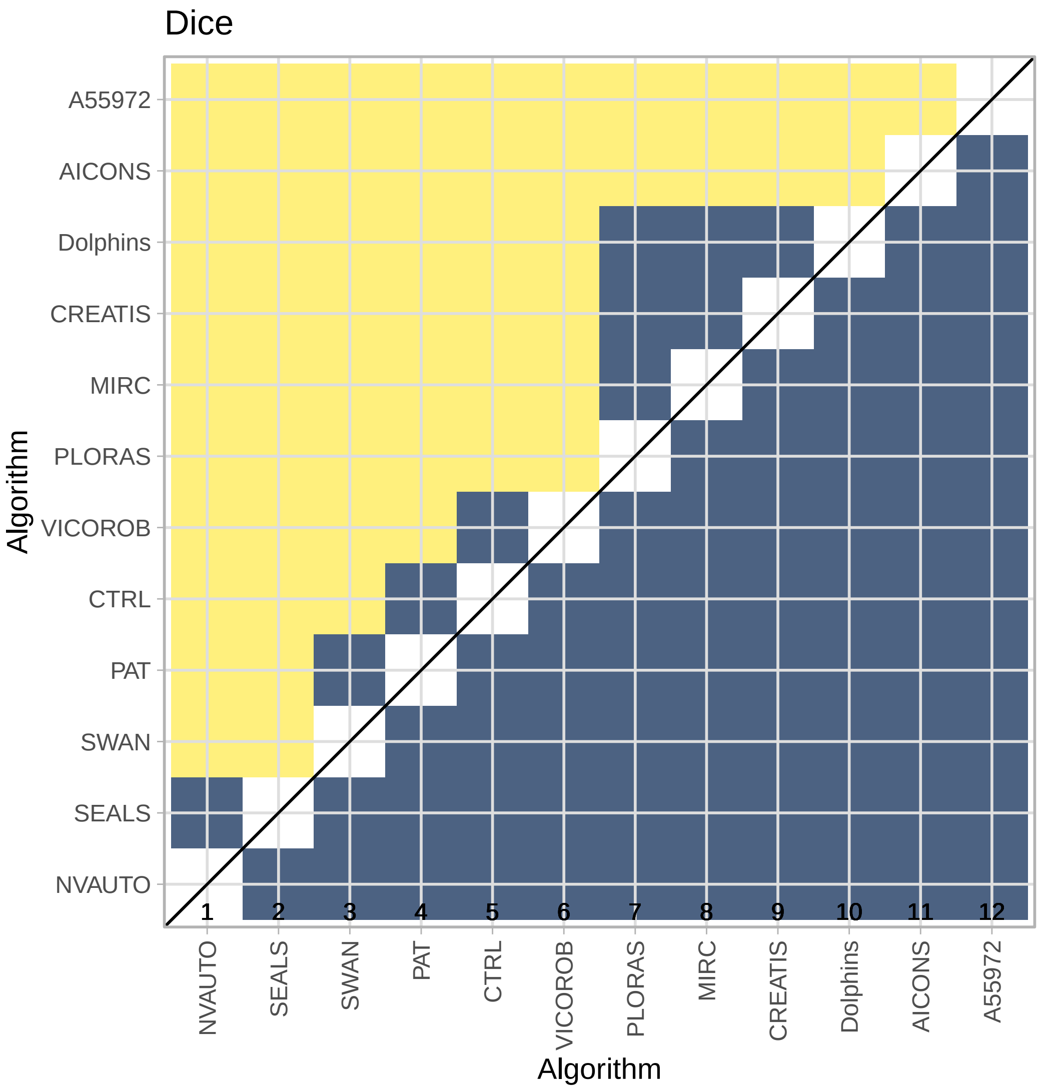

Figure S4.19: Dice Significance Map.

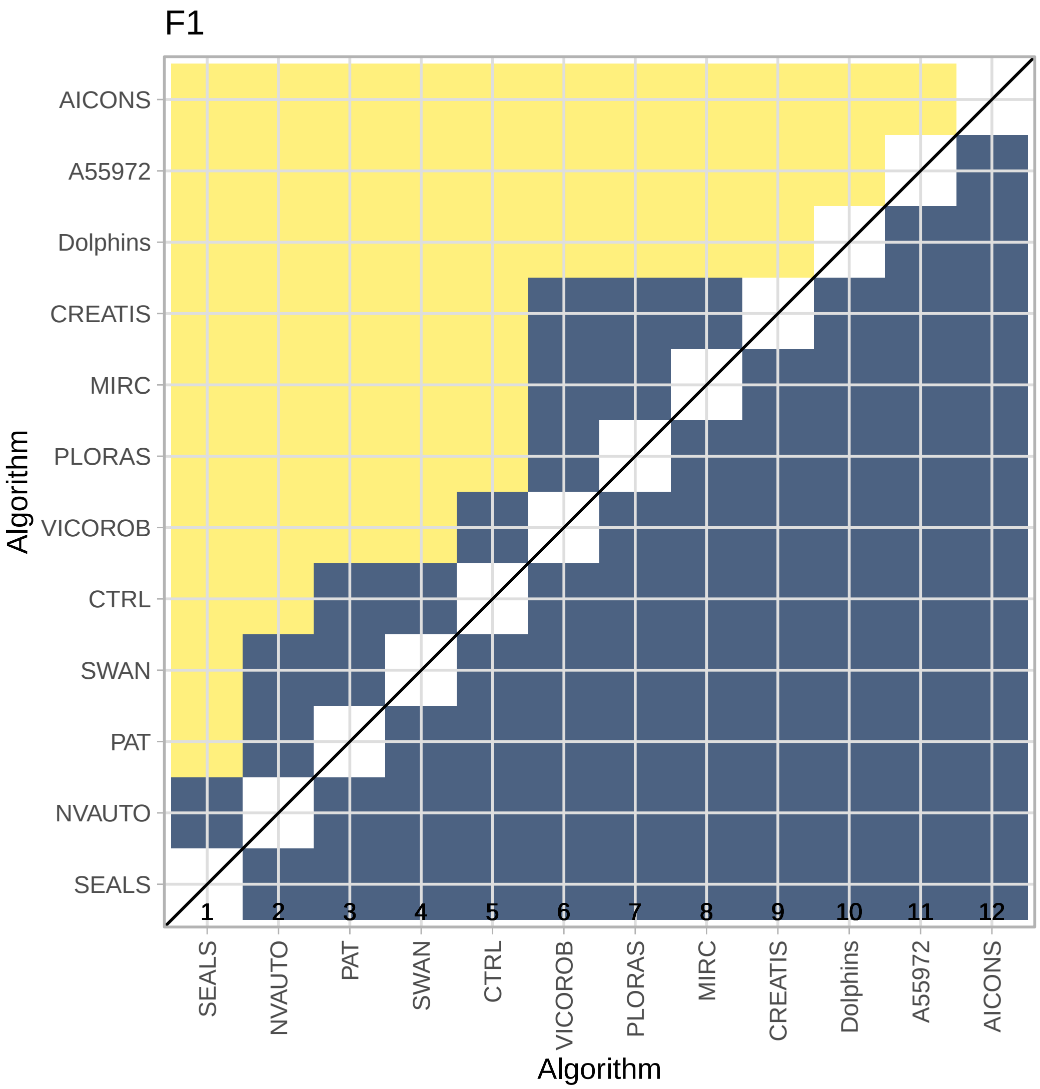

Figure S4.20: F1 Significance Map.

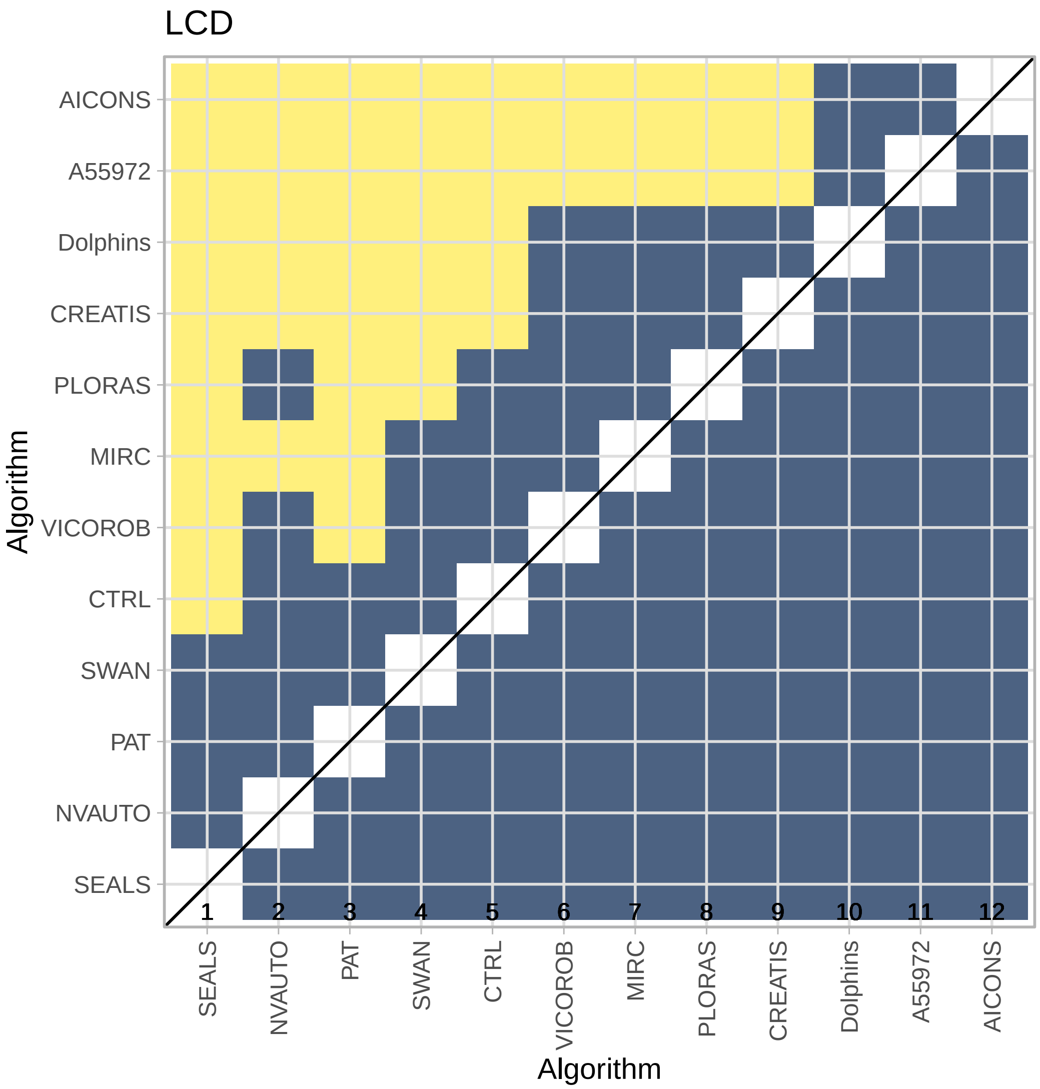

Figure S4.21: LCD Significance Map.

3.4 Ranking robustness to ranking methods

Line plots for visualizing ranking robustness across different ranking methods. Each algorithm is represented by one colored line. For each ranking method encoded on the x-axis, the height of the line represents the corresponding rank. Horizontal lines indicate identical ranks for all methods.

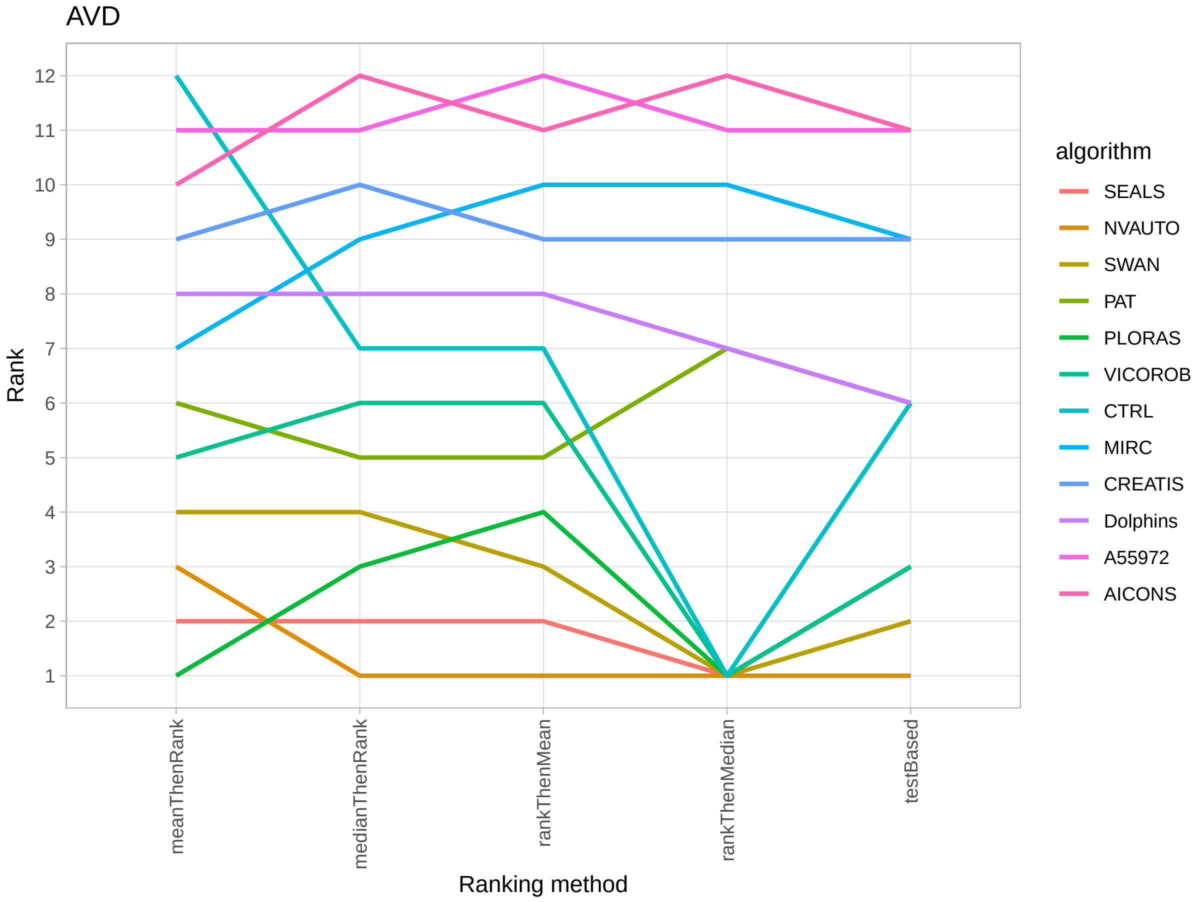

Figure S4.22: AVD Ranking Robustness Across Methods.

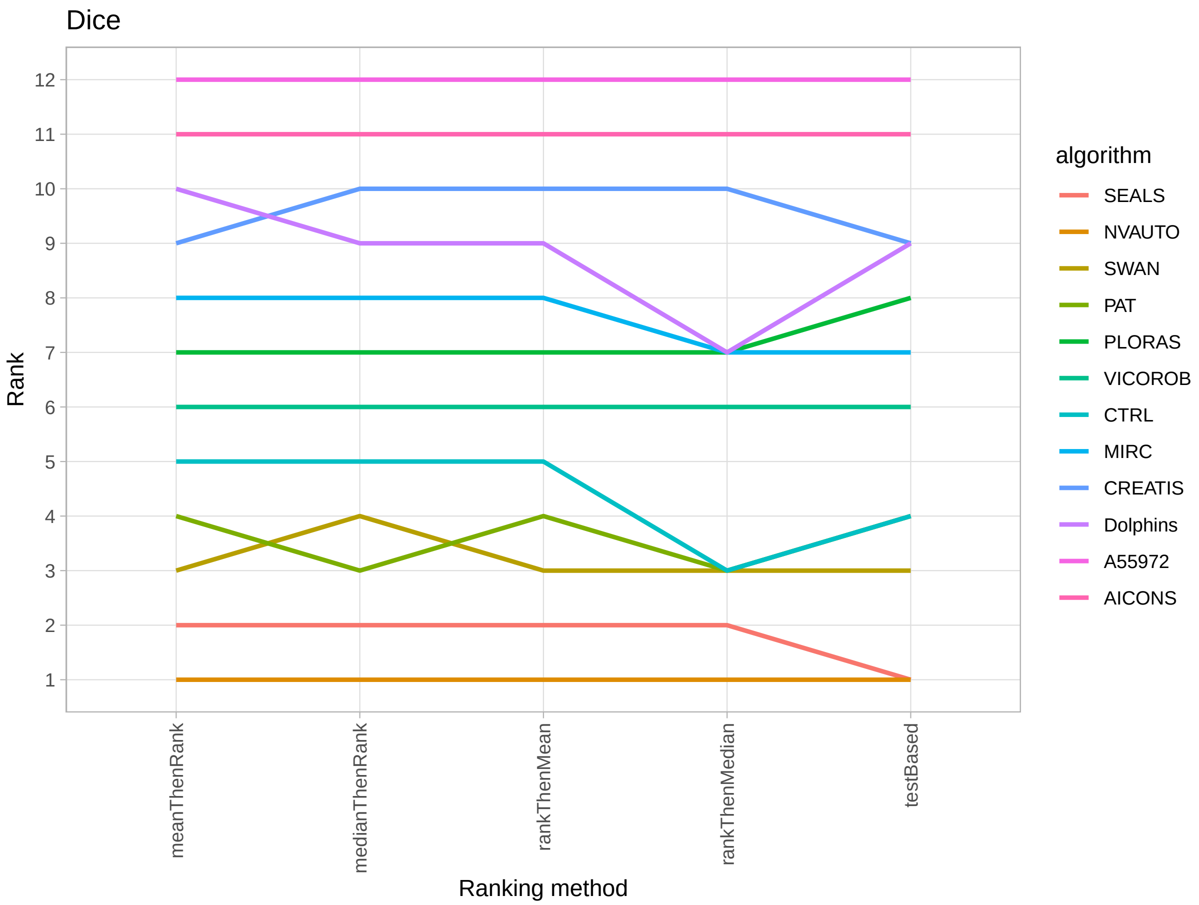

Figure S4.23: Dice Ranking Robustness Across Methods.

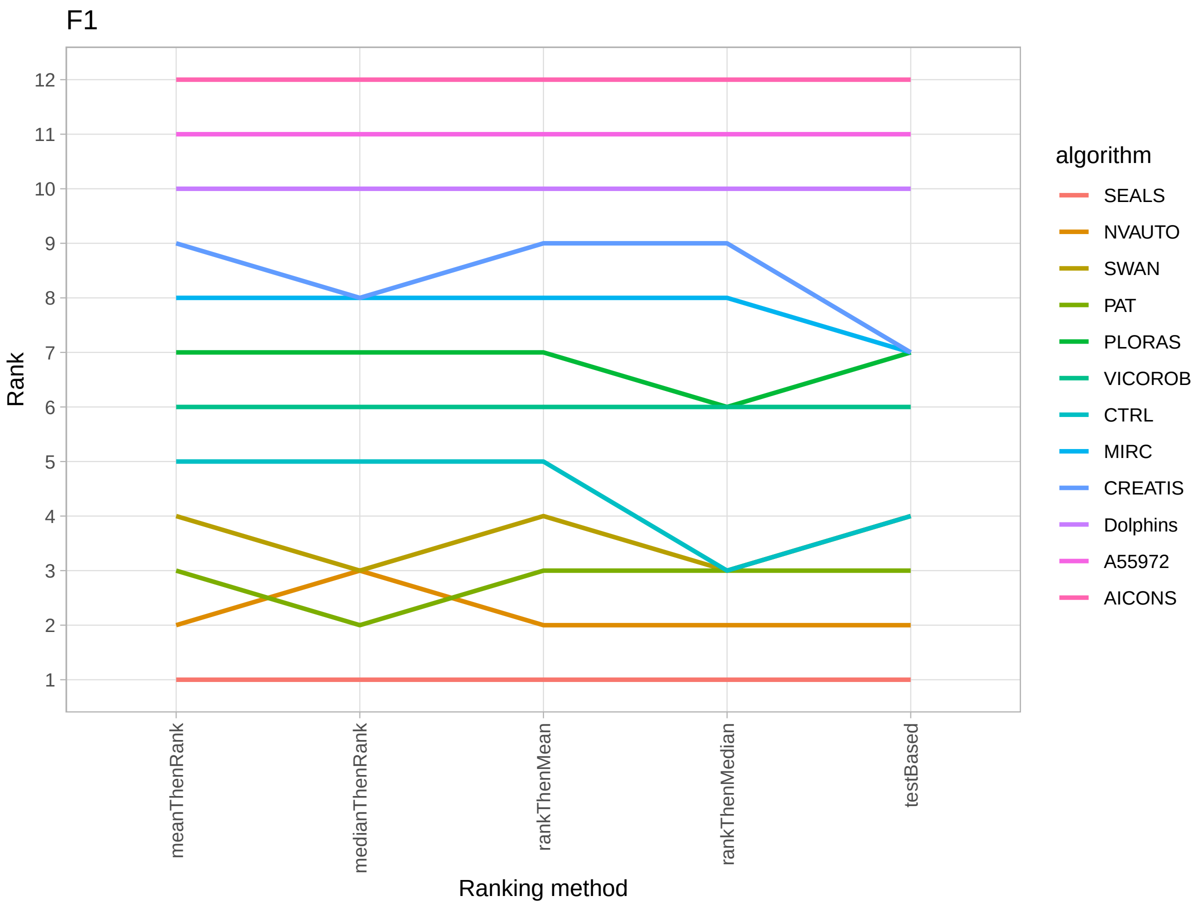

Figure S4.24: F1 Ranking Robustness Across Methods.

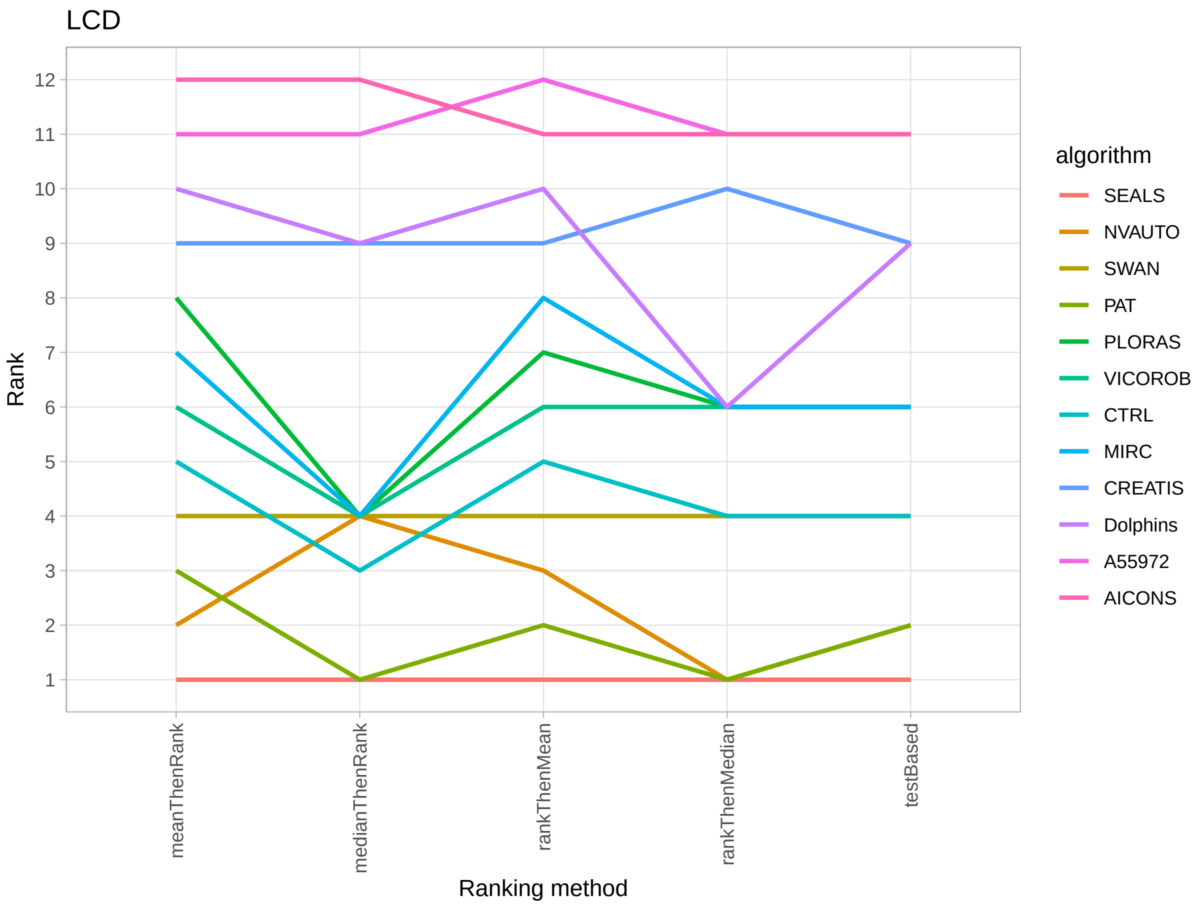

Figure S4.25: LCD Ranking Robustness Across Methods.

4 Visualization of cross-task insights

The algorithms are ordered according to consensus ranking.

4.1 Characterization of algorithms

4.1.1 Ranking stability: Variability of achieved rankings across tasks

Algorithms are color-coded, and the area of each blob at position $(A_i, \text{rank } j)$ is proportional to the relative frequency A_i achieved rank j across multiple tasks. The median rank for each algorithm is indicated by a black cross. This way, the distribution of ranks across tasks can be intuitively visualized.

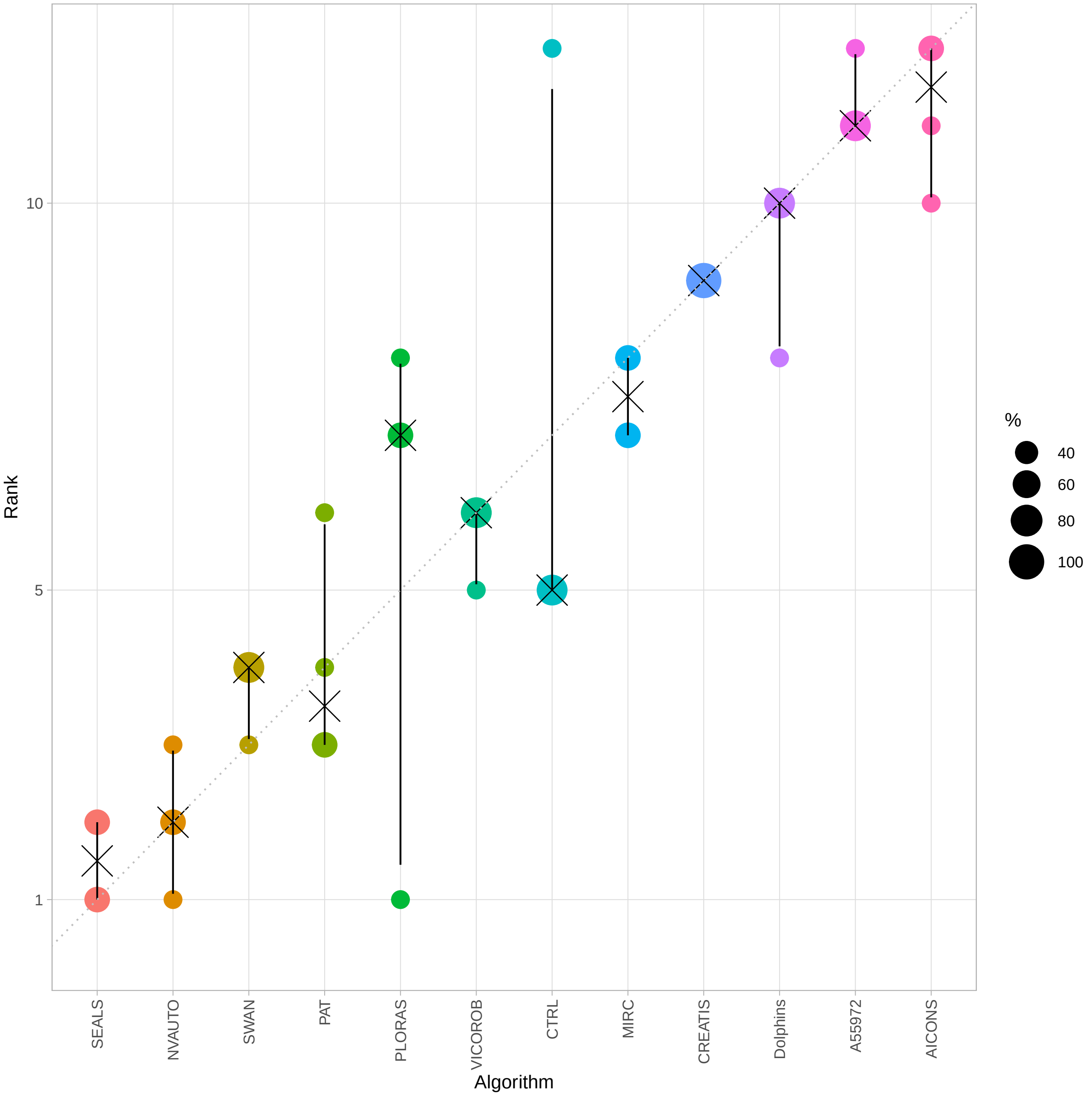

Figure S4.26: Cross-Task Ranking Stability – Blob Plot.

4.1.2 Ranking stability: Ranking variability via bootstrap approach

A blob plot of bootstrap results over the different tasks separated by algorithm allows another perspective on the assessment data. This gives deeper insights into the characteristics of tasks and the ranking uncertainty of the algorithms in each task.

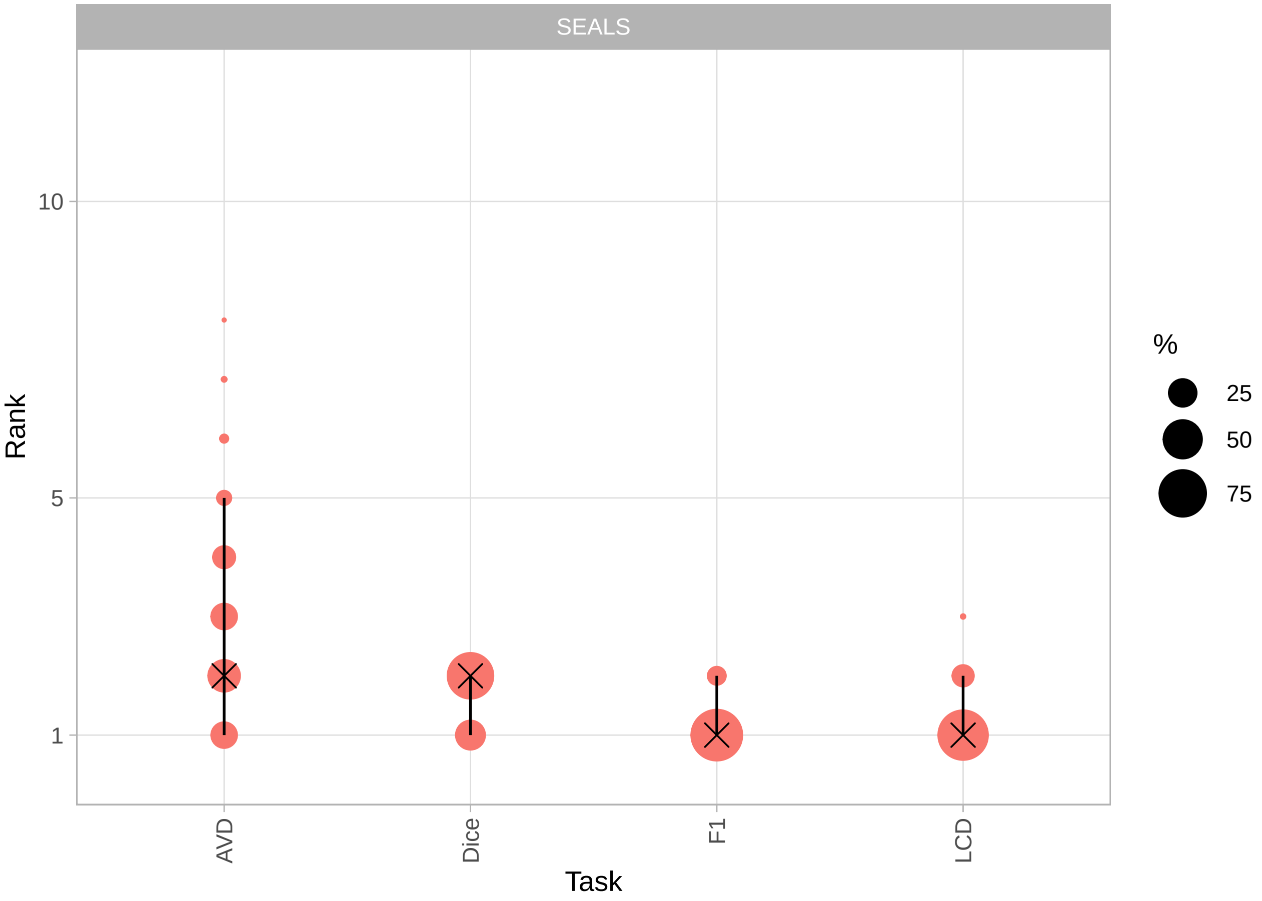

Figure S4.27: Bootstrap Ranking Stability – SEALS.

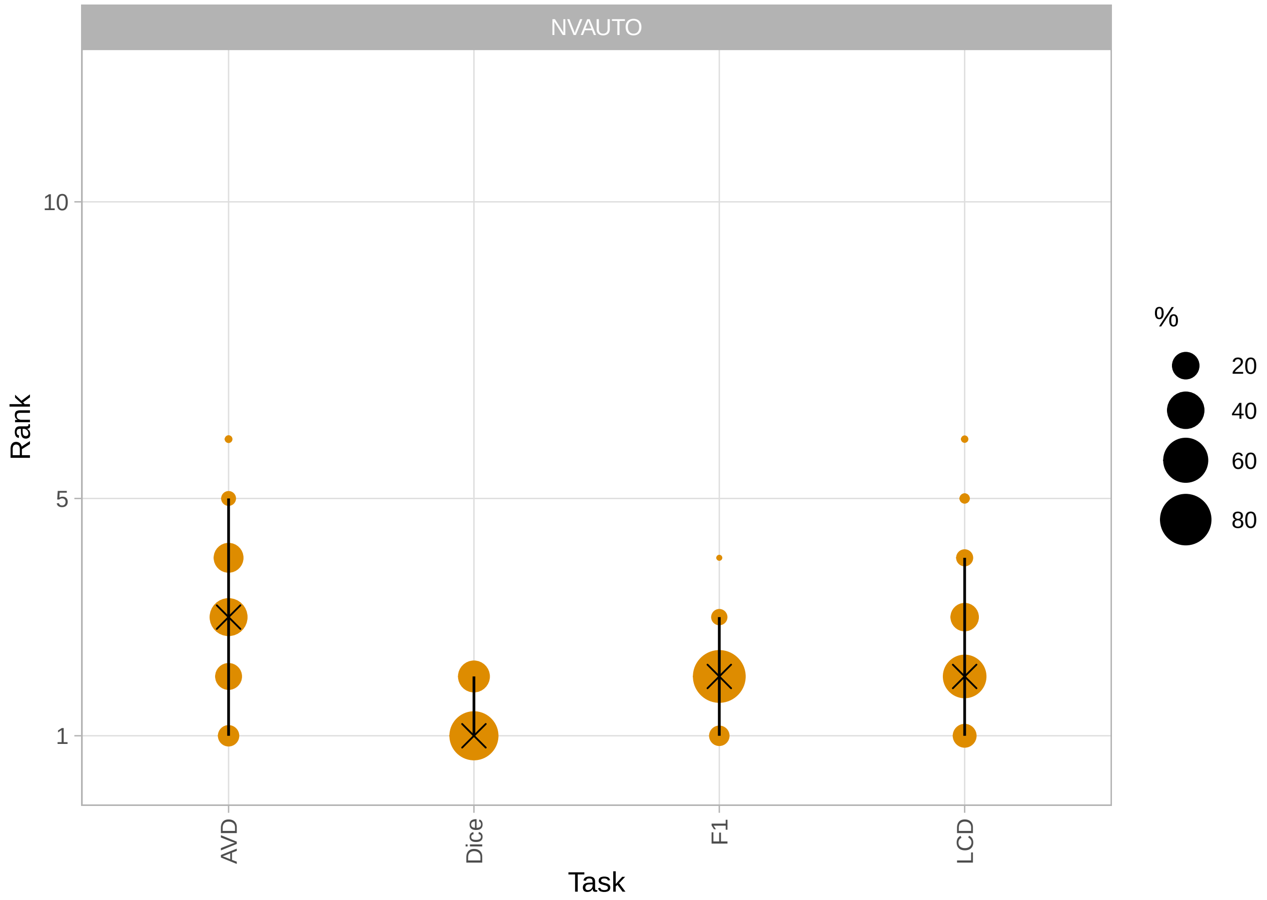

Figure S4.28: Bootstrap Ranking Stability – NVAUTO.

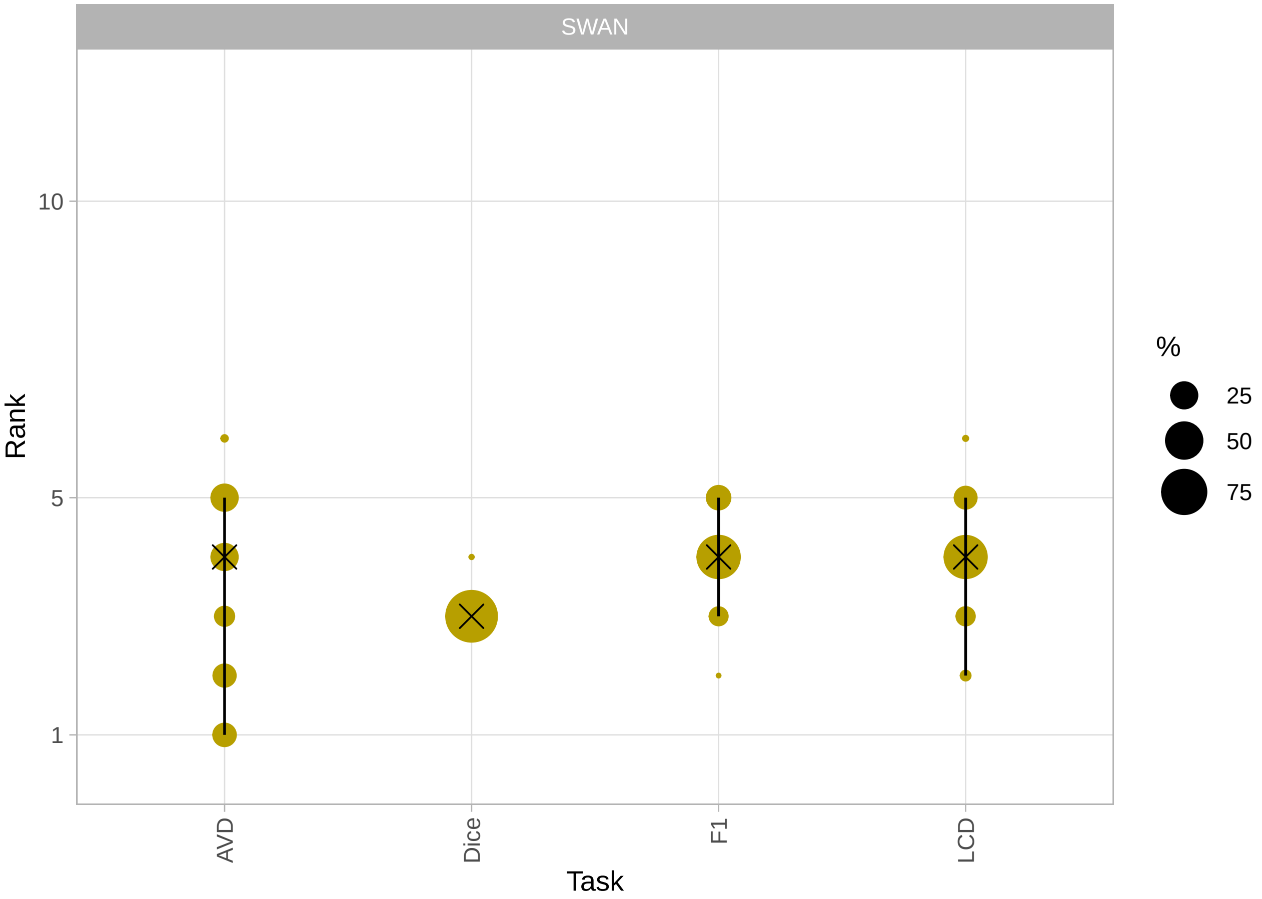

Figure S4.29: Bootstrap Ranking Stability – SWAN.

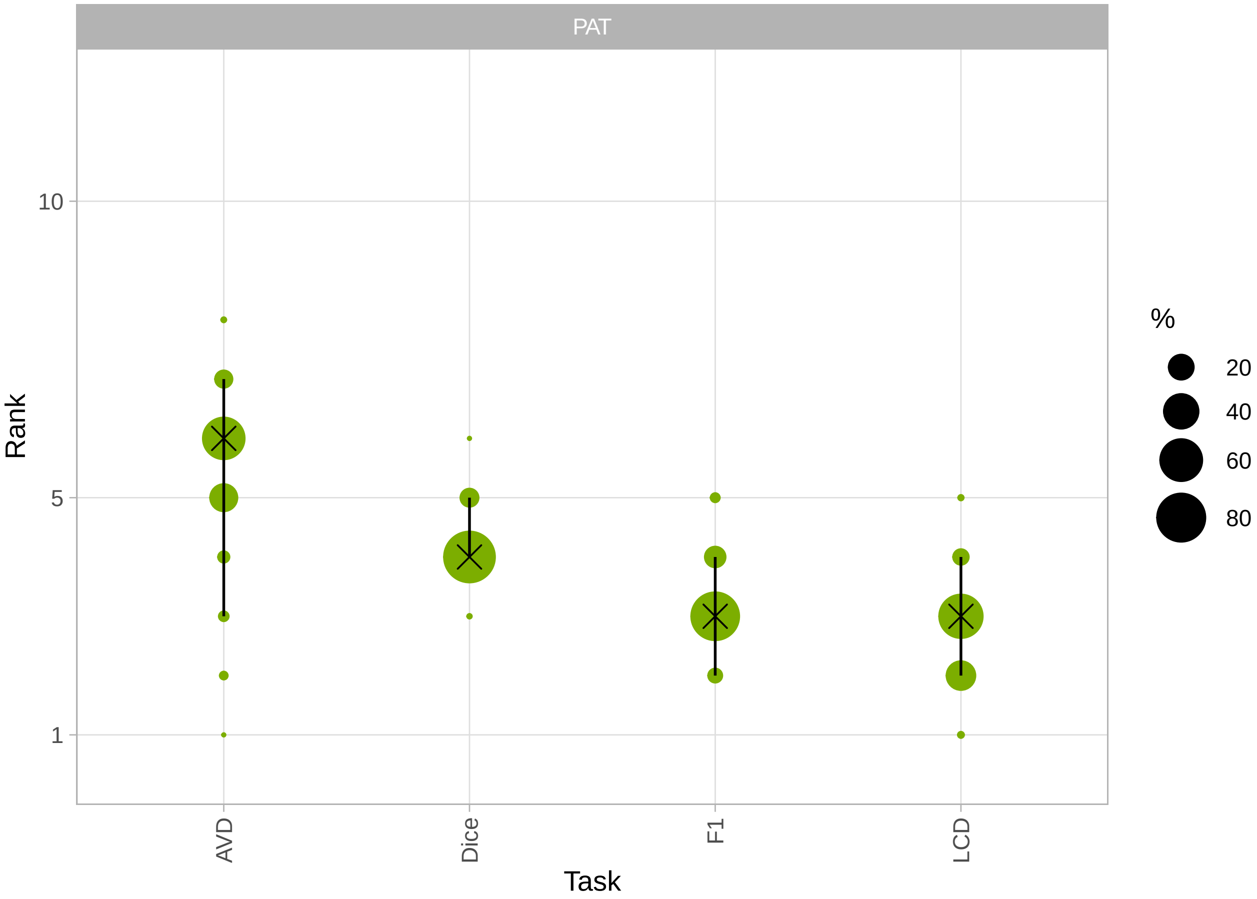

Figure S4.30: Bootstrap Ranking Stability – PAT.

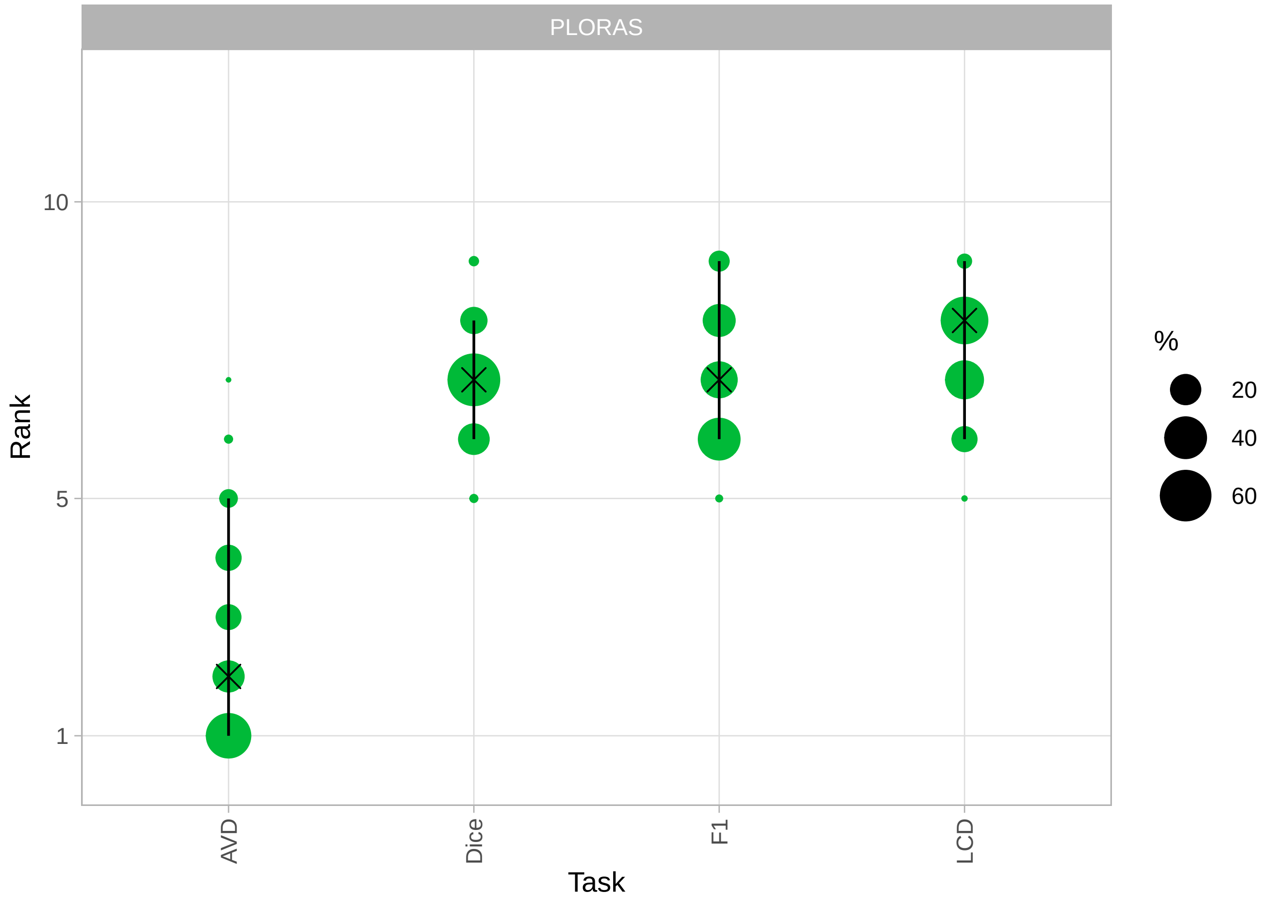

Figure S4.31: Bootstrap Ranking Stability – PLORAS.

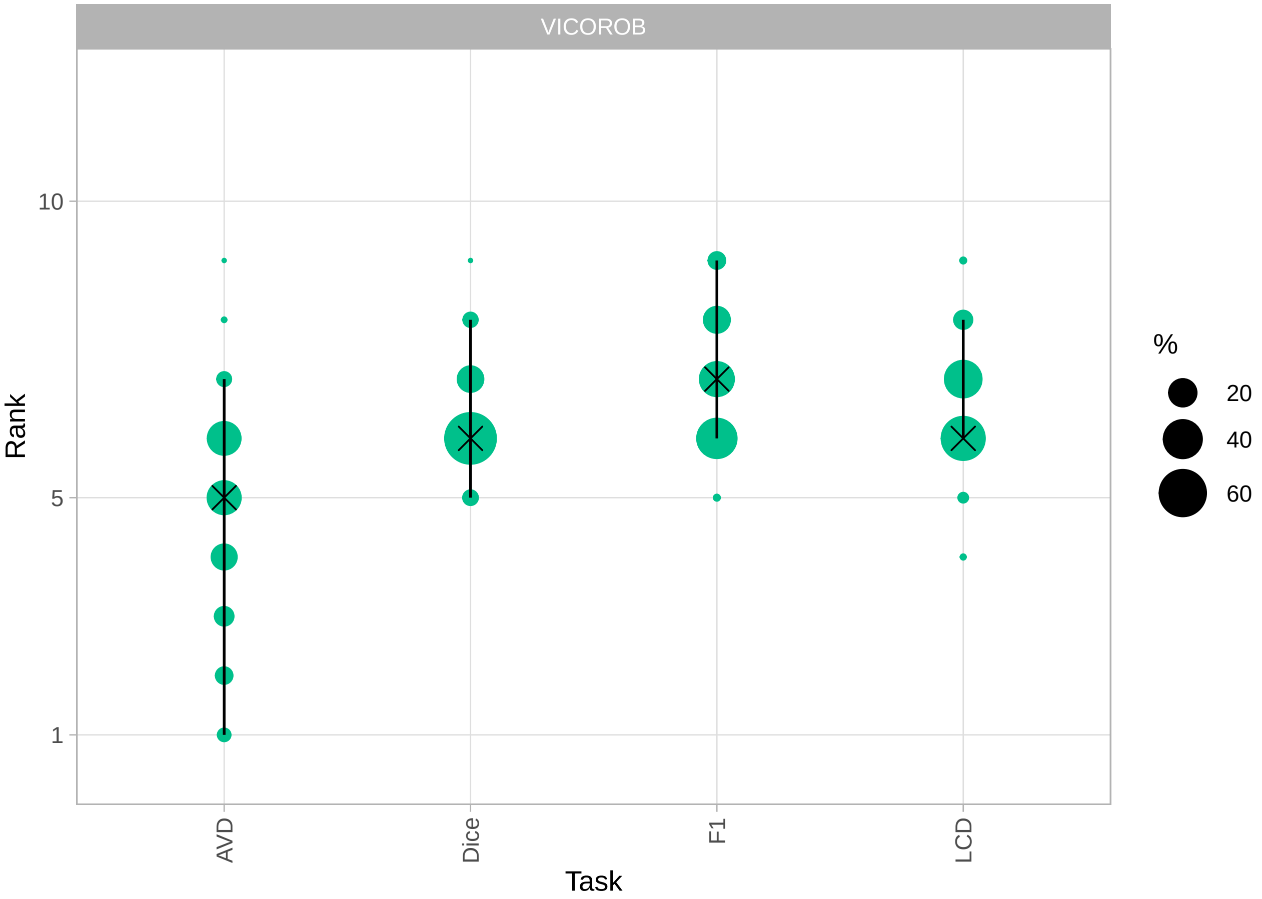

Figure S4.32: Bootstrap Ranking Stability – VICOROB.

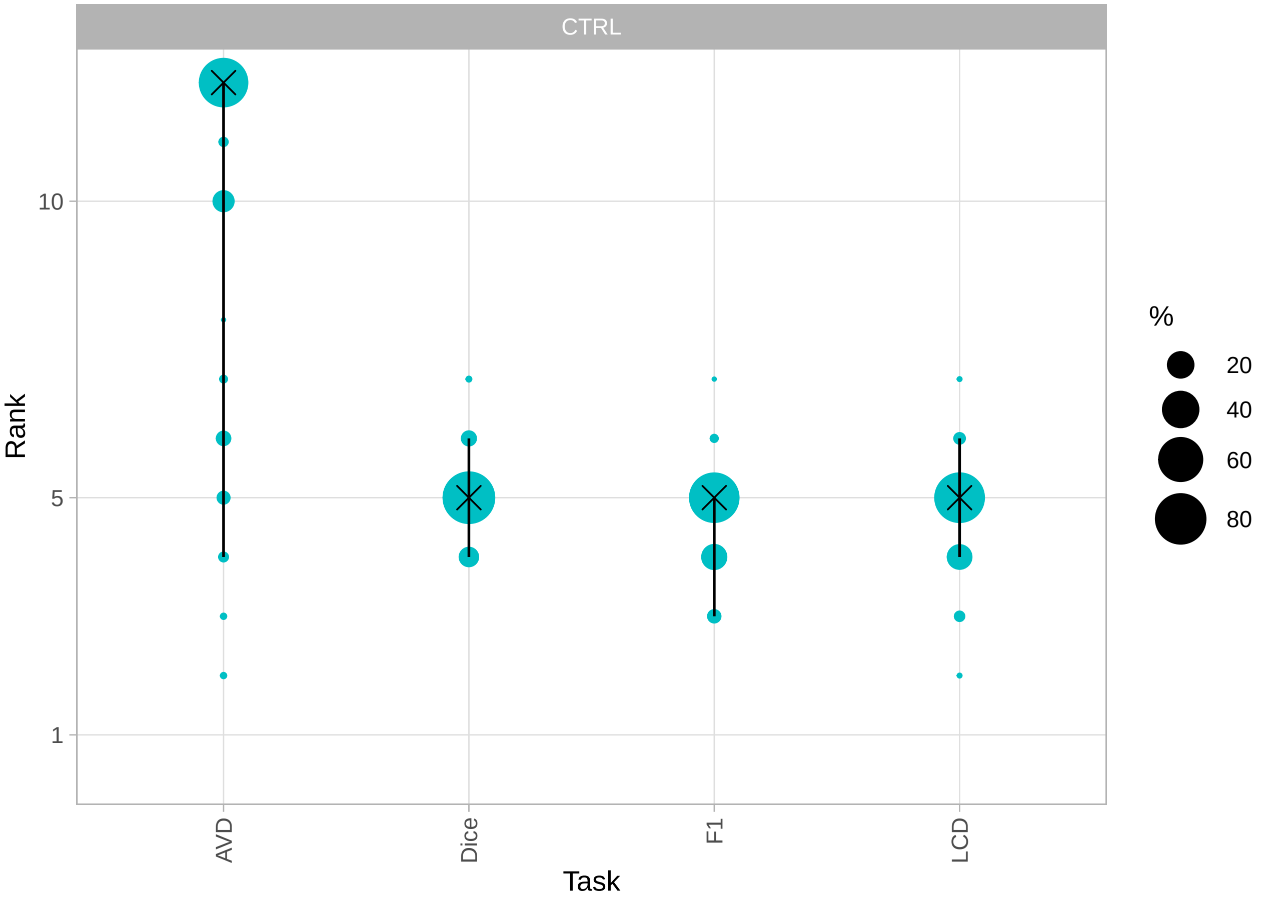

Figure S4.33: Bootstrap Ranking Stability – CTRL.

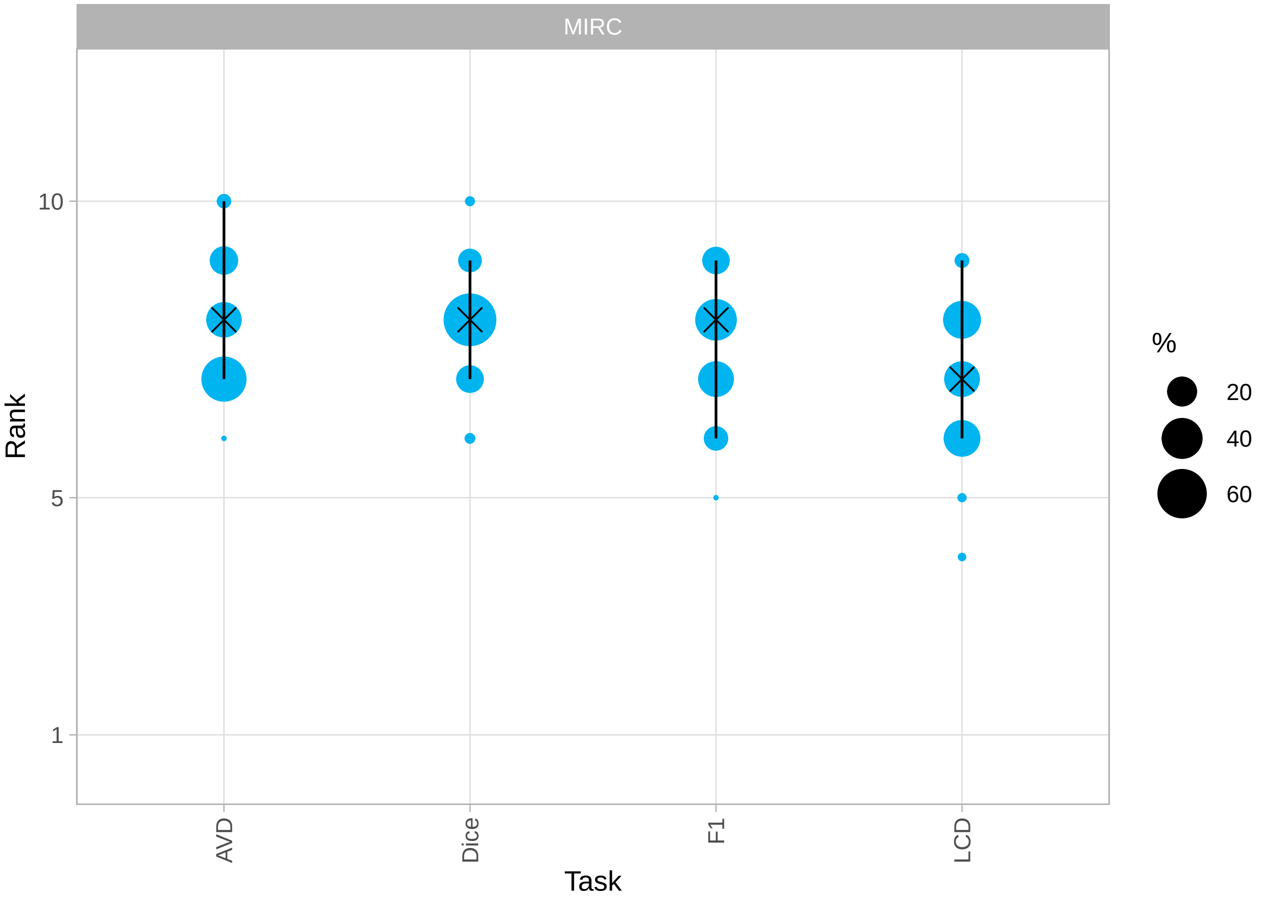

Figure S4.34: Bootstrap Ranking Stability – MIRC.

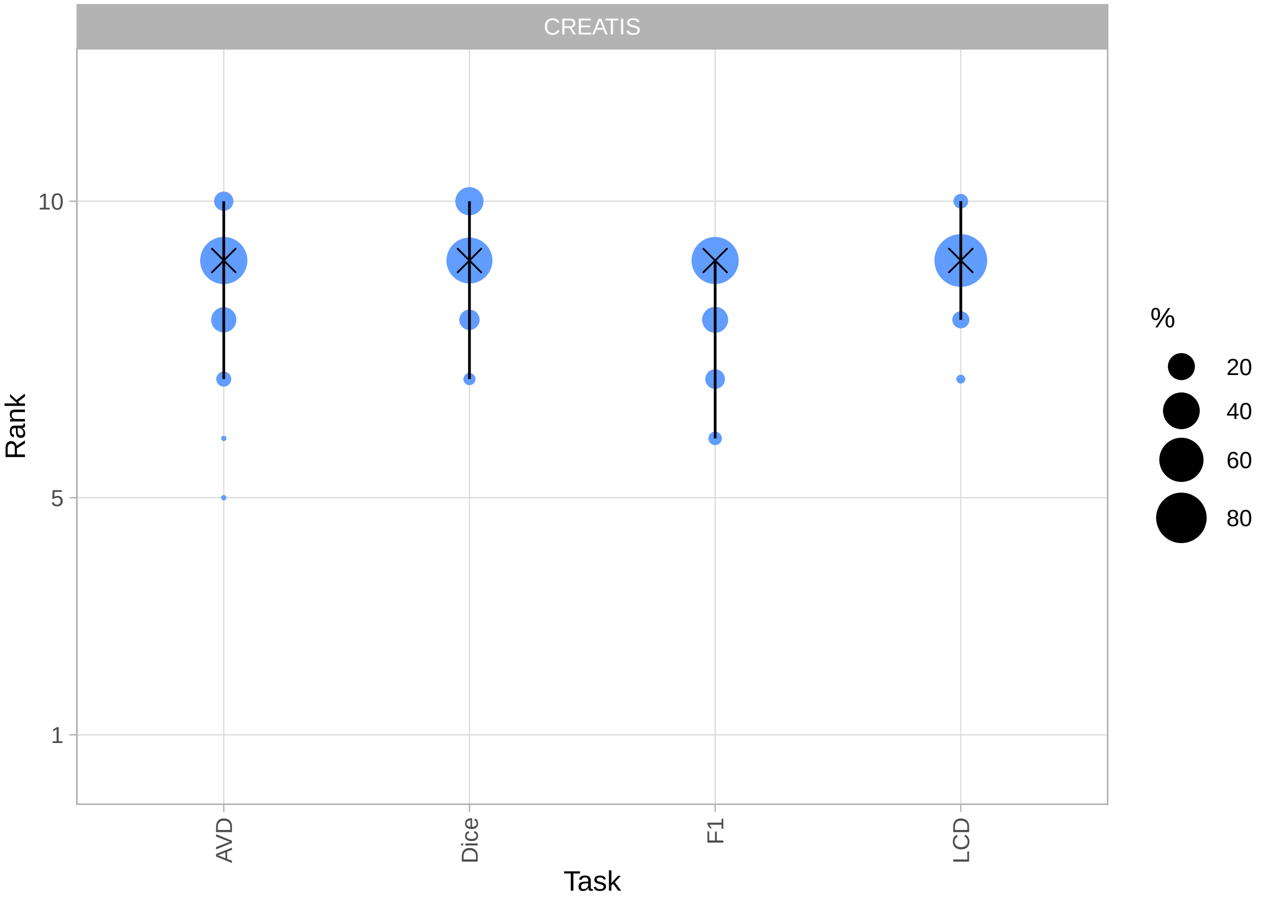

Figure S4.35: Bootstrap Ranking Stability – CREATIS.

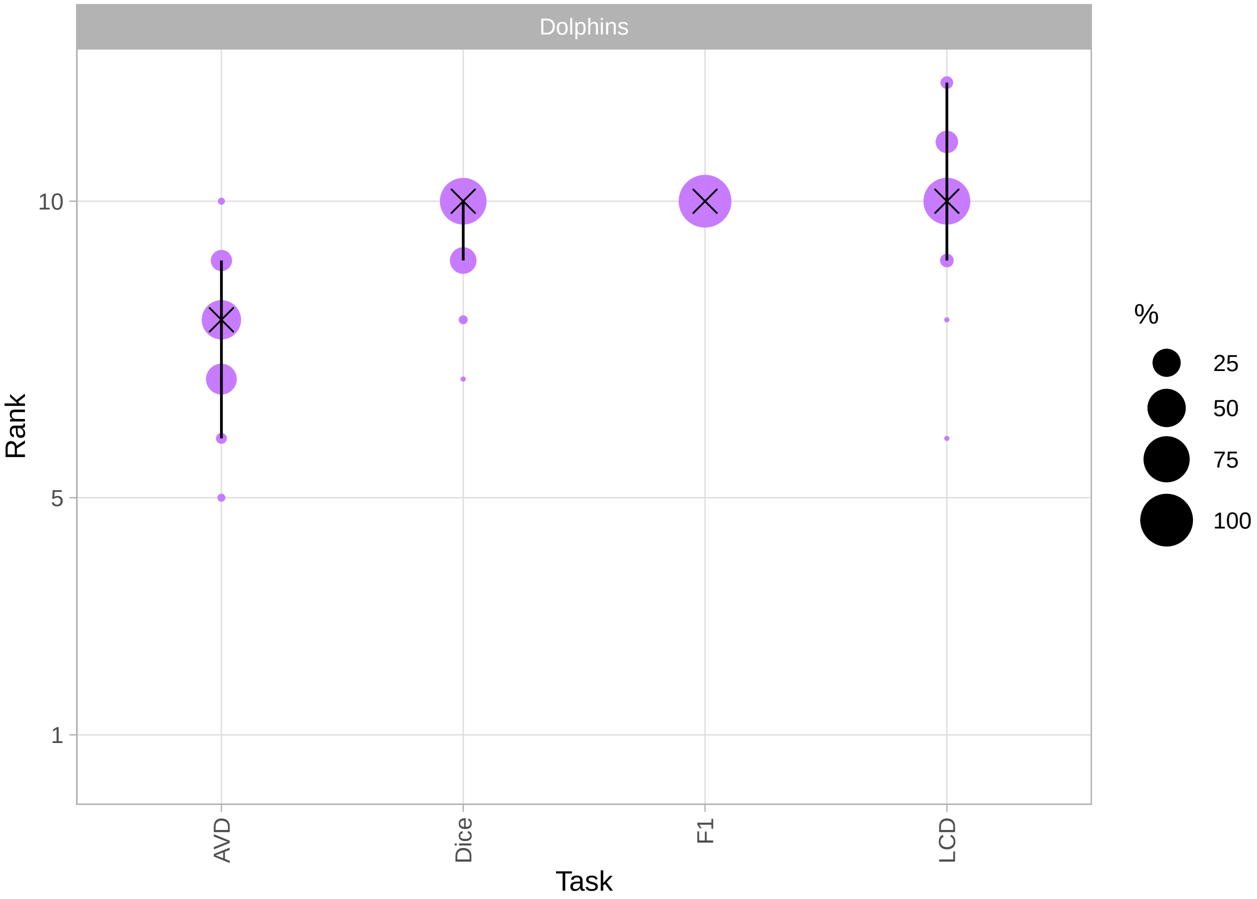

Figure S4.36: Bootstrap Ranking Stability – Dolphins.

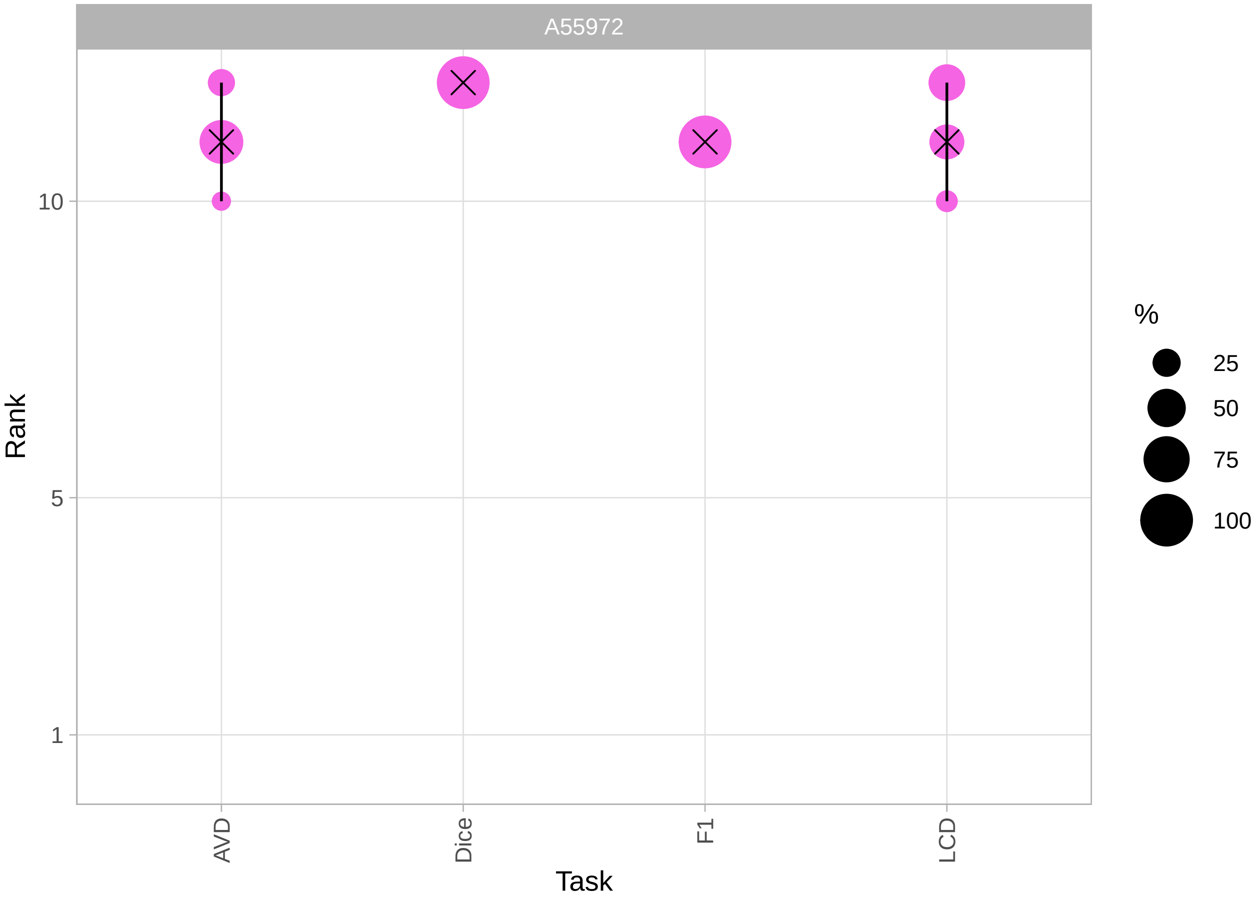

Figure S4.37: Bootstrap Ranking Stability – A55972.

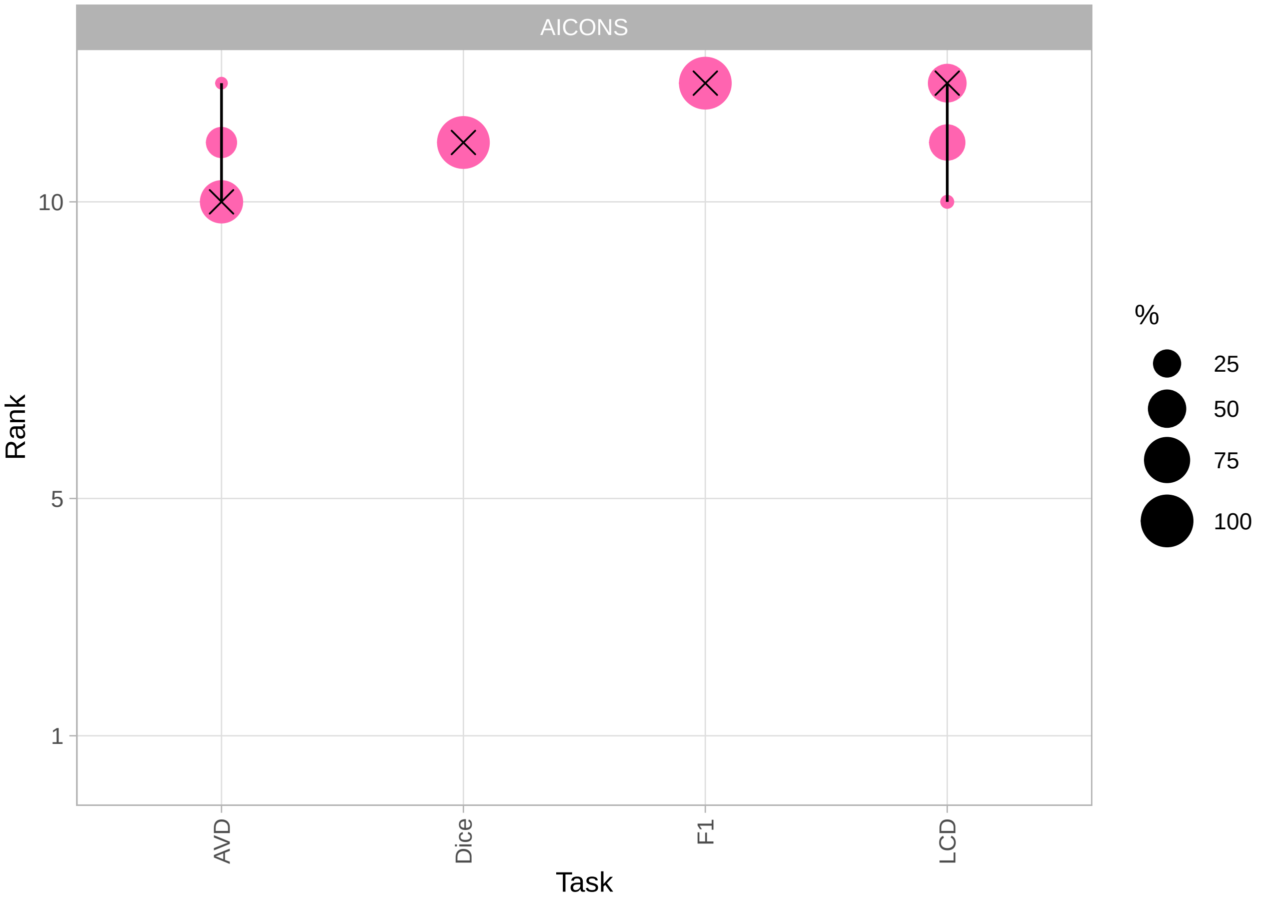

Figure S4.38: Bootstrap Ranking Stability – AICONS.

An alternative representation is provided by a stacked frequency plot of the observed ranks, separated by algorithm. Observed ranks across bootstrap samples are displayed with coloring according to the task. For algorithms that achieve the same rank in different tasks for the full assessment data set, vertical lines are on top of each other. Vertical lines allow to compare the achieved rank of each algorithm over different tasks.

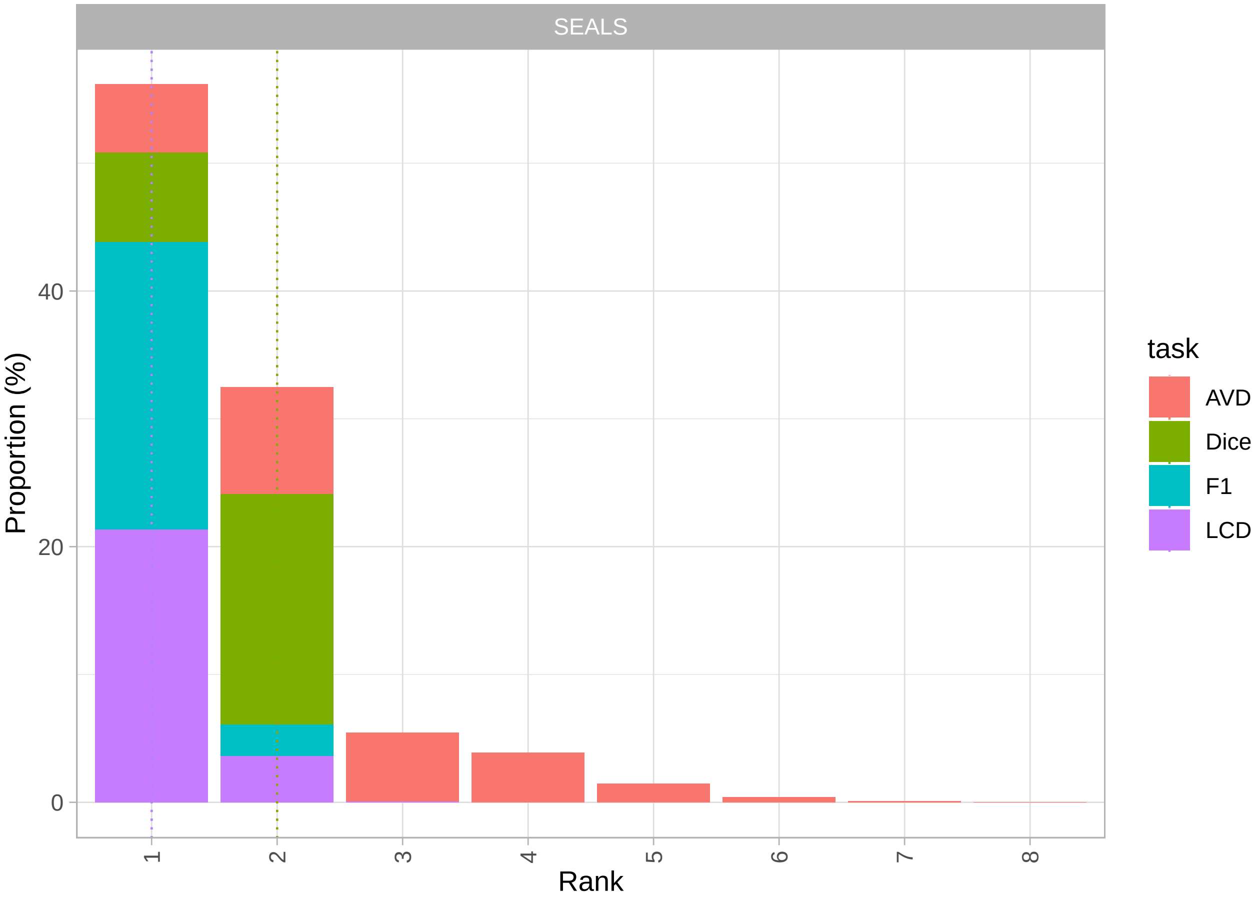

Figure S4.39: Stacked Rank Frequencies – SEALS.

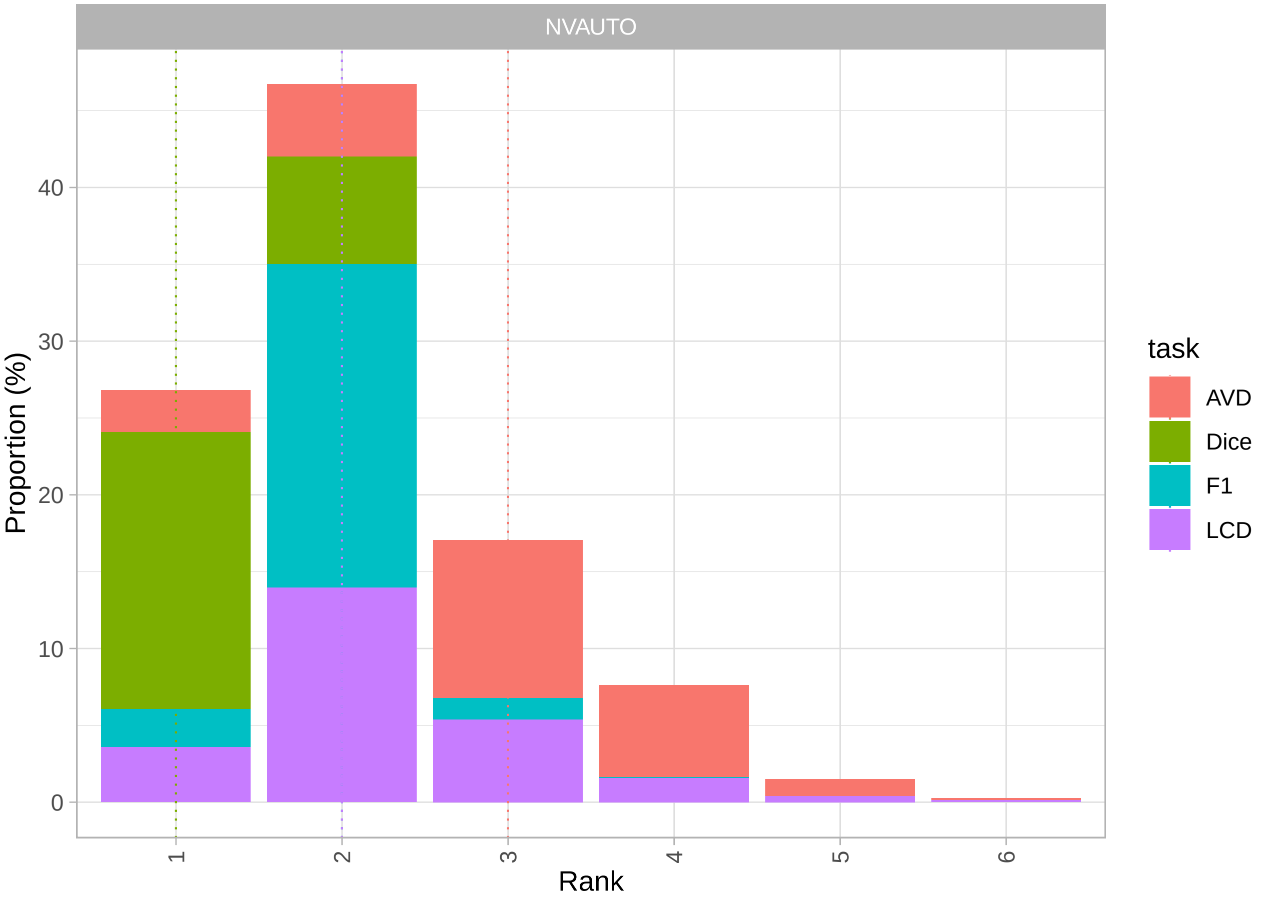

Figure S4.40: Stacked Rank Frequencies – NVAUTO.

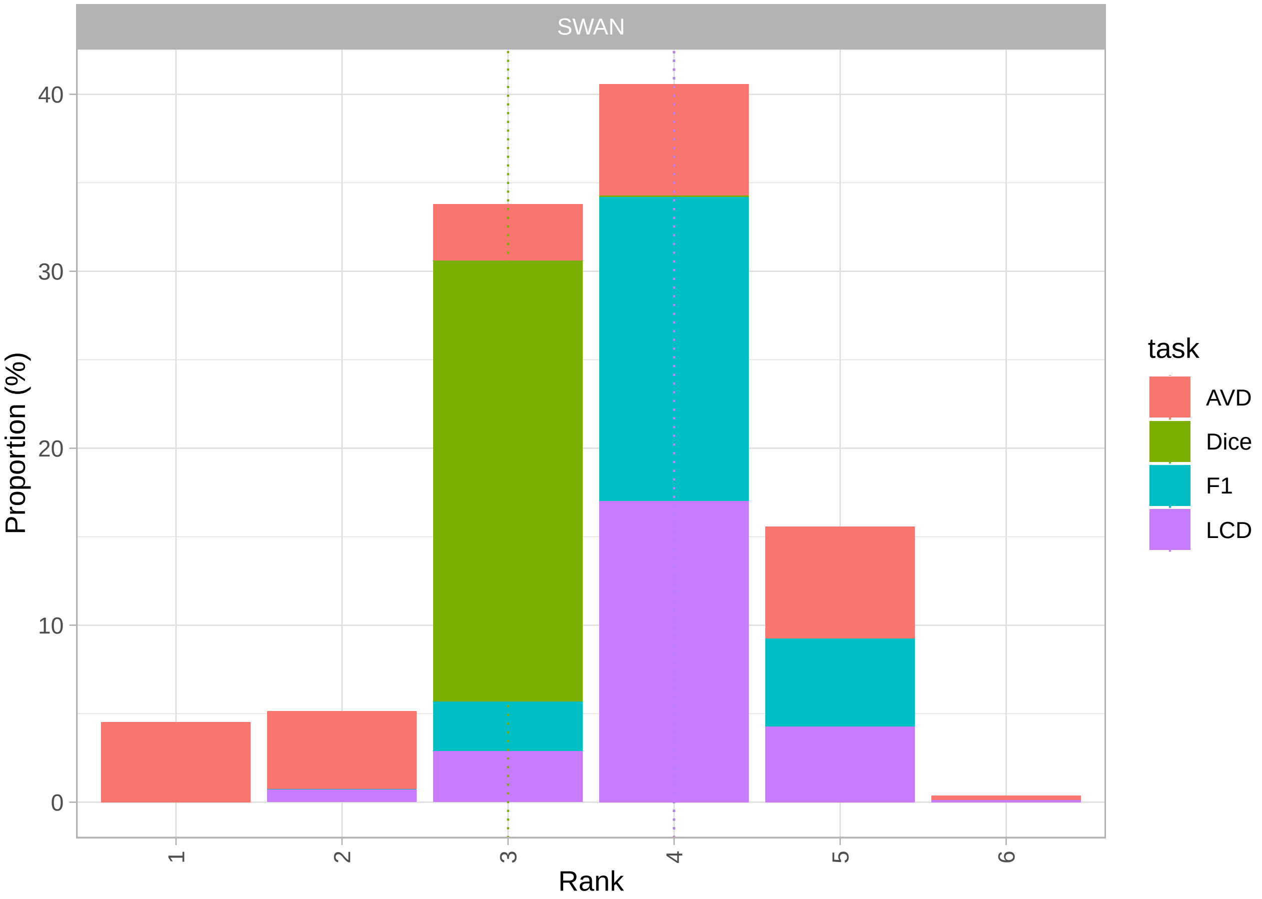

Figure S4.41: Stacked Rank Frequencies – SWAN.

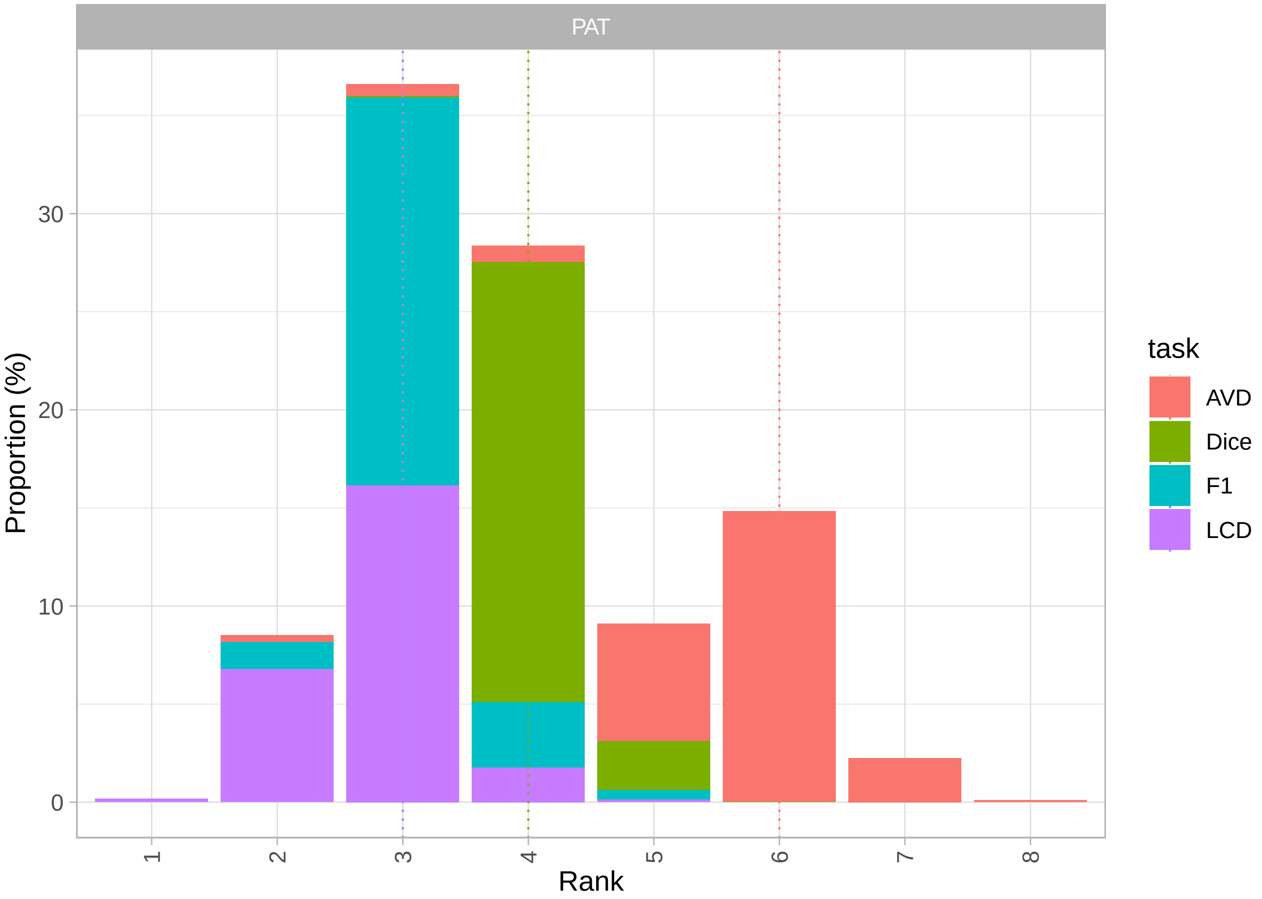

Figure S4.42: Stacked Rank Frequencies – PAT.

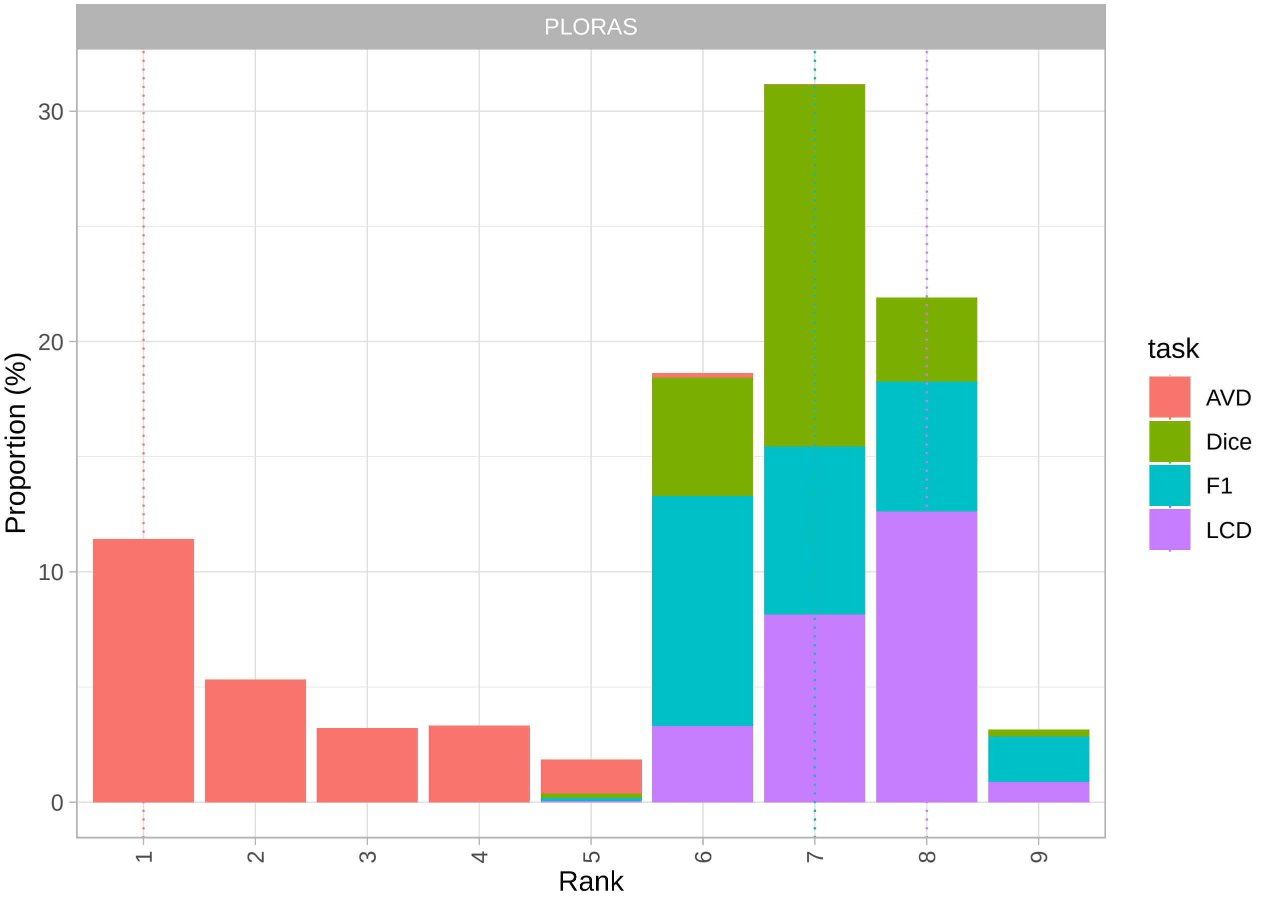

Figure S4.43: Stacked Rank Frequencies – PLORAS.

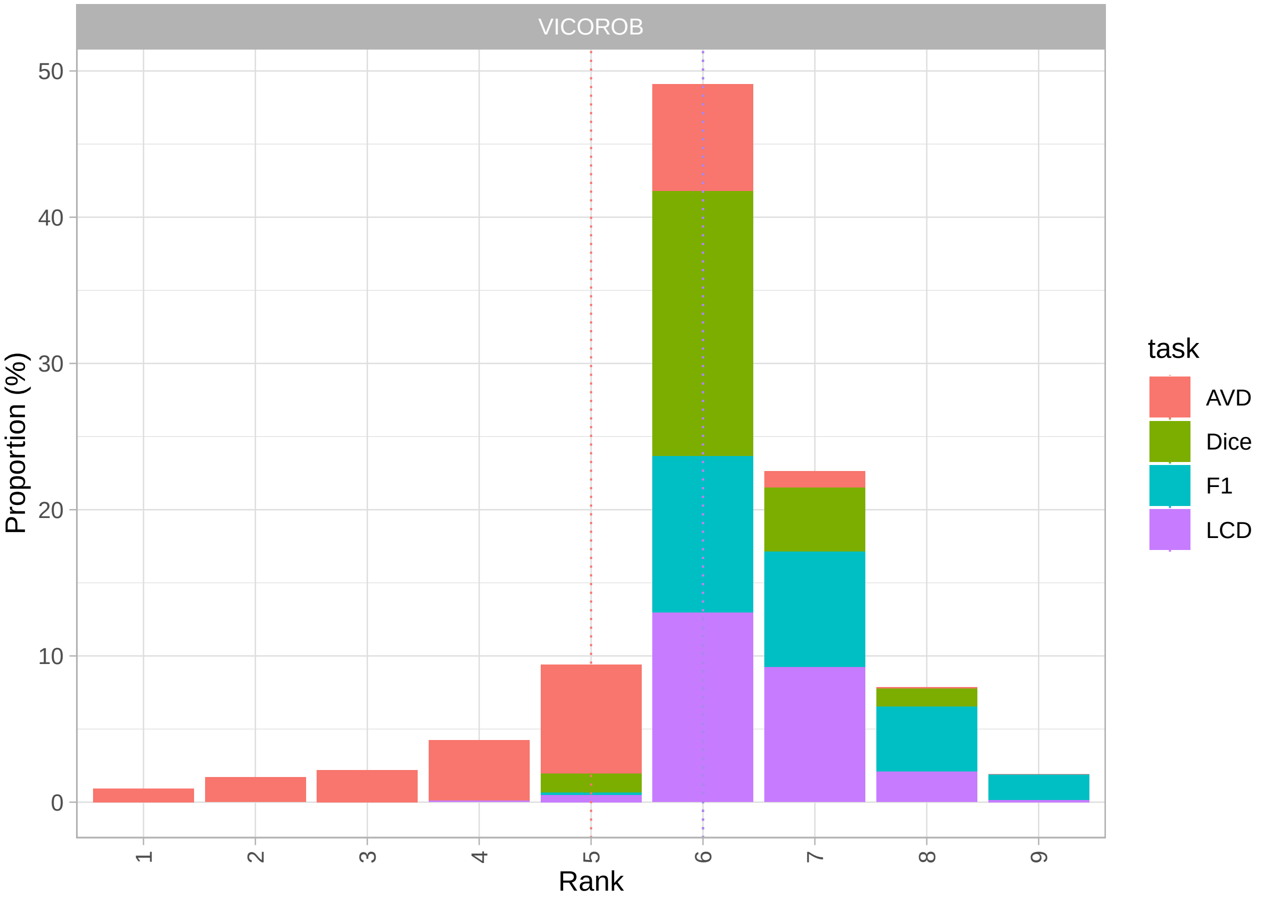

Figure S4.44: Stacked Rank Frequencies – VICOROB.

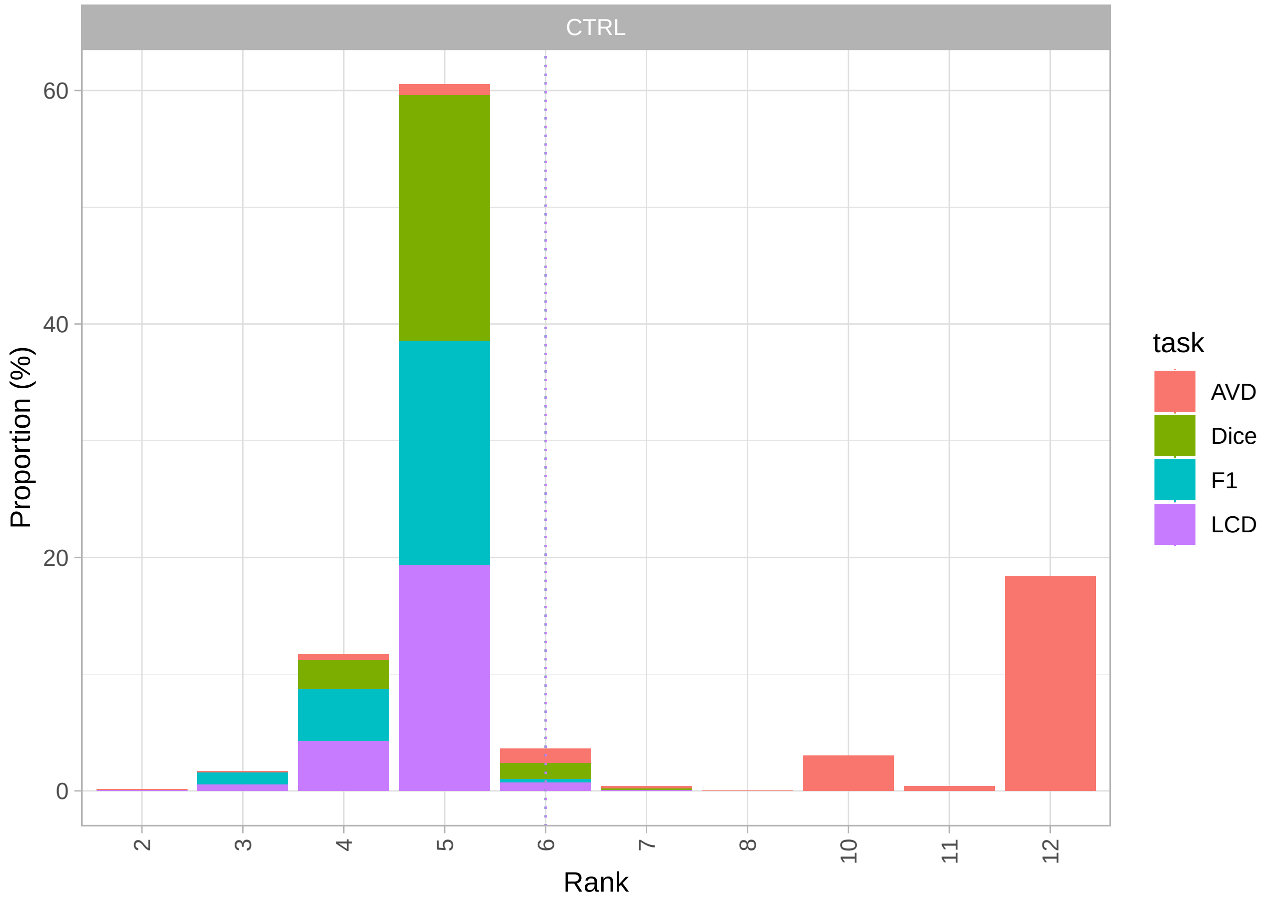

Figure S4.45: Stacked Rank Frequencies – CTRL.

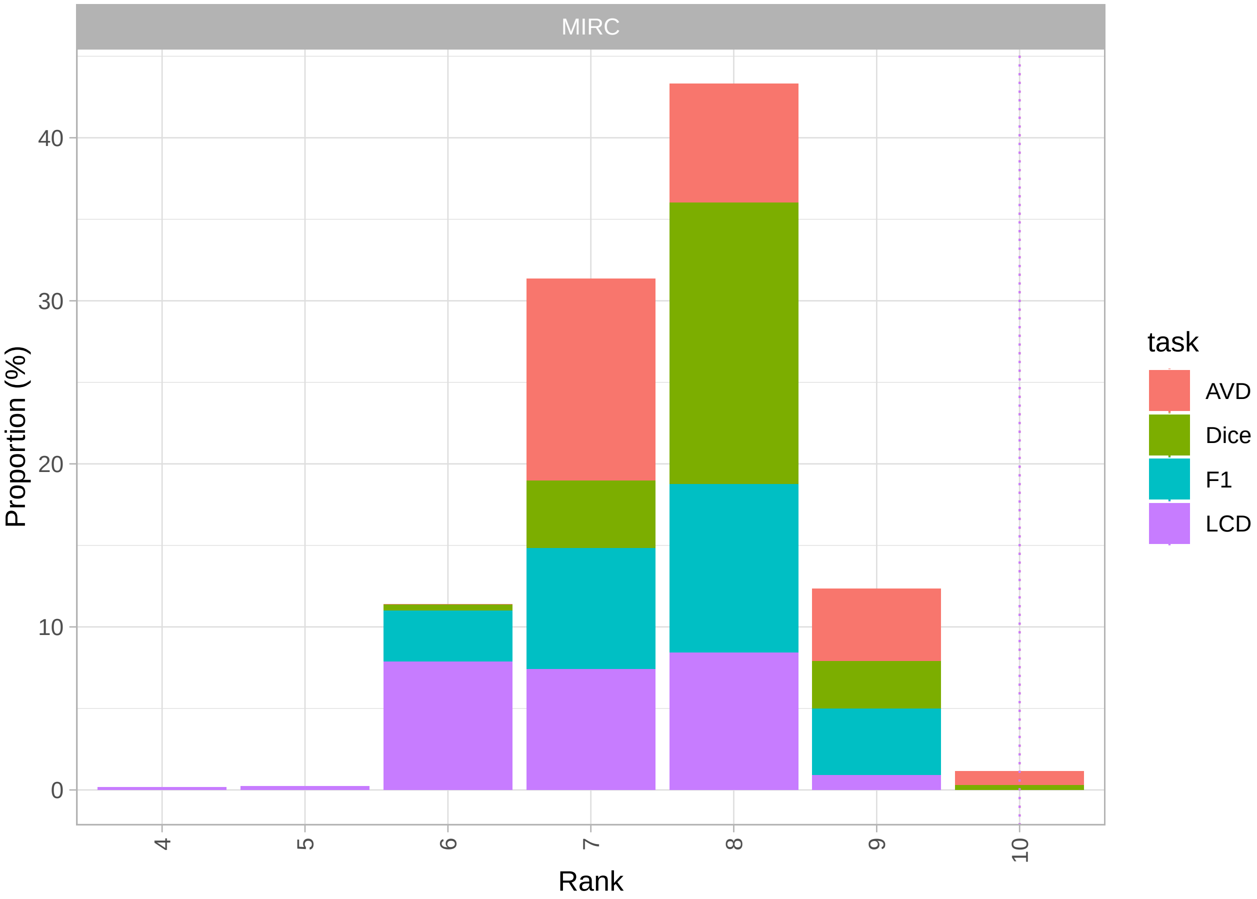

Figure S4.46: Stacked Rank Frequencies – MIRC.

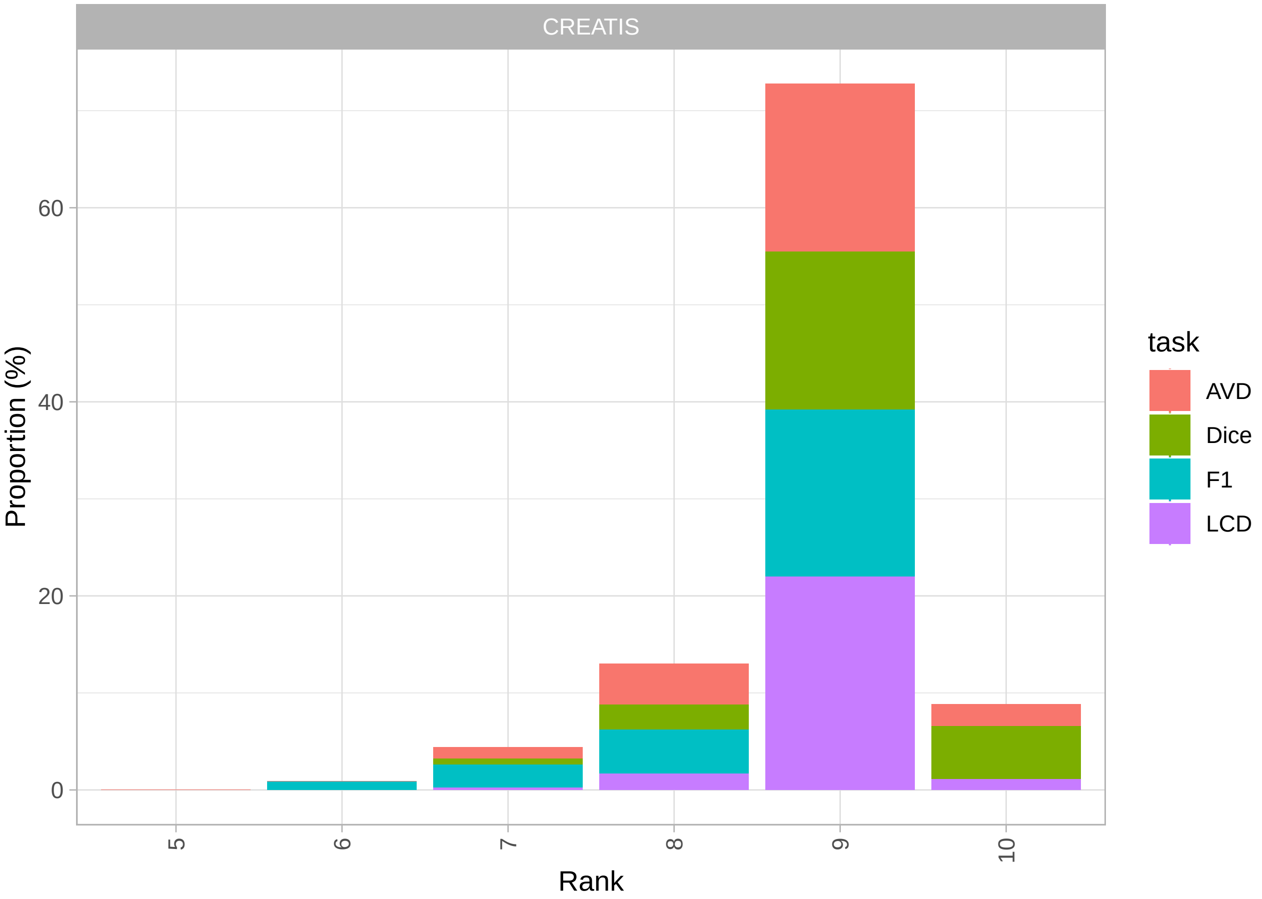

Figure S4.47: Stacked Rank Frequencies – CREATIS.

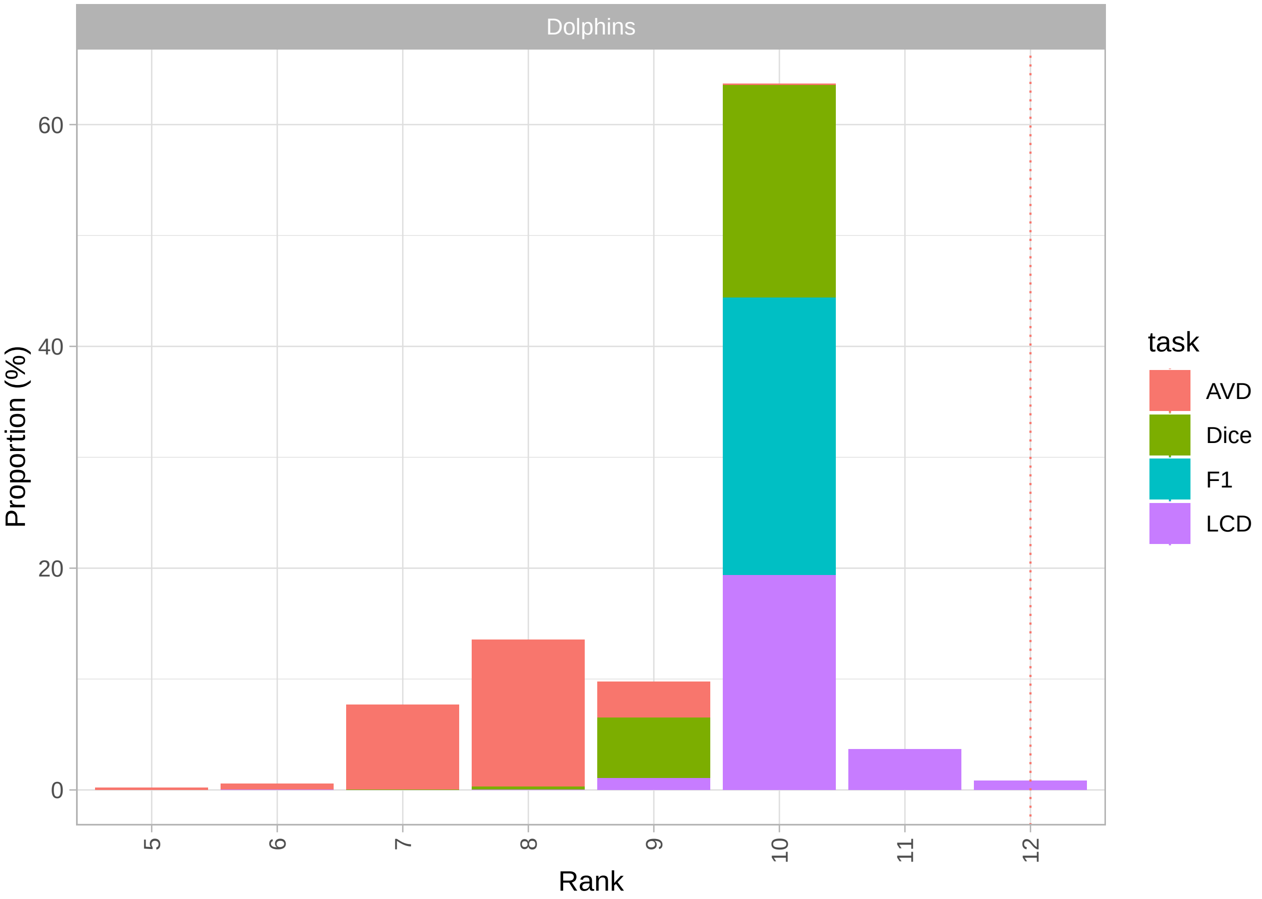

Figure S4.48: Stacked Rank Frequencies – Dolphins.

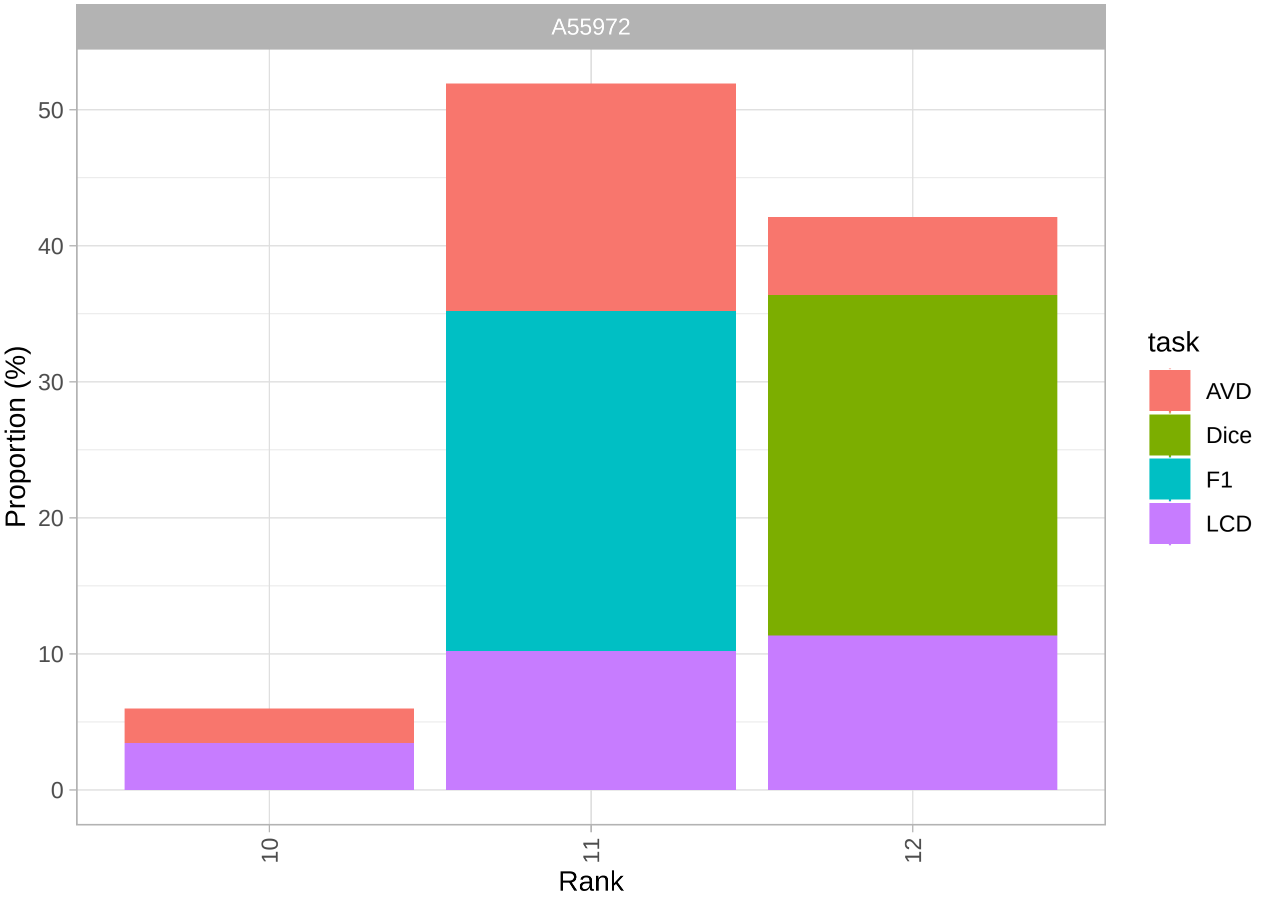

Figure S4.49: Stacked Rank Frequencies – A55972.

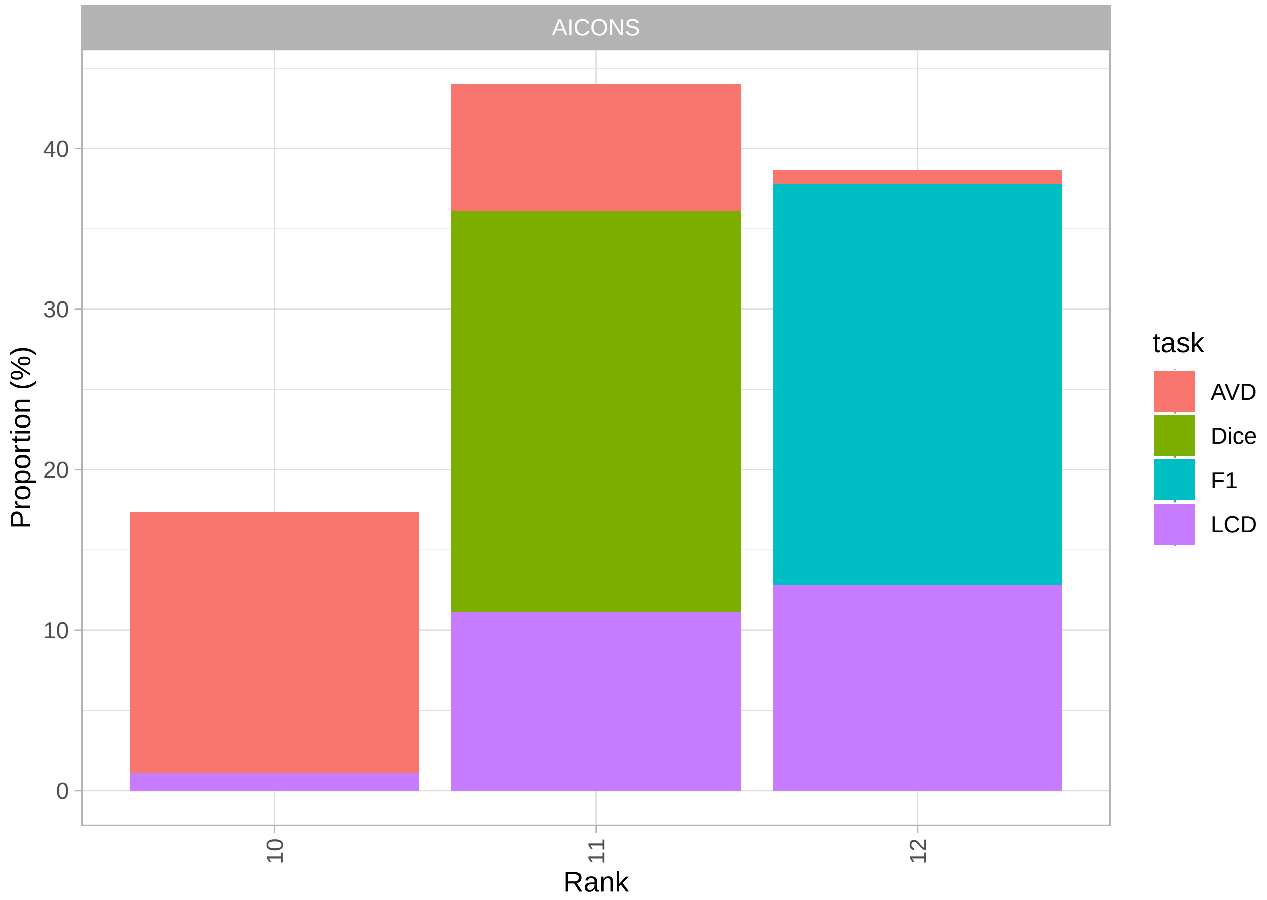

Figure S4.50: Stacked Rank Frequencies – AICONS.

4.2 Characterization of tasks

4.2.1 Visualizing bootstrap results

To investigate which tasks separate algorithms well (i.e., lead to a stable ranking), a blob plot is recommended.

Bootstrap results can be shown in a blob plot showing one plot for each task. In this view, the spread of the blobs for each algorithm can be compared across tasks. Deviations from the diagonal indicate deviations from the consensus ranking (over tasks). Specifically, if rank distribution of an algorithm is consistently below the diagonal, the algorithm performed better in this task than on average across tasks, while if the rank distribution of an algorithm is consistently above the diagonal, the algorithm performed worse in this task than on average across tasks. At the bottom of each panel, ranks for each algorithm in the tasks are provided.

Same as in Section 3.1 but now ordered according to consensus.

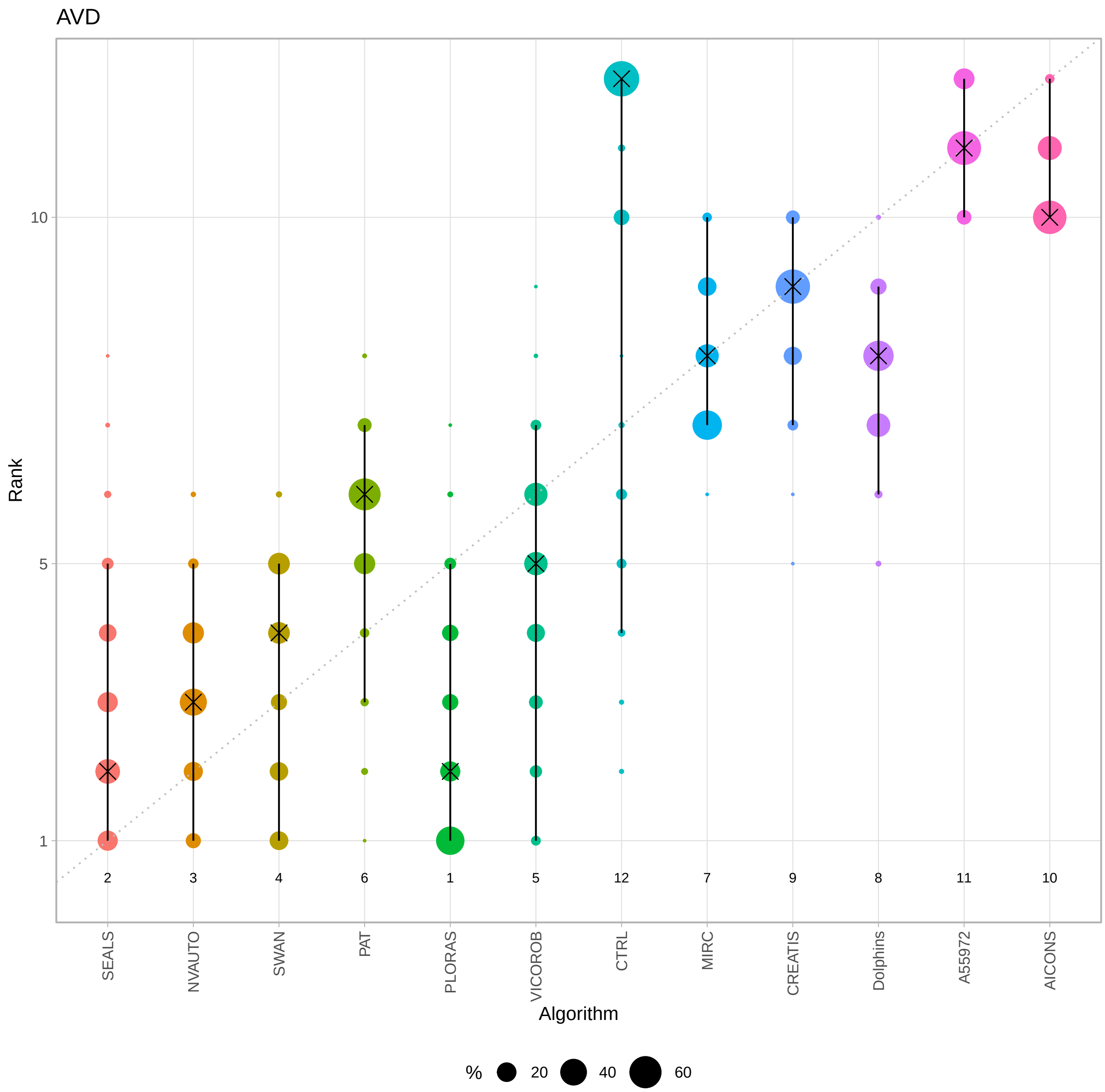

Figure S4.51: AVD Task-Level Ranking Stability – Blob Plot.

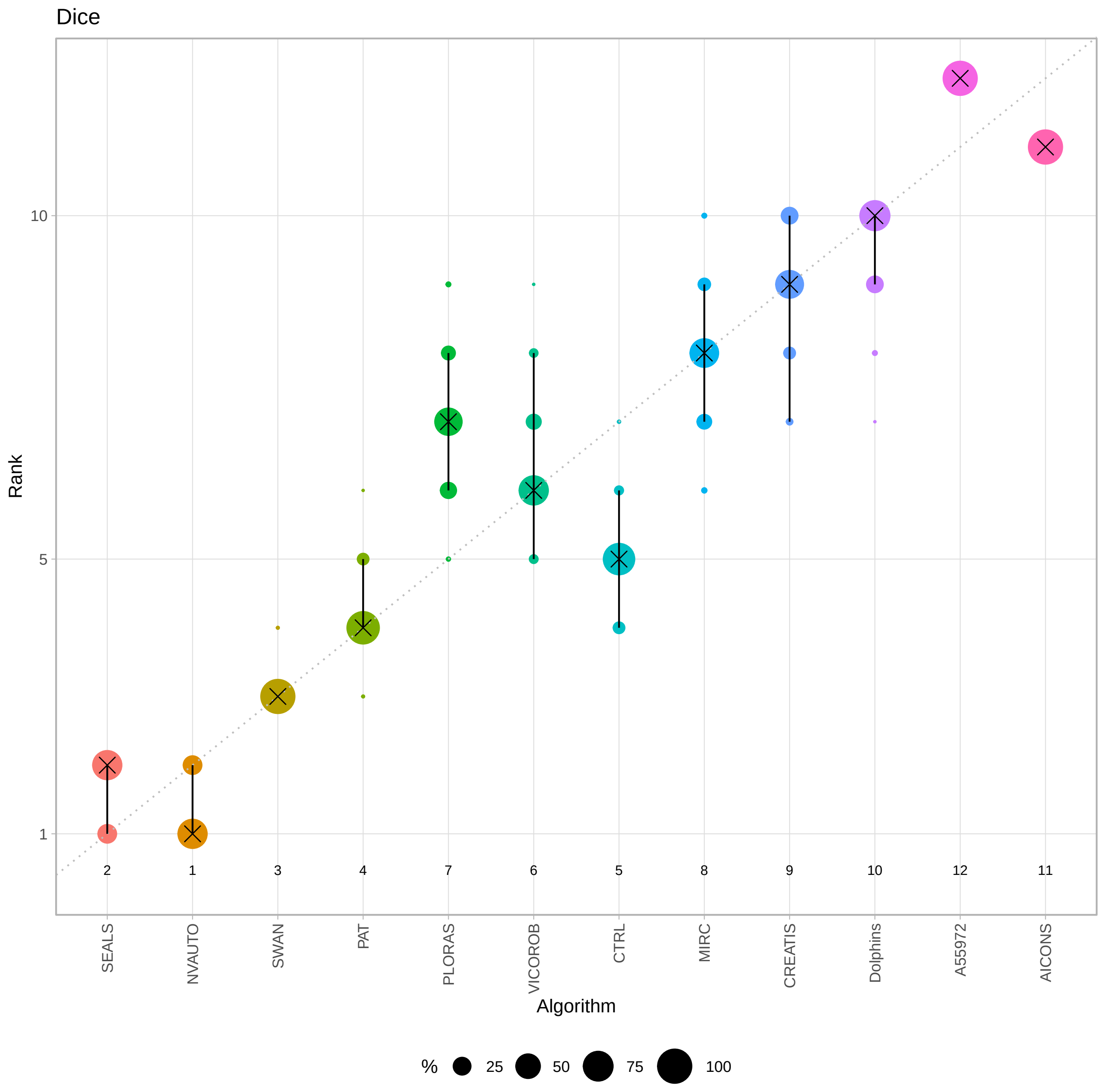

Figure S4.52: Dice Task-Level Ranking Stability – Blob Plot.

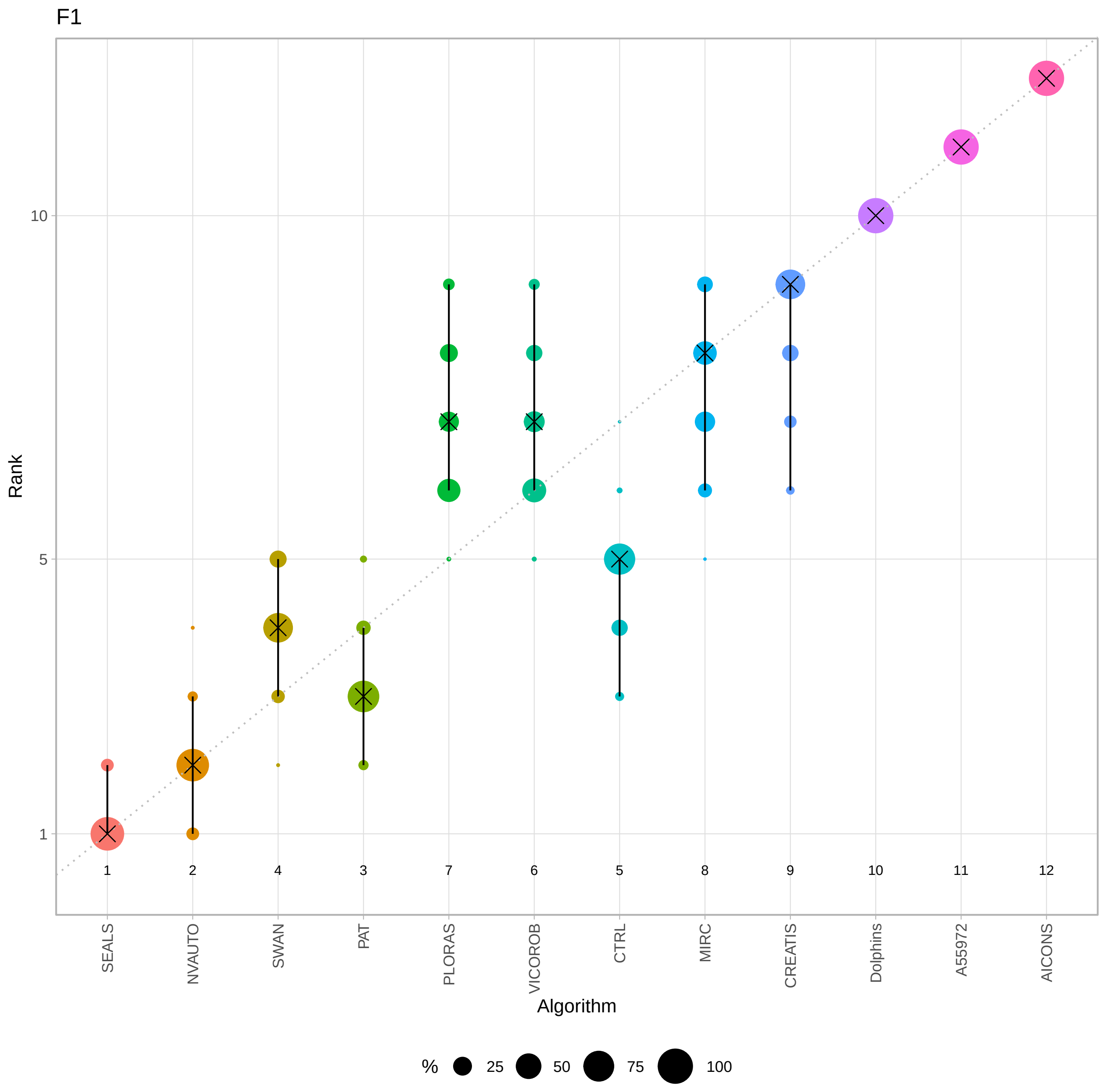

Figure S4.53: F1 Task-Level Ranking Stability – Blob Plot.

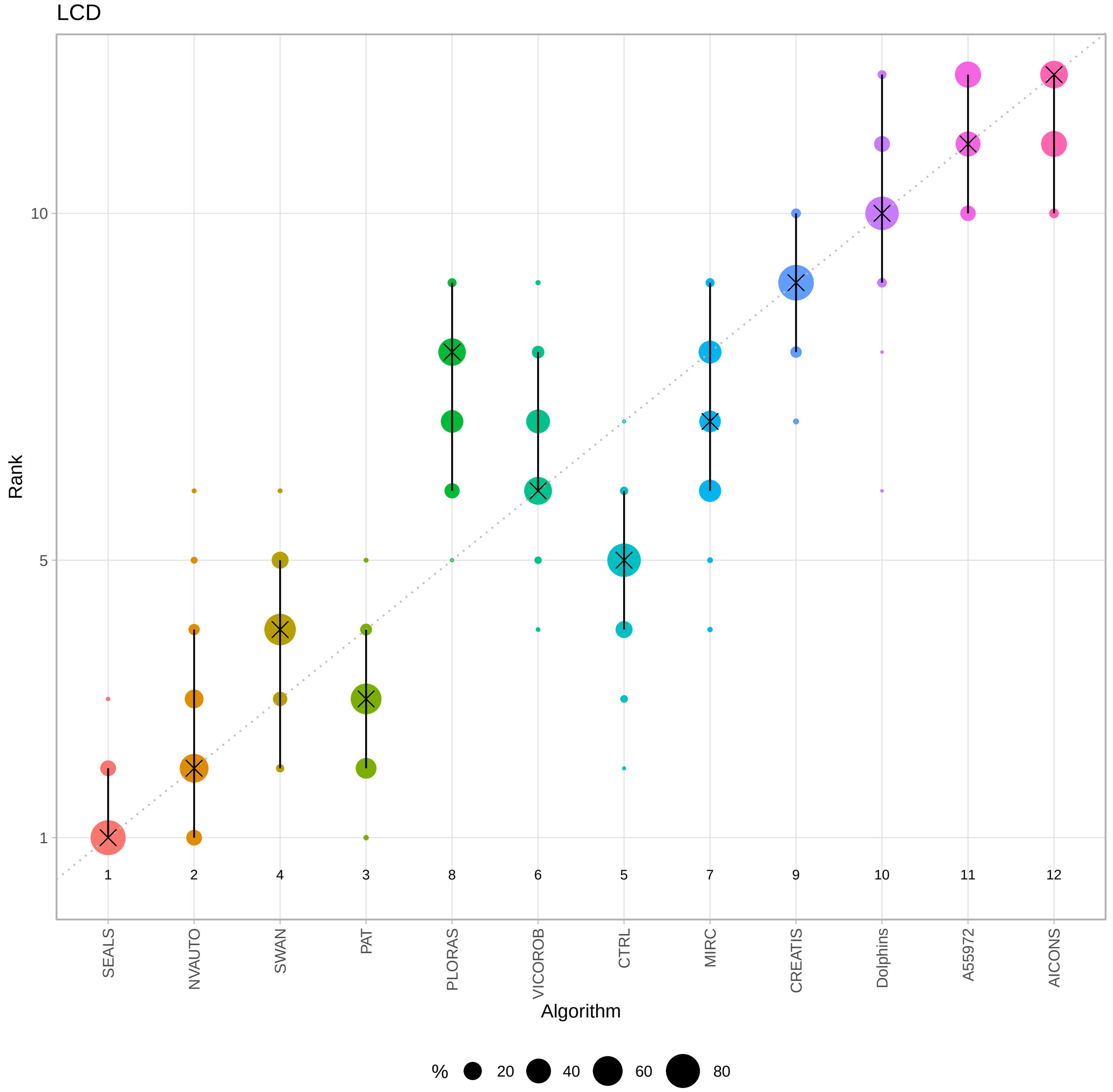

Figure S4.54: LCD Task-Level Ranking Stability – Blob Plot.

4.2.2 Cluster Analysis

Dendrogram from hierarchical cluster analysis and *network-type graphs* for assessing the similarity of tasks based on challenge rankings.

A dendrogram is a visualization approach based on hierarchical clustering. It depicts clusters according to a chosen distance measure (here: Spearman's footrule) as well as a chosen agglomeration method (here: complete and average agglomeration).

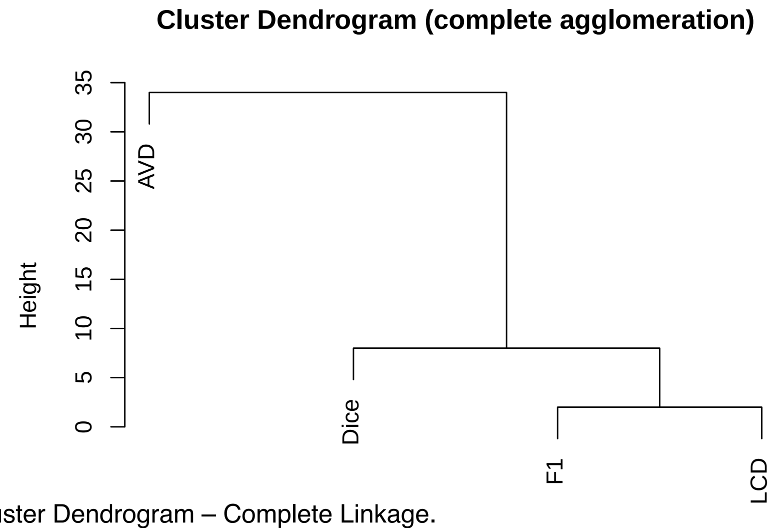

Figure S4.55: Cluster Dendrogram – Complete Linkage.

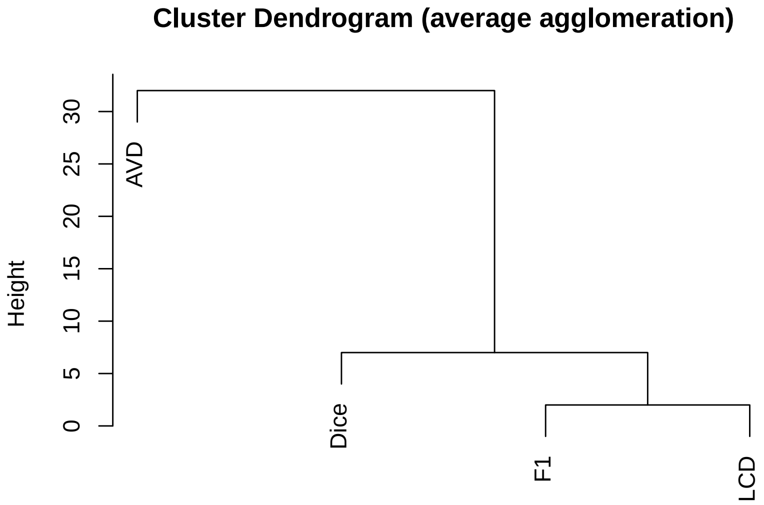

Figure S4.56: Cluster Dendrogram – Average Linkage.

5 References

Wiesenfarth, M., Reinke, A., Landman, B.A., Eisenmann, M., Aguilera Saiz, L., Cardoso, M.J., Maier-Hein, L. and Kopp-Schneider, A. Methods and open-source toolkit for analyzing and visualizing challenge results. *Sci Rep* **11**, 2369 (2021). https://doi.org/10.1038/s41598-021-82017-6

M. J. A. Eugster, T. Hothorn, and F. Leisch, "Exploratory and inferential analysis of benchmark experiments," Institut fuer Statistik, Ludwig-Maximilians-Universitaet Muenchen, Germany, Technical Report 30, 2008. [Online]. Available: http://epub.ub.uni-muenchen.de/4134/.

Supplementary material #5.

Benchmarking report for ISLES'22 - Emulated challenge including DeepISLES

created by challengeR v1.0.5

06 December, 2024

This document presents a systematic report on the benchmark study "ISLES'22 - Emulated challenge including DeepISLES". Input data comprises raw metric values for all algorithms and cases. Generated plots are:

- Visualization of assessment data: Dot- and boxplots, podium plots and ranking heatmaps
- Visualization of ranking stability: Blob plots, violin plots and significance maps, line plots
- Visualization of cross-task insights: Blob plots, stacked frequency plots, dendrograms

Details can be found in Wiesenfarth et al. (2021).

1 Rankings

Algorithms within a task are ranked according to the following ranking scheme:

rank then aggregate using function ("mean")

Ranking for each task:

AVD : The analysis is based on 13 algorithms and 150 cases. 0 missing cases have been found in the data set.

	rank_mean	rank
NVAUTO	5.226667	1
DeepISLES	5.253333	2
SEALS	5.413333	3
SWAN	5.540000	4
PLORAS	5.880000	5
PAT	6.060000	6
VICOROB	6.106667	7
CTRL	6.213333	8
Dolphins	6.620000	9
CREATIS	7.060000	10
MIRC	7.966667	11
AICONS	10.820000	12
A55972	11.993333	13

Table S5.1: AVD Ranking.

Dice : The analysis is based on 13 algorithms and 150 cases. 0 missing cases have been found in the data set.

	rank_mean	rank
DeepISLES	3.046667	1
NVAUTO	3.480000	2
SEALS	3.900000	3
SWAN	4.500000	4
PAT	4.886667	5
CTRL	5.253333	6
VICOROB	6.373333	7
PLORAS	7.920000	8
MIRC	8.306667	9
Dolphins	8.466667	10
CREATIS	8.760000	11
AICONS	11.666667	12
A55972	12.346667	13

Table S5.2: Dice Ranking.

F1 : The analysis is based on 13 algorithms and 150 cases. 0 missing cases have been found in the data set.

	rank_mean	rank
SEALS	2.553333	1
DeepISLES	3.233333	2
NVAUTO	3.580000	3
PAT	3.720000	4
SWAN	4.186667	5
CTRL	4.273333	6
VICOROB	5.693333	7
PLORAS	5.973333	8
MIRC	6.160000	9
CREATIS	7.220000	10
Dolphins	8.233333	11
A55972	9.720000	12
AICONS	11.813333	13

Table S5.3: F1 Score Ranking.

LCD : The analysis is based on 13 algorithms and 150 cases. 0 missing cases have been found in the data set.

	rank_mean	rank
SEALS	3.566667	1
DeepISLES	3.766667	2
PAT	4.233333	3
NVAUTO	4.253333	4
SWAN	4.420000	5
CTRL	4.913333	6
VICOROB	5.693333	7
MIRC	5.900000	8
PLORAS	5.900000	8
CREATIS	6.760000	10
Dolphins	6.846667	11
AICONS	8.720000	12
A55972	8.946667	13

Table S5.4: LCD Ranking.

Consensus ranking across tasks according to chosen method “euclidean”:

	value	rank
DeepISLES	1.750	1
SEALS	2.000	2
NVAUTO	2.500	3
PAT	4.500	4
SWAN	4.500	4
CTRL	6.500	6
VICOROB	7.000	7
PLORAS	7.375	8
MIRC	9.375	9
CREATIS	10.250	10
Dolphins	10.250	10
AICONS	12.250	12
A55972	12.750	13

Table S5.5: Consensus Ranking.

2 Visualization of raw assessment data

The algorithms are ordered according to the computed ranks for each task.

2.1 Dot- and boxplot

Dot- and boxplots for visualizing raw assessment data separately for each algorithm. Boxplots representing descriptive statistics over all cases (median, quartiles and outliers) are combined with horizontally jittered dots representing individual cases.

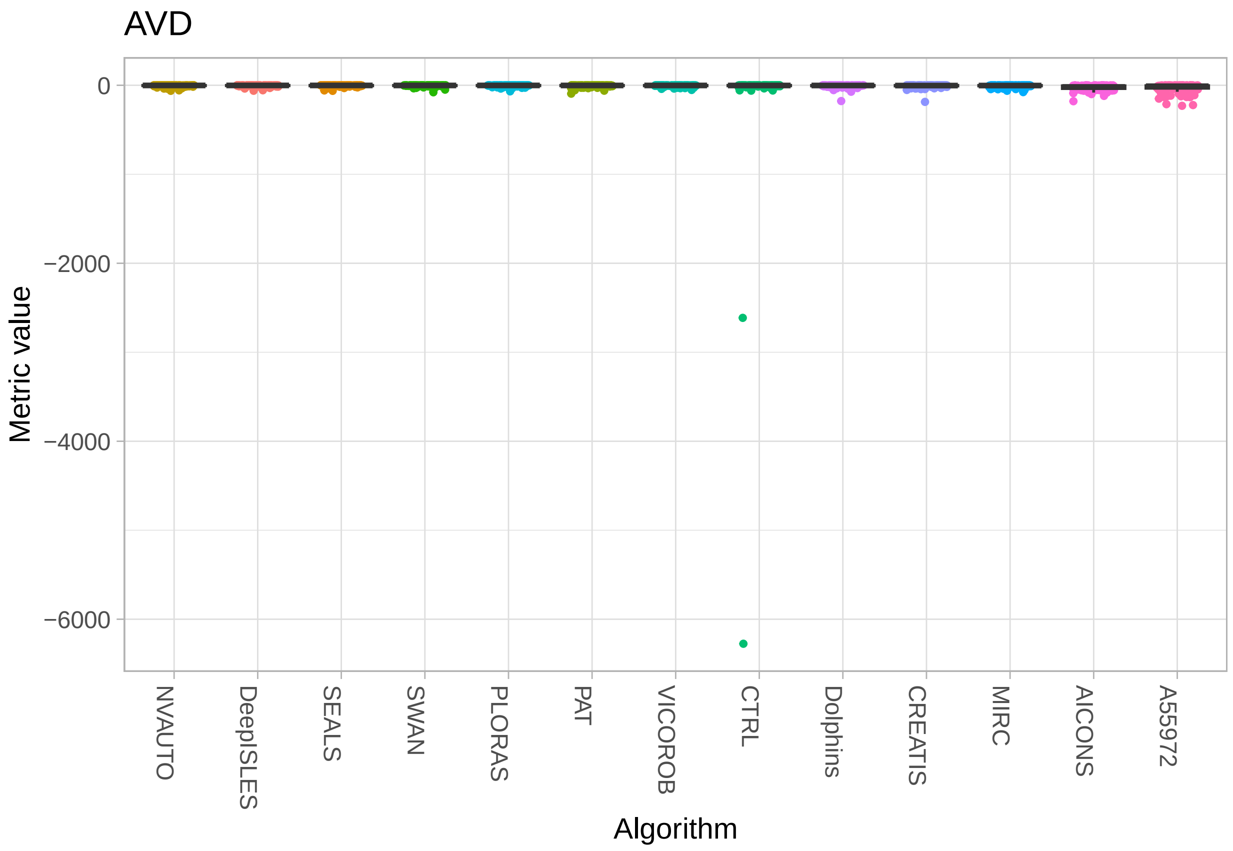

Figure S5.1: AVD Boxplots.

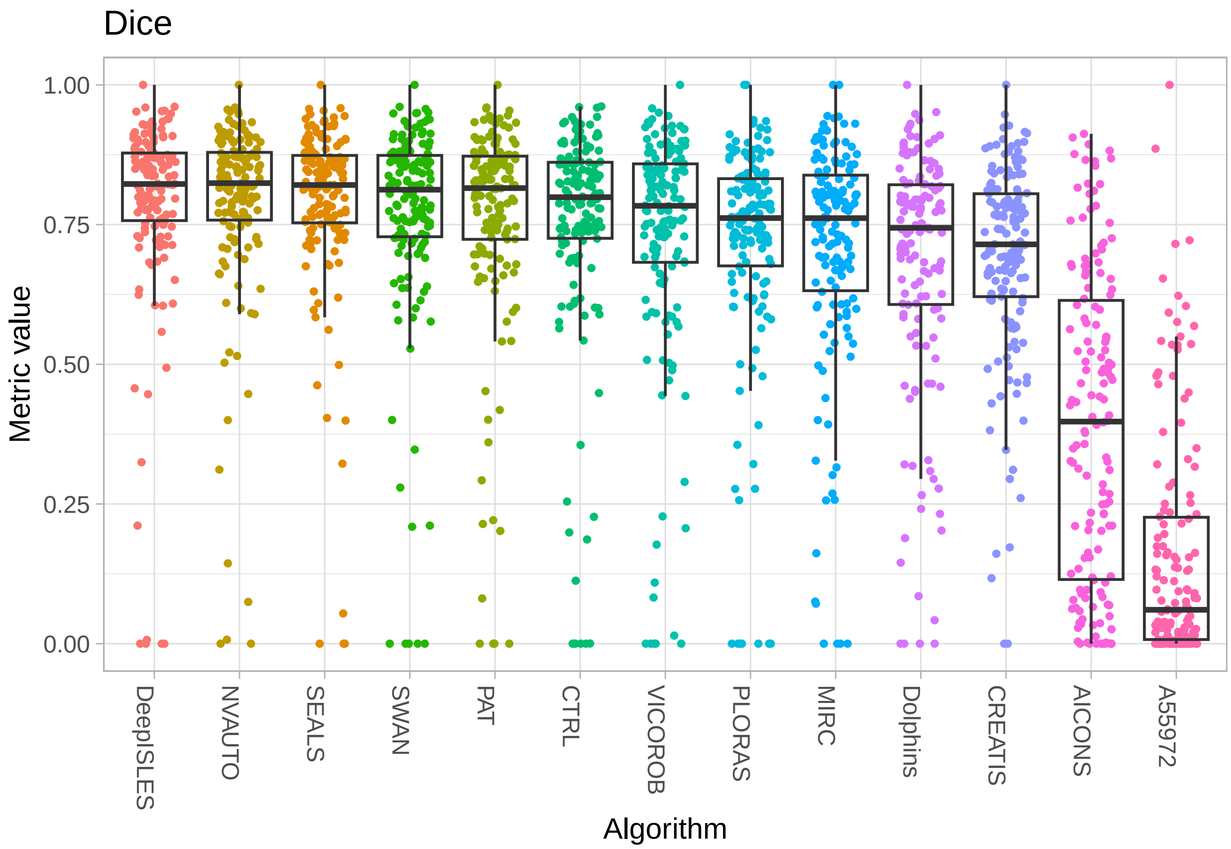

Figure S5.2: Dice Boxplots.

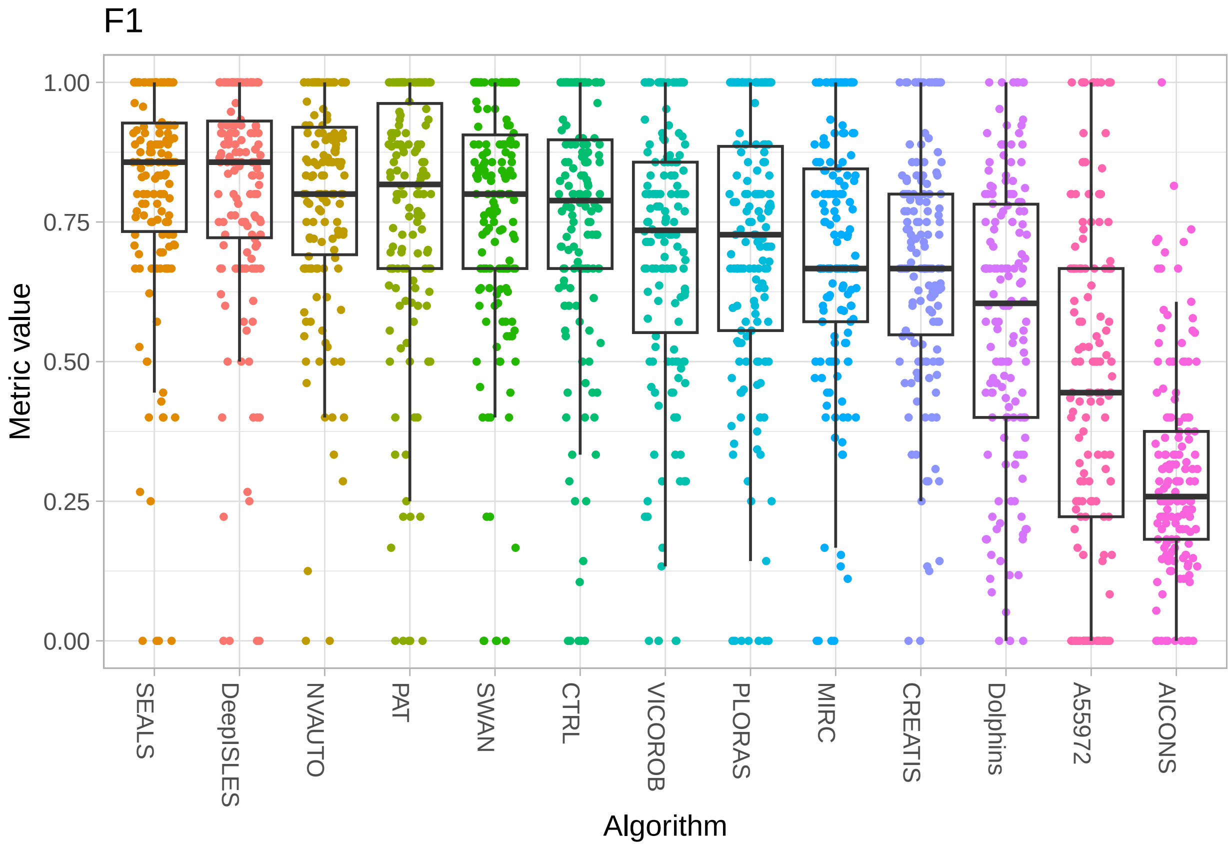

Figure S5.3: F1 Boxplots.

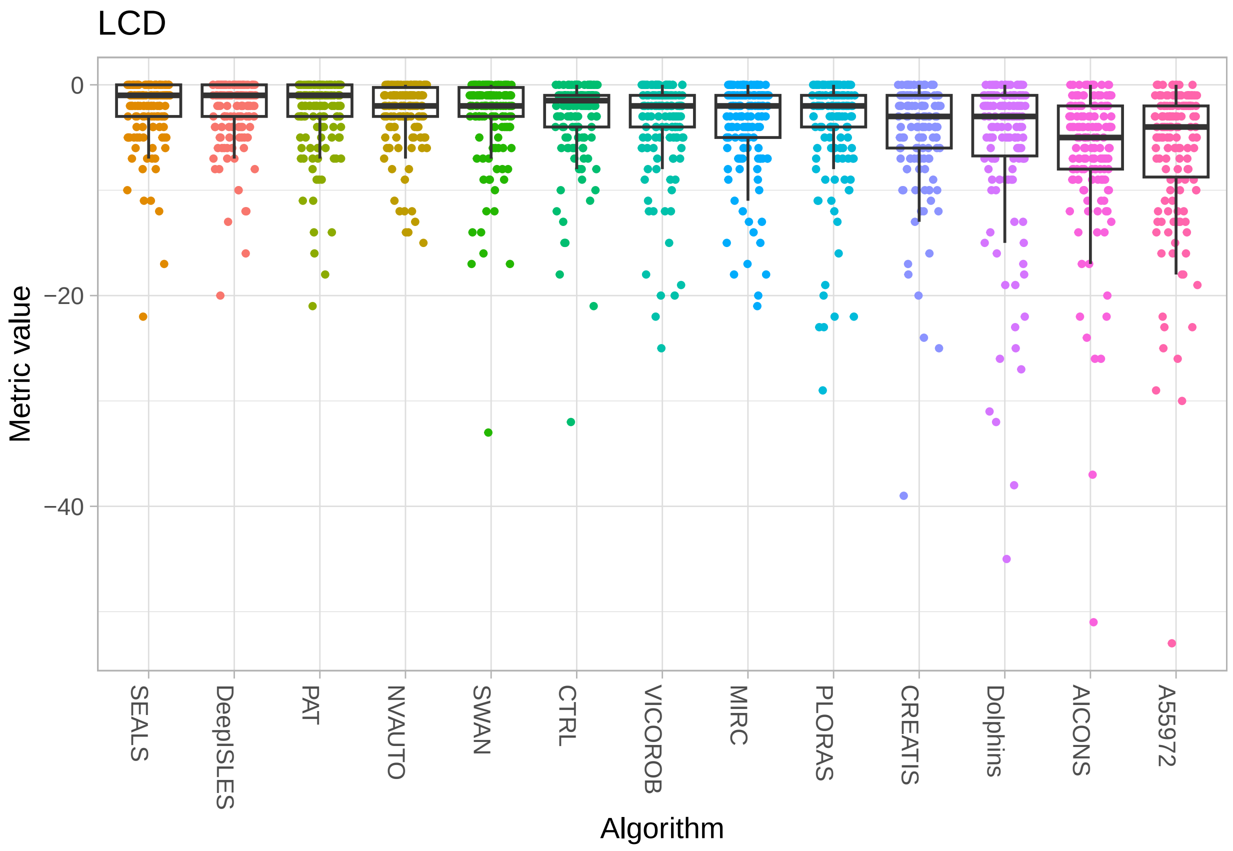

Figure S5.4: LCD Boxplots.

2.2 Podium plot

Podium plots (see also Eugster et al., 2008) for visualizing raw assessment data. Upper part (spaghetti plot): Participating algorithms are color-coded, and each colored dot in the plot represents a metric value achieved with the respective algorithm. The actual metric value is encoded by the y-axis. Each podium (here: p=13) represents one possible rank, ordered from best (1) to last (here: 13). The assignment of metric values (i.e. colored dots) to one of the podiums is based on the rank that the respective algorithm achieved on the corresponding case. Note that the plot part above each podium place is further subdivided into p "columns", where each column represents one participating algorithm (here: p = 13). Dots corresponding to identical cases are connected by a line, leading to the shown spaghetti structure. Lower part: Bar charts represent the relative frequency for each algorithm to achieve the rank encoded by the podium place.

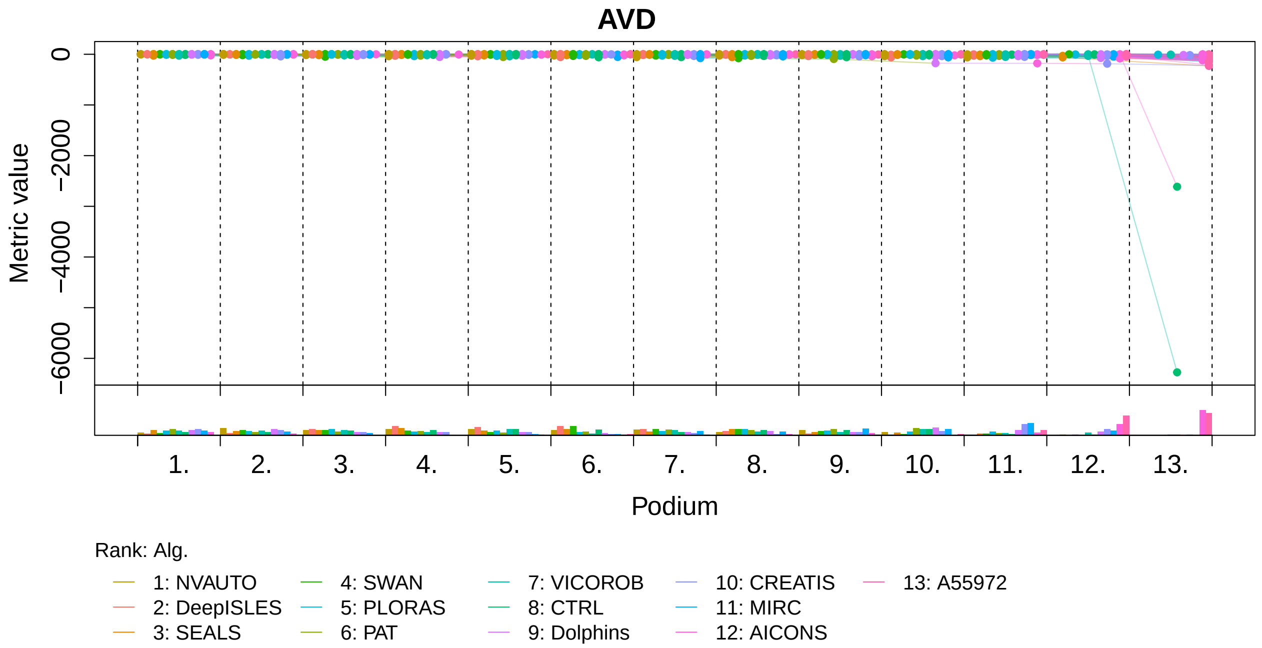

Figure S5.5: AVD Podium Plot.

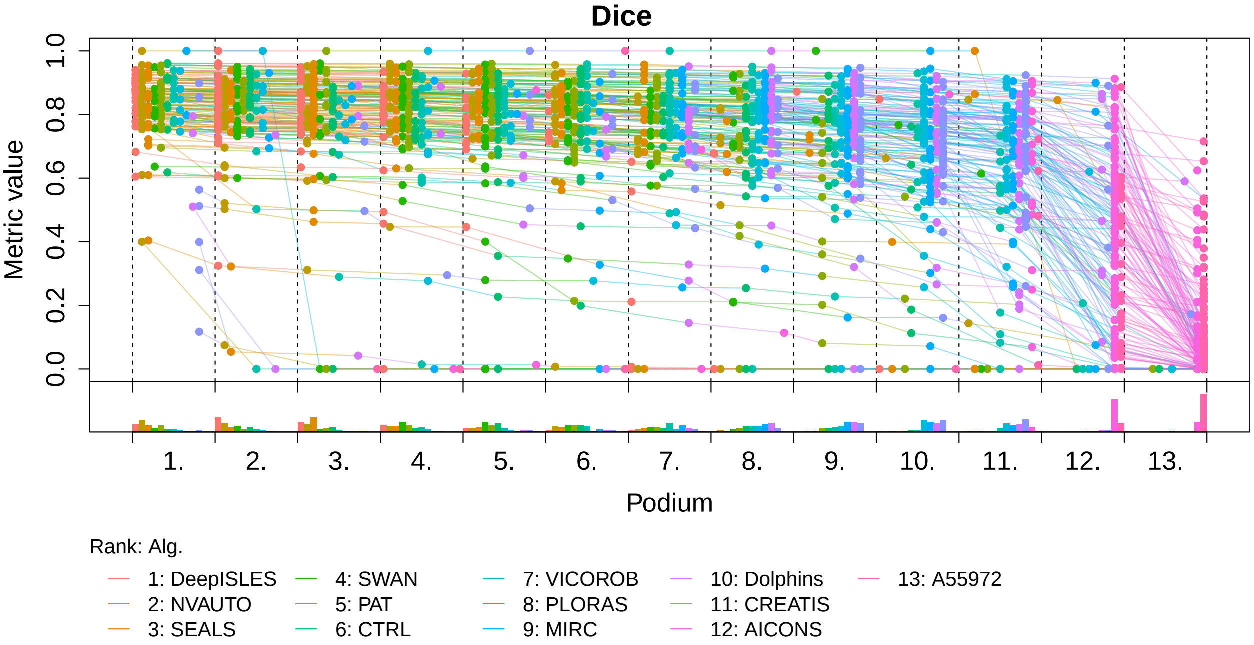

Figure S5.6: Dice Podium Plot.

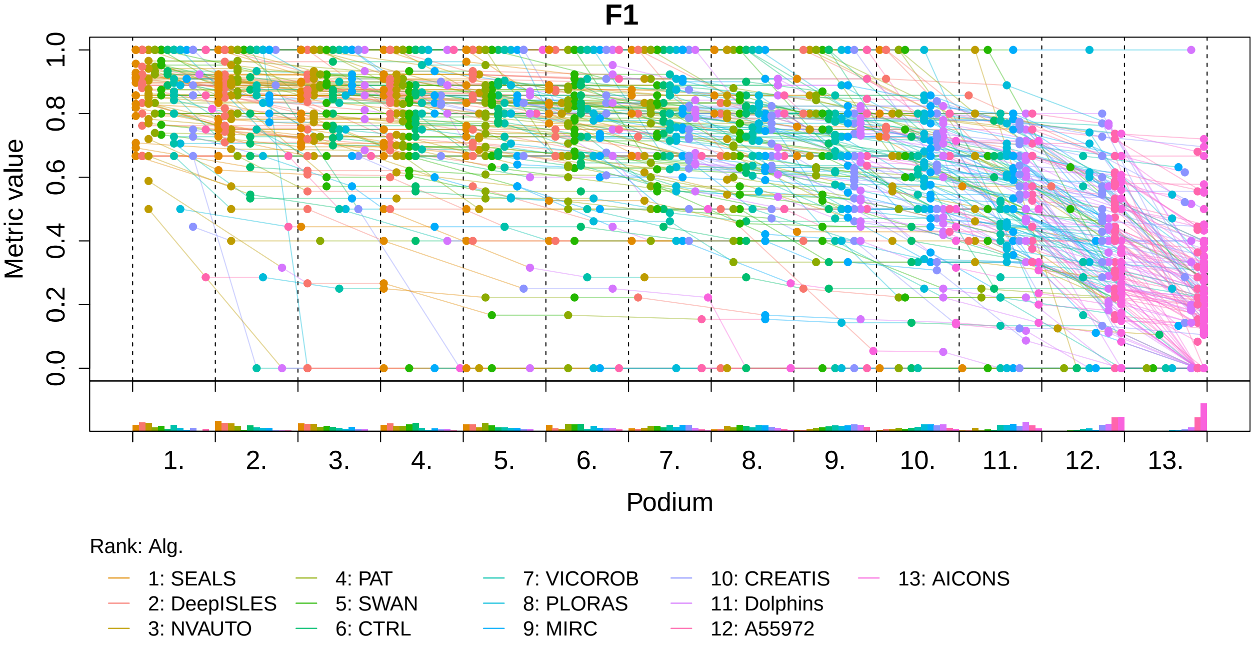

Figure S5.7: F1 Podium Plot.

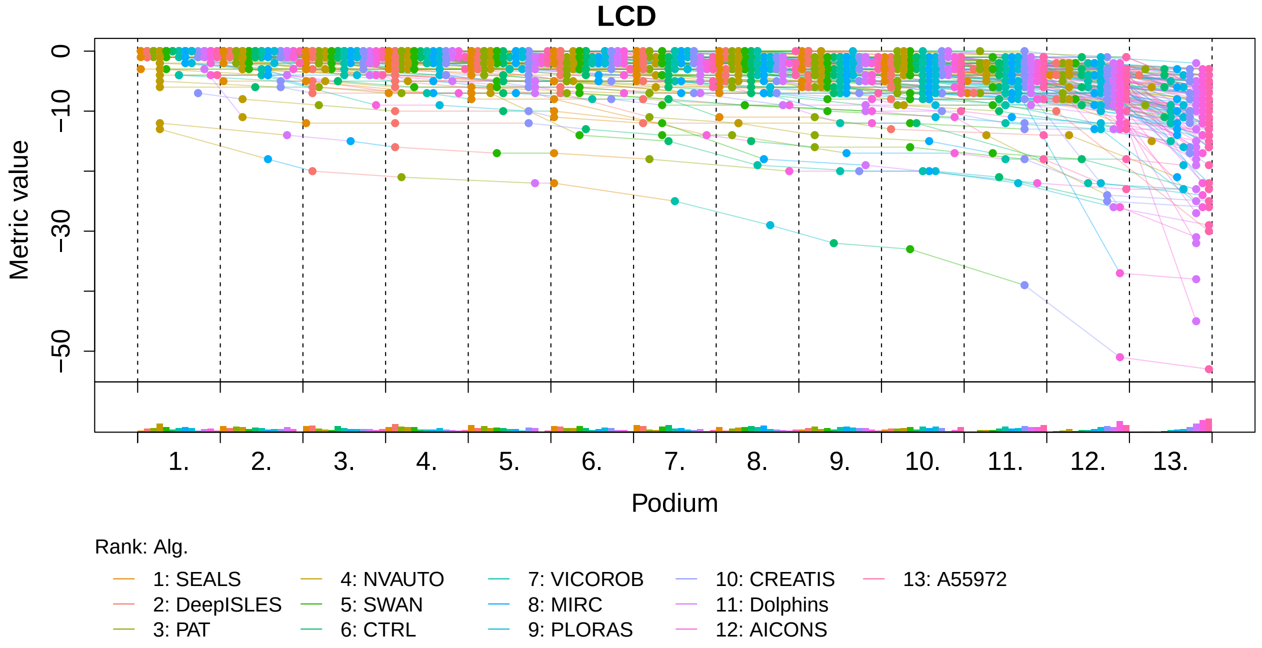

Figure S5.8: LCD Podium Plot.

2.3 Ranking heatmap

Ranking heatmaps for visualizing raw assessment data. Each cell (i, A_j) shows the absolute frequency of cases in which algorithm A_j achieved rank i.

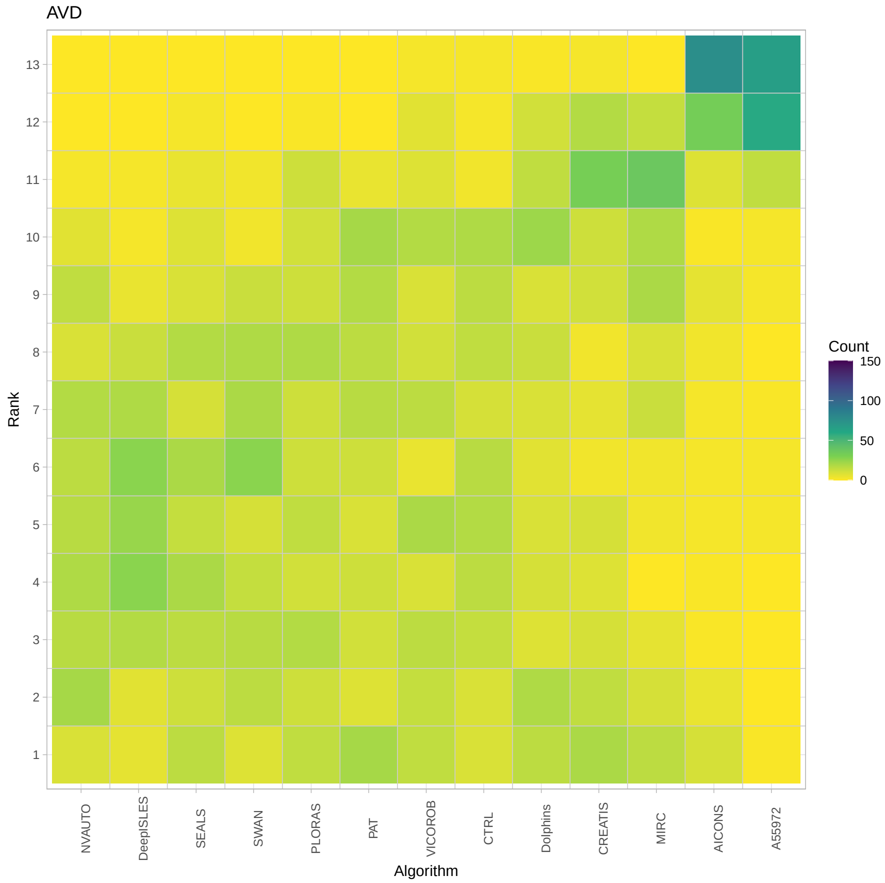

Figure S5.9: AVD Ranking Heatmap.

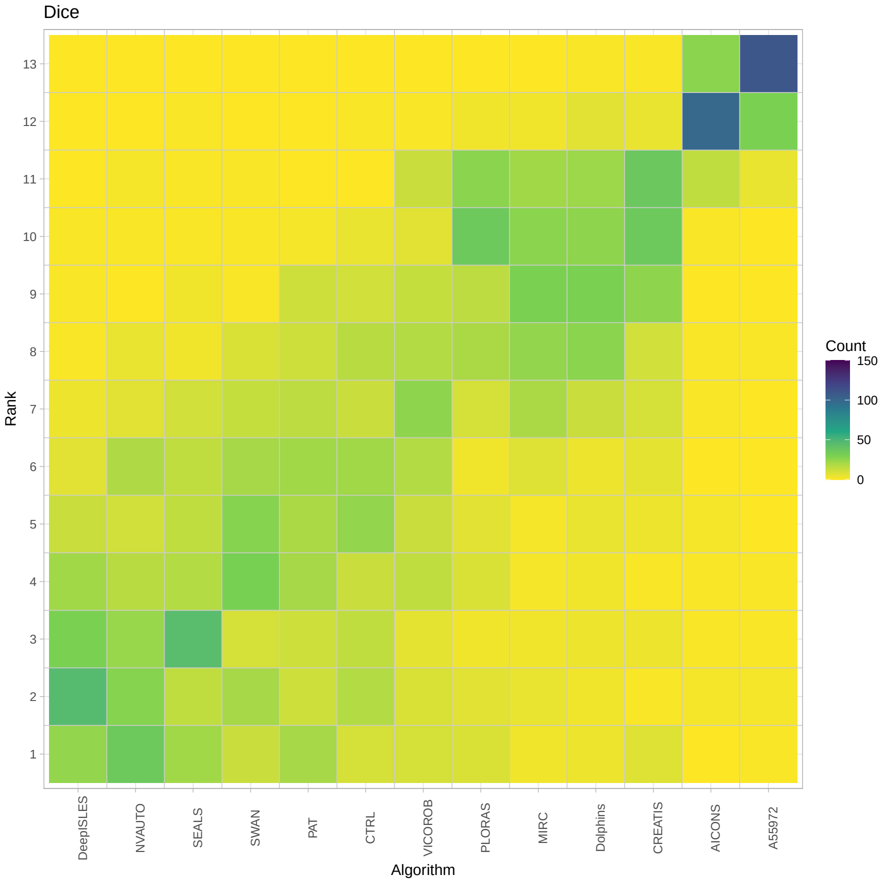

Figure S5.10: Dice Ranking Heatmap.

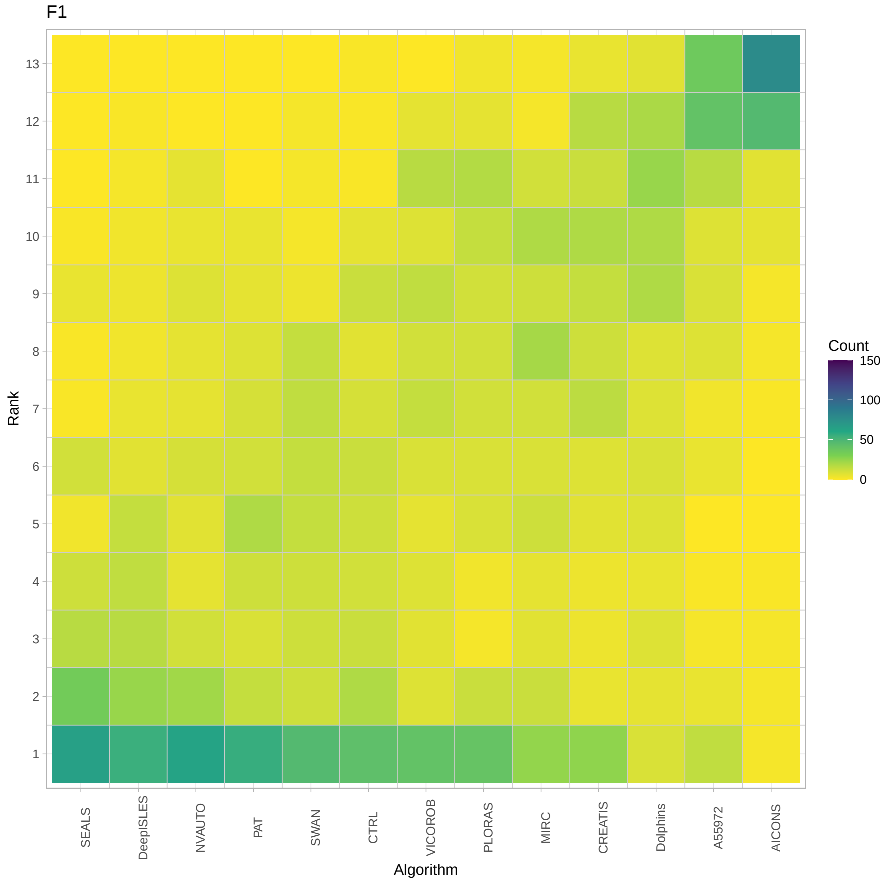

Figure S5.11: F1 Ranking Heatmap.

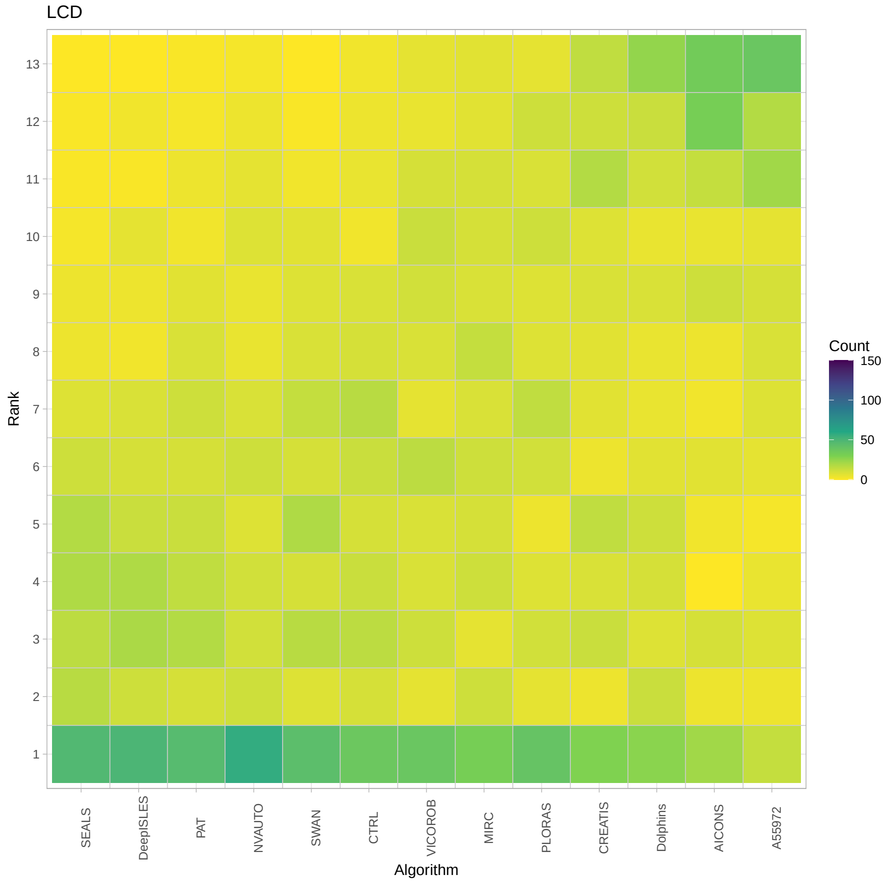

Figure S5.12: LCD Ranking Heatmap.

3 Visualization of ranking stability

3.1 *Blob plot* for visualizing ranking stability based on bootstrap sampling

Algorithms are color-coded, and the area of each blob at position $(A_i, \text{rank } j)$ is proportional to the relative frequency A_i achieved rank j across $b = 1000$ bootstrap samples. The median rank for each algorithm is indicated by a black cross. 95% bootstrap intervals across bootstrap samples are indicated by black lines.

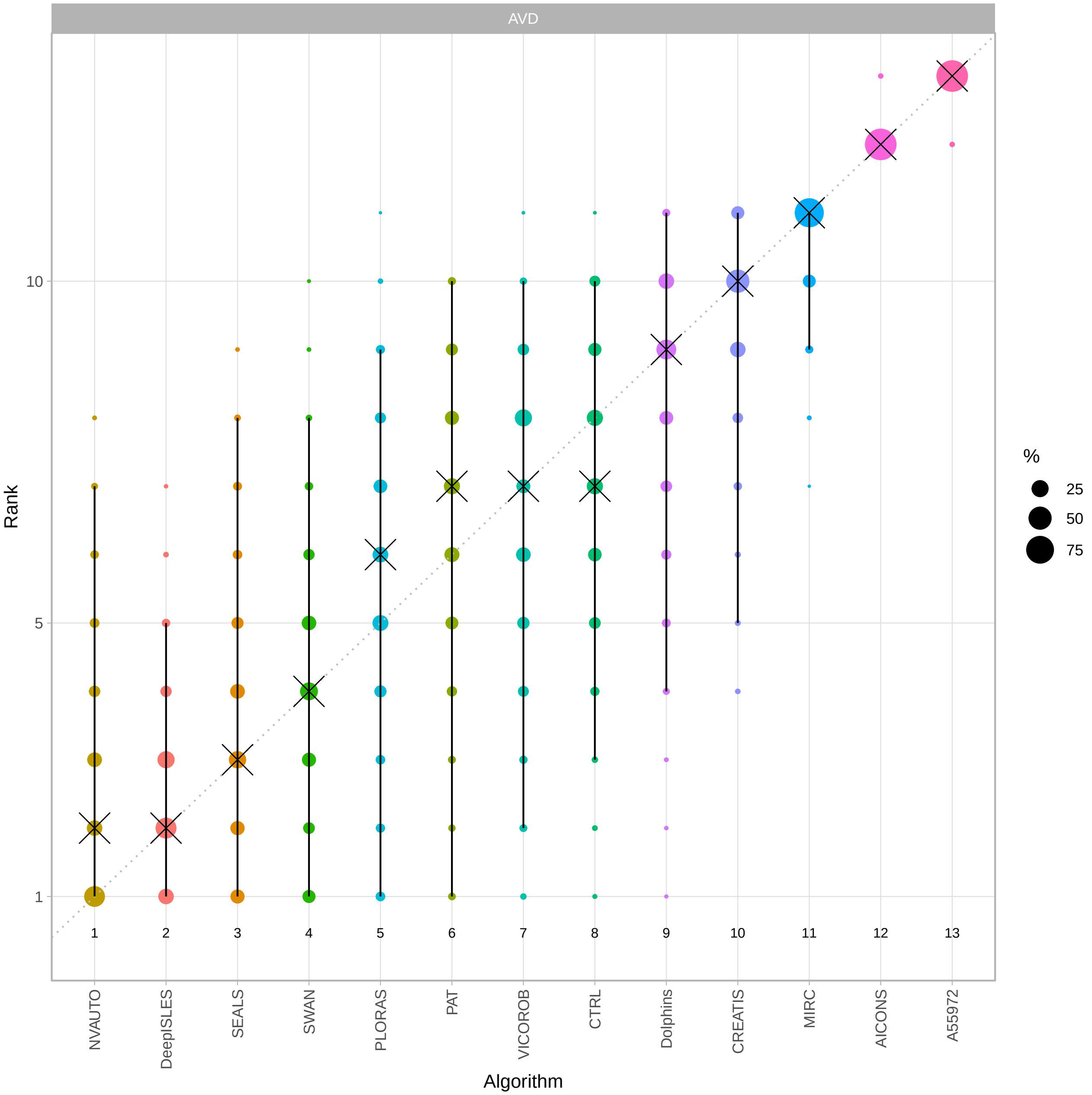

Figure S5.13: AVD Ranking Stability – Blob Plot.

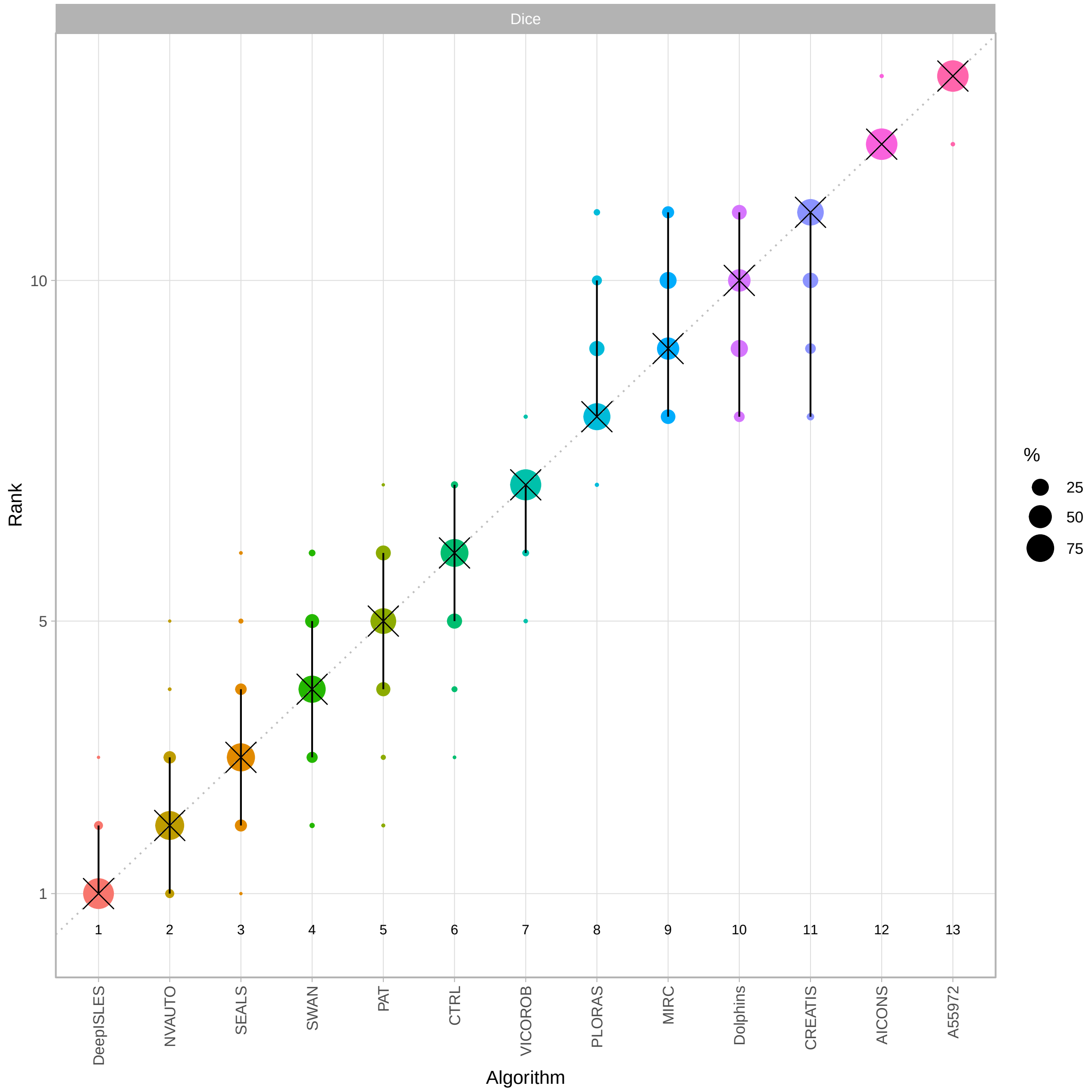

Figure S5.14: Dice Ranking Stability – Blob Plot.

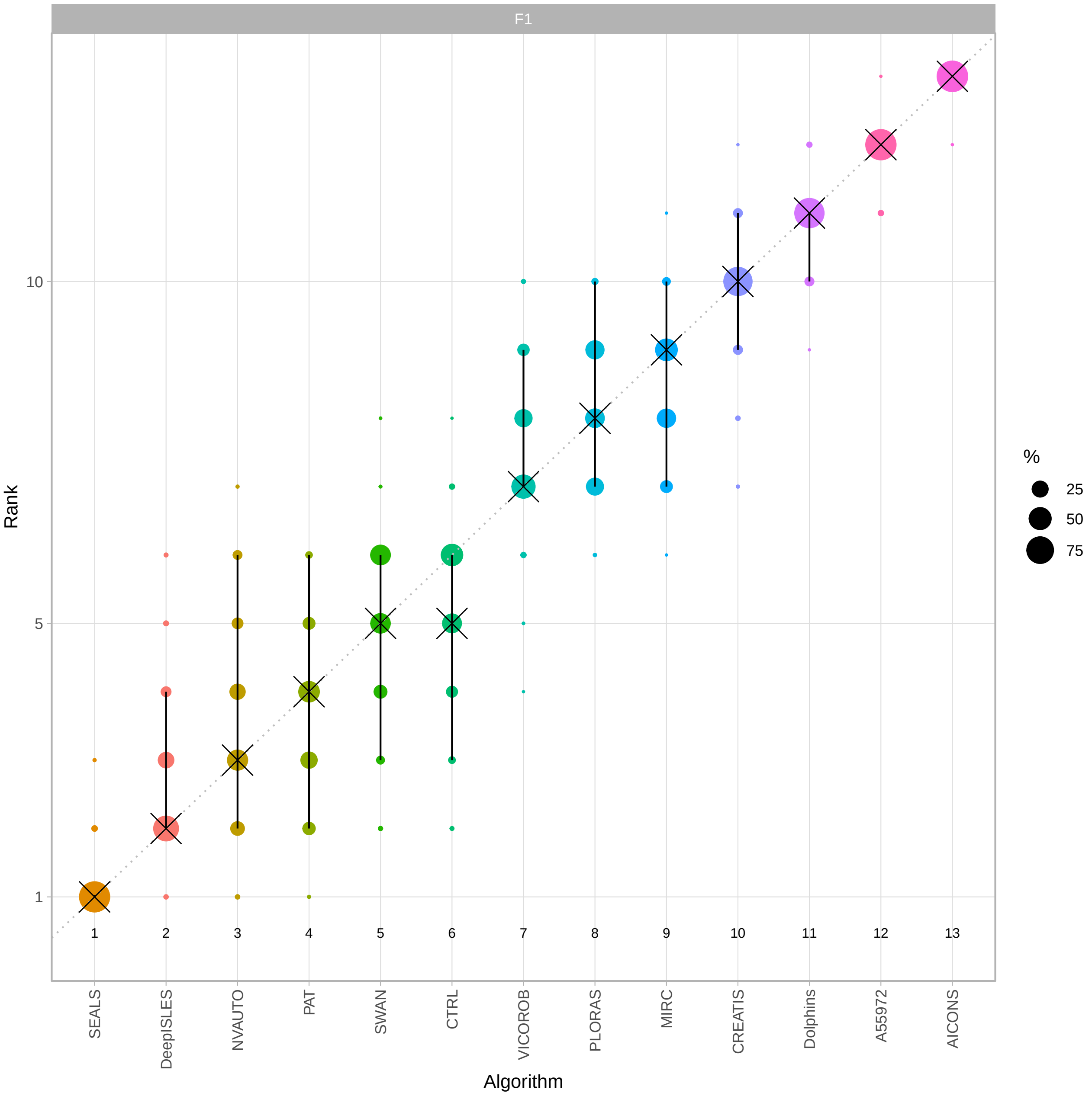

Figure S5.15: F1 Ranking Stability – Blob Plot.

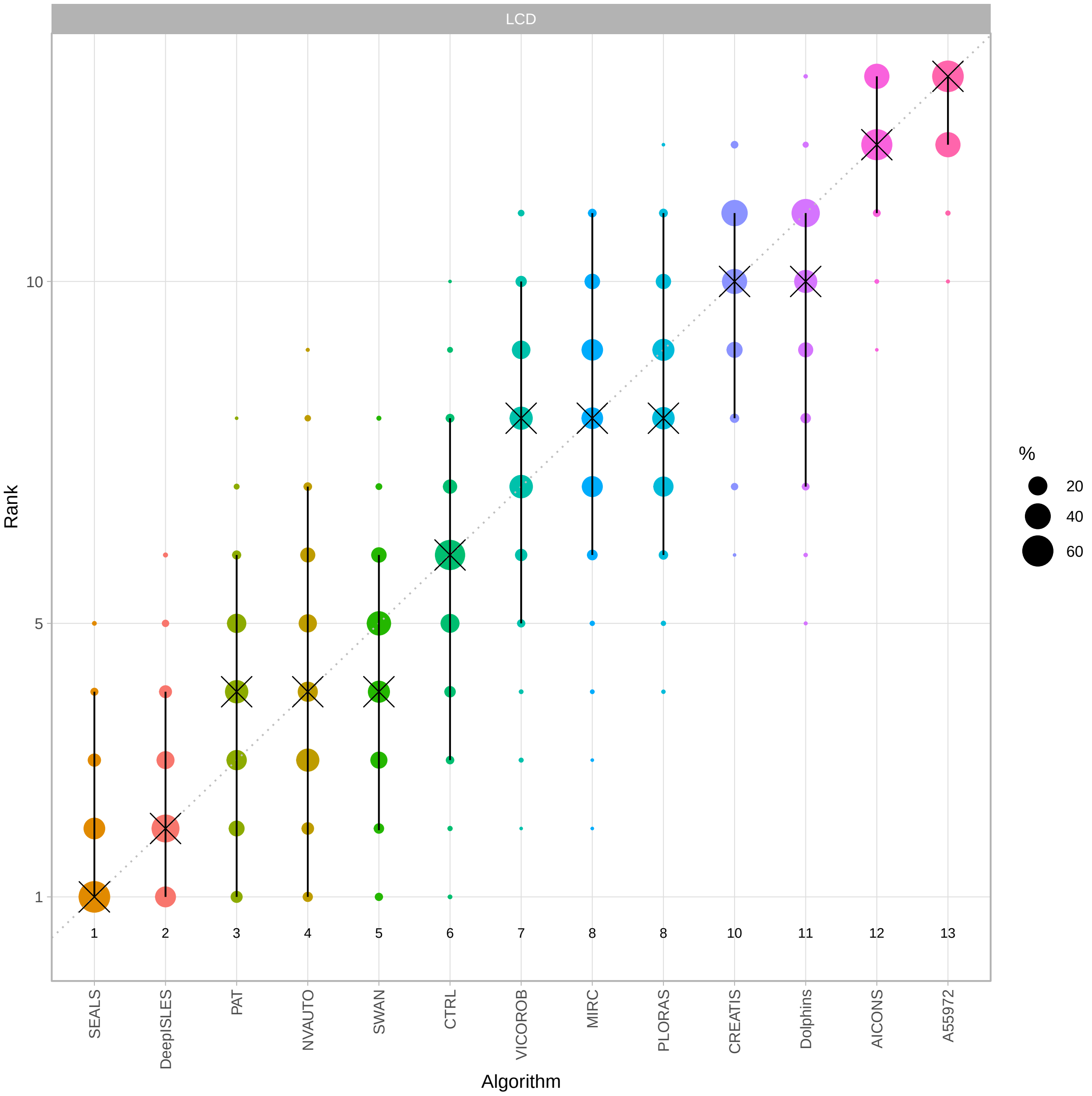

Figure S5.16: LCD Ranking Stability – Blob Plot.

3.2 *Violin plot* for visualizing ranking stability based on bootstrapping

The ranking list based on the full assessment data is pairwise compared with the ranking lists based on the individual bootstrap samples (here $b = 1000$ samples). For each pair of rankings, Kendall's τ correlation is computed. Kendall's τ is a scaled index determining the correlation between the lists. It is computed by evaluating the number of pairwise concordances and discordances between ranking lists and produces values between -1 (for inverted order) and 1 (for identical order). A violin plot, which simultaneously depicts a boxplot and a density plot, is generated from the results.

Summary Kendall's tau:

Task	mean	median	q25	q75
Dice	0.9521270	0.9487179	0.9230769	0.9743590
F1	0.9216419	0.9230769	0.8974359	0.9487179
LCD	0.8571128	0.8645341	0.8129201	0.8903411
AVD	0.7846052	0.7948718	0.7435897	0.8461538

Table S5.6: Kendall's Tau Summary for Ranking Stability.

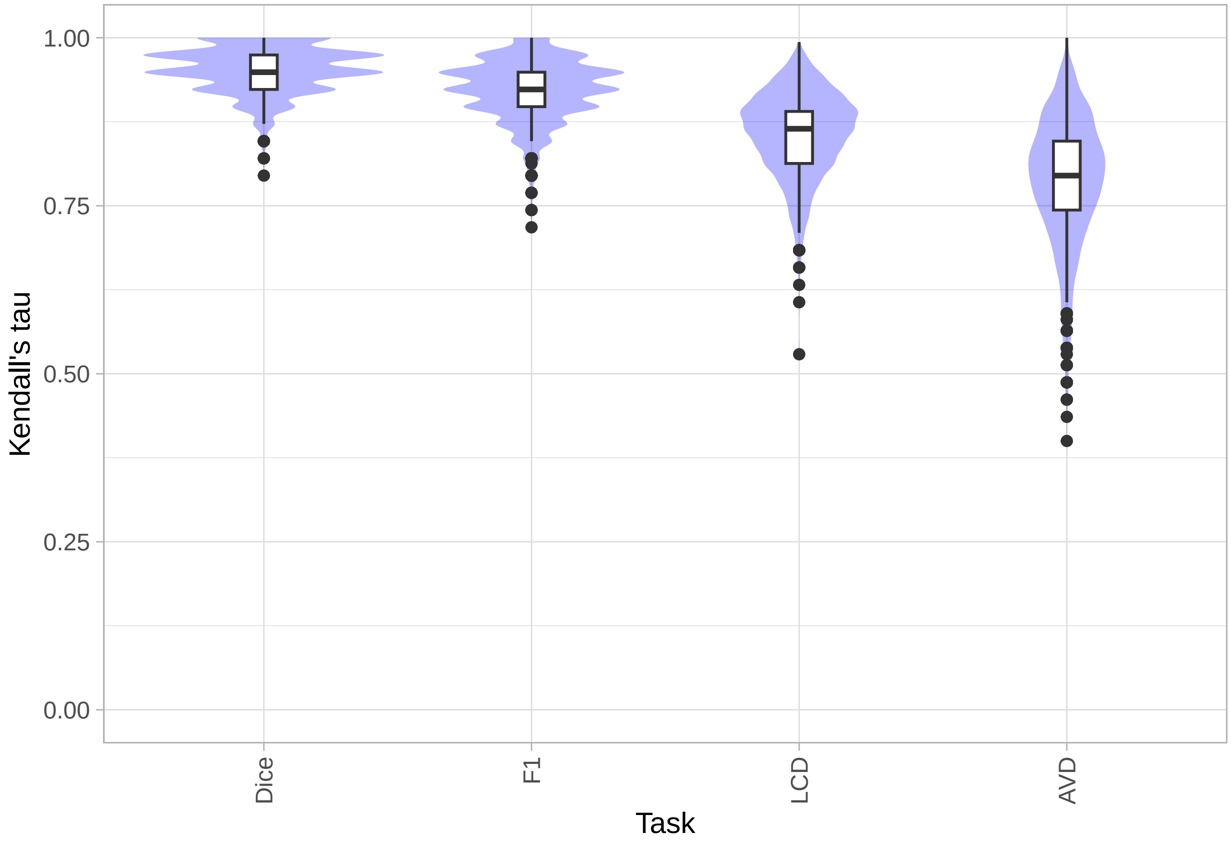

Figure S5.17: Kendall's Tau Violin Plot for All Metrics.

3.3 *Significance maps* for visualizing ranking stability based on statistical significance

Significance maps depict incidence matrices of pairwise significant test results for the one-sided Wilcoxon signed rank test at a 5% significance level with adjustment for multiple testing according to Holm. Yellow shading indicates that metric values from the algorithm on the x-axis were significantly superior to those from the algorithm on the y-axis, blue color indicates no significant difference.

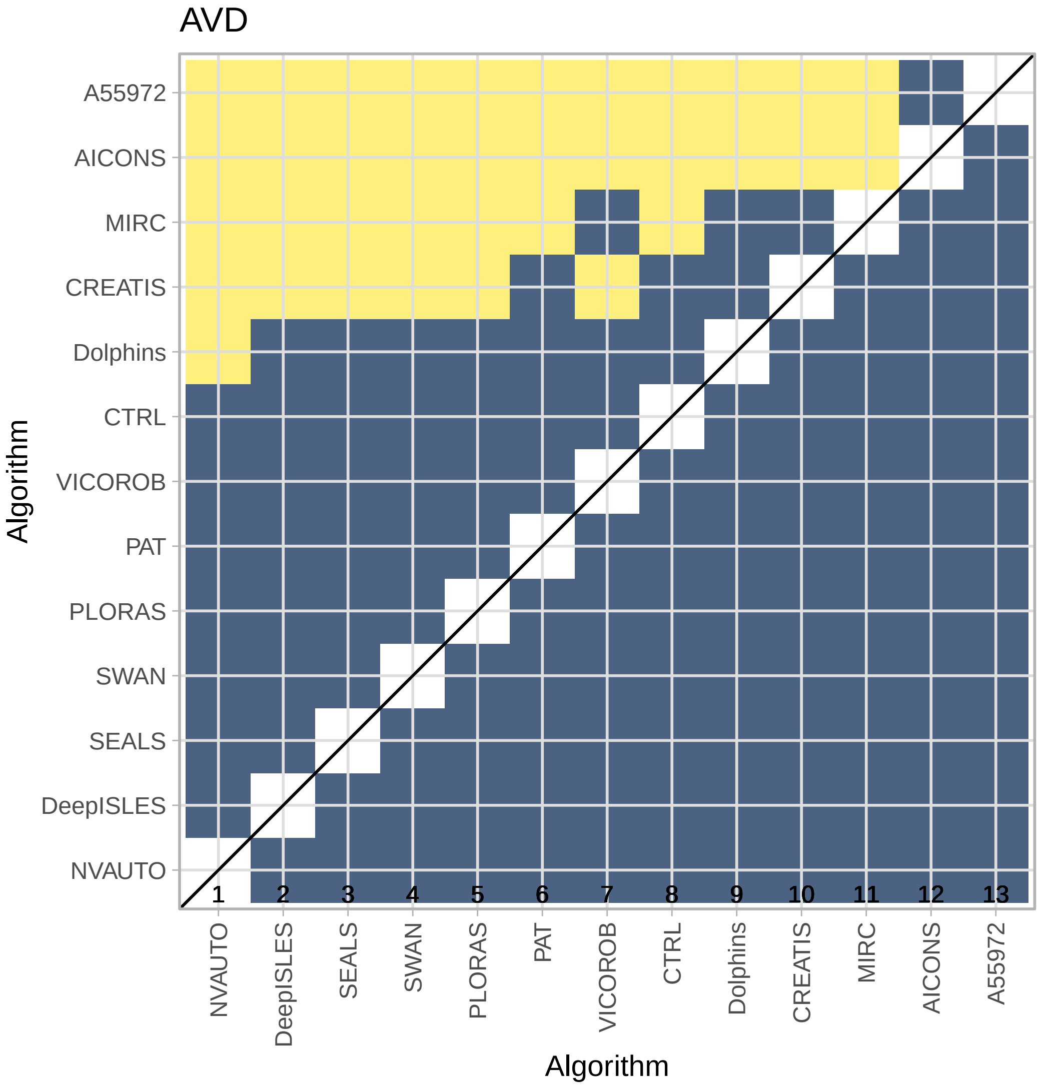

Figure S5.18: AVD Significance Map.

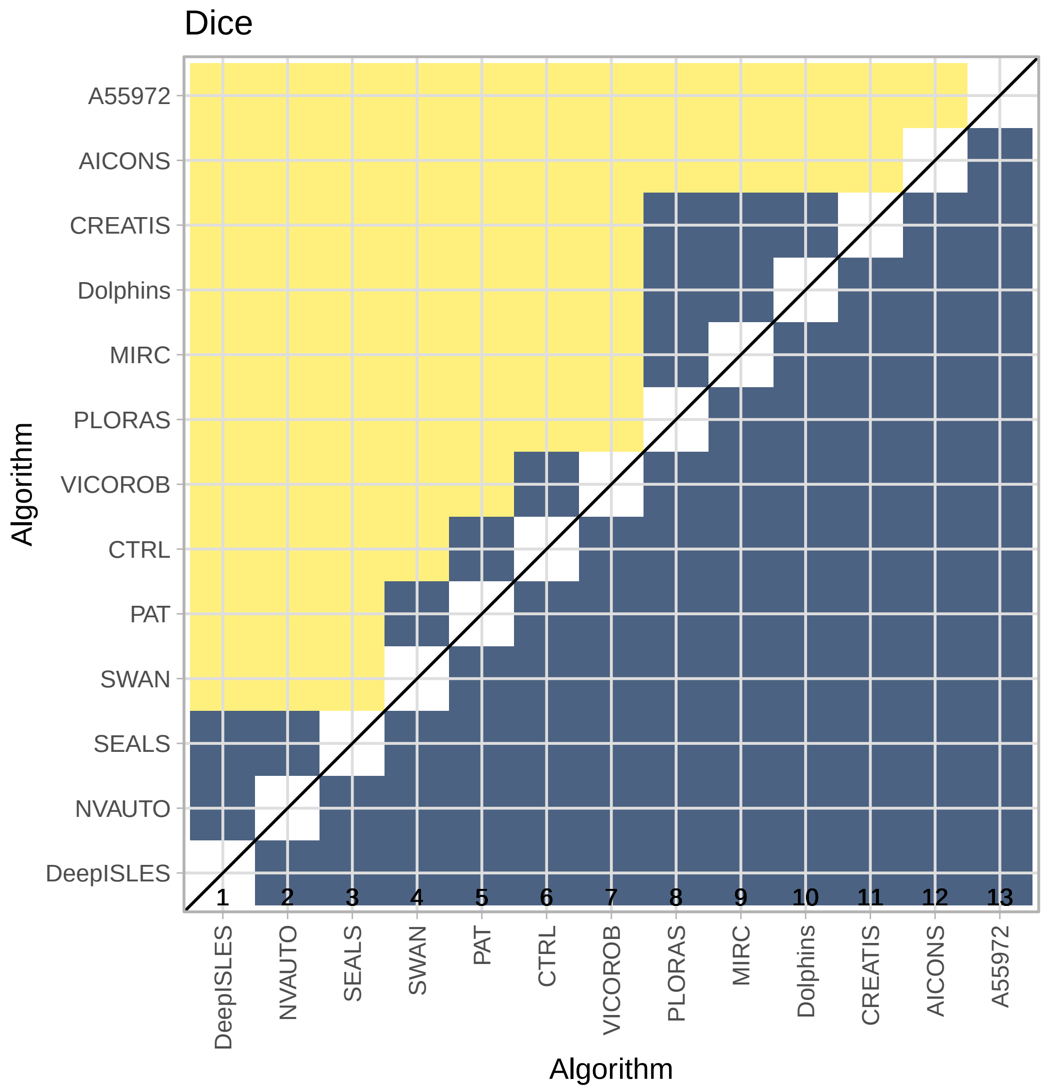

Figure S5.19: Dice Significance Map.

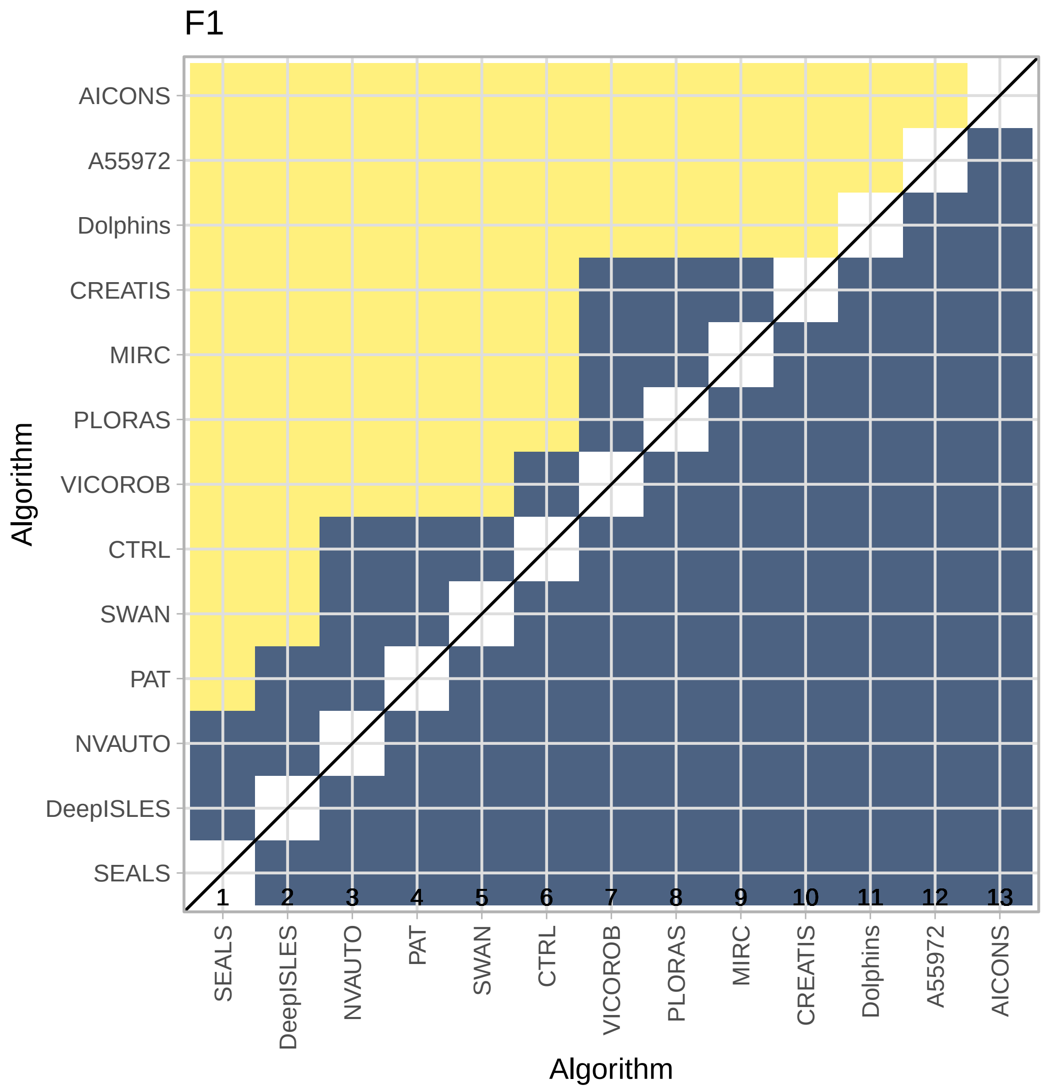

Figure S5.20: F1 Significance Map.

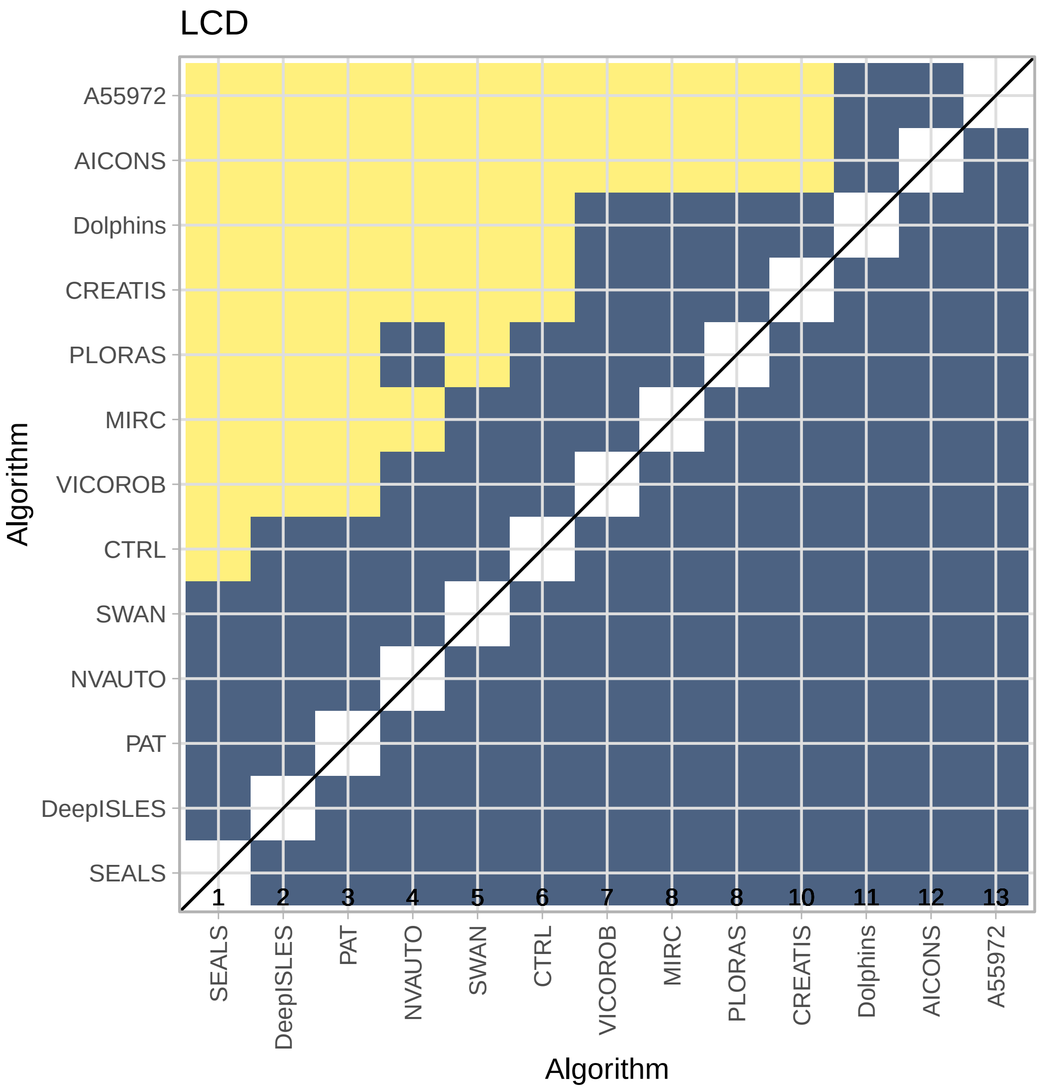

Figure S5.21: LCD Significance Map.

3.4 Ranking robustness to ranking methods

Line plots for visualizing ranking robustness across different ranking methods. Each algorithm is represented by one colored line. For each ranking method encoded on the x-axis, the height of the line represents the corresponding rank. Horizontal lines indicate identical ranks for all methods.

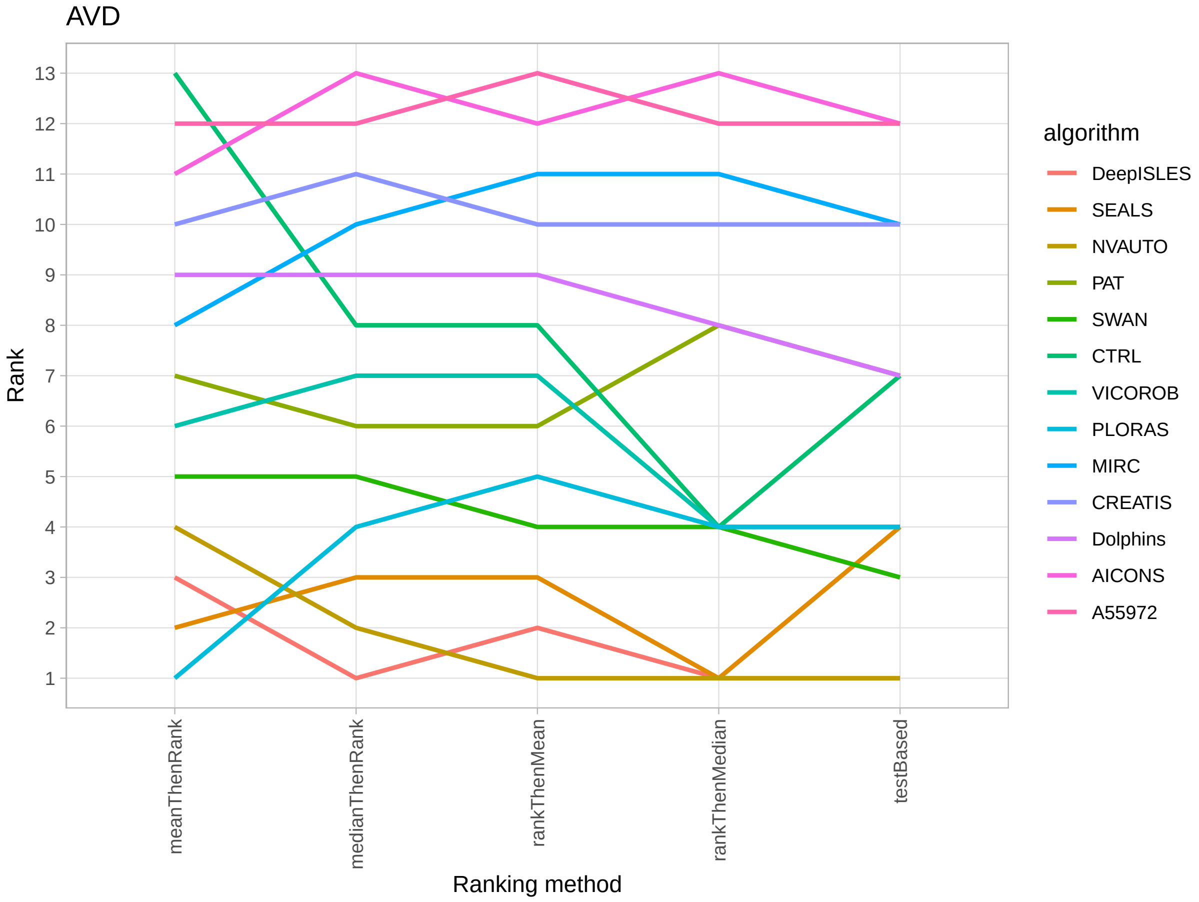

Figure S5.22: AVD Ranking Robustness Across Methods.

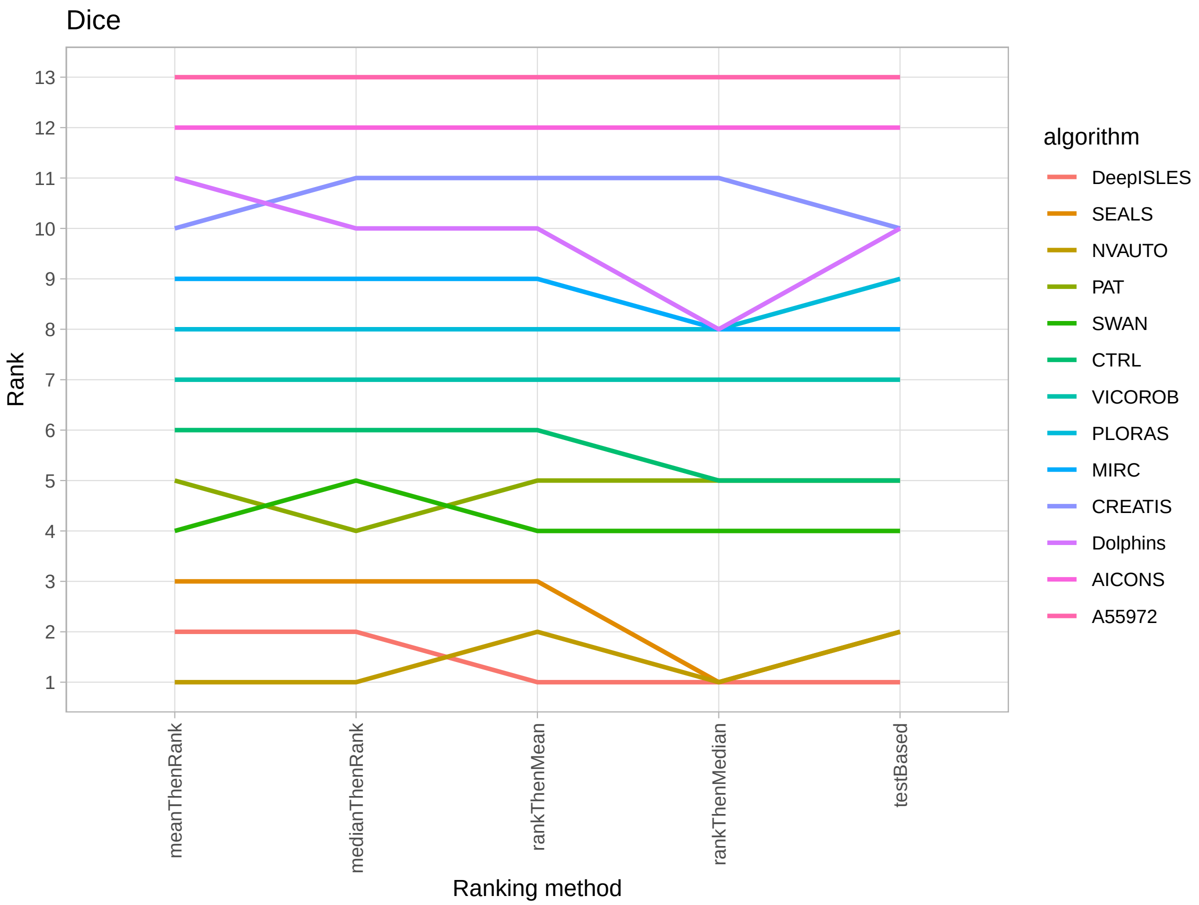

Figure S5.23: Dice Ranking Robustness Across Methods.

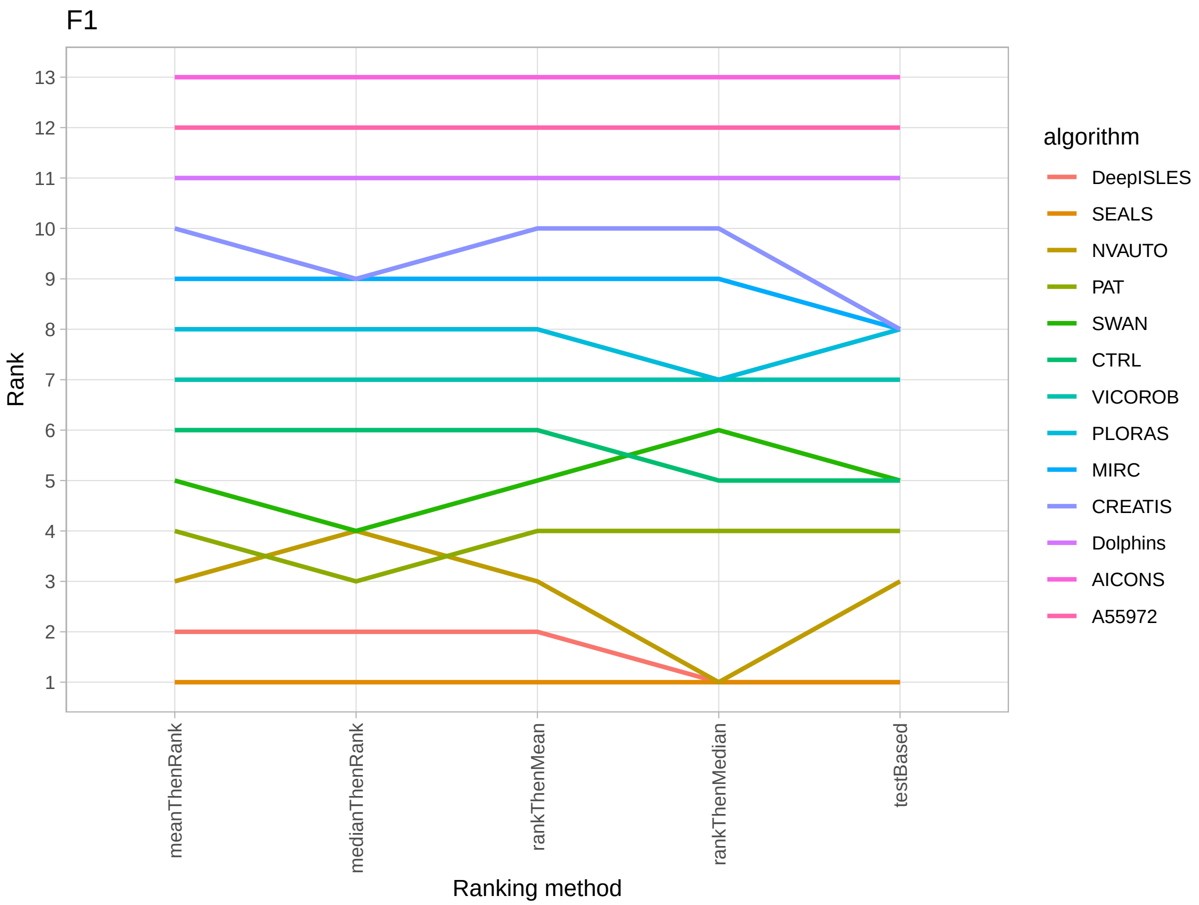

Figure S5.24: F1 Ranking Robustness Across Methods.

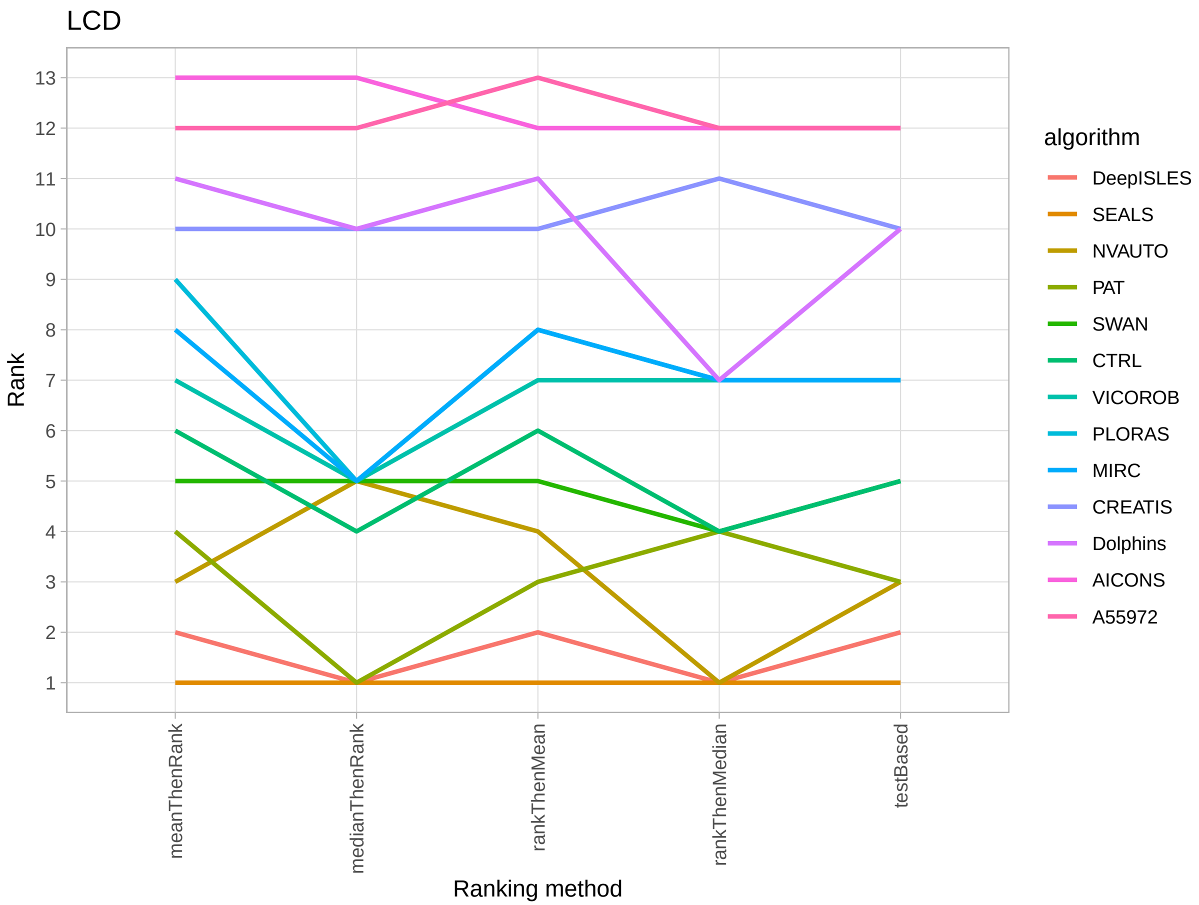

Figure S5.25: LCD Ranking Robustness Across Methods.

4 Visualization of cross-task insights

The algorithms are ordered according to consensus ranking.

4.1 Characterization of algorithms

4.1.1 Ranking stability: Variability of achieved rankings across tasks

Algorithms are color-coded, and the area of each blob at position $(A_i, \text{rank } j)$ is proportional to the relative frequency A_i achieved rank j across multiple tasks. The median rank for each algorithm is indicated by a black cross. This way, the distribution of ranks across tasks can be intuitively visualized.

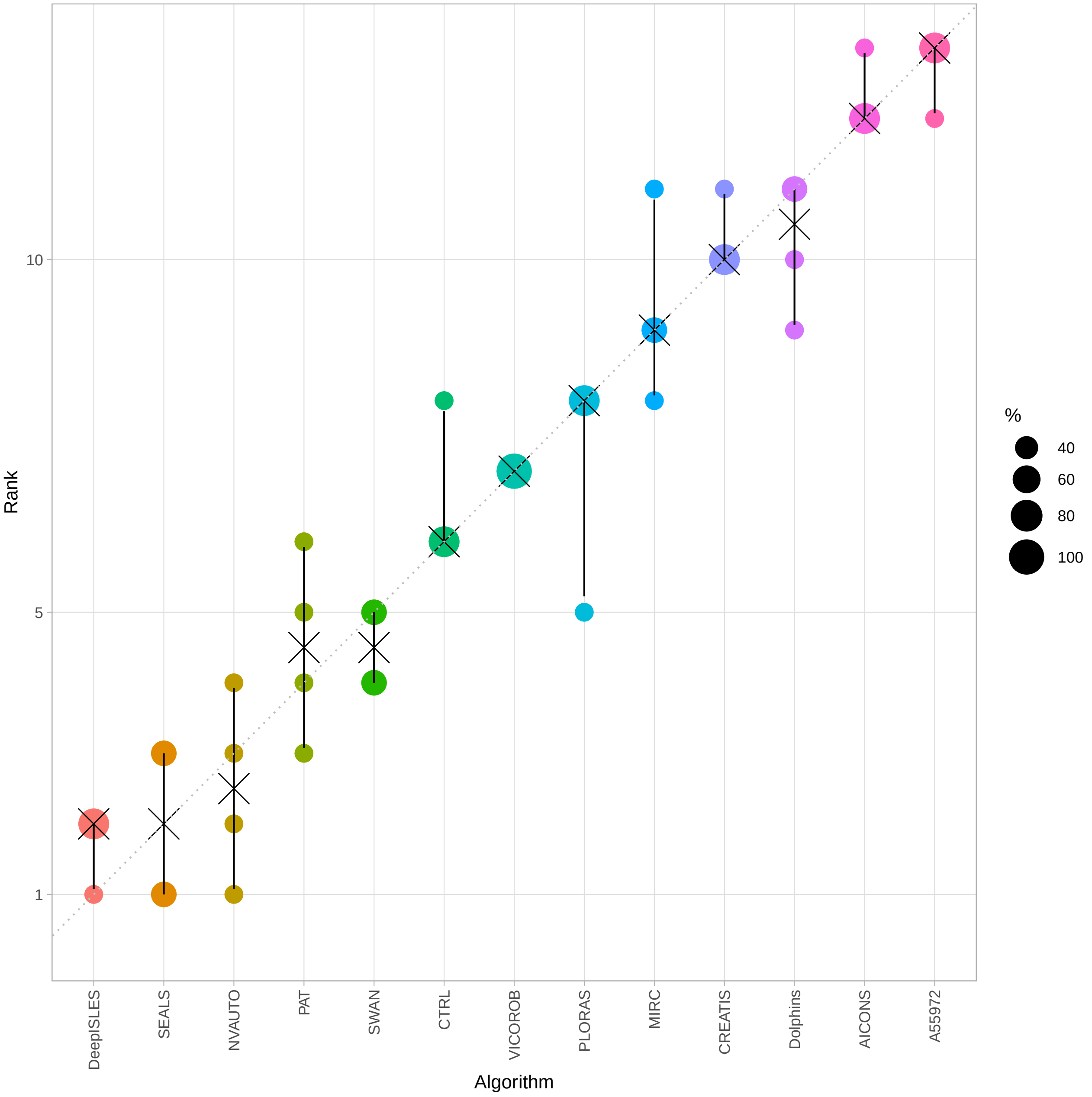

Figure S5.26: Cross-Task Ranking Stability – Blob Plot.

4.1.2 Ranking stability: Ranking variability via bootstrap approach

A blob plot of bootstrap results over the different tasks separated by algorithm allows another perspective on the assessment data. This gives deeper insights into the characteristics of tasks and the ranking uncertainty of the algorithms in each task.

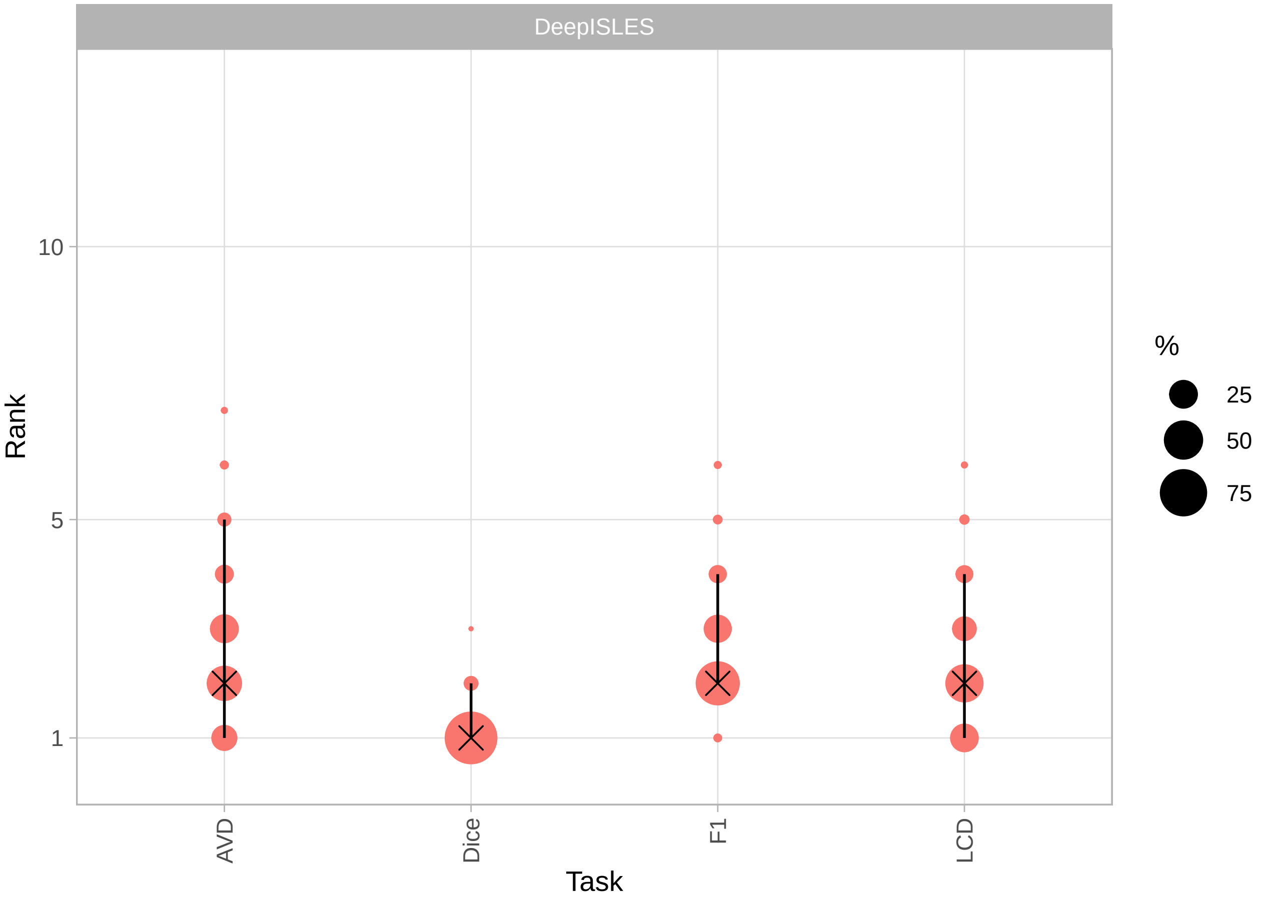

Figure S5.27: Bootstrap Ranking Stability – DeepISLES.

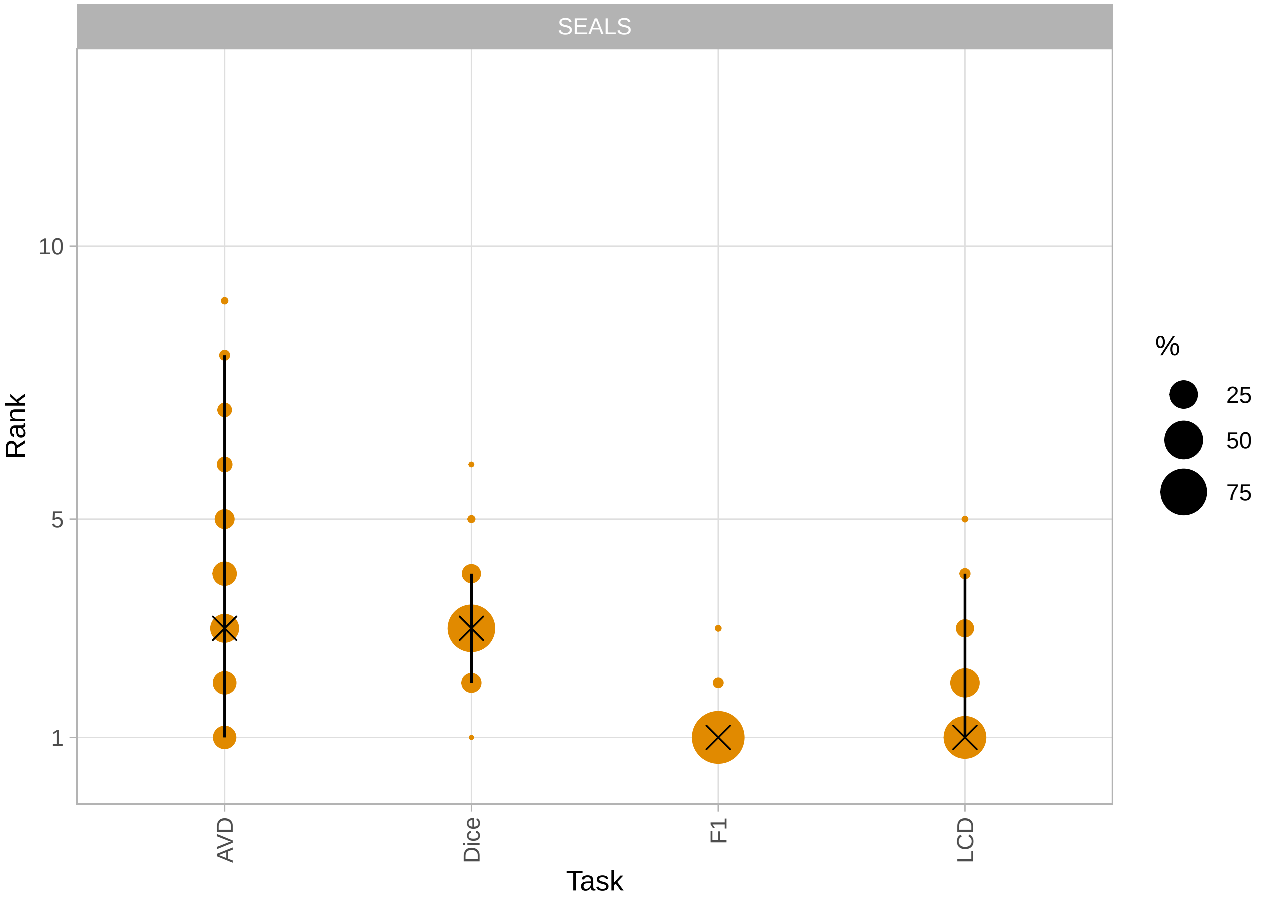

Figure S5.28: Bootstrap Ranking Stability – SEALS.

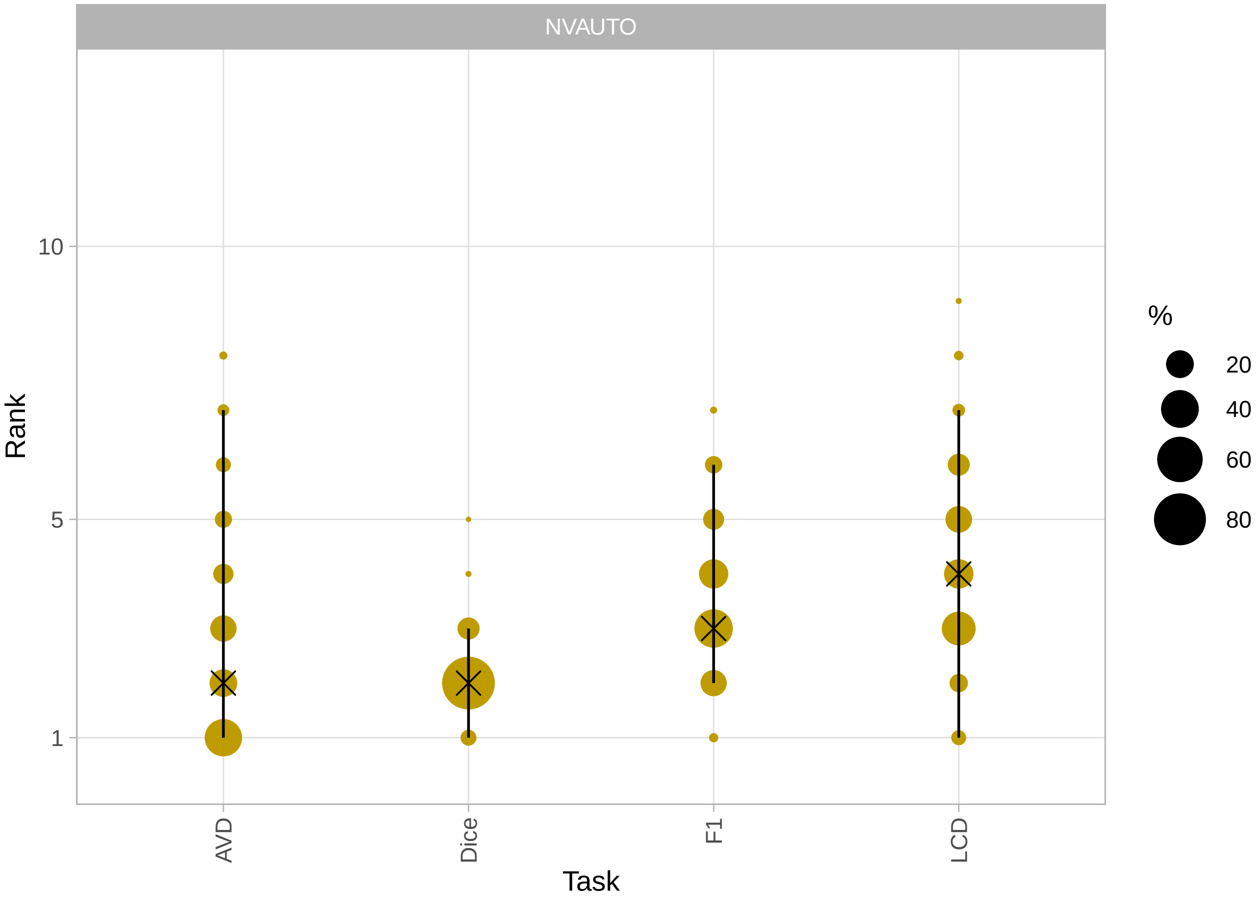

Figure S5.29: Bootstrap Ranking Stability – NVAUTO.

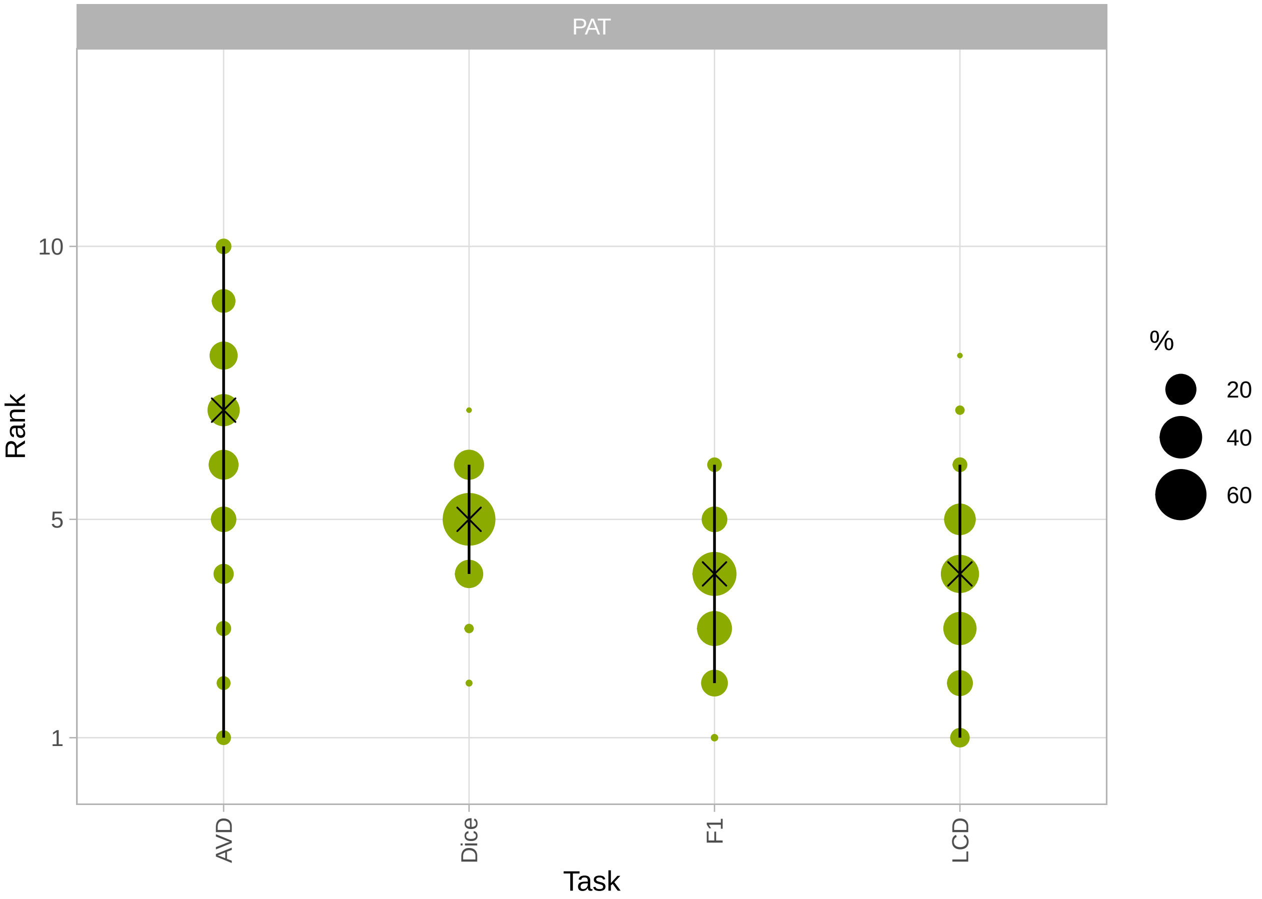

Figure S5.30: Bootstrap Ranking Stability – PAT.

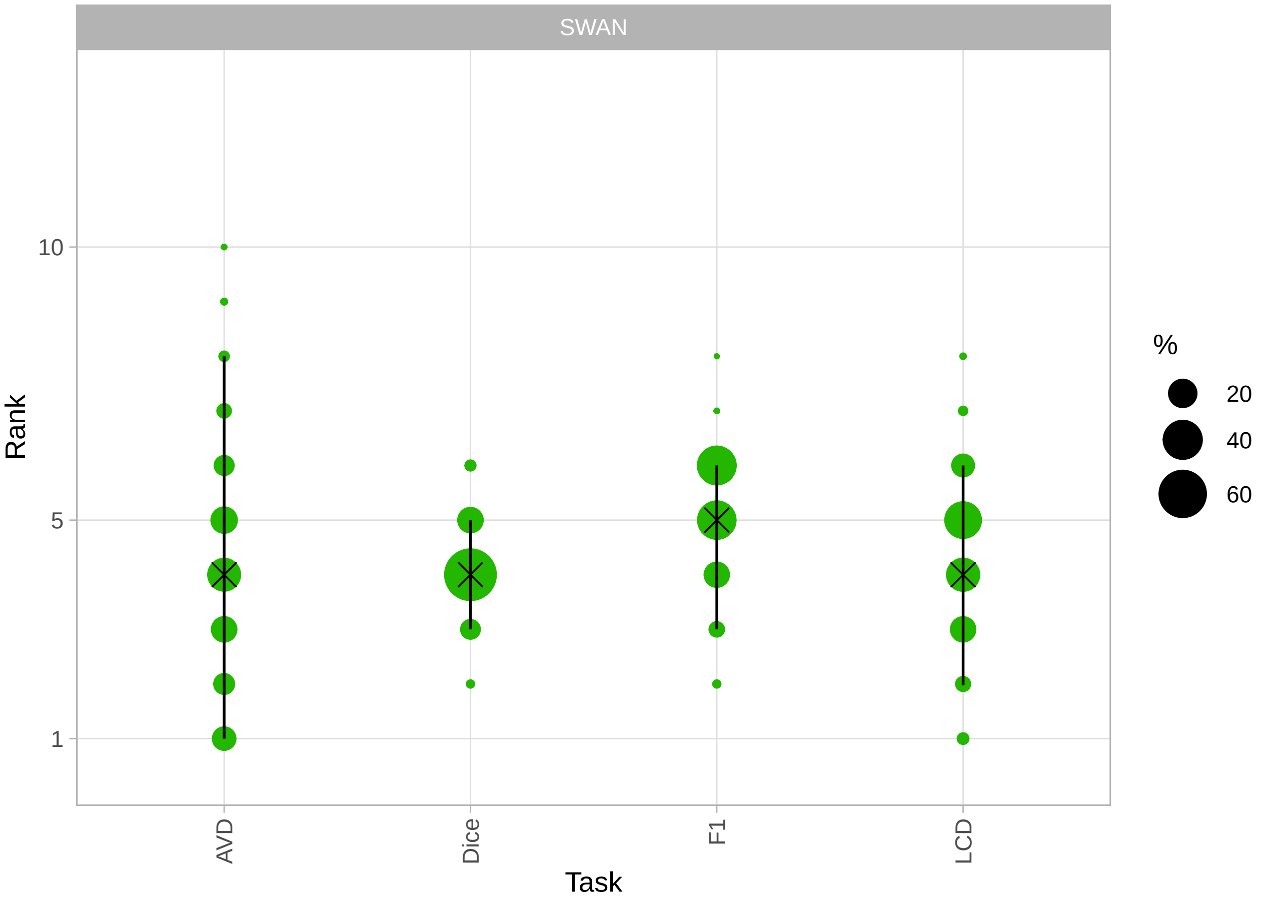

Figure S5.31: Bootstrap Ranking Stability – SWAN.

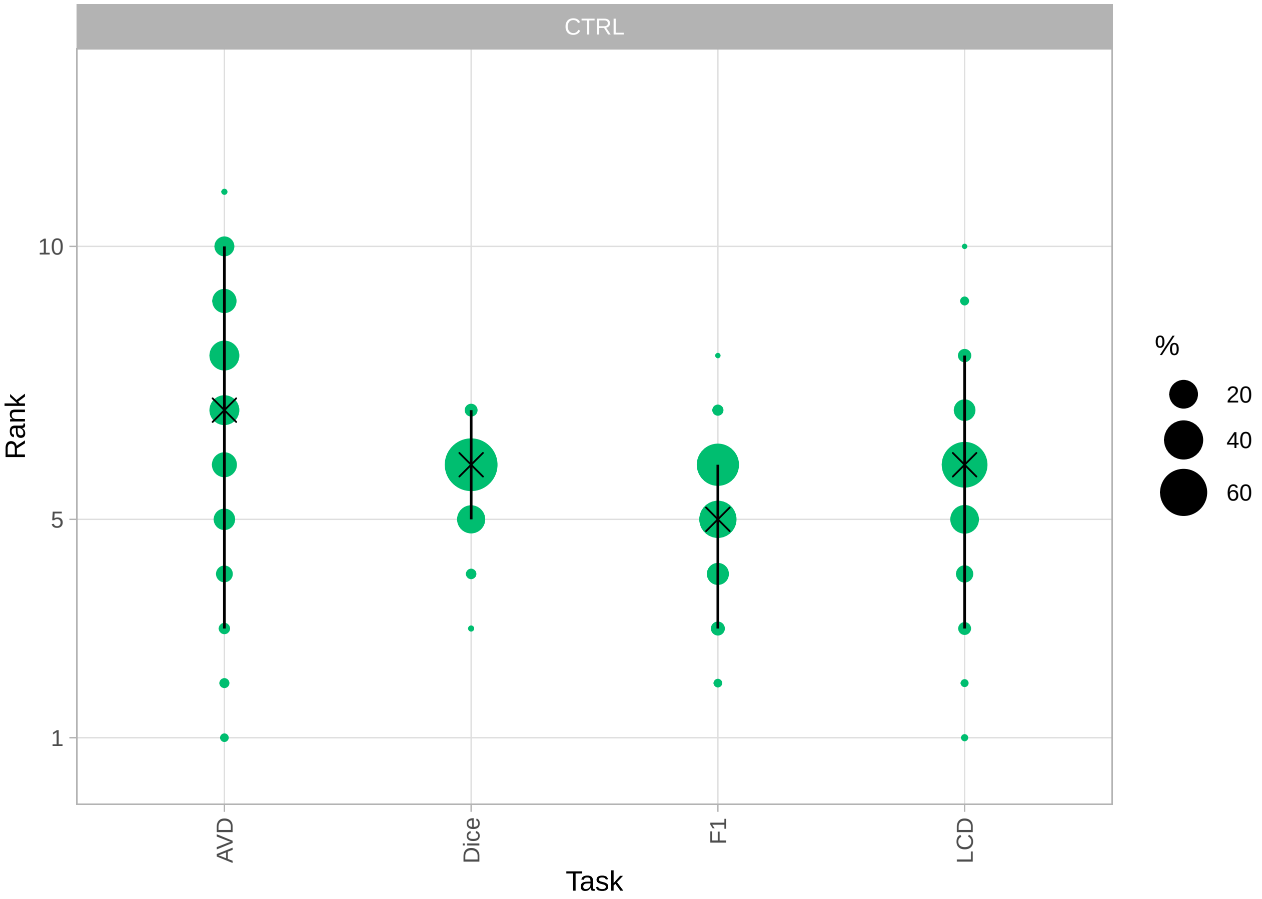

Figure S5.32: Bootstrap Ranking Stability – CTRL.

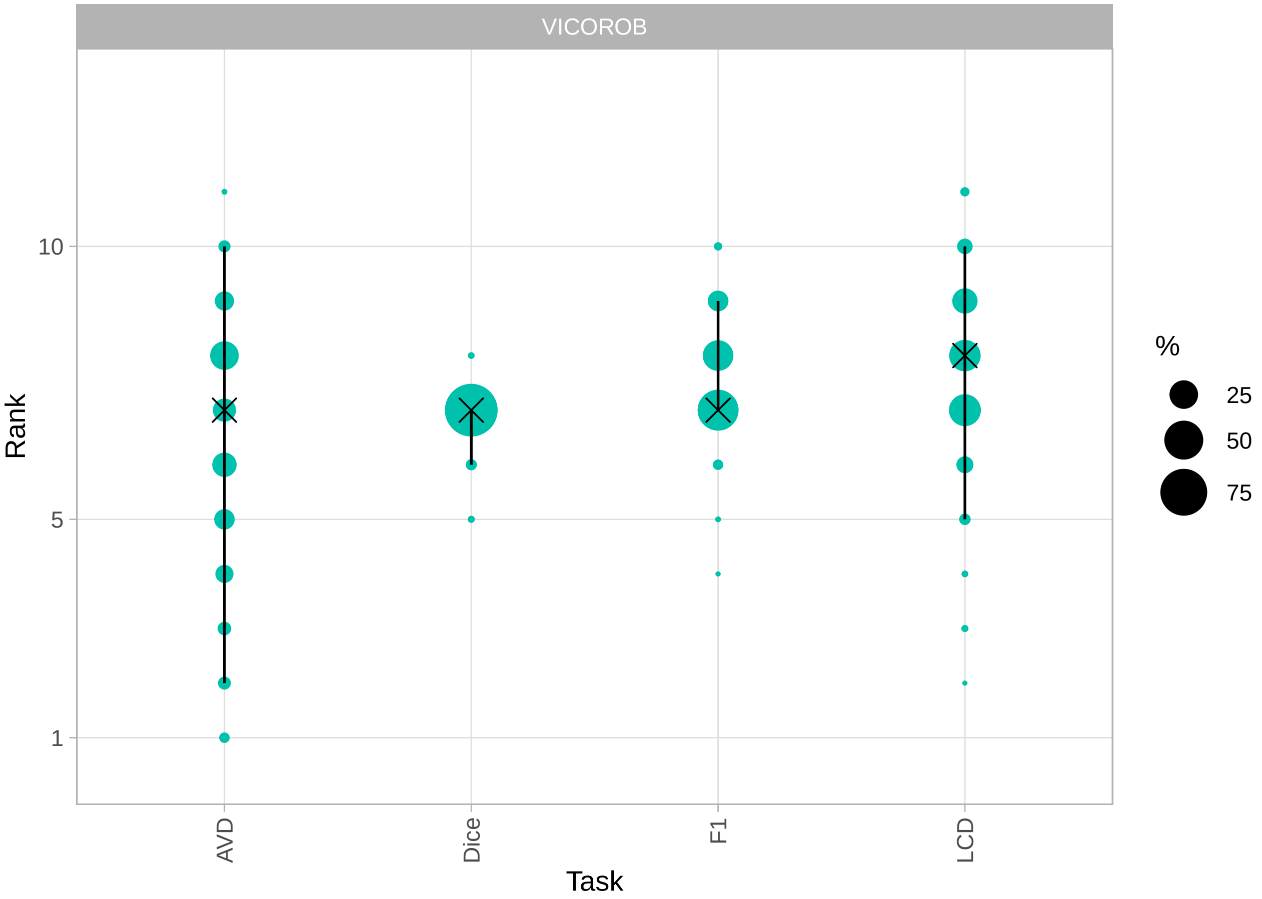

Figure S5.33: Bootstrap Ranking Stability – VICOROB.

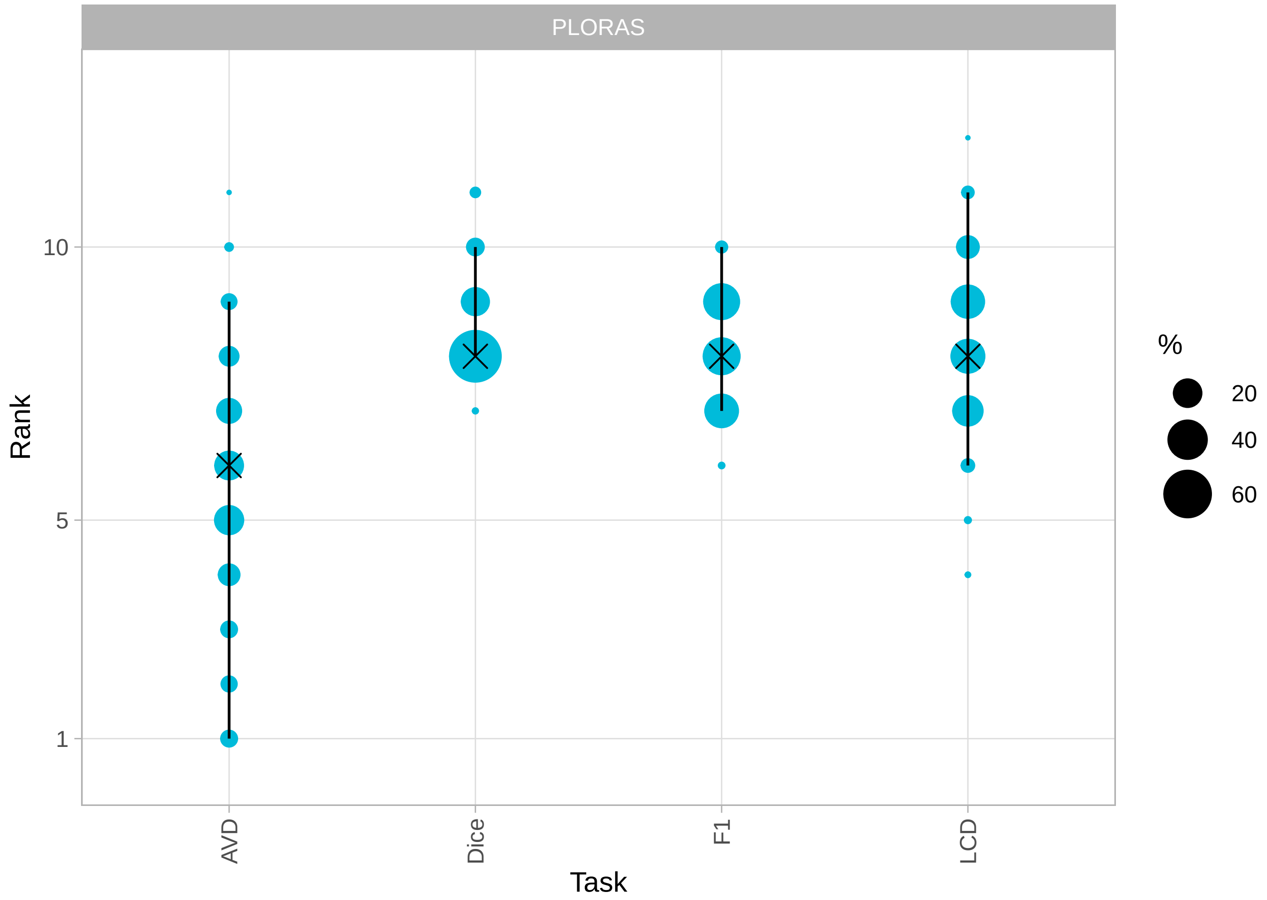

Figure S5.34: Bootstrap Ranking Stability – PLORAS.

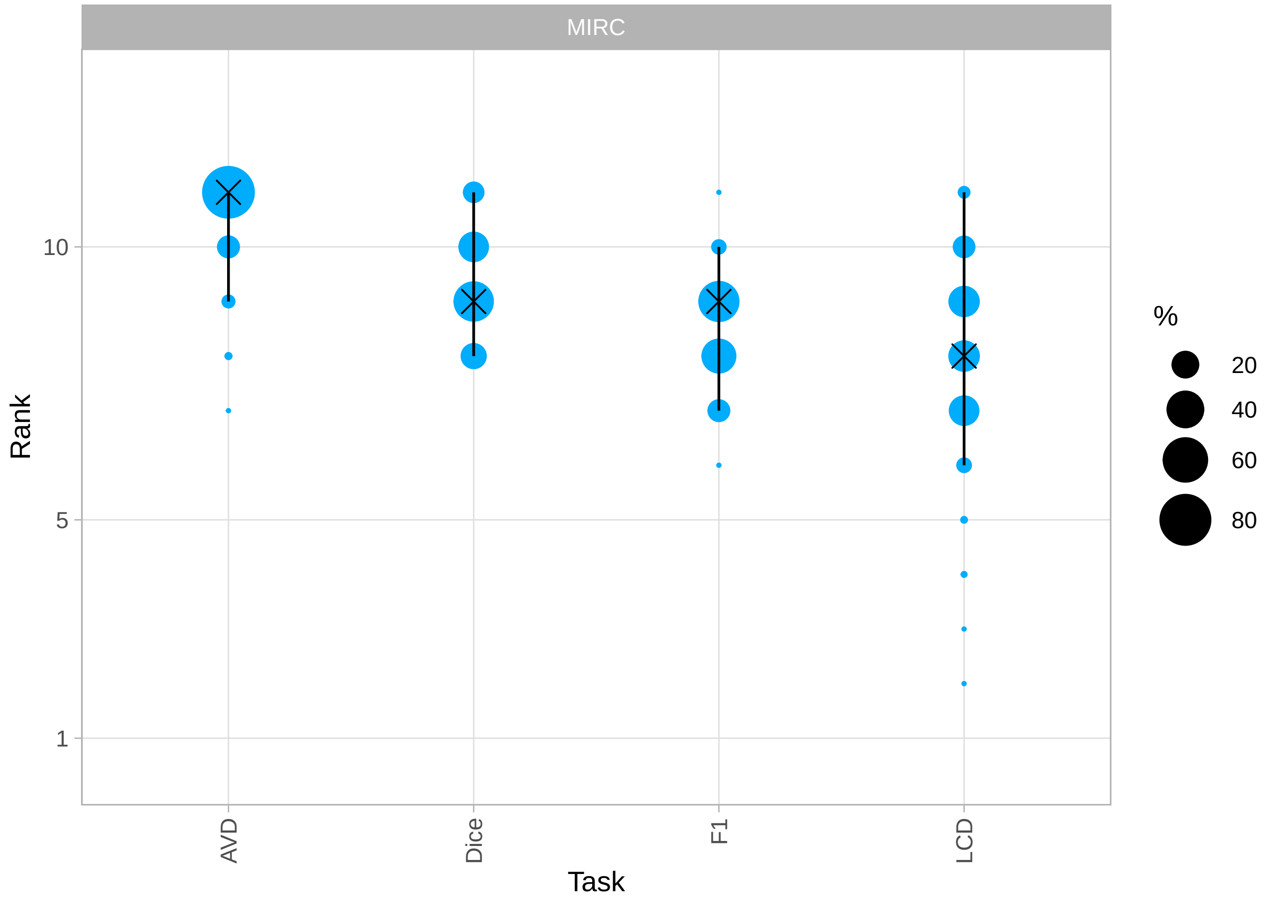

Figure S5.35: Bootstrap Ranking Stability – MIRC.

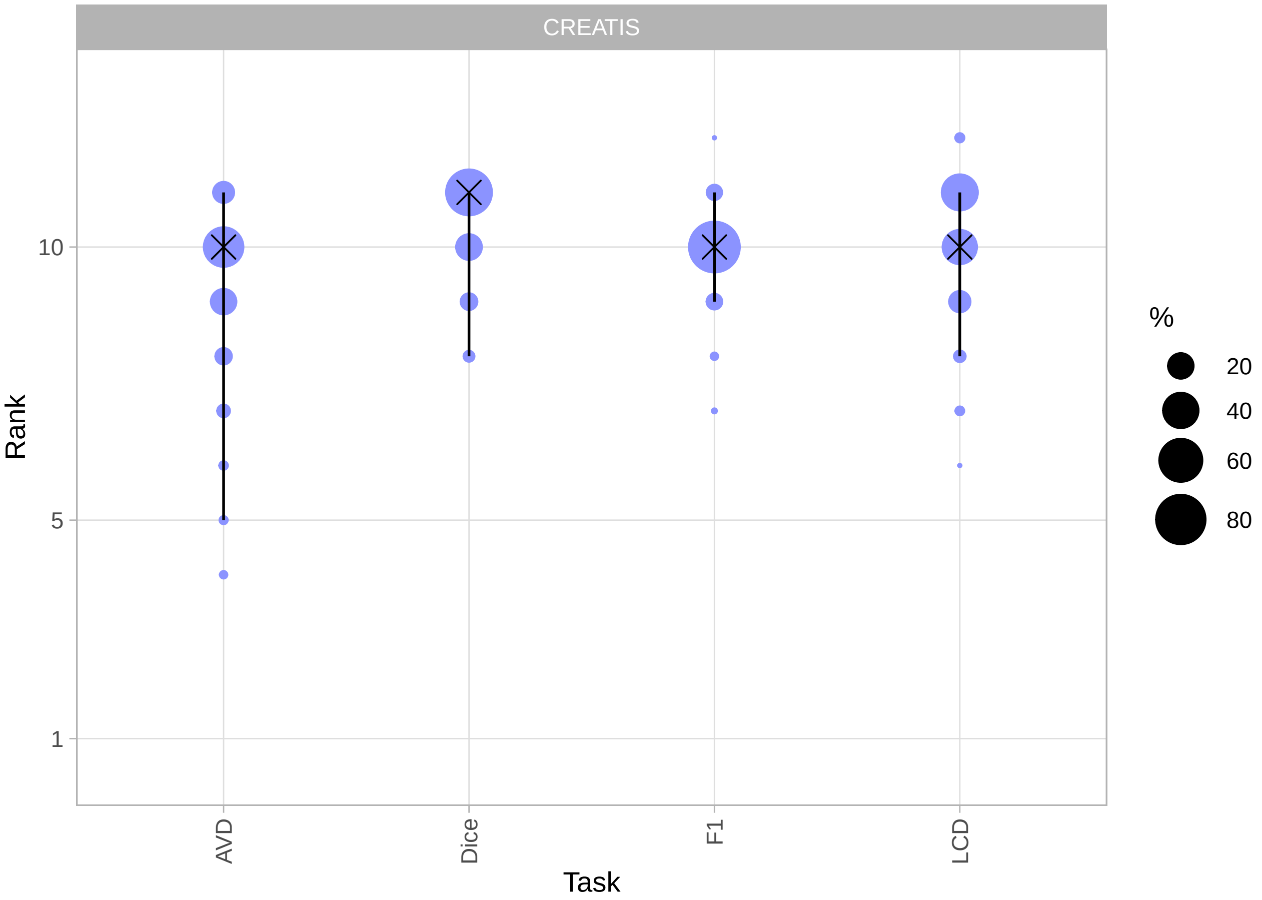

Figure S5.36: Bootstrap Ranking Stability – CREATIS.

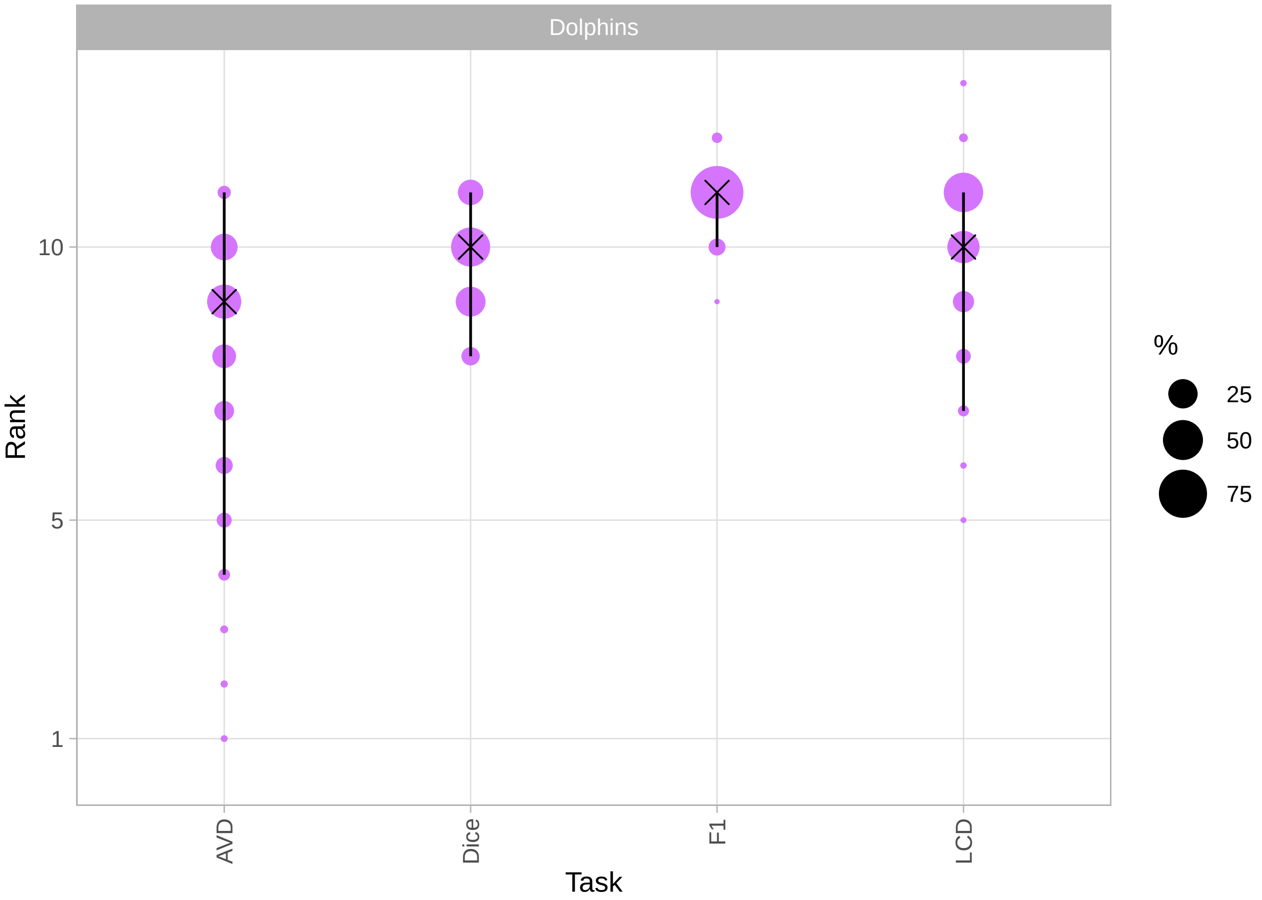

Figure S5.37: Bootstrap Ranking Stability – Dolphins.

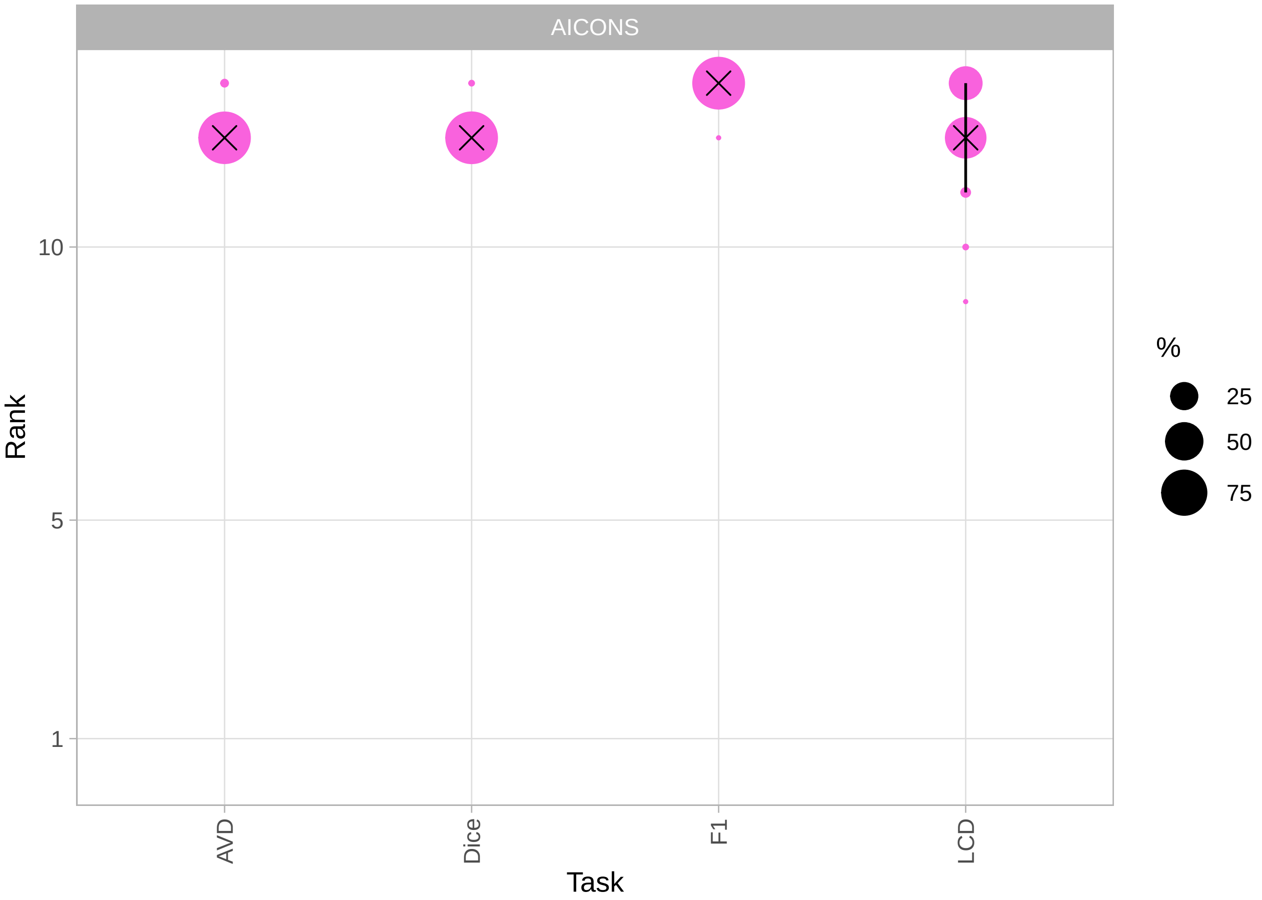

Figure S5.38: Bootstrap Ranking Stability – AICONS.

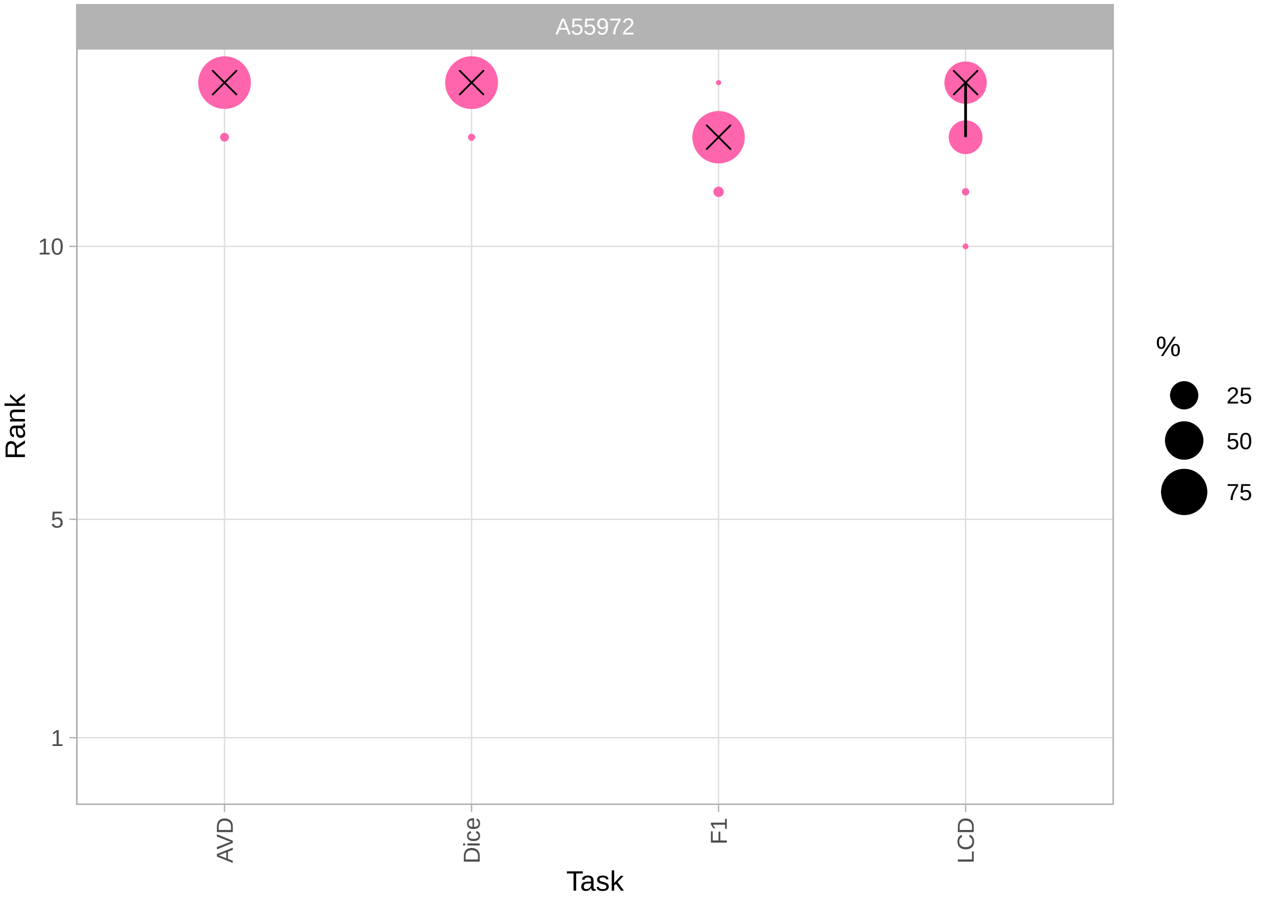

Figure S5.39: Bootstrap Ranking Stability – A55972.

An alternative representation is provided by a stacked frequency plot of the observed ranks, separated by algorithm. Observed ranks across bootstrap samples are displayed with coloring according to the task. For algorithms that achieve the same rank in different tasks for the full assessment data set, vertical lines are on top of each other. Vertical lines allow to compare the achieved rank of each algorithm over different tasks.

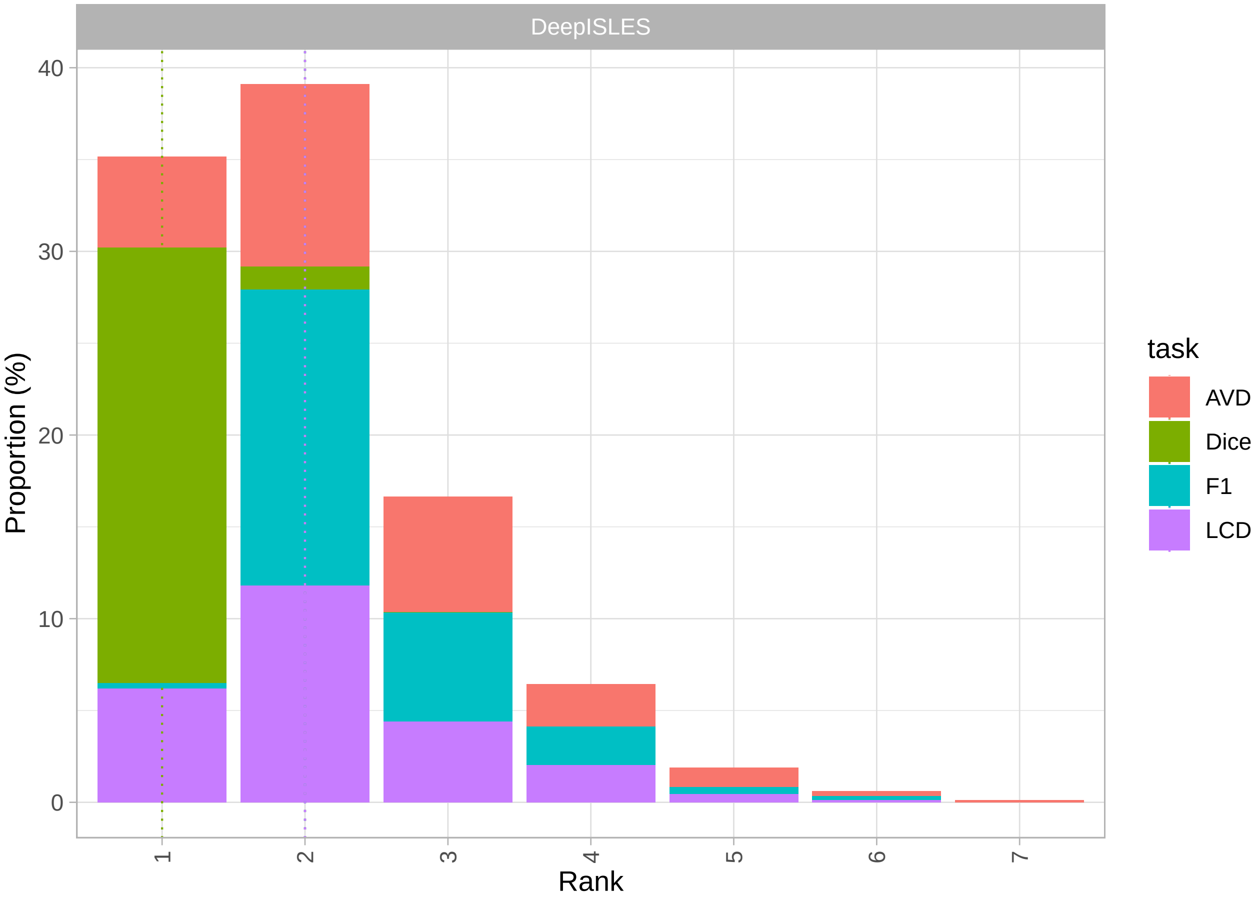

Figure S5.40: Stacked Rank Frequencies – DeepISLES.

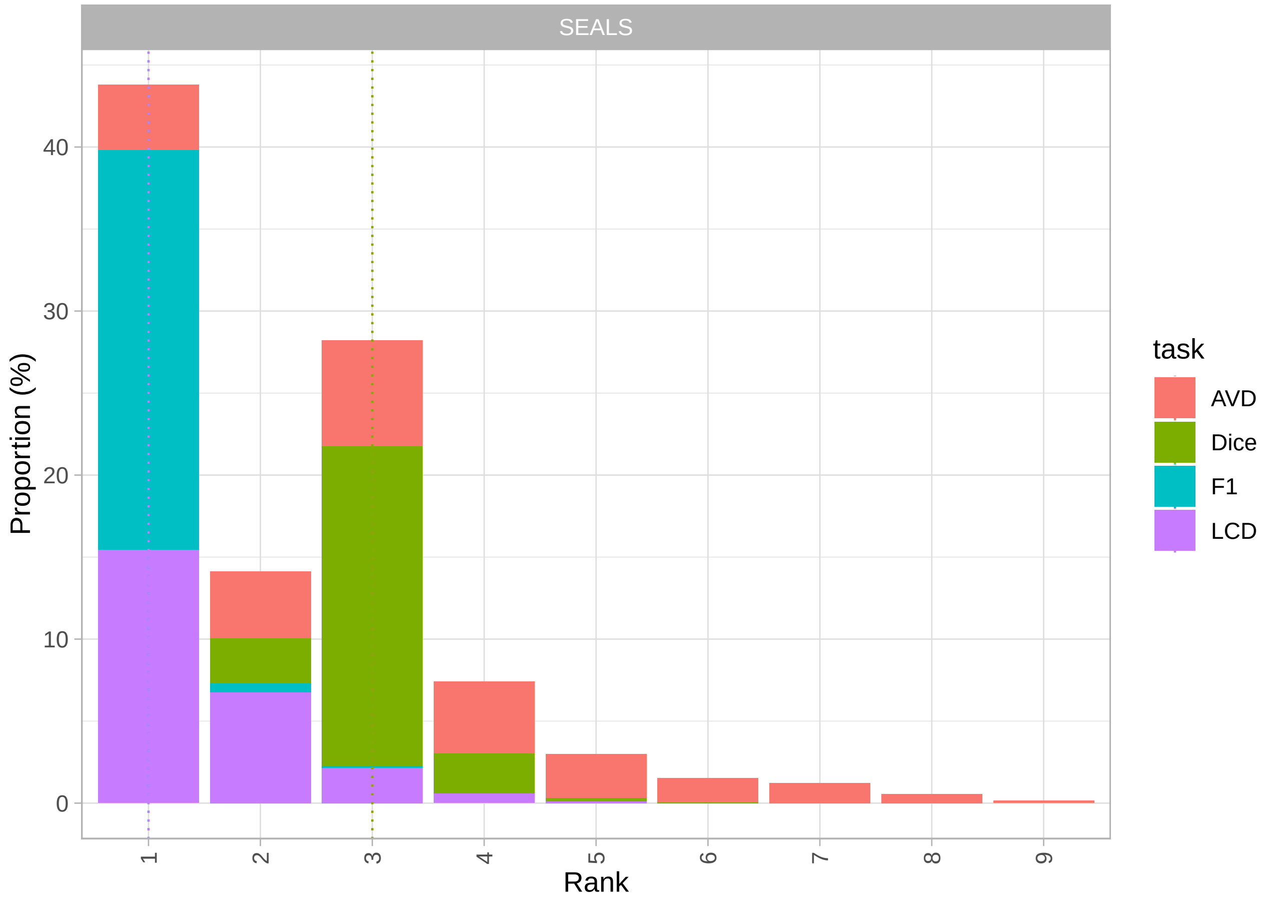

Figure S5.41: Stacked Rank Frequencies – SEALS.

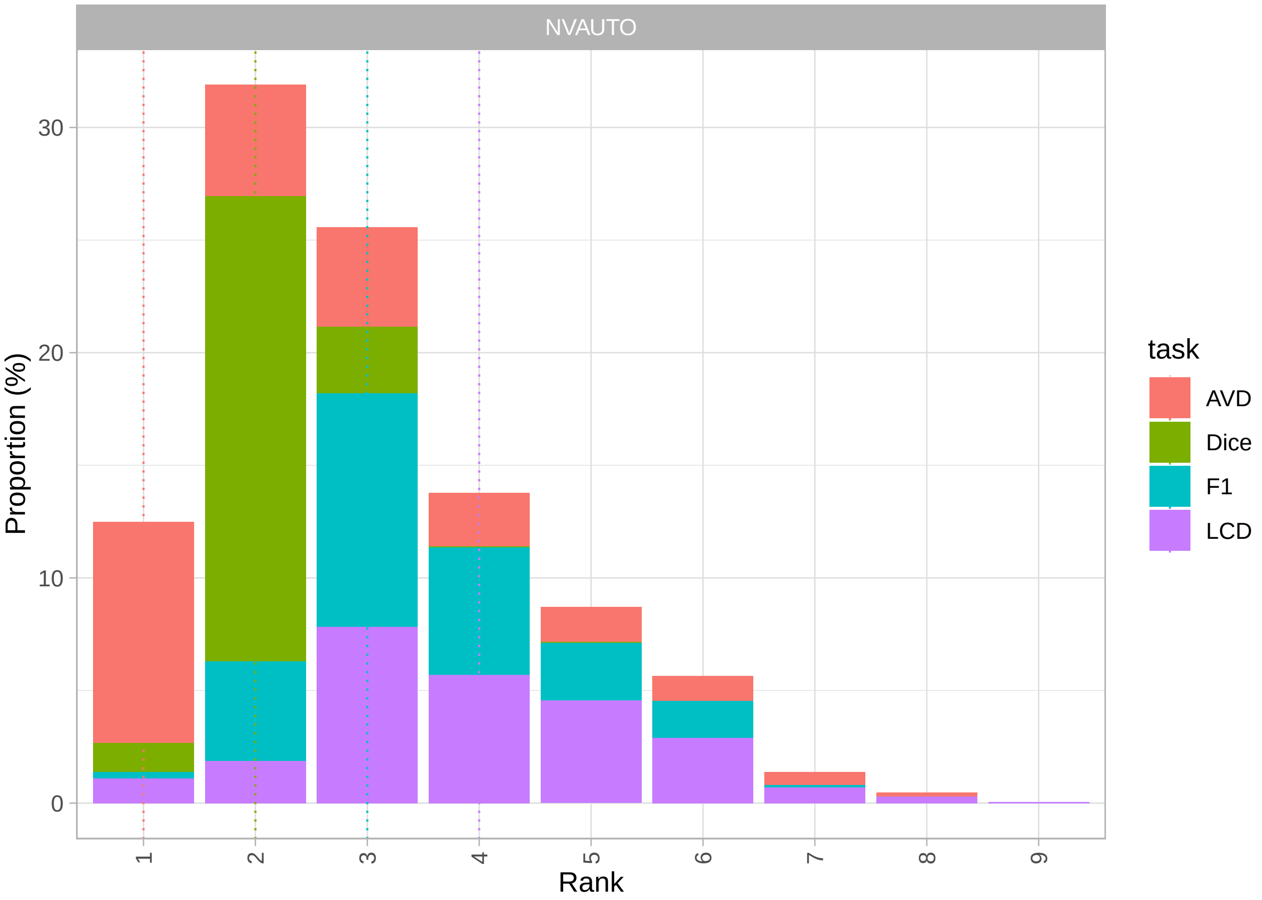

Figure S5.42: Stacked Rank Frequencies – NVAUTO.

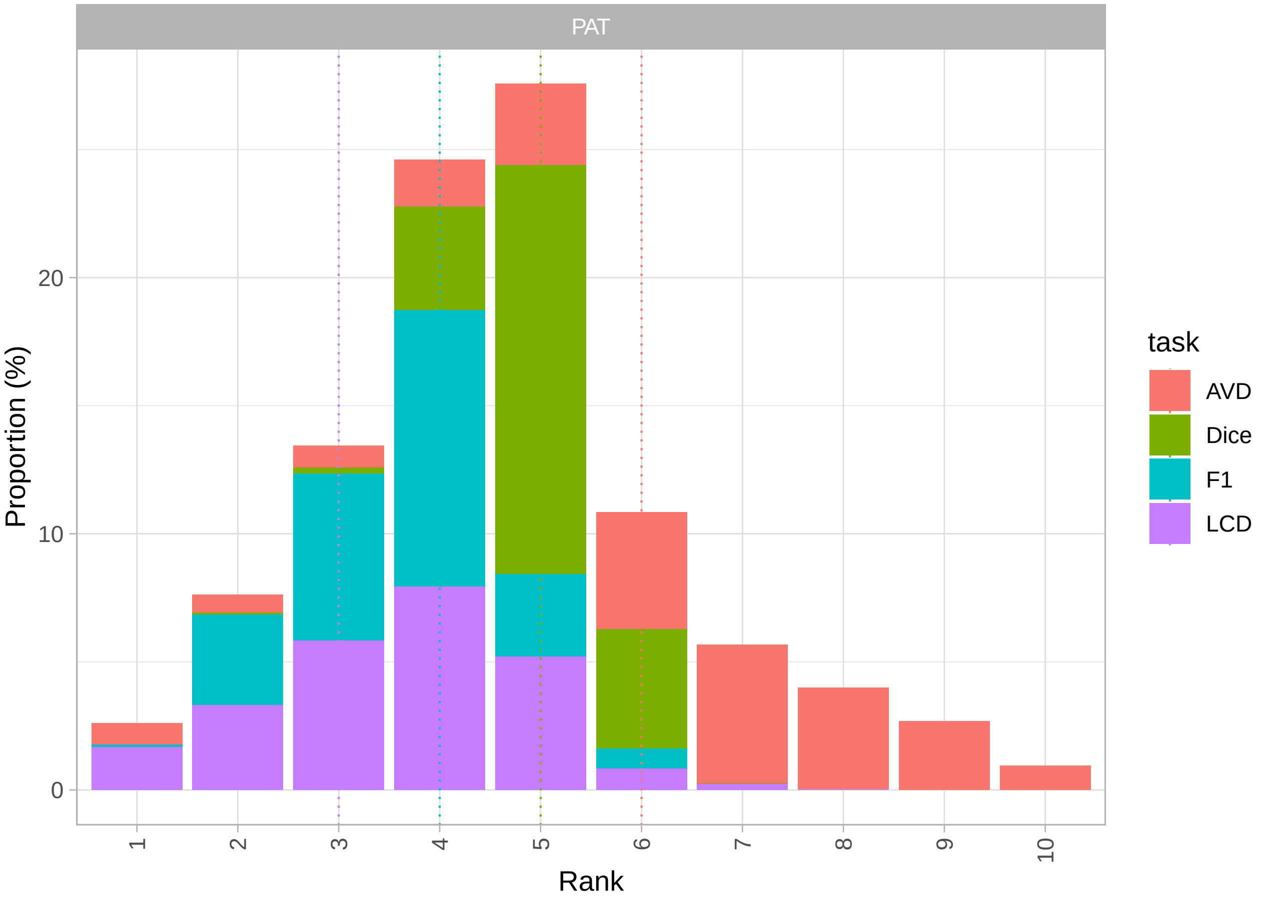

Figure S5.43: Stacked Rank Frequencies – PAT.

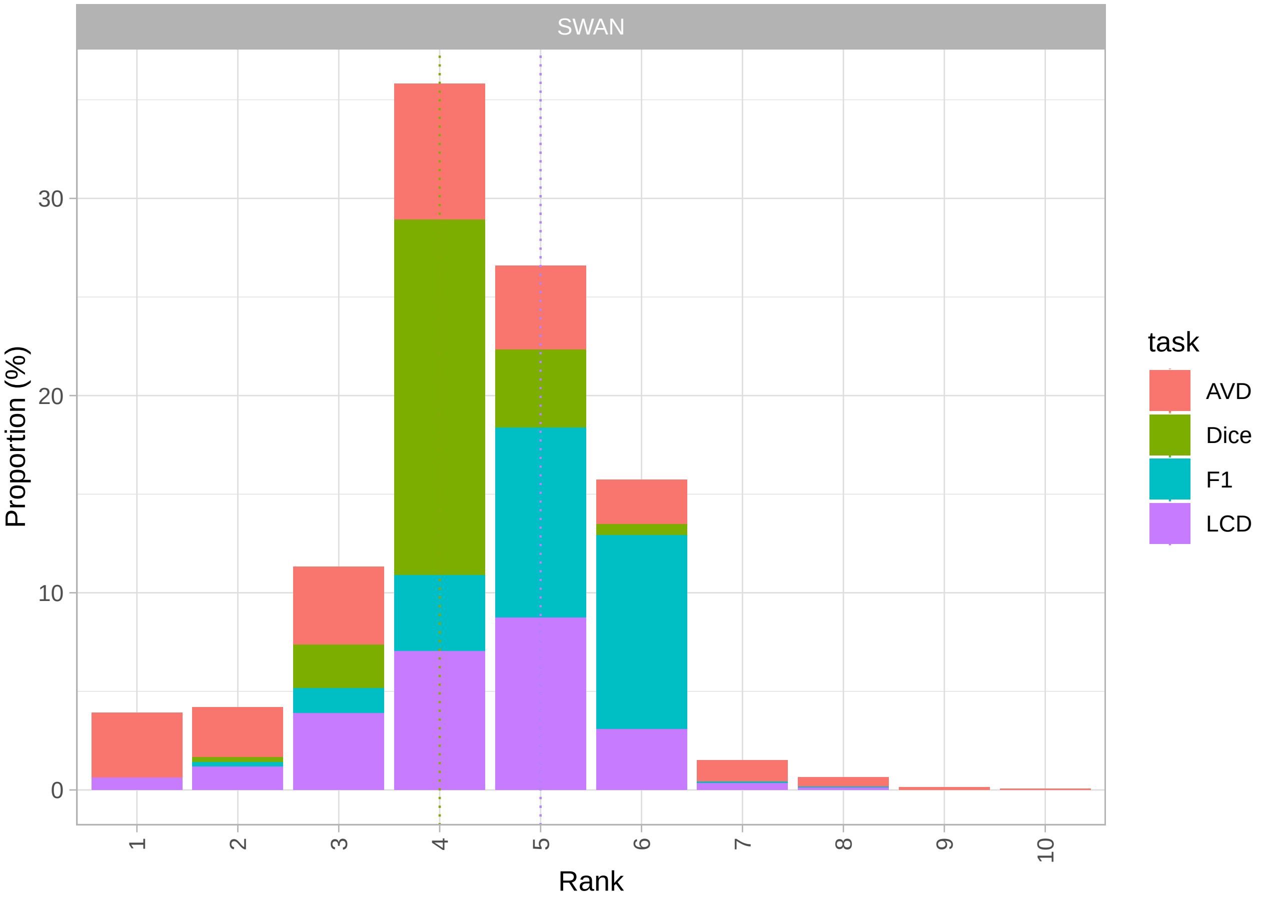

Figure S5.44: Stacked Rank Frequencies – SWAN.

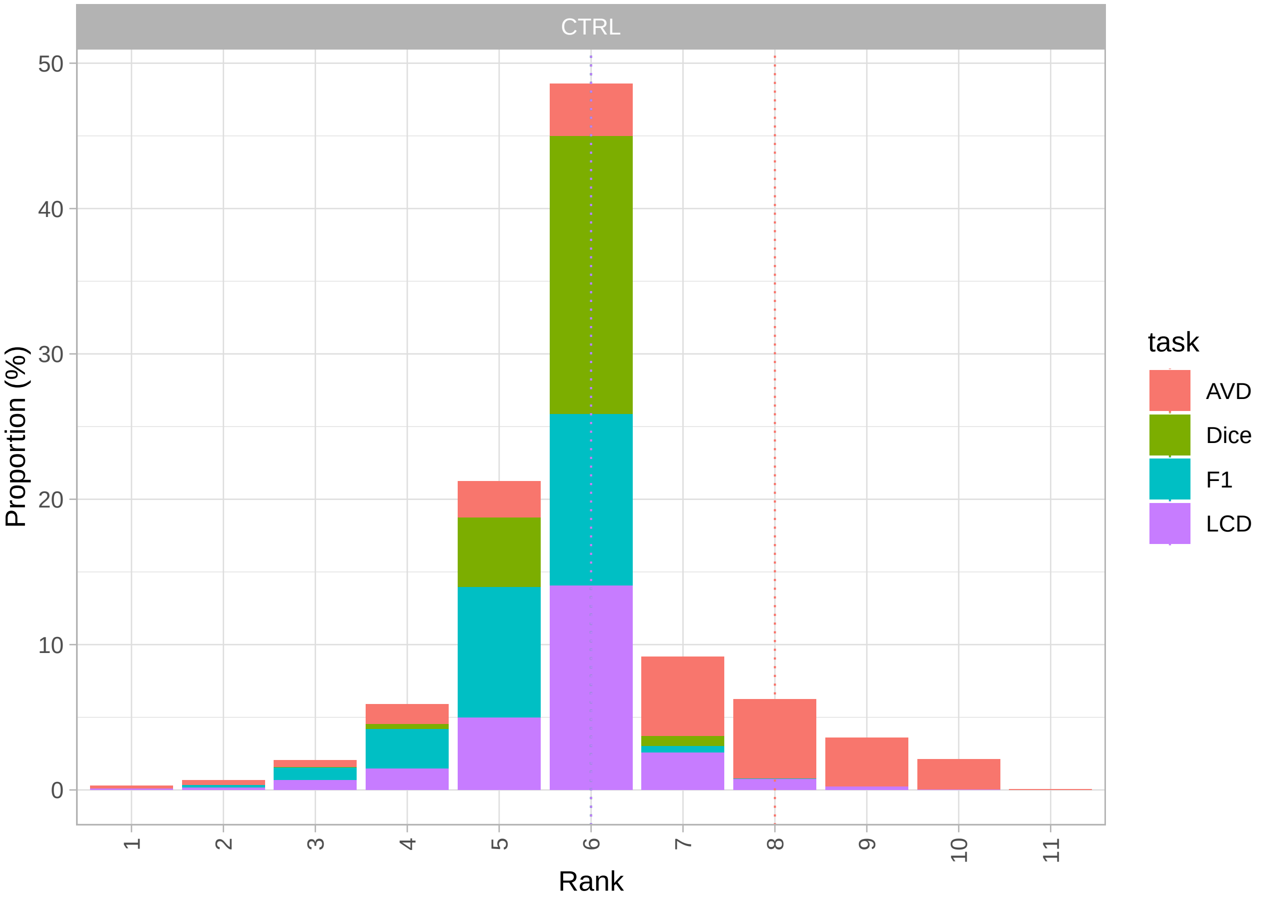

Figure S5.45: Stacked Rank Frequencies – CTRL.

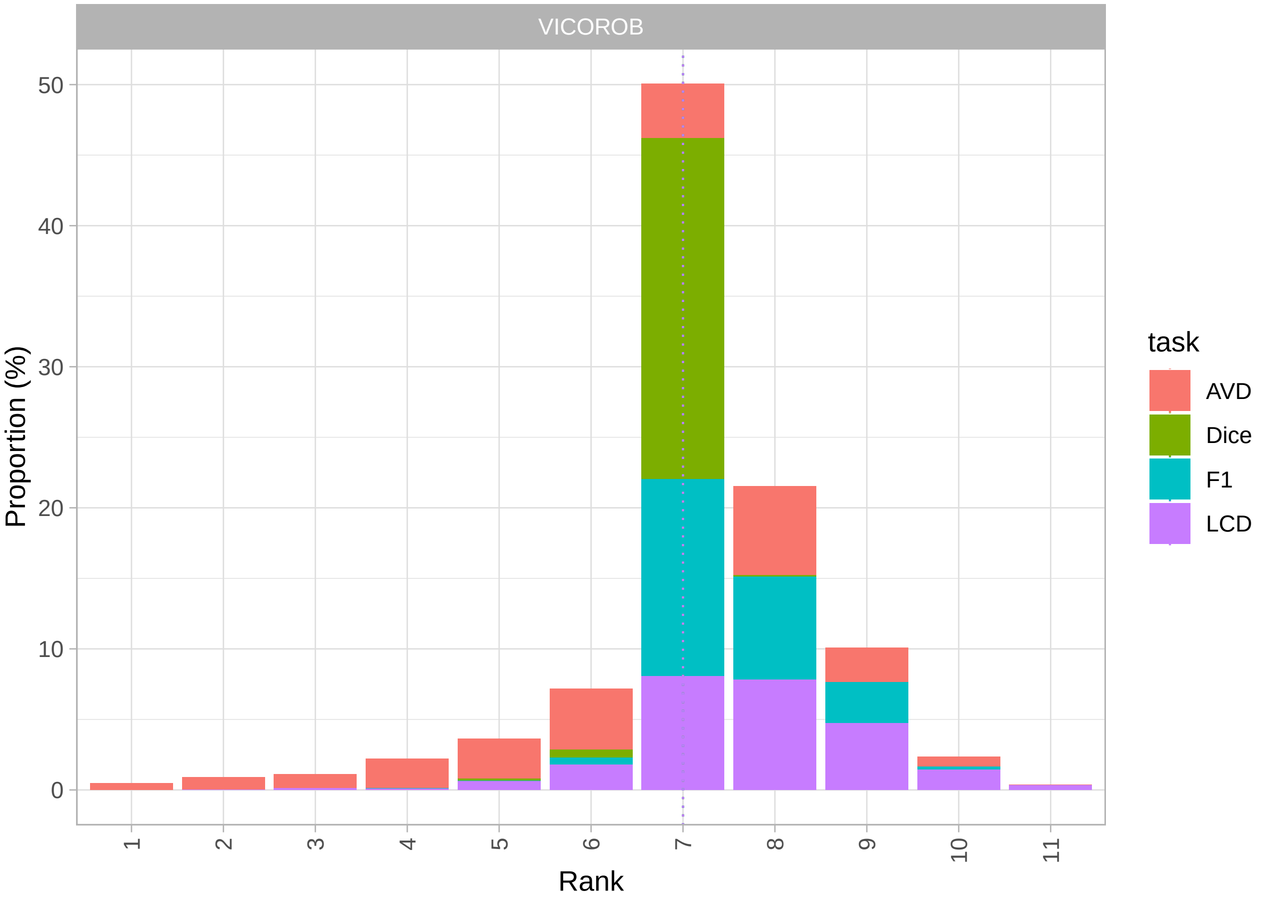

Figure S5.46: Stacked Rank Frequencies – VICOROB.

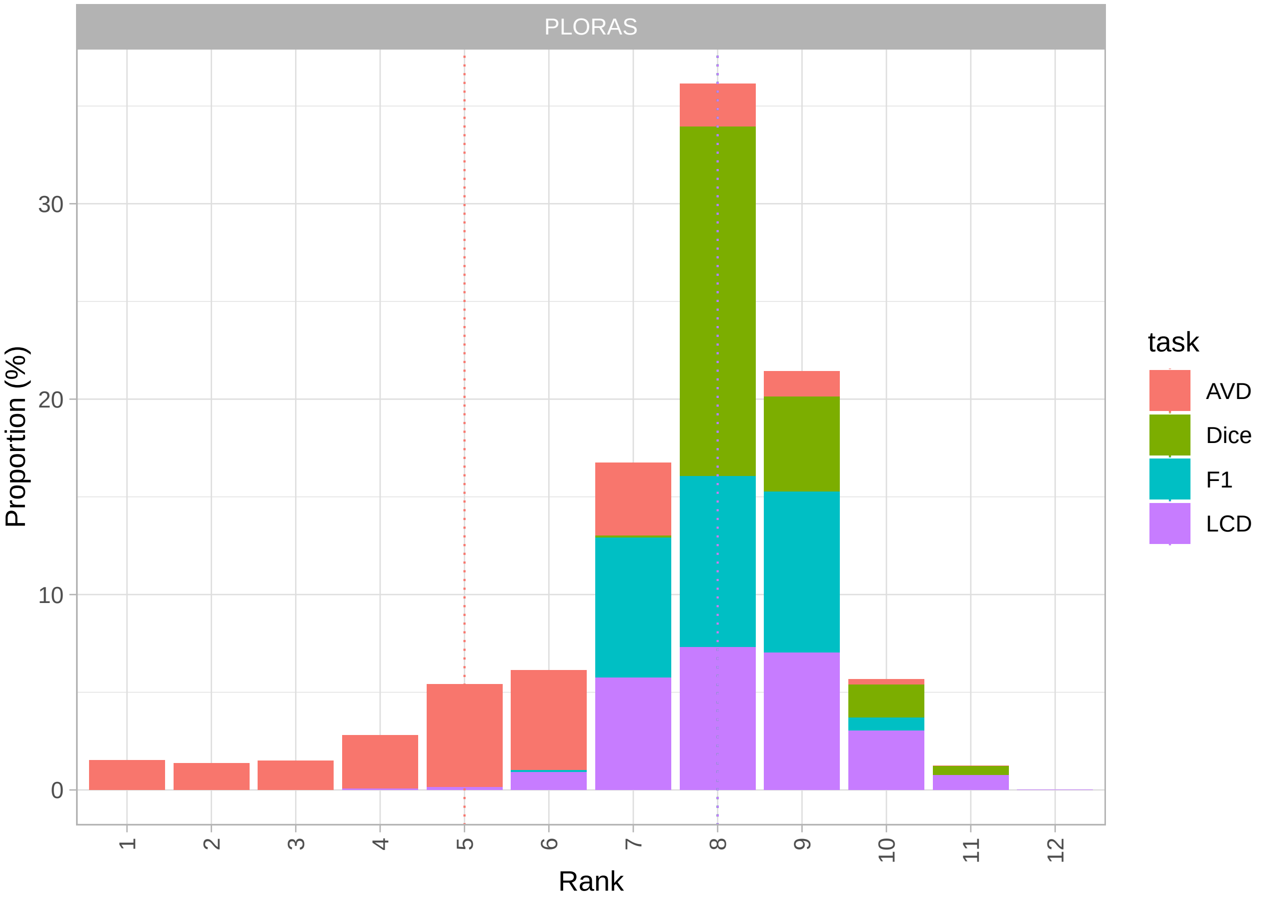

Figure S5.47: Stacked Rank Frequencies – PLORAS.

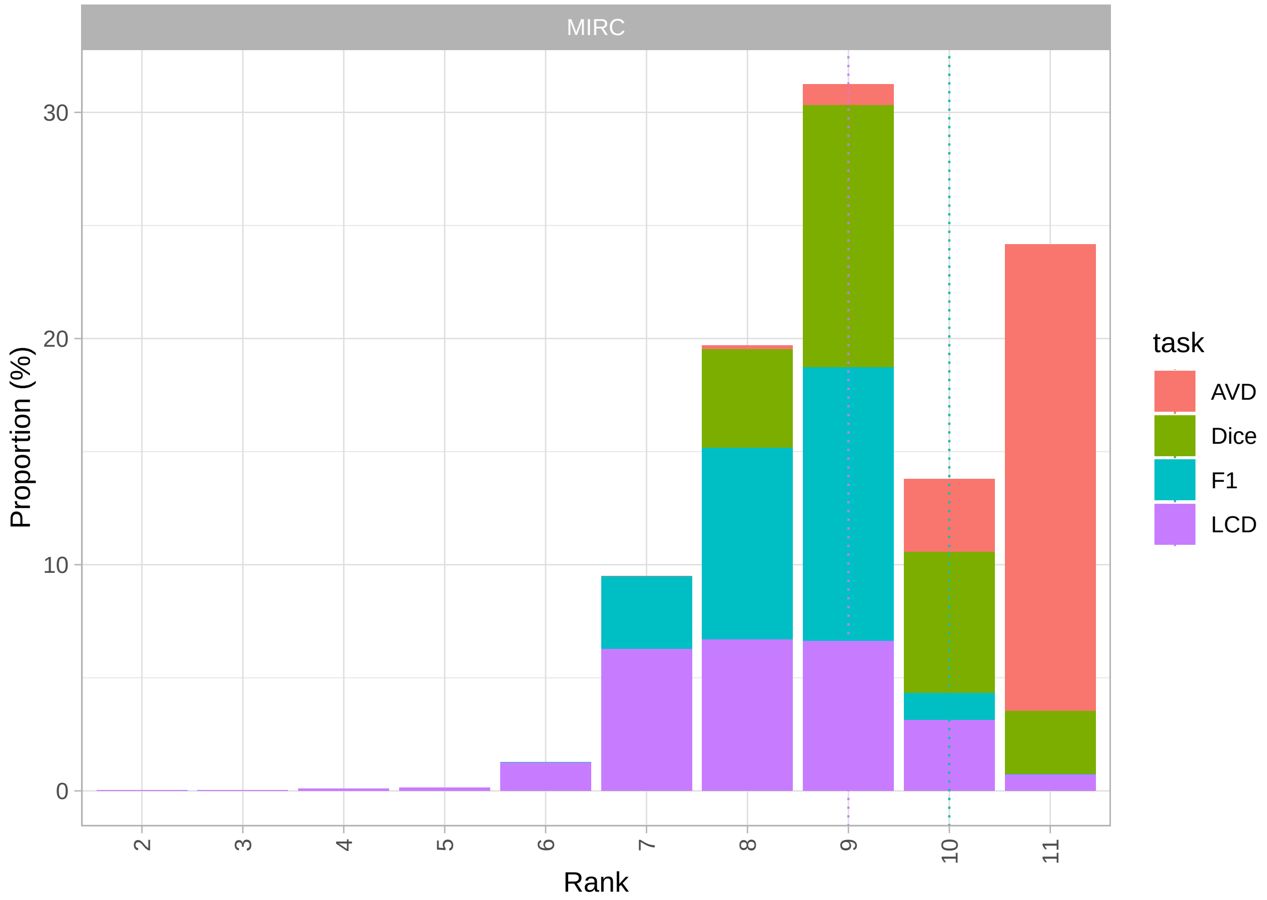

Figure S5.48: Stacked Rank Frequencies – MIRC.

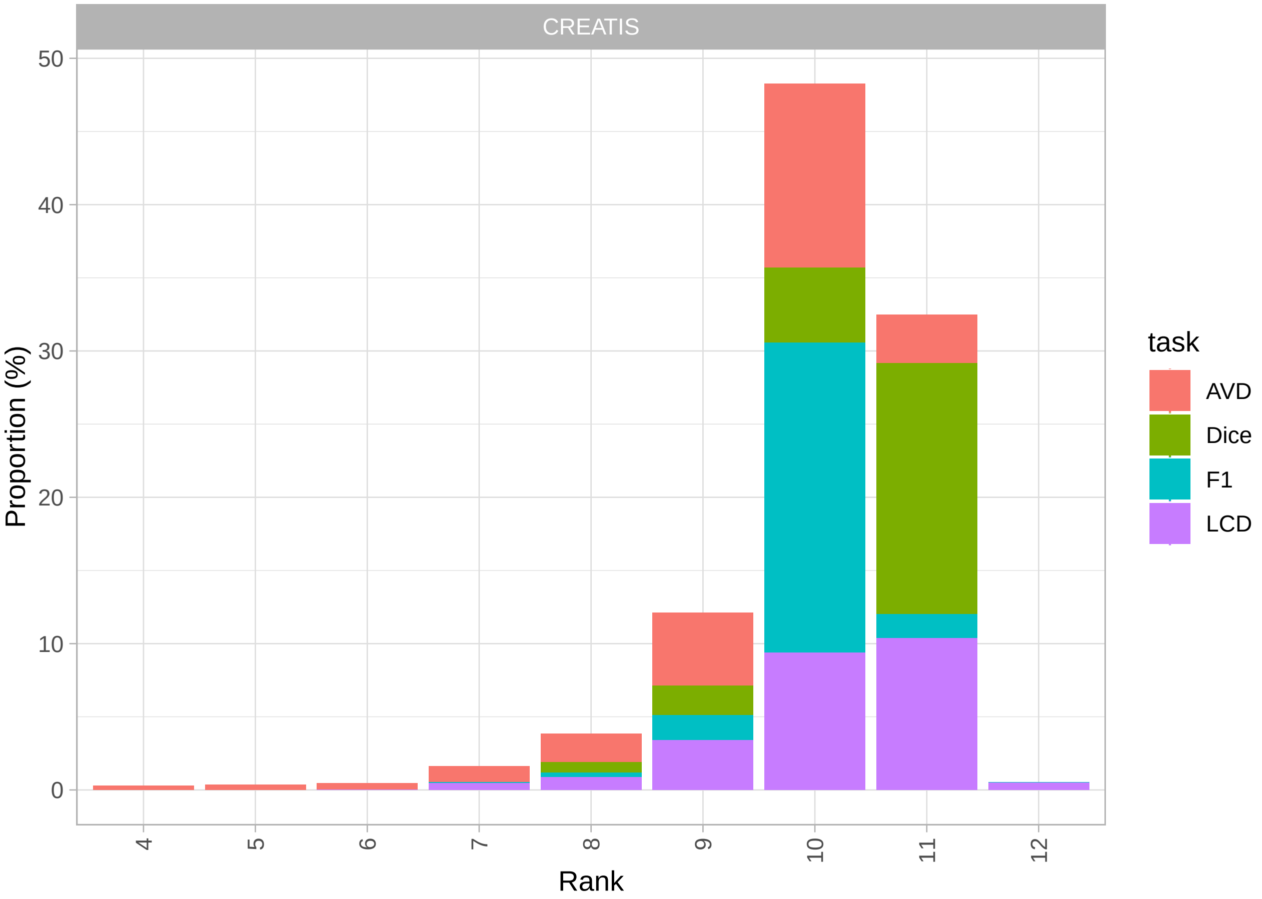

Figure S5.49: Stacked Rank Frequencies – CREATIS.

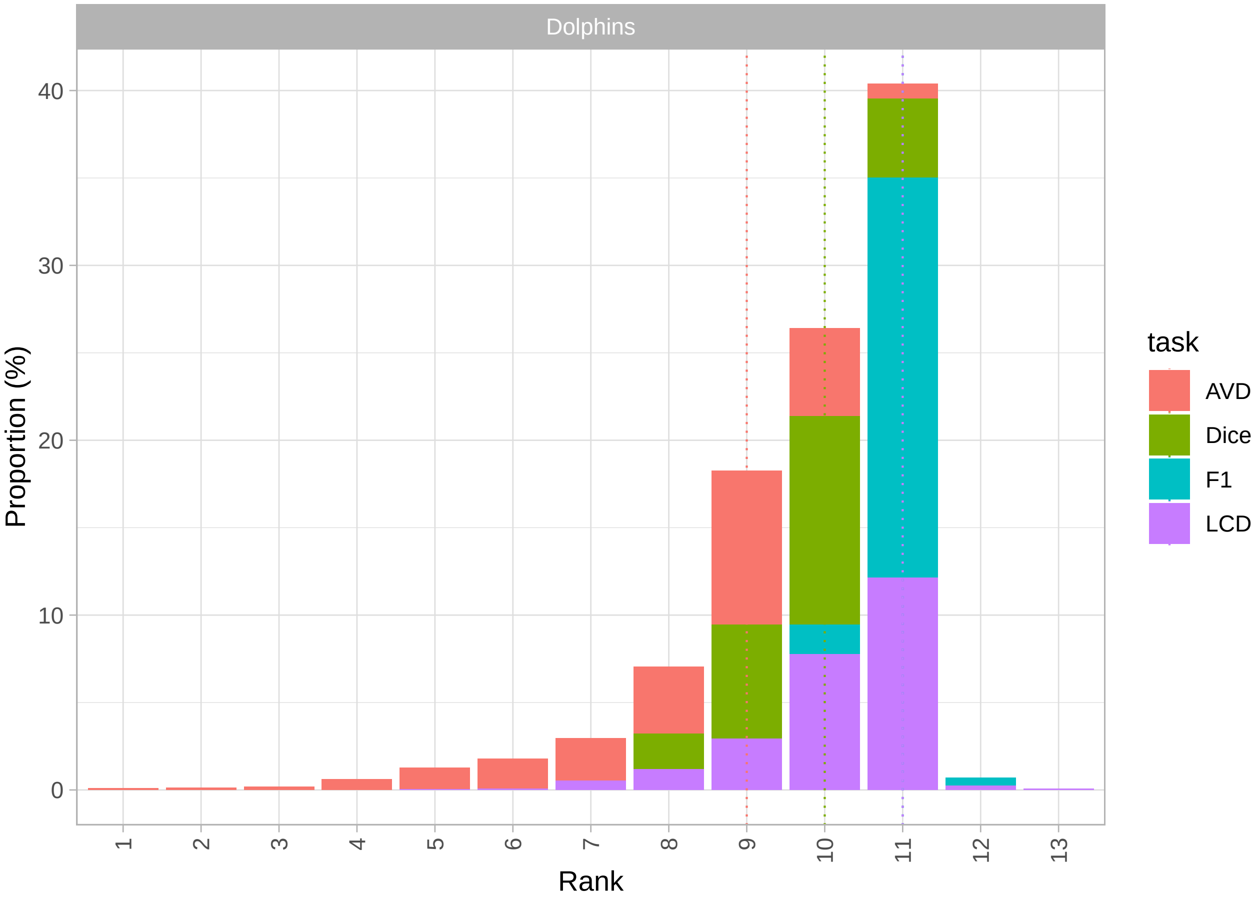

Figure S5.50: Stacked Rank Frequencies – Dolphins.

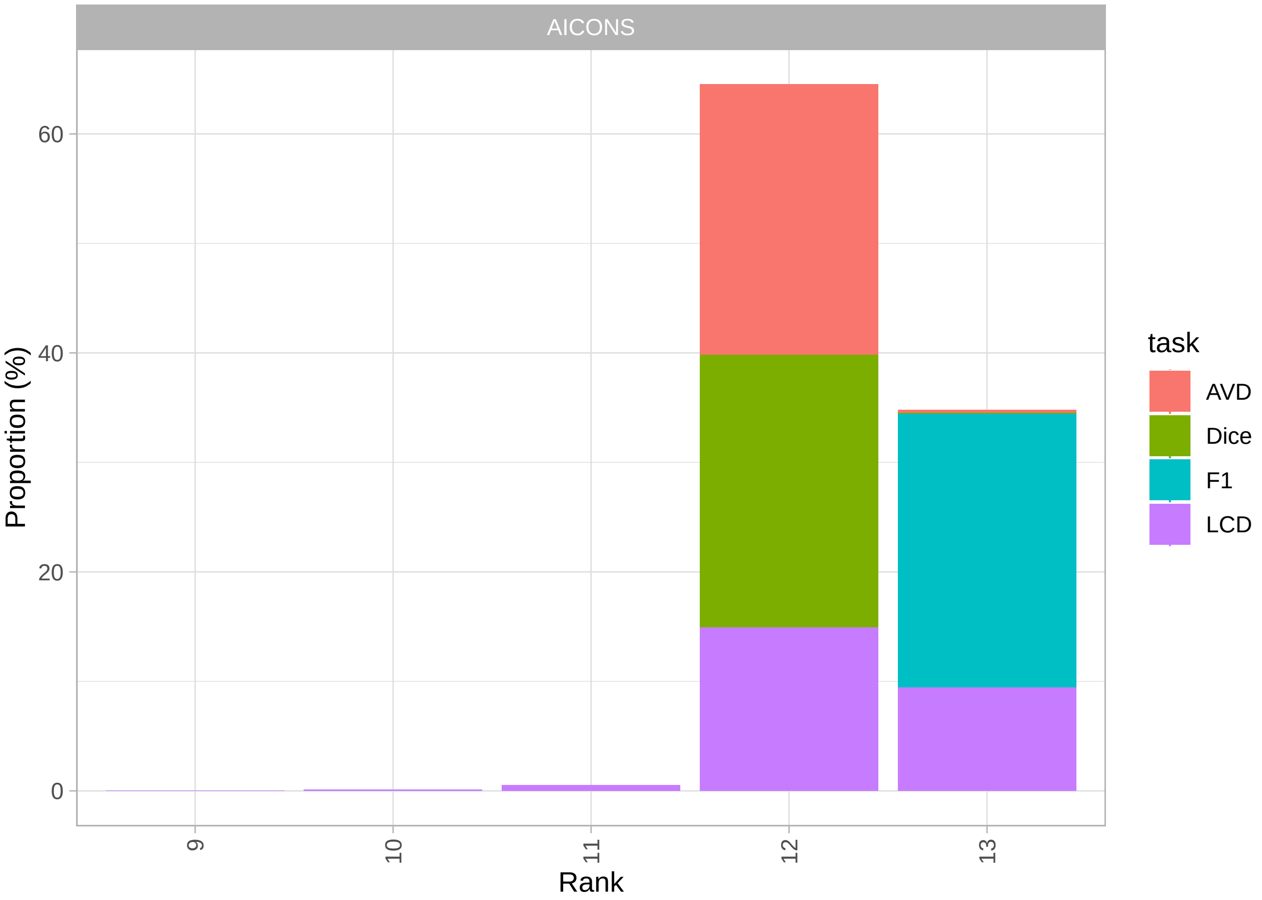

Figure S5.51: Stacked Rank Frequencies – AICONS.

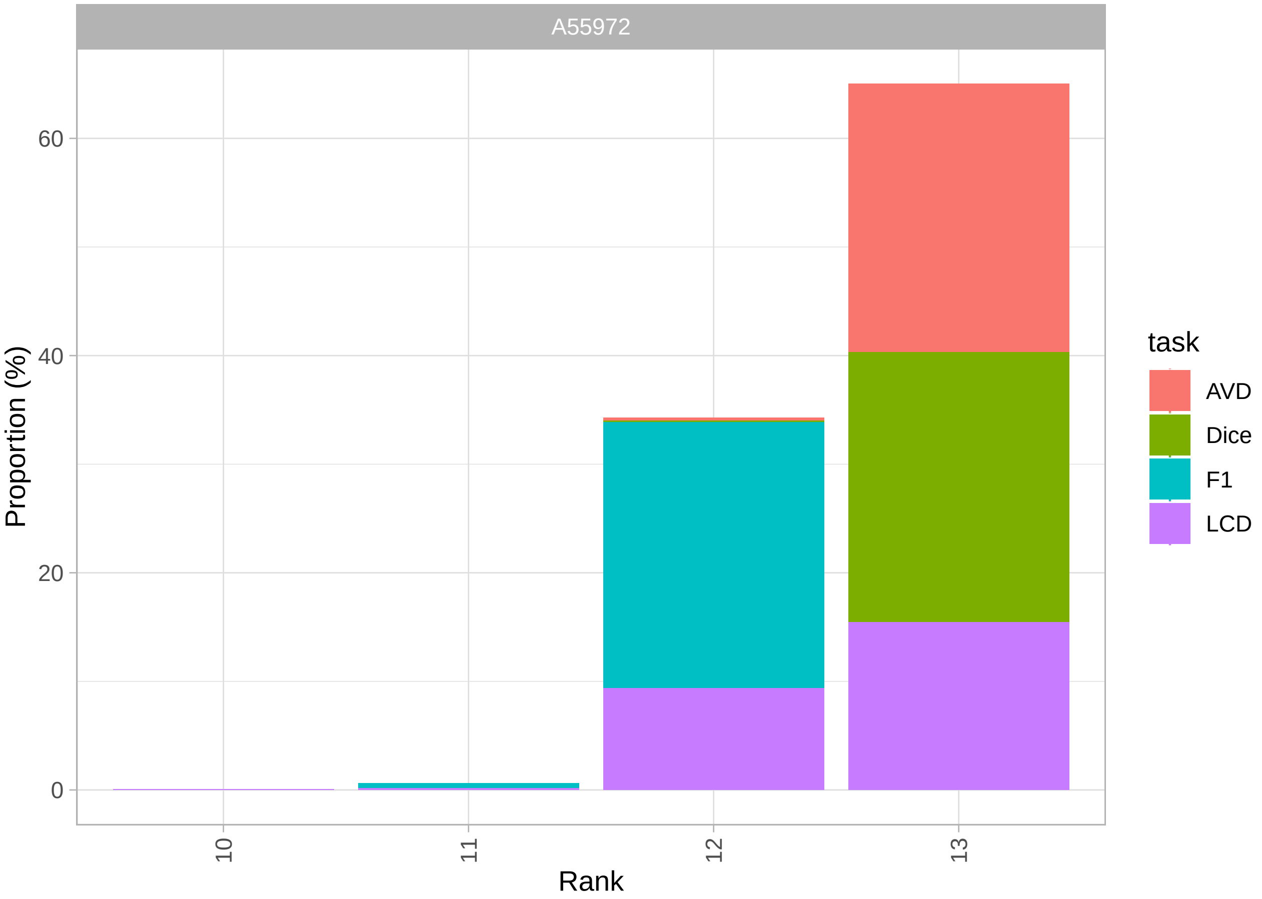

Figure S5.52: Stacked Rank Frequencies – A55972.

4.2 Characterization of tasks

4.2.1 Visualizing bootstrap results

To investigate which tasks separate algorithms well (i.e., lead to a stable ranking), a blob plot is recommended.

Bootstrap results can be shown in a blob plot showing one plot for each task. In this view, the spread of the blobs for each algorithm can be compared across tasks. Deviations from the diagonal indicate deviations from the consensus ranking (over tasks). Specifically, if rank distribution of an algorithm is consistently below the diagonal, the algorithm performed better in this task than on average across tasks, while if the rank distribution of an algorithm is consistently above the diagonal, the algorithm performed worse in this task than on average across tasks. At the bottom of each panel, ranks for each algorithm in the tasks are provided.

Same as in Section 3.1 but now ordered according to consensus.

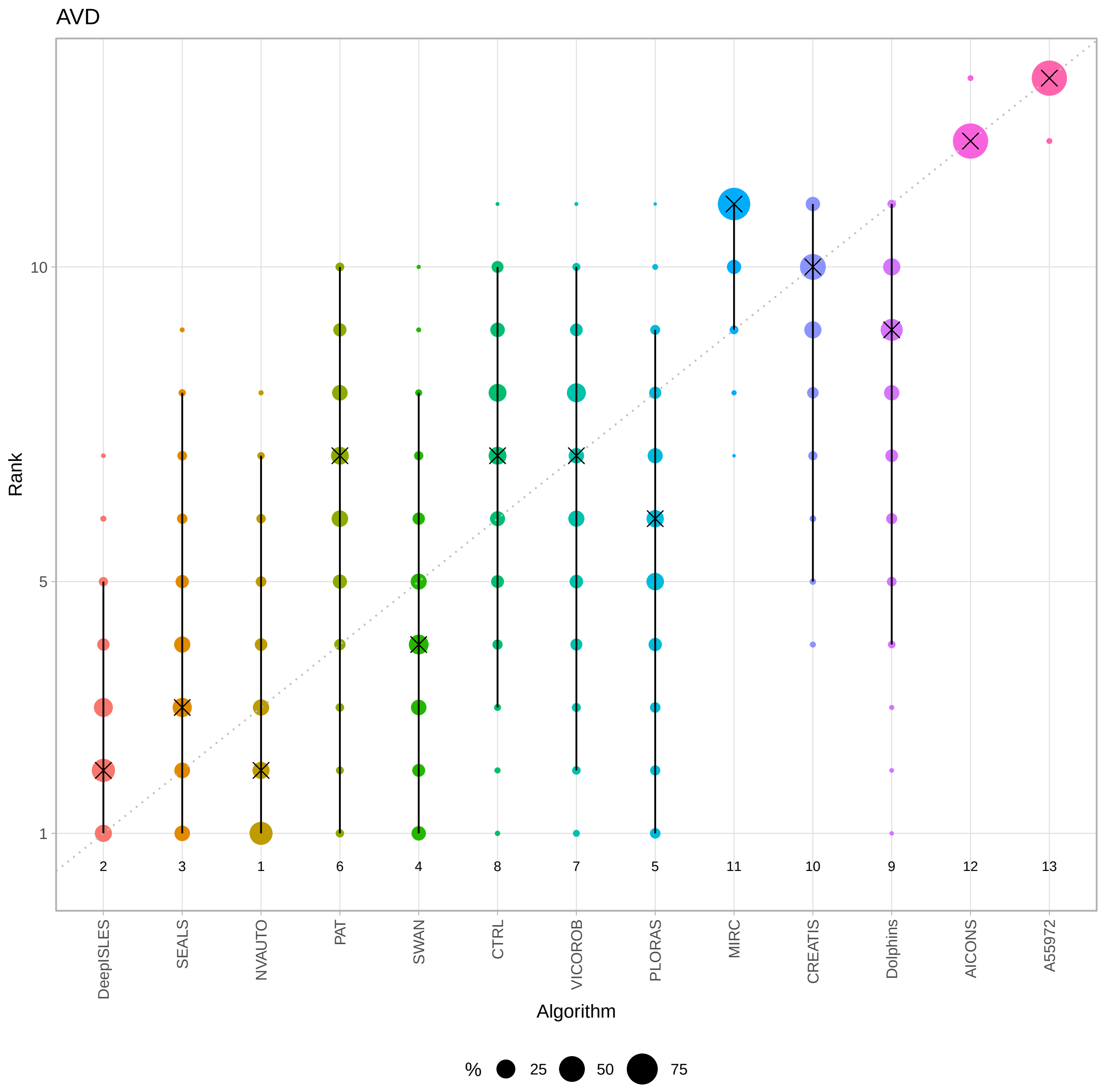

Figure S5.53: AVD Task-Level Ranking Stability – Blob Plot.

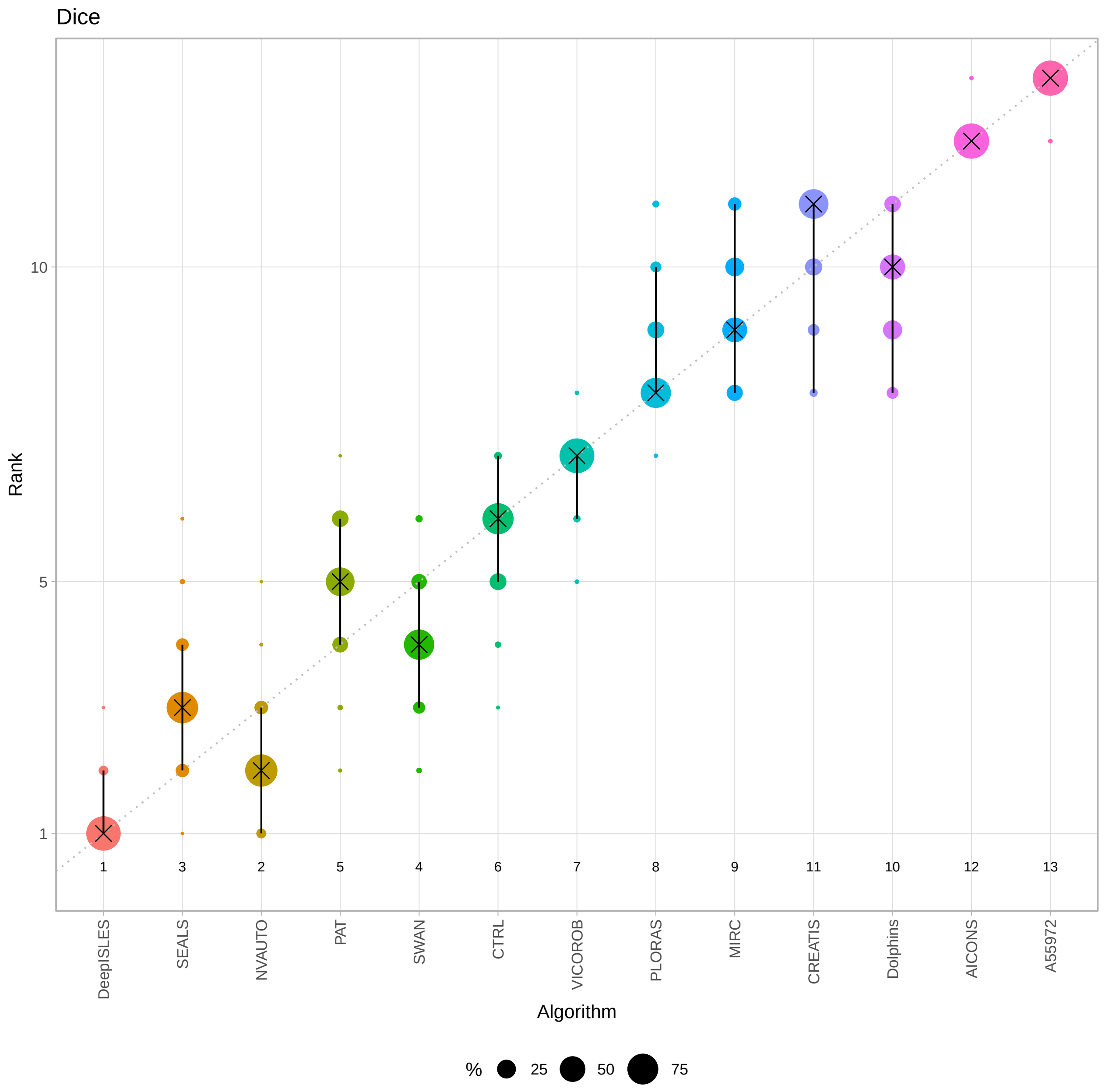

Figure S5.54: Dice Task-Level Ranking Stability – Blob Plot.

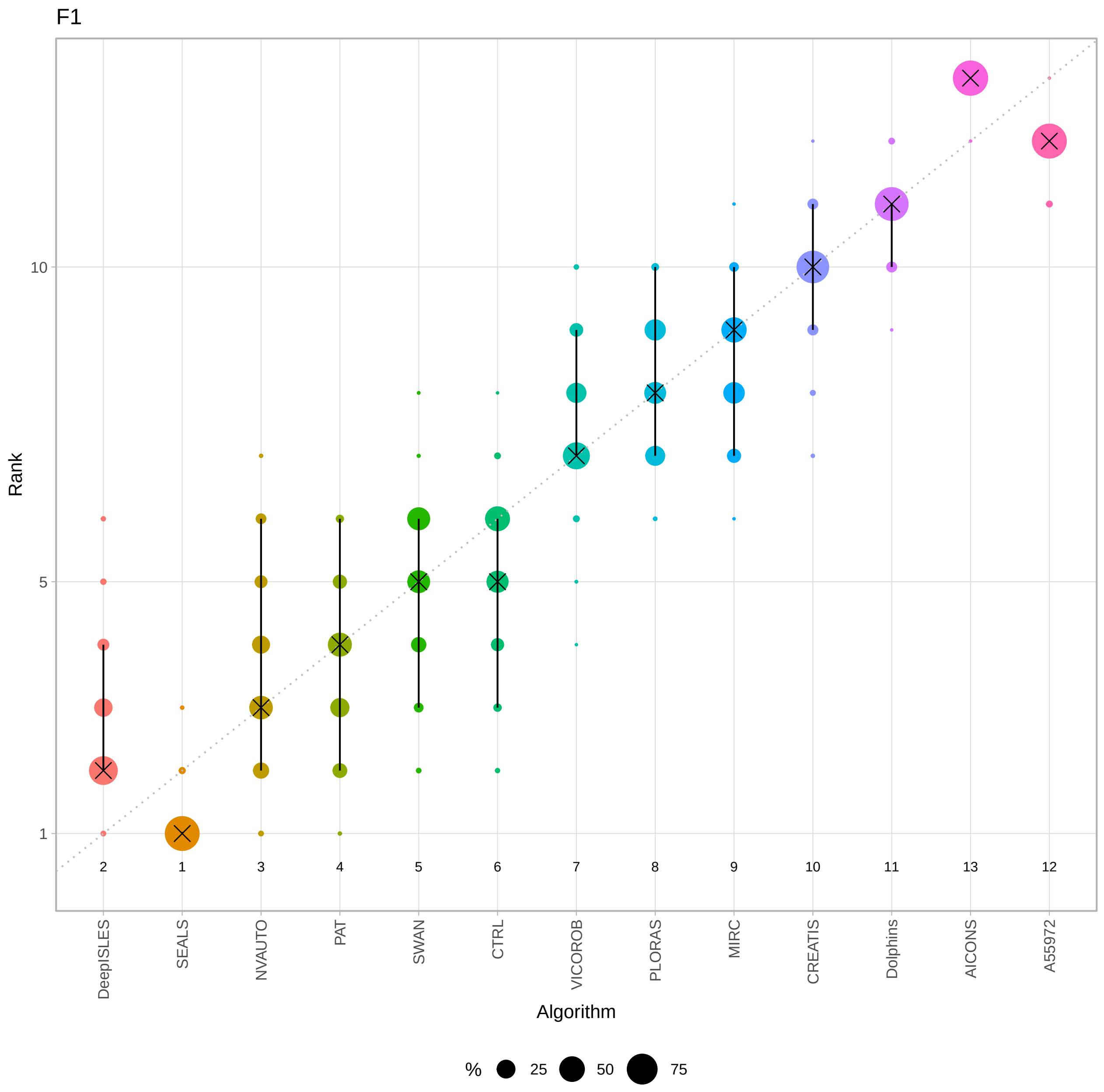

Figure S5.55: F1 Task-Level Ranking Stability – Blob Plot.

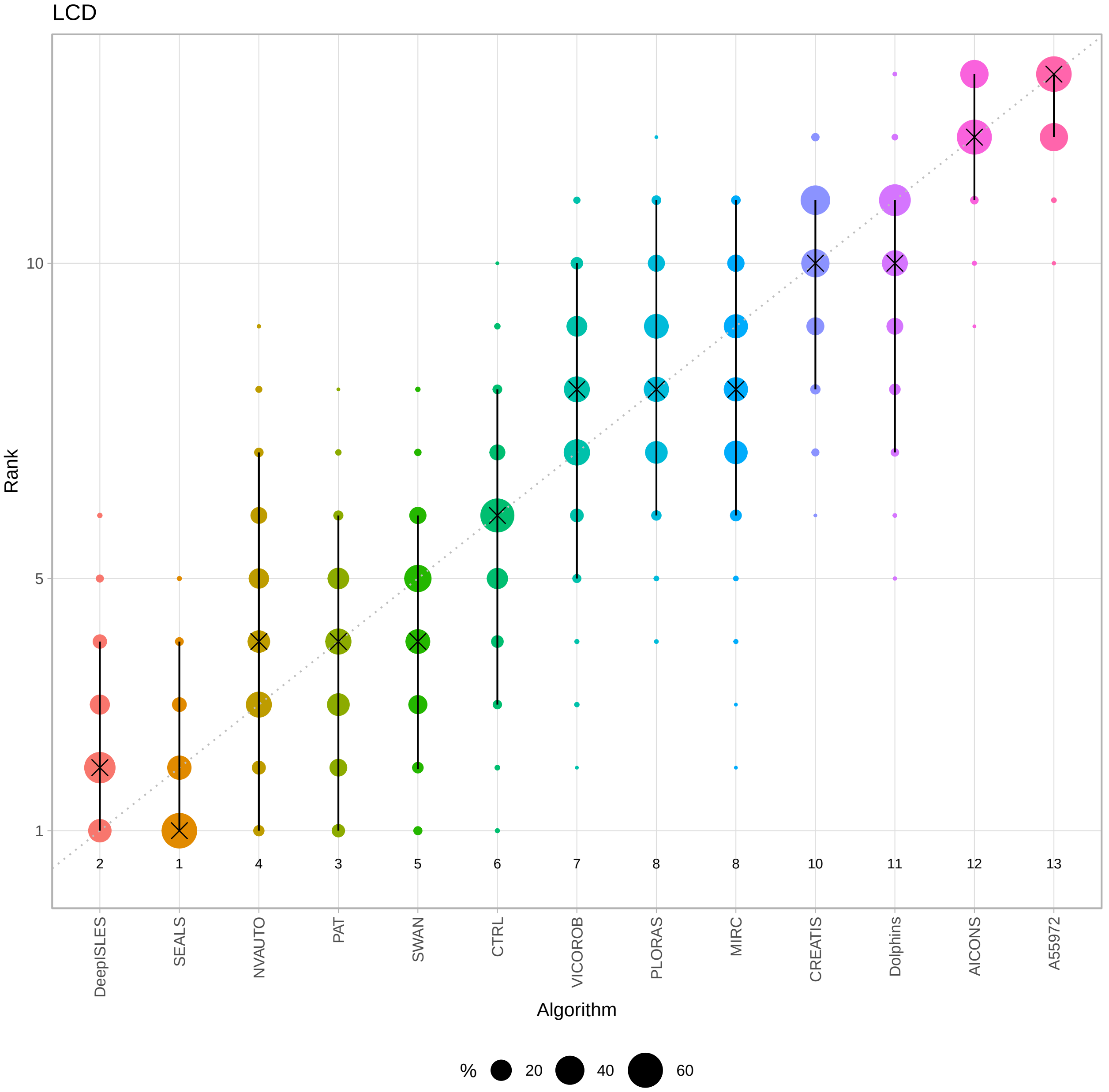

Figure S5.56: LCD Task-Level Ranking Stability – Blob Plot.

4.2.2 Cluster Analysis

Dendrogram from hierarchical cluster analysis and *network-type graphs* for assessing the similarity of tasks based on challenge rankings.

A dendrogram is a visualization approach based on hierarchical clustering. It depicts clusters according to a chosen distance measure (here: Spearman's footrule) as well as a chosen agglomeration method (here: complete and average agglomeration).

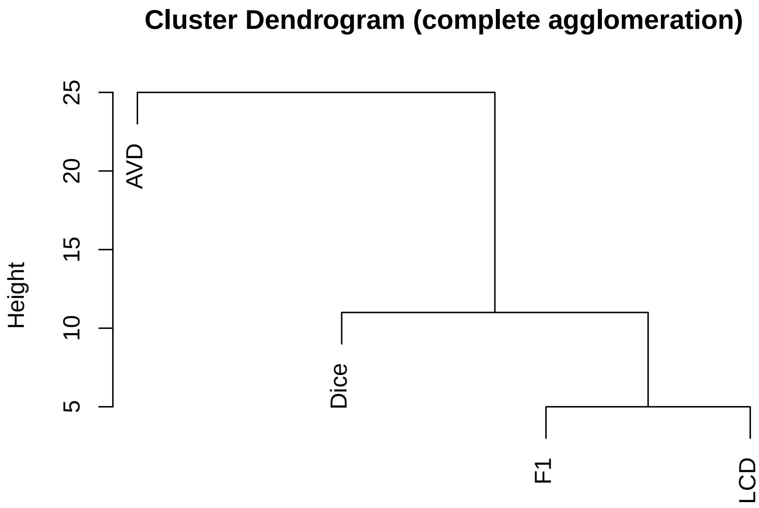

Figure S5.57: Cluster Dendrogram – Complete Linkage.

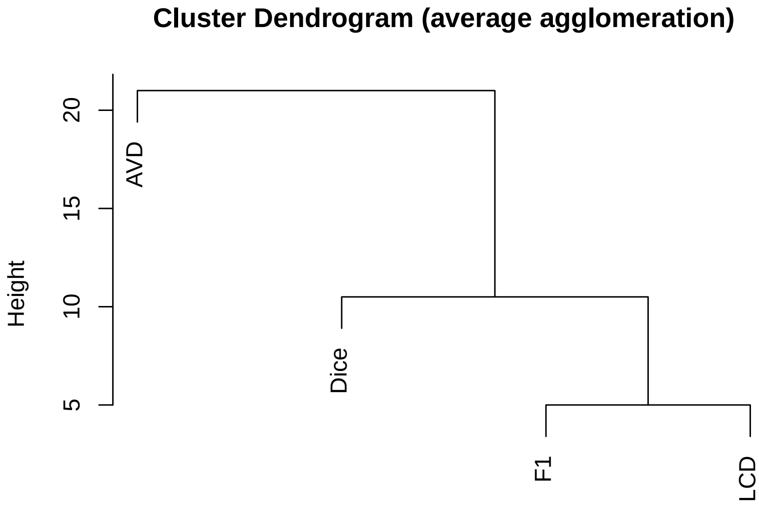

Figure S5.58: Cluster Dendrogram – Average Linkage.

5 References

Wiesenfarth, M., Reinke, A., Landman, B.A., Eisenmann, M., Aguilera Saiz, L., Cardoso, M.J., Maier-Hein, L. and Kopp-Schneider, A. Methods and open-source toolkit for analyzing and visualizing challenge results. *Sci Rep* **11**, 2369 (2021). https://doi.org/10.1038/s41598-021-82017-6

M. J. A. Eugster, T. Hothorn, and F. Leisch, "Exploratory and inferential analysis of benchmark experiments," Institut fuer Statistik, Ludwig-Maximilians-Universitaet Muenchen, Germany, Technical Report 30, 2008. [Online]. Available: http://epub.ub.uni-muenchen.de/4134/.

Supplementary material #6.

Deep learning versus experts in a Turing-like test:

RATING CRITERIA

Completeness of the lesion mask	
**Score**	**Criterion**
1	Lesion(s) fully missed, only false-positive segmentation (if any)
2	< 30% of lesion voxels segmented, and/or predominant false-positive segmentation
3	30% - 70% of lesion voxels segmented, and/or relevant false-positive segmentation
4	> 70% of lesion voxels segmented, and/or some false-positive segmentation
5	Lesion(s) almost perfectly segmented, very few false-positive voxels
6	Lesion(s) fully segmented, no false-positive voxels

Table S6.1.Completeness criterion for Turing-like scoring.

**Correctness of border delineations of correctly identified lesions.**<br>Completely false positive and false negative lesions are not taken into account.	
**Score**	**Criterion**
1	<30% of lesion(s) borders are correct
2	30%-70% of the borders are ok
3	>70% of the borders are ok
4	>90% of the borders are ok
5	Only single voxels of the border are off by not more than one voxel
6	All borders completely perfect

Table S6.2.Correctness criterion for Turing-like scoring.

Supplementary material #7.

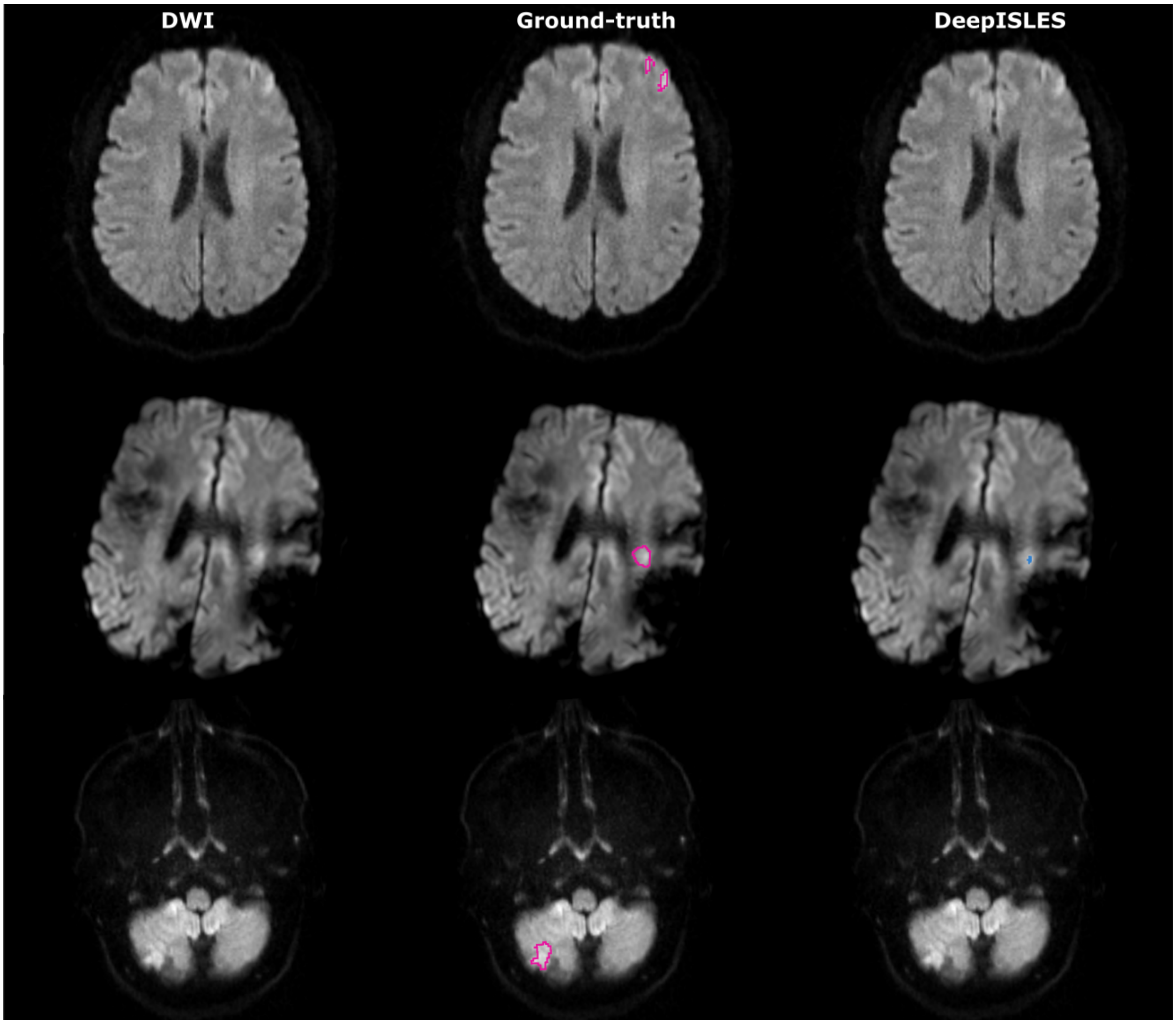

Figure 7.1. Examples of DeepISLES failure and suboptimal performance on challenging cases from the Johns Hopkins dataset.
